# Supplementary material for: Frequent Acquisition of Glycoside Hydrolase Family 32 (GH32) Genes from Bacteria via Horizontal Gene Transfer Drives Adaptation of Invertebrates to Diverse Sources of Food and Living Habitats
Source: Int J Mol Sci. 2024 Jul 30;25(15):8296. doi: 10.3390/ijms25158296 (PMC11311677; doi:10.3390/ijms25158296)
Supplement: Supplementary file 1 [file ijms-25-08296-s001.zip › ijms-3090238-supplementary.pdf]

Figure S1. Classification of animals with glycoside hydrolase family 32 (GH32) genes. The common names for each group of animals are given on the right side in red.

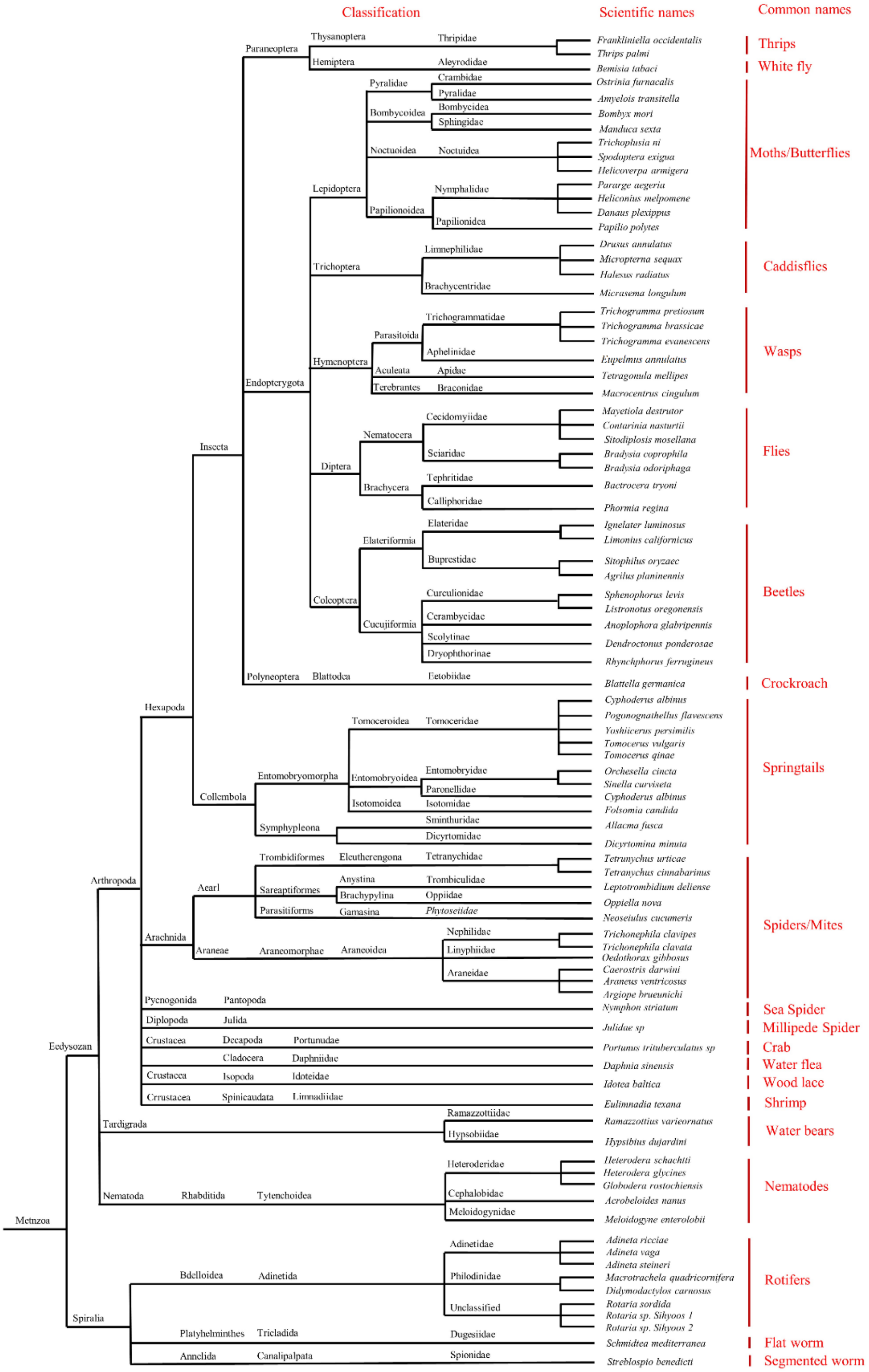

Classification
Scientific names
Common names
Metazoa
Ecdysozan
Arthropoda
Hexapoda
Insecta
Paraneoptera
Thysanoptera
Thripidae
Frankliniella occidentalis
Thrips palmi
Thrips
Hemiptera
Aleyrodidae
Bemisia tabaci
White fly
Endopterygota
Lepidoptera
Pyralidae
Crambidae
Ostrinia furnacalis
Pyralidae
Amyelois transitella
Bombycoidea
Bombycidea
Bombyx mori
Sphingidae
Manduca sexta
Noctuoidea
Noctuidea
Trichoplusia ni
Spodoptera exigua
Helicoverpa armigera
Papilionoidea
Nymphalidae
Pararge aegeria
Heliconius melpomene
Danaus plexippus
Papilionidea
Papilio polytes
Moths/Butterflies
Trichoptera
Limnephilidae
Drusus annulatus
Micropterna sequax
Halesus radiatus
Brachycentridae
Micrasema longulum
Caddisflies
Hymenoptera
Parasitoida
Trichogrammatidae
Trichogramma pretiosum
Trichogramma brassicae
Trichogramma evanescens
Aphelinidae
Eupelmus annulatus
Aculeata
Apidae
Tetragonula mellipes
Terebrantes
Braconidae
Macrocentrus cingulum
Wasps
Diptera
Nematocera
Cecidomyiidae
Mayetiola destrutor
Contarinia nasturtii
Sitodiplosis mosellana
Sciaridae
Bradysia coprophila
Bradysia odoriphaga
Brachycera
Tephritidae
Bactrocera tryoni
Calliphoridae
Phormia regina
Flies
Coleoptera
Elateriformia
Elateridae
Ignelater luminosus
Limonius californicus
Buprestidae
Sitophilus oryzaee
Agrilus planinennis
Cucujiformia
Curculionidae
Sphenophorus levis
Listronotus oregonensis
Cerambycidae
Anoplophora glabripennis
Scolytinae
Dendroctonus ponderosae
Dryophthorinae
Rhynchphorus ferrugineus
Beetles
Polyneoptera
Blattodea
Ectobiidae
Blattella germanica
Crockroach
Collembola
Entomobryomorpha
Tomoceroidea
Tomoceridae
Cyphoderus albinus
Pogonognathellus flavescens
Yoshiicerus persimilis
Tomocerus vulgaris
Tomocerus qinae
Entomobryoidea
Entomobryidae
Orchesella cincta
Sinella curviseta
Paronellidae
Cyphoderus albinus
Isotomoidea
Isotomidae
Folsomia candida
Symphypleona
Sminthuridae
Allacma fusca
Dicyrtomidae
Dicyrtomina minuta
Springtails
Arachnida
Acari
Trombidiformes
Eleutherengona
Tetranychidae
Tetranychus urticae
Tetranychus cinnabarinus
Sarcoptiformes
Anystina
Trombiculidae
Leptotrombidium deliense
Brachypylina
Oppiidae
Oppiella nova
Parasitiforms
Gamasina
Phytoseiidae
Neoseiulus cucumeris
Araneae
Araneomorphae
Araneoidea
Nephilidae
Trichonephila clavipes
Trichonephila clavata
Linyphiidae
Oedothorax gibbosus
Araneidae
Caerostris darwini
Araneus ventricosus
Argiope bruennichi
Spiders/Mites
Pycnogonida
Pantopoda
Nymphon striatum
Sea Spider
Diplopoda
Julida
Julidae sp
Millipede Spider
Crustacea
Decapoda
Portunidae
Portunus trituberculatus sp
Crab
Cladocera
Daphniidae
Daphnia sinensis
Water flea
Crustacea
Isopoda
Idoteidae
Idotea baltica
Wood lace
Crrustacea
Spinicaudata
Limnadiidae
Eulimnadia texana
Shrimp
Tardigrada
Ramazzottiidae
Ramazzottius varieornatus
Hypsobiidae
Hypsibius dujardini
Water bears
Nematoda
Rhabditida
Tylenchoidea
Heteroderidae
Heterodera schachtii
Heterodera glycines
Globodera rostochiensis
Cephalobidae
Acrobeloides nanus
Meloidogynidae
Meloidogyne enterolobii
Nematodes
Spiralia
Bdelloidea
Adinetida
Adinetidae
Adineta ricciae
Adineta vaga
Adineta steineri
Philodinidae
Macrotrachela quadricornifera
Didymodactylos carnosus
Unclassified
Rotaria sordida
Rotaria sp. Silwood 1
Rotaria sp. Silwood 2
Rotifers
Platyhelminthes
Tricladida
Dugesiidae
Schmidtea mediterranea
Flat worm
Annelida
Canalipalpata
Spionidae
Streblospio benedicti
Segmented worm

Figure S2. Amino acid sequences with names and protein accession numbers. Putative secretion signal peptides are bold and italic. Three active site regions are bold and in red, green, and blue colors for regions 1, 2, and 3, respectively. Information on active site regions either not detected or key residue substituted is given in parenthesis.

Thrips (Thysanoptera, Insecta, Arthropoda)

Frankliniella occidentalis

>Focc_GH32_1, XP_026287851 (**active site region 1 not detected**)
MSWGHAVSTDMVHWTELPLAIPYTADEQIFSGSVVVDWACSSGLCATPDMPVLIAMYTSFSQHEDANGKHIQSQHIA
SSNDLGRTWLKYAHNPVLDKND**PEFRDPKVQ**RYGDSWLVLVQRSTEHKLEFYKSTDLKNWGDAVGSFGPAGET**SGVW**
ECPDMFELAVDGDPNNKKWVLIVNINPGNRVAGSAGQFFIGDFDGKEFKAETTTDWLDWGMDNYATITWDNAPDGQR
FSIGWMSNWIYTELAPTGPNFRNTFTSPRLLKLQTIDGKVRLTQMPVNYLDSIRSGPVKQEDKVTLKDASHKKDLRG
RAVDIEVVFTNTDATEFGVRVHAGENQETLVGYDVATGKAFVDRTKSGTVNFHEKFPGRHDAPFALQSGSKLKLRVL
VDHGSVEAFFGEGEIAITDVVFPDPTKDAIEFYAKGGSVVIESYSVWQMKETHHLV
>Focc_GH32_2, KAE8746860
MRPAVSHTLVGAVVAAVVAGITVAIMYSTLPLCDEEDTVSPSPPPSPPPSPSPPPPPLDPLRPAYHYTPKSG**WVNDP**
NGLVYYKGEYHLFYQHDYETILHGNMSWGHAVSTDMVHWTELPLAIPYTADEQIFSGSVVVDWACSSGLCATPDMPV
LIAMYTSFSQHEDANGKHIQSQHIASSNDLGRTWLKYAHNPVLDKND**PEFRDPKVQ**RYGDSWLVLVQRSTEHKLEFY
KSTDLKNWGDAVGSFGPAGET**SGVWECPDM**FELAVDGDPNNKKWVLIVNINPGNRVAGSAGQFFIGDFDGKEFKAET
TTDWLDWGMDNYATITWDNAPDGQRFSIGWMSNWIYTELAPTGPNFRNTFTSPRLLKLQTIDGKVRLTQMPVNYLDS
IRSGPVKQEDKVTLKDASHKKDLRGRAVDIEVVFTNTDATEFGVRVHAGENQETLVGYDVATGKAFVDRTKSGTVNF
HEKFPGRHDAPFALQSGSKLKLRVLVDHGSVEAFFGEGEIAITDVVFPDPTKDAIEFYAKGGSVVIESYSVWQMKET
HHLV
>Focc_GH32_3, KAE8748783
MAPWTHIVVLAALALARAGARMPPDARALHEDFKGQALAGAPPPEAKAAAAAAALAEEAATTPAADEEECLTAPFPR
PLVHFTPKT**NWINDPNGL**LYAGGEWHLFYQFSPLSPLPRNISWGHAVSRDLVRWTELSSEGKPAIPFDGDRELIFSG
SAVLDQDNTSGLGTTENPPMVAIYTSHNASKQAQSLAYSLDAGRTWTKYAGNPVLDENE**KEFRDPKVQ**WHEPTRRWL
MAVAMPDERKVRFYSSPDLKAWTLLSAFGPMGAV**AGQYECPDL**FPLPVDGDLARIKWVLIVNVNPGGLQGGSSGQYF
IGDFDGERFVPDNKDPAAVRWLDYGKDYYAAISWVGAPDGKRYMIGWMSNWLYATQTPTSPWRNSMSVPRVMSLRSR
AGAPDGVDLVQEPVPALEATLRPMQMAPVSITVRRRPMLLYSGCNGAYYLEVTVTLGTGATGVSILLRASPNFTSGT
DVTWNANKGELSVDRRESGATDFSPDFPGVHVAPLARDTLPDGKLKLKILVDEGSVEVFADGGRVAITDLIFPKDED
NLIVVKTNGPGEATFGNLTVTPLRPYRESCQAKESTCRLLDRPHEI
>Focc_GH32_4, XP_026273122
MSFGVVAGKRGGGGVPLQVLEKQRDPPPRWKWCGVASGEAVSKFEFAKRSTEIHINDFTVEDRREEAMAVVRCPGRV
PEDAQEAGRGAERAAAAAALAEEAATTPAADEEECLTAPFPRPLVHFTPKT**NWINDPNGL**LYAGGEWHLFYQFSPLS
PLPRNISWGHAVSRDLVRWAELSSEGKPAIPFDGDRELIFSGSAVLDQDNTSGLGTTENPPMVAIYTSHNASKQAQS
LAYSLDAGRTWTKYAGNPVLDENE**KEFRDPKVQ**WHEPTRRWLMAVAMPDERKVRFYSSPDLKAWTLLSAFGPMGAV**A**
GQYECPDLFPLPVDGDLGRIKWVLIVNVNPGGLQGGSSGQYFIGDFDGERFVPDNKDPAAVRWLDYGKDYYAAISWV
GAPDGKRYMIGWMSNWLYATQTPTSPWRNSMSVPRVMSLRSRAGAPDGVDLVQEPVPALEATLRPMQMAPVSITVRR
RPMLLYSGCNGAYYLEVTVSLGTGATGVSILLRASPNFTSGTDVTWNANKGELSVDRRESGATDFSPDFPGVHVAPL
ARDTLPDGKLKLKILVDEGSVEVFADGGRVAITDLIFPKDEDNLIVVKTNGPGEATFGNLTVTPLRPYRESCQAKES
TCRLLDRPHEI

Thrips palmi

>Tpal_GH32_1, XP_034232383
MRPLVANVVVGVVAAVVAAAVTAGIILGTLPGCADDAPAALAVDMMRPAFHYTPRF**GWVNDPNGL**VYADGEWHLFYQ
ADYETTLHGNMSWGHAVSTDLVHWQELGVAISYDQHEQIFSGSAVVDKDCTSGLCAKAGEPVMIAIYTSYDQVPDPN
SPLDDQGHSTNRHFQSQHIASSTDRGRTWTKYAHNPVLDKKM**YEFRDPKVS**RRGDHWLMLVQKSEEHVLQFYKSSDL

IKWGDGKGGDGPAGEFTGLGAT**GGVWECPDM**FELPVDGDAKNTKWVLIVNINPGSRFGWGSAAQFFIGQFDGDTFHA
DGDYSWLDWGADNYATITWDSAPNGEVVAIGWMSNWQYTQVTPASTWRNAFTVPRLLELVTIDGKVRVVQNPVKNID
SLRDLSNAVHQSNLAVANDYTEPAIRGRALDIVVEFDAGSAAEFGVKVYQGEHQETLIGYDKAKGQVFIDRRISGTV
NFDALFPDRHSADLPLVGNKLKLRILVDHGSVEVFANRGEVAITDVIFPDPFKDGVSFYALNGTATVVSVDVYPMKT
IHGLL
>Tpal_GH32_2, XP_034233419
MCPSEPFRPHVHFTPRA**LWMNDPNGL**VYAAGEWHLFYQFNPVSDVPGNISWGHAVSPDLVRWTELTGEGNPAIPYDG
DTELIFSGSAVVDQDNTSGLGTPENPPMVAIYTSANATKQAQSLAFSLDNGRSWTKYEGNPVLDEEQ**KEFRDPKVQ**W
HTPTRRWLMAVALADDRKVRFYSSPDLKEWTQLSEFGPMGAV**EGQYECPDL**FPLPVDGDLERLKWVLVVNVNPGGLQ
GGSSAQYFVGDFDGERFVADDQDPKAVRWLDYGKDYYAAVSWVGAPDGKRYMIGWMSNWQYAAKTPTSPWRNAMSVP
RVMSLRSRPDGGVDLLQEPVPALASTLHRTAEPIMTAATLNVSAGGAAVVANGCDGAFLLEVTVSLVDGATGATVLV
RAAEDGSSGTGIVWDSEKGELAVDRRKSGLVDFSADFPGVHGAPLPYASLADGKLRLTVLVDEGSVEVFADGGRVAI
TDIVFPDKERKAVVLRAGGAGGAEGERGSVVFADVRVVPLRQYREGCAPREKTCRLAL

White fly (Hemiptera, Insecta, Arthropoda)

Bemisia tabaci

>Btab_GH32_1, VMEI01003277
MNDPNGLVIKDHEFHLFYQYDPYAPIIGNVHWGHAVSGDLVRWVTLPVAINETADGQAFSGSAVIFNNSLIAAIYTR
ASETKQAQELAFSSDNGRTFQEYKGNPVLDRNS**DSFRDPQVI**RGSFQKTHEYKMTVVKARKHQVLIYGSRDLVNWRE
LGSFGPAGIL**GIDYECPNL**MRVPTEDGHYKWVLAISINPGSPLGGSGTQYFVGDFDGTTFTPDYYETKFVDFGKDFY
ALQTYSNARAPLGIAWLSNWQYANFTPTGDWRGVMTLARGFGLRYTEDFKYFLTQKPVGLENLYRREIPVGAESCSV
PGDKALEIHAIVELRPRSRISFSLLSENHEQLVWGYDANAGQAWIDRGRTFGFSQRFFTDKMSVAVIPGTTNIDLHA
IFDKSTFELFIDDGTFVGTCLVFFEKLPNCLRYSLVGDGRVTNLTVNVLTP

>Btab_GH32_2, VMEH01001658
MNDPNGLVIKDHEFHLFYQYDPYAPIIGNVHWGHAVSGDLVRWVTLPVAINETSDGQAFSGSAVIFNNSLIAAIYTR
ASETKQAQELAFSSDNGRTFQEYKGNPVLDRNS**DSFRDPQVI**RGTHEYKMTVVKARKHQVLIYGSRDLVNWRELSSF
GPAGIL**GIDYECPNL**MRVPTEDGHYKWVLVISINPGAPLGGSGTQYFVGDFDGTTFTPDYYETKFVDFGKDFYALQT
YSNARAPLGIAWLSNWQYANFTPTGDWRGVMTLARGFGLRYTEDFKYFLTQKPVGLENLYRREIPVGAESCSIPGDK
ALEIRAIVELRPRSRISFSLLSENHEQLVWGYDANAGQAWIDRGRTFGFSQRFFTDKMSVAVIPGTTNIDLHAIFDK
STFELFLDDGTFVGTCLVFFERLPNCLRYSLVGDGRVTNLTVNVLTP

>Btab_GH32_3, VMEG01001706
MTVVKARKHQVLIYGSRDLVNWRELSSFGPAGILGIDYECPNLMRVPTEDGHYKWVLVISINPGAPLGGSGTQYFVG
DFDGTTFTPDYYETKFVDFGKDFYALQTYSNARAPLGIAWLSNWQYANFTPTGDWRGVMTLARGFGLRYTEDFKYFL
TQKPVGLENLYRREIPVGAESCSVPGDKALEIRAIVELRPRSRISFSLLSENHEQLVWGYDANAGQAWIDRGRTFGF
SQRFFTDKMSVAVIPGTTNIDLHAIFDKSTFELFLDDGTFVGTCLVFFEKLPNCLRYSLVGDGRVTNLTVNVLIP

Moths/Butterflies (Lepidoptera, Insecta, Arthropoda)

Ostrinia furnacalis

>Ofur_GH32_1, XP_028169311
MAFLHQLVILIIAVAVSLQSPKRRTSKSELADYIAHKREEVNQRYKLLYHVTPPV**GWMNDPNGF**SFYKGEYHLFYQF
YPYDSVWGPMHWGHSTSQNLVDWKEQPTALIPGIEQCFSGSGVVDGDQLVLMYTGHEDRDTPPYYRETQYLAFSNDG
INFEKYEGNPVLLPSGS**PDFRDPKVW**RHGDHWYVVLGSKSDDLQRGRVLLYRSTDLKNWEFLSVIGESDGTL**GYMWE
CPDF**FELDGKYILLMSPQGMEPQGDRYKNTFQTGYIIGNFNYETFEFVPEVGFQEIDYGHDFYATQTIGNDGKRYVV
GWFGMWEVPHPEAADGWAGAMTIIRELKLVGDRIIQTPVEGMINLREQGNRISLAPYNVVEFEKTAEIIVNPDLNQA

IELQVEGKYGGGGKIWLKWDPAVRKVIIDRGSYDIRQVEWAPIGSHSWRIFLDASSVELFCGEGEAVFSSRVYPDGD
WKVTNLSPQTLDIETYHLRKSITELN
>Ofur_GH32_2, XP_028169317 (1)
MAFLYQLVLVLLIAVAVSSQTSKETNKTELAQYIADKRETINQRYKLLYHVTPPV**GWMNDPNGF**SFYKGEYHLFYQF
YPYDSVWGAMHWGHSTSPNLVDWKEQPTALIPEKEQCFSGSGVVDGDQLVLMYTGHEDRDTPPYYRETQYLAFSNDG
INFEKYEGNPVLLPSGS**PDFRDPKVW**RHGDHWYVVLGSKSDDLQRGRVLLYRSTDLKNWEFLSVIGESDGTL**GYMWE
CPDF**FELDGKYILLMSPQGMEPQGDRYKNIFQTGYIIGNFNYETFEFVTEVEFQEIDFGHDFYATQTIDNDGKRYMV
GWFGMWEVPHPEAVDGWAGAMTIIRELKLVGDRIVQIPVEAMVTLREQGTTTSWAPNTTIEFQKTGEIIVNGTLNQT
IELLIEGKDGGEKAWLTWDPVVGKVIVDRGSKSNDTRQVEWAPISSHSWRIFLDASSLELFCGEGEVVFSS
>Ofur_GH32_3, XP_028169317 (2)
MALLHQLVVSFCVIAAVTSKSFRQHDSVLEVEEYIANKKADINPRYRLHYHVSPPV**GWMNDPNGF**SFYKGEYHLFYQ
FYPYDSVWGPMHWGHVSSPNLVEWQQLPTALIPEQEMCFSGSGVVDGDQLVLMYTGRINLDVEPFYNETQYLAYSDD
GVTFQKYEGNPVLPAAPNGS**PDFRDPKVW**KYEDHWYVVIGSKTDDERGRVLLYRSPDLKTWEFLSIIGESQGDM**GYM
WECPDF**FELDGKFILLMSPQGLESQGDRYKNTYQTGYIIGSFNYETFEFVPEVEFQEIDFGHDFYATQSMEKDGKRY
VIGWFSMWDVPLPEDVDGWAGTMTIVRELNLVGDRVIMKPIDEMVILREQNLFTGALEPNSVVEFEKTGEIIVNGDL
SQNIELLIEGSNGGGKAWVRWDAGEGKVIVDRGSDDVRQVEWEPIKSHSWRIFLDASSLELFCGEGEVVFSSRVYPD
GDWRVTNLSPQTLDIEAYHLRRSVPE
>Ofur_GH32_4, XP_028161384
MGYLPQFSIICCLLIGIKAAVNDRYYPRYHLAPPQ**GWMNDPNGF**SVFNGEYHLFYQYNPLSSLEPGIAHWGHAVSED
LFHWEHLPIAMYPNNTYDSSGVFSGSALIEDDIMYLFYTGNVNHPNEQPNHEQRQALAESTDGIHVTKYENNPIIMA
DDRQ**PNIRDPKVW**KHGSKYYMVLGNSFENDTLGRALLYSSDDKINWEEISVLHESDGSL**GFMYECPDF**FKLNGKYVL
LFSPQGIEPQGDKYRNLYQTGYIVGNFNYETNEFHPITSFQELDHGHDFYATQTIKDNRGRRLLVAWFDMWERDYPQ
AQDGFTGQMTIVRELHLTRDNKLIQEPVSEVKAVRANRVRRGKGRVGDVVTFEDKAGEIDILGDSSEDLEVVIEGEG
STVTISYDASNGTVTLDRGGDDGVRRTEWKPSGSILKWTIYVDASSIELFCGKGEVTFSSRFFPDGEVSARISSGCP
DKFAVYNLKRTVQSPEDETER
>Ofur_GH32_5, XP_028174410 (**D in active site region 1 substituted to S, D in
active site region 2 substituted to N, but there is an adjacent D**)
MEVLLLTVLLSVAAAEFAQDLAHVEAFIEQKRYVLKKKYRPLYHISAPV**GWLNSPAGF**AYFKRRYHIFYQYHPYNGA
WGTMHWGQAISDNLIDWVHYPPAVLPKDFYDRHGCLAGTAVMHNNYLTLFYTGVVQTQNQTIQTQNIAISGDGIIFQ
KYLYNPVIRQYPANV**TDFRNPKVW**RFRNKWFMILGSATENRQGQLILFSSPDMFHWKYNRTLARSYGDM**GYMWENPD
L**FEVDGQHVLILSVQGIQAESYRFRNLYQTGYVVGNFNYHSLRFENLEVSMATFTELDYGHDFYAAHTLQAYDGRRL
LIAWLGMWESELVESRDGWASMMTLIREVRLSPQGRLLMTPIKEMVDLRTEILEDAWYSPGEAFHAGSKSFELIVNA
SSVLYDAAVTFEWHGERQYAIAYSAERGRITVDRGGIDGIRKADWSPTGHMHWRIFVDSSSIEVFCGDGEVVFSSRI
YPKRGIQIRIGGEMQLHVTQYKIRRSVGYDEKLRQYLKHNFVNRIKY

Amyelois transitella

>Atra_GH32_1, XP_013198851 (**D in active site region 1 substituted to A, D in
active site region 2 substituted with N, but there is an adjacent D**)
MEALPLIVWLALVAVICYEAHENVDEFMEQNKVLRFTYRKKYRPAYHISAPM**GWLNAPSGF**VHFKQEYHIFYQYYPY
NGAWGTMRWGHVVSKNLVDWYYASPALIPKEYYELHGCFSGSAVVENEYLTLFYTGVRIMFNITTRTQNIAVSTDGV
YFEKHLHNPIIRKNR**DDARNPKVW**KFRNNWYMVLASTSRTGTPRITLYISEDLFNWNYNGTIVESLGDM**GTLWEHPD
F**FEMEGQYVLTFTVRGLVPDIDRFKNIYHTGYVLGKFNYLTAKFEDVEISMATFNELDYGHDFYAAHSMQALDGRRL
MVGWLGMWQSDFEELRDGWAGMLTLIREVSLSENEKILLQPLREIVDLRLELLESAFYHPGETFQAGTKYFELLLNA
STTINDVGLTFDWGGDKQFVIKYLSKRGQILIDRGGTDGVRRADWVPDRQIFWRIFMDSSSVELYCGEGEVVFTSRI
YPKKGLIVKIEGEQMVHIVQHKLRRSVSYSEHLREHLREMHELTLQNNVDNRKFNGFCK
>Atra_GH32_2, XP_013192327
MKYLFNMSLKIIVTLLVLLTIVQAKLLRTTEQQKQELADYIKQKKTEINPRFRLRYHVSPPV**GWMNDPNGF**SYYKGE
YHIFYQFYPYDSKWGPMHWGHSSGPNLVIWNEQPTALIPDEEQIFSGSAVTEGNTMVLMYTGHKTNNESQYLAYSKD
GVNFTKYEGNPVITLDTS**PDFRDPKIW**KYGDYWYVVIGTKTSDQKYGKVVLYRSSNLTSWEYLSVIGESDGAM**GYMW
ECPDF**FELDGKFVLLMSPQGMKAQGDRYKNTYQTGYILGNFSYETYEFIPETEFVEIDYGHDIYATQTLEKDGKRYL
IAWFGMWDVTHPEEADGWTGALTIVRELKLVGNRILMQPVEAIESLRDKTVFIGYFNKSQFLEFDTTAEIIVEGDMN

LKIELLLEGRKNNSNFNQTRVLYNPADGKVSVRRGNEDVRQVEWVPIGSKQMRLFLDTSSLELFCGDGEVVFSSRLY
VDGPWKVTNVSPQTLKIEAYHLKDSVPASSLGIVAVSINYVVCVLMPLVSLTLMK

>Atra_GH32_3, XP_013192330
MNLCFSTMALTKLLVTAFFLSVVNAKLVRQSDEVKNELEEYVQNKKAEINPRYRLHYHVMPPV**GWMNDPNGF**SYYKG
EYHIFYQYYPYDSIWGPMHWGHSSSPDLVNWKELPTALIPEEEMCFSGSAVVDGDSLILIYTGRINLEGDNYNESQY
LAVSDDGINFYKHVGNPVLALPSGGS**PDFRDPKVW**KHGDYYYVVIGTTTNNQQTGIVVLYRSSDLISWEYLSVIGES
GDDL**GYMWECPDF**FELDGKFVLLMSPQGVEPQGDRYKNLYQTGYIIGSFDYETFQFVPEVDFQELDYGHDFYASQTM
EHDGKRYVLGWFSMWEQPHPEKDDGWSGALTMVREIKLVDNRILMSPVEAMKQLRNDTVLIGDFNEGQVLSFDNTAE
IIVEGDLEYKIELLIEGRNGGDQALITWDPAVGKVAIDRGSDDVRQVEWSPIGSKSWRLFLDTSSLELFCGEGEVVF
SSRLYVEDPWNVINLSPQSLKIESWHLKRSVPEP

>Atra_GH32_4, XP_013197813
MTPTHKHLIFALLFYICKAEVDDEFYPRYHLAPPH**GWMNDPNGF**CFFQGEYHLFYQYNPISSLEPGIAHWGHAKSKD
LFRWEHLPIALYPDEWYDKSGVFSGSATVENDTMYLLYTGNLNHPNEDPDHEQHQALAYSTDGVNVTKYEGNPVIDS
LEHQ**PNIRDPKVW**KHEDTYYMVLGNSFNNGTLGRALLYSSEDLKSWAEVSVLDESDGAL**GYMWECPDF**FELDGKFIL
LFSPQGVEPEGNKYRNLYQTGYIVGDFDYDSHKFTPITEFVELDHGHDFYATQTILDHYNRRLLVAWFDMWEQNYPE
RDDGWTGQMTIVRELFLTKDNHLIQKPVAEVMNARGKLLYSARNTKGSKSVELSDKTGEIRVKAKASSDINLFIESK
NQSATVKISYDHKQGLVTLDRGGNDGVRQTEWKPKEKKLRLRIFVDASSIEVFCGRGEVTFSSRFFPEDNVAVRLGE
NTEVEQMIVTAMRRTVKRPDSTE

>Atra_GH32_5, XP_013200229
MTPTHKHLIFALLFYICKAEVDDEFYPRYHLAPPH**GWMNDPNGF**CFFQGEYHLFYQYNPISSLEPGIAHWGHAKSKD
LFRWEHLPIALYPDEWYDKSGVFSGSAIVENDTMYLLYTGNLNHPNEDPDHEQHQALAYSTDGVNVTKYEGNPVIDS
LEHQ**PNIRDPKVW**KHEDTYYMVLGNSFNNGTLGRALLYSSEDLKSWTEVSVLDESDGAL**GYMWECPDF**FELDGKFIL
LFSPQGVEPEGNKYRNLYQTGYIVGDFDYDSHKFTPITEFVELDHGHDFYATQTILDHYNRRLLVAWFDMWEQNYPE
RDDGWTGQMTIVRELFLTKENHLIQKPVTEVMNARGKLLYSARNIKGSKSVELSDKIGEIRVKAKASSDINLFIESK
NQSATVKISYDHKQGLVTLDRGGNDGLRQTEWKPKEKKLRLRIFVDASSIEVFCGRGEVTFSSRFFPEDKVAVRLGE
NTEVEQMIVTAMRRTVKRPDNTE

Bombyx mori

>Bmor_GH32_1, NP_001119721
MFAWSTPVALAAVLAVTLGQALRQQNETTKRELEEYIADKKAEINPRYRPHYHISPPV**GWMNDPNGF**SYYKEKFHLF
YQFYPYDSVWGPMHWGHVSSSNLIDWEHLPTALIPETEMCFSGGAVVHGDDLVLLYTGRVTTDTDPFYNETQYLAFS
NDGVNFRKYEGNPVLSYVPDNS**ADFRDPKIW**KFKDHWYVVIGSSSNKRGRVLLYRSGDLFNWEFLSVLGESDGDMGY
MWECPDLFELGGKTIFLWSPQGLEPKGDRYKNTYQTGYYIGELDYETFEFKTDKYFQELDYGHDFYATQTIQGDGKT
YLIGWFNMWEVPHLEKEDGWAGTTTLVRELQLIGTRITMNPLEGIQDLRTDSVHNGDLEPQQAIEFGPTAEIILQGC
LDQKIELLIQGKEGGLVTTVTWDPEVGKVIVNRSGEVRQVEWVPIGKTSWRLFLDASSLELFCGEGEVVFSSRIFSD
GDWVVKNSSPQTLSVEAYRLRRSVPA

>Bmor_GH32_2, XP_004922046 (**D in active site region 1 substituted to H, D in active site region 2 substituted to N, but there is an adjacent D**)
MDVLVVALYLRFVCAVFAVDLSHIETFVQENRYCLKQRYRPLYHISAAH**AWTNHPSAF**VVYKRQYHIFYQYHPYNGA
WGPISWGHAVSDNLVDWTFYPPALIPGELYDKHGCLSGSGIAHNGYLVLFYTGNAVSDNATLQTQNVAISTDGIVFQ
KYIYNPVIRDGAFGA**EDSRNPKVW**RFRNVWYMLLTNTREGVGRLLLYTSMDLFNWKLNGTLALSLGDT**GYAWESPDL**
IEIDGQHVLMLCLQGVPSDGFRFKNLYQTGYIVTNFNYVNGQFDDLEVSTATFTELDHGHDFYAPKTVLAVDGRRLL
IGWLGMWESHFKESKHGWASMLTIVREMKLTPQGRLLMPPVREVAELRTEILEDAWYNPGEAFYAGTRTFELIVSTA
KVFYDVAVIFEWDGDQQYTVGYSADRGHIIVDRGGVDGLRRADWAPNDKLKLRIFVDYSSIEVFCGSGDVVFSSRVY
PKKNIRVKISGESQLHVVQYKLRRSVGYDSKLRKYLKEHVLERA

Manduca sexta

>Msex_GH32_1, KAG6455701

MAAWISCLVFLCAFASVHLKYLSEDENAKKELAEYIQKKKASINPRWRLHYHVMPPV**GWMNDPNGF**SYYKGEYHLFY
QYYPYASVWGPMHWGHSASSNLIDWRELPTALVPDKEQCFSGSGIVDKDTLVLMYTGHVITDKDPFYNETQYLAFSD
DGVNFHKYEGNPVLSVSPNGT**ADFRDPKVW**RHGDYYYVVLGSKTTDKRGRVLLYRSQNLYNWEFLKVLGESHGEL**GY**
MWECPDFFELDGKHVLLMSPQGVAPQGDRYKNTFQTGYLIGNFNYDTFEFVQEVSFQELDFGHDFYATQTIDADGKR
VVVAWFAMWEVPHPEDVDGWVGAMTIMRELKISGNRILQKPLDGMLSLRNGSVHNGPVDRNESLVFEKTGELIINGD
LAKKIELEIVGTNGGNKTWIRWDPAVGKVAVDRNGDVRQVEWSPIGSHSWRLFLDASSLELFCGEGEVVFSTRIYPD
GDLKVNNLSDQSLNVEAYKLRRSVPA

>Msex_GH32_2, XP_037293842
MAAWISSLLFLCVIASVHLKYLSEDEDAKRELAEYIQETKKSINPRWRLHYHVIPPV**GWMNDPNGF**SYYKGEYHLFF
QYYPYDSVWGPMHWGHSVSPNLVDWRELPTALAPDEEMCFSGSALVDGDKLVLMYTGRLNTDTDPFYNETQYLAFSD
DGVNFYKYEGNPVLARTPDGA**YDFRDPKIW**KYGDYWYVVIGSSTHDARGRVLLYRSQNLYDWEFLTVLGESNGEL**GY**
MWECPDFFELDGKYILLMSPQGLEPQGDRYKNTHQTGYIIGSFNYETFEFVPEVDFQELDFGHDFYATQTLDADGKR
IVAGWFSMWELPHPEDVDGWAGAITIMRELKLSGNRLLQQPLDEMLSLRNGSVHNGSFNKDESLVFEKTGELIINGD
LEQKIELEIAGTNGGNNIWIRWEPEVKKVVVDRGGDVRQVEWSPIGSRSWRLFLDASSLELFCGEGEVVFSTRFYPD
GDLRVNSFSDQSLNVEAYKLGRSVPRSAGHHSISLSV
>Msex_GH32_3, XP_030037369 (**D in active region 2 substituted to N, but there is an adjacent D**)
MDVLLLATWLCVATAEFATDLAHIEAFIQQEKYLLRKKYRPVYHISAPV**GWLNDPSSF**VYFKRQYHVFYQYHPYNGA
WGHTRWGHAISDNLVDWAHYPPALVPKEYYEKHGCLSGSALVHNNYLTVFYTGHVISNNKTYQTQNVAISSDSIVFQ
KYLYNPIIRKGPYGV**SDFRNPMV**WNYRNWWYMMVGTAKDGLGHLLLYSSADLFSWTLNGTLAKSFGDM**GHMWENPDL**
FEIDGLTVLILSVQGIQAEGYRFRNLYQTGYVVGKFNYVTARFEDFEVSTATFKELDYGHDFYAAKTVQAADGRRLL
IAWLGMWESDFQESRDGWASLLTLVREVKLTSQGRILMMPVKETANLRTEILEDAWYSPGEAFSAGTKSFELIVNST
AVVYDAIITLEWNGRRQYTIGYIADQGHITVDRGGKDGVRRADWLPNGHLHLRIFVDYSSIEIFCGMGEVVFSSRIY
LKRPILVKIGGDTQLHITQYKLRRGVGYDNKLRKHLKEHIIAKKG

>Msex_GH32_4, XP_030033238
MRTKMYIKTATFLLCVFLGSVSSCCVNGRYYPRYHLSPPH**GWMNDPNGF**CYFKGEYHMFYQYNPMSSLDAGIAHWGH
AKSKDLCHWKHLDLAIYPDQWYDQTGAFSGSALVENDVMYIYYTGNVNLTDEMPFEGQFQALGVSTDGVYVEKYKDN
PIMYTPNHQ**PHIRDPKVW**EHDGSYYMVLGNAYDDYTNGQIVMYESSDKINWQEVTILYKSNGSF**GYMWECPDL**FEID
GKFVLLFSPQGVKSVGDMYQNLYQAGYIVGEFNYDTHSFTVLTEFRELDHGHDFYATQTMKDPSGRRIVVAWASTWE
YAYPERADGWAGMLTLPRTLSLTKDLKLIQTPIREIDQVFRRRLYSGKASAGKTVALPDKAGKVELKWDTPRNIKVV
IESQNECQNVVISYDHEDGTITLDRGGDDAIRRTHWDPRGHLKWTIFIDASSIELSCGDGEVWFTSRFFPEGVVSVR
LGEDTCVDKFTVHSIRRTTPDPEAHCRCESEE

Trichoplusia ni

>Tni_GH32_1, XP_026725338 (**D in active site region 2 substituted to N, but there is an adjacent D**)
MEVFILAVWLCVATAELAQDLAHIEDFIQQKRYLLKKKYRPMYHISAPV**GWLNDPNGF**IYFKRQFHIFYQYHPYNGA
WGPIHWGHVISDNLVDWAFYPPALVPKDHYDKHGCLSGSAVIHNSYLTLFYTGHVMTNNKTHQTQNVAISADGIIFQ
KYLYNPVVREGPYGN**QDFRNPKVW**RFRNSWYMLIGTSNLKEAYLLLYTSPDLFNWKLNGTIAKSYGDM**GHMWESPDF**
FEMDGFNVLILSVQGIQGDGHRFRNLYQTGYVVGNFNYLNGQFEDLEISTATFNELDFGHDFYAAKTMQSHDGRRLL
VAWLGMWESEFEESNDGWASMLTLVRELKLTFQGRILMVPVREIVELRAEVLEDAWYSPGEAFYAGSRAFEMLVNSS
TVMYDAAIVLEWSGERQYTIAYSSDRGFVSVDRGGADGIRRADWNPVNHIHWRIFVDYSSIEVFCGMGEVVFSSRIY
PRKSMRIRIAGDTQLHVTQYRLRRSVGYDNKLRKQLKNHVVNRYH

>Tni_GH32_2, XP_026725413
MISIKTFVVFLSVVGTVYLKSVKQSDAIEEVERYIAEKKAEINPRYRLQYHVAPPV**GWMNDPNGF**SYFRGEYHLFYQ
FYPYDSQWGPMHWGHVSSPNLVDWKQLPTALVPEEEMCFSGSAVVKDDTMILMYTGRLTTDVEPFFNETQYLAFSDD
GVVFHKYEGNPVLSFTPNGS**PDFRDPKVW**KHEGYWYVVIGSKTLDERGRVLLYRSPDMVNWEFLSIIGESTGGM**GYM**
WECPDFFEIKGKHILLMSPQGIEPQGDRYKNTHQTGYIIGSFNYETYEFVPESDFQEIDFGHDFYATQTTEVDGKRI
LIAWFGMWDVPYPEDVDGWAGAMTIFRELDLVANRVTMKPVEAMTDLRTETLFEGEFSEDGIIEFGKTGELIVKGDL

SKNIELEIKGSNGGGQVIVRWDTAVRKVVVDREGDVRQVEWLPLKSESWRIFLDSSSLELFCGEGEVVFSTRVYPDG
DWYVTNRSPQALDTVAYKLRRSVPE
>Tni_GH32_3, XP_026725412 (**D in active site region 1 substituted to A**)
MNLFKIYIILVSSFMTVQLESLRDVLAKAELEIYIEEKKLEINPRHKLHYHITPPV**GWMNAPNGF**VFFKGEYHVFYQ
FYPYDTQWGPMHWGHVTSRNLVDWRHQPTALLPGREQCFSGSVIDNDGILAVMYTAHQISVDPPYYNESQYMAFSID
GIDFHKYKNNPVIVSSPFSS**PDFRDPKIW**KHGNVWYTVIGSRTDDHRGAALLYRSINIINWEFVSTLAESNGEL**GFM
WESPDF**FELNGKHVLLISPQGIVAQGDRYKNTYQTGYIIGNFNYENFEFTAEATFQELDFGHDFYGTQTTEKDGKRY
LIAWFGMWESAHSEDVDGWAGAMTLFRELDLVGTRIIMKPVDAITNLRMQTISEGELARNSSIQFEQTAELIINVDL
SERIELELKGNGVGGDWAIVRWEVDEAKMVLDRAGDVRQVGWEPLDSMTWRLFLDTCSLELFCGEGEVVFSTRIYPN
GQWRVKNLGPQPIHVVAYKLRRSFPE
>Tni_GH32_4, XP_026725409
MQSIMGSLTVCVFLLSLCAGVLSCELKSSEKVISQRASLETYIKEKKPSIGNQYRLLYHVAPPV**GWMNDPNGF**SYFK
GQFHLFYQFYPYNSSLSPAASIHWGHSTSANLIDWTEQPTALIPDEEQIFSGSAIEISGTLVLMYTAHINPAIAGQN
ATELQYLAFSNDGIVFNKYKNNPVISRSPNGS**PDFRDPKVW**KYGDYYYVVLGSRTDQGLGRVLLYRSQDMASWTFLS
VVGQPSSRM**GYMWECPDF**FELGGKYILLMSPQGIIAEGDRYKNLYQTGYIIGDFNYQNHEFVERVPFQEIDYGHDFY
ATQTAERDGKRYLIAWFGMWNTTFPESAEGWAGAMTIFRELTLRGDRILMRPVSTINSLRESSAFNGDLIQNAAIQL
NKTGELNVSGNLSQAIDLDIKGSEGGGVRIRWNATSKIVSVDRDGDVRQGPWTPVGSTSWRIFLDASSMELFCGEGE
VVFSSRAYPTGHWVVTNLSAQSLSVSAFRLRKSV

Spodoptera exigua

>Sexi_GH32_1, KAF9412149
MTLQSAMAVLKLYVALLGVLATTLACDLTPSAAETELKLLAETYINVTKPTIDSRYRQHYHVAPPV**GWMNDPNGF**SY
FKENYHLFYQFYPYNTSTLNIHWGHVISKDLVKWTQLPVALAPETEQIFSGSAIEKDGTLVLIYTAHKGKDPDTNET
QYLAFSNDGVIFTKYKKNPVISFSPNTA**KDFRDPKIW**KHGDYWYVVLGSKTVDTKGEVLVYRSADLGYWEFLNVVAS
SNGDL**GYMWECPDF**FELSGNYVLLMSPQGMNASGDRYQNQFQTGYIIGSFDYQTGKFVEKYAFQEIDYGHDFYATQT
MERNNKRYLIAWMGSWNGEFPERADGWAGSMTIVRELTLNGDRILMKPIDAITELRESAVYNASLAQNQHITGLEKT
GELLISFDLAQDISLAIRGVNDVSNNTNVIIGLRWDASAKKVIVDRQGVIRQGEWVPVSSKSLRVFLDASSIEVFCG
EGEVVFSSRAYPNGNWQITNLGTQSVDVVAYTLS
>Sexi_GH32_2, KAF9412150 (**D in active site region 1 substituted to G**)
MHISTYIILMCLGSVQLDQLKEILEKAELEIYIEETIPSVNPRYKLHYHITPPV**GWMNGPNGF**AFYKGEYHLFYQFY
PYDTQWGPMHWGHVSSPNLVDWKTLPTALRPGNEQCFSGSAIDNDGILVLLYTAHQSIEDSPFYNESQFMAFSIDGL
DFHKYKGNPVNIFPPTGT**QDFRDPKIW**KNGNYWYVVLGSKTADHRGAVLLYRSKNLLSWEFKSVLAESNGGL**GYMWE
SPDL**FEINGKFILLISPQGMVSRGDRFKNTYQTGYIVGSFSYETCQFKPEINFQELDYGHDFYTTQTTETNGKRYLV
AWFGMWESLHPEDIDGWVGAMTLIRELDLVGSRILMKPVEAITKLRQETILEGEFHHNSTIEFEKAGEVIVNIDLSE
NIELEFVGNNGGDRTSLRWSVDDGKVVLDRAGEVRQGIWEPLDTITWRIFVDASSIEVFCGEGELVFSSRVYPNGNW
RVINRGTQALHVVAYTLKRFQNE
>Sexi_GH32_3, KAH9628466
MTILKYVVFLSFFAVVHLKTIKQNNAINEIEAYIEEKRAEINPRYRLHYHVAPPV**GWMNDPNGF**SFYKGEFHLFYQF
YPYDSQWGPMHWGHAVSPNLVDWKTLPTALVPEEEMCFSGSAVVKDDEMVLMYTGRRTTEEEPFYNETQYLAFSNDG
VEFHKYEGNPVLPYTPNGS**PDFRDPKVW**QHGDHWYVVIGSKTDDERGRVLLYRSVDLINWEFLSVIGESSGDM**GYMW
ECPDF**FQLKGKHILLMSPQGLVPQGDRYMNVHQTGYIIGKFSYETFEFVPEVSFQEIDFGHDFYAATTTEKDGKRYI
VAWFNMWDVPHPEDVDGWAGAMTIVRELELVGNRIVMKPVETITSLREETVWDGNLMENEFVPFDKTGELLITGDLS
QNIELLIKGSNGGGQVVLRWDPQVRKVVVDREGDIRQVEWLPLGSEQWRIFLDASSLELFCGEGEVVFSTRVYPDGG
WQVTNLSPQPLDVIAYALRRSVPE
>Sexi_GH32_4, KAF9407421(**D in active region 2 substituted to N, but there is
an adjacent D**)
MEVFVLAVWLCVVSAELAQDLAHIEDFIQQKRYLLKKKYRPMYHISAPV**GWLNDPNGF**IFFKRQYHIFYQYHPYNGA
WGPIHWGHVISDNLVDWAFYPPALVPKDHYDKHGCLSGSAVIHNSYLTLFYTGHVLSNNKTYQTQNVAISADGIIFQ
KYLYNPIIRDGPFGN**ADFRNPKVW**RFRNSWYMLIGTSRLQEAYLLLYTSSDLFNWKLNGTIAKSYGDM**GYMWESPDF**
FEIDGFAVLILSVQGIQGDGHRFRNLYQTGYVIGTFNYQSGQFEDLEISTATFNELDYGHDFYAAKTMQSVDGRRLL
VAWLGMWESEFEESNDGWASMLTLVRELKLTFQGRLLMTPVREIVELRAEVLEDAWYSPGEAFYAGSRAFEMLVNSS

TVMYDAAIILEWSGERQYTIAYSSERGFVSVDRGGVDGVRRADWLPVTHIHWRIFVDYSSIEVFCGMGEVVFSSRIY
PRKSMRIRIAGDTQLHVTQYRLRRSVGYDNKLRKQLKNHVINRYH

Helicoverpa armigera
>Harm_GH32_1, PZC86172 (**D in active site region 1 substituted to G**)
MNLLSTNVIMISLLTSVHLDTLKEMLDKSELEMYITEAVATVNHRYKLHYHITPPV**GWMNGPNGF**LFYKEEYHLFYQ
YYPYDTQWGPMHWGHVASTNLIDWRPLPTALRPADEQCFSGSAIDNQGIMVLMYTAHQSTKNPPFYNESQYLAFSVD
GVDFHKYKGNPIAIKPPPGT**QDFRDPKV**WRHGMHWYVVLGSKTRDQRGAAILYRSTDMIHWQFQSVLAESSGAL**GNM**
WEWPDFFEINGKFILMISPRGIIPSGDRFKNTCQTGYIVGTFSYATCQFKQEVGFQELDYGHDFYATHTAERNGKRY
LIAWFGMWESVHPEDADGWVGAMTLVRELDLVGSRILMKPVEAITSLREETILEGEFHHNASIEFEKTGEIIVNLDL
TENIELEITGTLGGDRLSVRWSLDEGKMVVDRMGEIRQVGWEPLDTVIWRIFLDTSSFELFCGEGEVVFSSRVYPLG
EWRVSNRGPQSLHVVAYTLKKKRKAN
>Harm_GH32_2, XP_021193708 (1)
MEGLKVYAVLLSVVAAALACDLRESAEDIARKTAVEKYINDTRPTVLANQKYRLQYHVAPPV**GWMNDPNGF**SYFKDN
YHLFYQFYPFDTSTGRIHWGHVISKDLVTWEQLPVALVPEEEQCFSGSAIEKEGTLVLMYTAHKNISGVVNESQYLA
FSNDGVVFNKYVKNPVISFTPDNS**PDFRDPKVW**KYGEYYYVVTGSRTSDAKGRVILYRSKDLGFWEYVSEIGKSNGD
L**GFMWECPDF**FELNGSFVLLMSPQGIENKTTDRYQNQFQTGYIIGSFDHSSGKFQERVPFQEIDYGHDFYATQTMEH
EGHRYLIAWMGTWNADYPERAHGWAGAMTIIRELTLKGNRILMNPIDAITNLRGSKAHNGTLEPEGIIALNKTGELI
LNVNLTQDLTLNIKGEADESLIVLKWDTATQKVVLDRKGVVRQGEWIPTSSSTAWRIFLDASSIEVFCGEGEMVFSS
RVYPEGMWRVINSGTEAVNVMAYELNRSVPM
>Harm_GH32_3, XP_021193708 (2)
MKYQSIMARLNLLVVFLCLIAIVLSKTVRHNDAISELEAYIEEKKAEINPRYRLQYHVAPPV**GWMNDPNGF**SYYKGE
YHLFYQFYPYDSQWGPMHWGHVASPNLVDWRQLPTALIPEDEMCFSGSAIVKDDELVLMYTGRLVTAEEPYFNETQF
LAFSDDGVKFHKYEGNPVLGSAPNGS**PDFRDPKVW**KHGDHWYVVIGSKTSDEKGRVLLYRSLDMYNWEFLSVIGEST
GEM**GYMWECPDF**FELKGKHILLFSPQGMVPQGDRYKNTHQTGYIIGSFNYETFEFIPEVSFQEIDFGHDFYATQTTE
HDGKRYLIAWFGMWEVPYPEDVDGWAGAMTIFRELKLVGNRLLMKPVETMTSLREDTVFEGNLAENVAIQFDKTGEL
LVNGDLSQKIELEIKGSDGGGQVSLQWDPEVRKVVVDREGDIRQVEWLPLGSEQWRIFLDASSLELFCGEGEVVFSS
RVYPDGGWKVTNLSPQTLDVVAYKLRRSVPE
>Harm_GH32_4 (XP_021182689)
MLTIATKFILIALFFNRCSGYDINPQFVPRYHVYPPY**GWMNDPNGF**CKFENEYHLFYQYNPLSSQEPGIAHWAHVKS
PDLIHWENLPIAMYPDQPYDKDGVFSGSAIIENGTMYLLYTGNVNFPGGNPDHQQVQALASSTDGVNVTKYEGNPVI
KGEKFQ**PNIRDPKVW**KHGNIFYMVLGNSFDNNTRGRALLYSSPDLIRWKERSVLDSSDGSL**GYMWECPDF**FELDGKY
VLLFSPQGIEPQGDKYRNLYQTGYIVGMFDYNSNMFTPITEFKELDHGHDFYATQTILDESGRRIVIAWLDMWERNY
PERDFGSTGQMTIPRVLSLTTDLRLIQKPVKELANARGSQLYSGRARGGHTIKLDEPAADIHVRASRFRDFNLALVT
DNTTVLLSYSYLRGTVTLDRGGDDGIRRTQWRPSWNKQLTWQILVDSSSVELFCGEGEVTFSSRFFPYSNVSIKIGE
NTSAEELTVYRMRRTIKLPDGSN
>Harm_GH32_5, XP_021182688
MSIATKFILTVLFLNRCWAGDVNPRYVPYYHVYPPSG**WMNDPNGF**CIFQGEYHLFYQYNPYTSQEPGVAHWGHVRSP
NLIQWEHLPTAMTPDQAYDINGVFSGSAIVENGTMYLLYTGNVNNPTNKQVQALAASQDGISVVKYPGNPVIEGADL
Q**PNIRDPKVW**KHGDFFYMVLGNSFDDNTRGRVLLYSSPDLISWTMESVLDESDGSL**GNVWECPDF**FELDGKFVLLFS
PQGMQPIGDKYKNLFQTGYLVGNFDYETKLFTPITEFRELDHGHDFYATQTILDHSGRRIVVAWFDMWESVHPERND
GWSGQITIPRELELTEGLRLLQKPVKEIAAARGAKLRTGKARARYTLSLDSAAADVKVTAPRLQDFELLLESKDFTL
SIKYDYKKGTVTLDRGGDDGVRRTKWRPEGNLVWQVLIDSSSVELFCGEGEVTFSSRFFPNGGLKIRIGDGSHADDL
TVYEMIRTIQAPGDN
>Harm_GH32_6, XP_021182690
MLSIATKFILTALFLNSCWAIDVNPRYVPHYHVYPPS**GWMNDPNGF**CIFEDEYHLFYQYNPYSSQEPGVAHWGHVKS
PDLINWEHLPTAMTPDQPYDINGVFSGSAIVENGTMYLLYTGNVNNPFNKQVQALAESQDGISVTKYAGNPVIEGAD
FQ**PNIRDPKVW**KHGDLFYMVLGNSFDDNTRGRVLLYSSPDLISWTLESVLDESDGSV**GHVWECPDF**FELDGKYVLLF
SPQGMQAIGDKYKNFFQTGYVVGNFDYETKLFTPITEFRELDHGHDFYATQTILDGSGRRVFVAWFSMWESVHPERN
DGWSGQIIIPRELELTEGLRLLQKPVKELDAARGPKLRSGKAKAGYTLGLEAAAADIRVTAPRLQDFELLLESDDST
LSIKYDYRRGTVTLDRGGDDGVRRTKWRPEGNLIWQVLVDSSSVELFCGEGEVTFSSRFFPNGALKIRLGDGSNADD
LTVYKMLRTIAAPGDN

>Harm_GH32_7, XP_021197299
MKILKVYSVIFSVIVTALACELRETNEEIQSKRLVEEYINKTRPVVLDNPRFRLHYHIAPPV**GWMNDPAAF**SYYKEN
YHLFYQYYPFNTTTGNTHWGHVISKDLVTWTQLPIALIPEEELCFSGSAIEVEGTLVLMYTAHKKNEYSFNETQYLA
FSNDGVVFNKYVKNPVISFANNNS**LDFRDPKVW**KYGALFYVVIGSRSSDDKGRVLLYKSKDMEYWEYISEIAKNNGD
L**GFMWECPDF**FELNGRFVLLISPQGLTNKTTDRYQNQYQVGYIIGTFDYATGDFKEEVAFQELDYGHDFYTTQTLER
KSIRYLIAWMGSWDAMFPEREDGWAGAMTITRQLTLNGDRILMKPVQAIENLRGPPIFDGYLEPDNIISLNQTGELI
ITVNVTEDLILNIRGENDISLIILKFDMATKKVVLDRNGEIRQGEWKPILSTSWRVFLDASSIEVFCGEGEMTFTSR
VYPNGMWRVVNSSVKGVNLTAYSLNRSVPL
>Harm_GH32_8, XP_021197300
MKTLQVYSVIFSIIVTALACELRETNEEIQSKRHVEEYINKTRPFVVDNPRYRLHYHIAPPV**GWINDPAGF**TYYKEN
YHVFYEYNPFNTSYGKTHWGHVISKDLVTWTQLPMALIPEEELCFSGSAIEIEGTLVLMYTAHKKNESSFNETQYLA
FSNDGVVFNKYVKNPVISFANNNS**LDFRDPKVW**RHGDRYYVVIGSRSSDDKGRVLLYKSKDMEYWEYVSEIAKNNGD
L**GYMWECPDF**FELNGRFVLLISPQGLTNKTTDRYQNQYQVGYIIGTFDYATGNFKEEIAFQELDYGHDFYTTQTLEH
EGIRHLIGWMGTWYAIFPEREDGWAGAMTITRQLTLNGDRILMKPVQAIENLRGPPIFDGYLEPDNIISLNQTGELI
ITVNLTEDLILNIRGENDISLIILKWDMATKKVVLDRNGEIRQGEWKPVLSTSWRVFLDASSIEVFCGEGEMTFTSR
VYPNGMWRVVNMSVQAVNVTAYALYRSVPL
>Harm_GH32_9, XP_021193953 (**D in active region 2 substituted to N, but there is an adjacent D**)
MEVFILAVWLCVATAELAQDLAHIEDFIQQKRYLLKKKYRPMYHISAPV**GWLNDPNGF**IFFKRQYHIFYQYHPYNGA
WGPIHWGHVISDNLVDWAFYPPALVPKDHYDKHGCLSGSAVIHNSYLTLFYSGHVLSNNKTYQTQNVAISADGIIFQ
KYLYNPIIREGPFGN**TDFRNPKVW**RFRNSWYMLIGSSRVQEAYLLLYTSPDLFNWKLNGTIAKSYGDM**GYMWESPDF**
FEIDGFNVLILSVQGIQGDGHRFRNLYQTGYVIGSFNYLNGQFEDLEISTATFNELDYGHDFYAAKTMQSLDGRRLL
VAWLGMWESEFEESNDGWASMLTLVRELRLTYQGKLLMAPVREIIELRAEVLEDAWYSPGEAFYAGSRAFEMLVNSS
TVMYDAAIILEWSGERQYTISFSAERGFVSVDRGGVDGVRRADWMPLTHIHWRIFVDYSSIEVFCGLGEVVFSSRIY
PRKSMRVRIAGDTQLHVTQYRLRRSVGYDNKLRKQLKNHVINRYH

Pararge aegeria

>Paeg_GH32_1, XP_039754427
MSVIHAGLILLSVVTIAYASSVSNYENAKAQLEEYIQNKKKEINPRFRPLYHVAPPV**GWMNDPNGF**SYHKGEFHLFY
QFYPYDSVWGPMHWGHVTSPDLVNWKQQPTALLPEKEQCFSGSAVTQGDQMTLMYTGHAAIDVEPYFNESQYLAFSD
DGVNFYKYEGNPVLPISPNGS**PDFRDPKVW**KRGDDYYVIIGSKTLNDRGRVLLYKSRDMKSWEFLSVIGESNGEL**GY
MWECPDF**FELDGKFVLLISPQGMSEKGDRYKNTNQNGYIIGSFNYDTNEFVQEVGFQEIDFGHDFYAATTTEANGKR
YLSAWFSMWGVPHNEDVDGWAGMLTIPRELRIVGNRITMNPVEDIVSIRDGVAVDGDVWSNQVLEFNKAVELIINSD
LNQKVDLLLEGREGGGSAWLRWDPEVRKVVVDRGWGDVRQVEWSPIGSKTWRVFLDSSSLELFCGEGEVVLSSRVFP
LGGWKLTNQSPQTINVKAYNLKRSAPE

>Paeg_GH32_2, XP_039750038
MALIRNSLLFICFVVQLNATSILSNYENAKIELEEYIENKKKEVNPRFRPLYHVAPPV**GWMNDPNGF**SFHNGEFHLF
YQFYPYDSVWGPMHWGHVSSPDLVHWKQLPTALLPEKEQCFSGSAISQENGLTLMYTGRVAIDVEPYFNETQYLAFS
DDGINFYKYDGNPVLSRAPNGS**PDFRDPKIW**MYEDYYYVVIGSQTTDKRGRVLLYKSSDMKSWNFLSVLGESNGDM**G
YMWECPDF**FELDGKFVLLMSPQGLEPQGDRYKNTHQTGYIIGNFNYDTNEFEQEVAFQEIDFGHDFYAATTTEVNGK
RYLIGWFSMWDEPHPEDVDGWAGMATLVRELRIVGSRITMQPVEEIVTLRNQIVLDSDIEPNQILVFGKAVELIINS
DINQKVDLLLEGRKGGSKAWLRWDPETRKVTIDRGSGDIRQVEWYPNGSIHWQIFLDTSSLELFCGEGEVVFSSRVY
ALGGWKLTNESNQTVHIKANSLKRSTPE
>Paeg_GH32_3, XP_039746214
MFLFVYSINAEITNNRYYPLYHFAPQQ**GWMNDPNGF**SVFKNVYHLFYQYNPVSSLEPGTAHWGHATSKDLVHWKHLP
IAMYPNNTYDKNGVFSGSALIEDDKMYLYYTGHINHPGETPDHEEHQALATSTDGVNVVKYENNPILYGTGRQ**PDIR
DPKVW**KHGDTYYMVLGNKFTKNNTSLGRVLLYSSKDKYNWKEVAVIGESDGFL**GYMWECPDF**FQLNGHFVFLWSPQG
MEPQGDKYRNLYQTGYIVGDFDYKTLSFKPITEFRELDHGHDFYATQTILDNKGRRIVVAWFDMWKRNYPEQLDGFT
GQLTLPRILTLTKDLRLIQRPVPEIKVARTKTLCTGQAKGGTKVTLKDKAGEINIRARVTNDMELFIDGRNTSMRIY

YSAFCGEISLDRGGEDGLRRTEWRPKKELMLNIYVDASSIEVFCGTGEVTMSSRYFTDGPVSVRLGDTSSVWQFKVT
NMRSTVPLNGSYERQ

>Paeg_GH32_4, XP_039746097
MIVKMGALCKNLIIFMFVNSINAEITNDRYYPRYHLAPPQ**GWMNDPNGF**SVFQQEYHVFYQYNPDSALEAGTAHWGH
AKSRDLLNWQHLPIAMYPNETYDSSGVFSGSALIEDDTMYLYYTAHTNHPGETPNHEEHQALAISTDGVNVVKYQNN
PLIEGADRQ**PDFRDPKVW**KHGDTYYMVLGNQFTKNDTRLGRVLLYSSKDKYNWSEVAIIGESDGSL**GYMWECPDF**FQ
LDGHFILLMSPQGIPAEGDKYKNLHQTGYIVGDFDYRTLTFKPTTSFQELDHGHDVYATQTILDNKGRRLVLAWFDM
WETNYPEQLDGFTGQITIPRILTLSKDFRLVQRPVPEIRAARTNIVYTGQASGGTVATLENKAGEINIRASALYDLE
FFIEGINASVQIYYNASSGQISLDRGGDDGLRRTEWQPANVLILNIYVDASSIEVFCGAGEVTLSSRYFPDGPVSVR
LGDSSEASQFRVINMRSTVPIN

Heliconius melpomene

>Hmel_GH32_1, CBH09256 (**D in active site region 1 substituted to A, D in active site region 2 substituted with N, but there is an adjacent E**)
MDHIRITVAVAFLFILGVVAQHNNLLRKRYRPIYHITAPE**GWISNAPTGF**TFYKRQYHIFYQYHSYNGAWGHMNWGH
AVSKNLVDWMHYPSALLPNDYYDRHGCFAGSAIVNRNFLLLFYTGRILSEKESYETQNVAVSGDGVFFQKYLYNPVL
RQSPNGI**GEFRNPKVW**RFGRRWYMIVGNTSTKRHGQLLLYTSEDLFSWNFNNTLVTSYGDM**GYIWENPDL**FELDGMH
VLIISVQGMELDGWRFRNLCQTGYVIGHFNHYKGRFDDIEVSSATFNQLDYGHDFYGAKSMIATDGRRILIAWLGMW
ESELKESTFGWASMLTIVRELRLNENGVLLMSPIREMEELRGELLEDAWYSPGETFPAESKSFELIVNSTSIYFDVG
LVFEWKLGRFTIGYSAEHGYVSIDRGGIDGIRRAYWSPVSHVYMRIFVDFSSIEVFCGNGEVVFSSRIYPKTIRVRV
VGNSQLYISQYKIRRSIGFDSELVQRLNLAINPYQS

Danaus plexippus

>Dple_GH32_1, OWR41324 (1)
MSLIQKILCLCLIGLSQTANLTYDELKRELESYIENKQKEINQRYRPLYHVAPPV**GWMNDPNGF**AYHNGLFHLFYQF
YPYDSKWGPMHWGHVTSTDLVHWKQKPTALIPEEEQCFSGSGISDDNKLVLMYTGHVLTEEEPFYKQSQYLALSEDG
VNFEKSKSNPIIESPPNNS**PDFRDPKIW**KHGNEFYAVIGSKDNGNGTVLLYKSSNLTTWEYLSVLGQSTGEL**GYMWE
CPDF**FELDGKFILLMSPQGVKANGDRYKNVYQTGYMVGTFDYNTSRFETDGVFQELDYGHDFYAATTTEAKGSRYLS
AWFSMWEVTHPEDVDGWIGTTTLVRELRFVNNRLIMKPVAEIVDLRESLALQGDFKANQVQEFGKAVEILIEGNLKQ
NIDLLLDGPNGGGQVQLKWDAKNGTVSVIREEEVRRVVWAPIDSQIWRIFLDTSSLELFCGEGEVVFSSRVYPLGGW
RLTNNSPQTLKITAYTLMRSVPDNSSIRIKSFSGTISVITLLALRISNIL
>Dple_GH32_2, OWR41324 (2)
MEFYRFIAFTCILALAYSKSVDNNYENEKEDLENYIQNKKNEINHRFRPLYHVAPPV**GWMNDPNGF**SYHNGEFHLFY
QFYPYKSEWGPMHWGHVISSDLVHWKQMPTALLPGTEQCFSGSAISQGDVLTLIYTGRRSIDEQPYFNESQYLAFSD
DAVNFYKYEGNPVIPNAPNNA**PDFRDPKVW**KYGDEYYVIIGSKTSDERGRVLLYKSKDMFDWEFLTVLGESNGSL**GY
MWECPDF**FELDGKFILLMSPQGVSPQGDRYKNSHQTGYIVGSFDYDTFQFIPEVEFQEIDYGHDFYAATTTQANGKR
YLLAWFSMWDVPYPEDVDGWAGMMTITRELNLVNNRILMKPVSDMLNLRNEVALKDEVKPGQVHQFGKAVEIIIESD
LYNKIDLLLDGQEGGGKVWIRWDPDIGKVVVDRGSGDIRQVEWKPIGSTTWRIFLDSCSLELFCGEGEVVLSSRVYP
LGGWRLKNQSPQTIRVEAYNLQRSVPE
>Dple_GH32_3 OWR41190 (**active site region 1 not detected**, **D in active region 2 substituted to N, but there is an adjacent D**)
MSWGHVVSKNLIDWTHYPSAVMPKDVYDRNGCLSGSALVINNFLTLFYTGHLASSNEVFQTQNIATSGDGIIFKKYI
YNPIIRQSPNGV**GDFRNPKVW**RFRNLWYMVVGTSSRERFGELLLYSSTDIFNWKLNGTFVKSYGDM**GHMWENPDI**FE
LDGQHVLIISVQGIEADGFRFRNLYQTGYVVGTFNYMKGKFEDLEVSIATFYQLDFGHDFYGAKTLLASDGRRILIA
WLGMWESDFIESTSGWASMLTIIREVRLNKRGRILMTPISEMEELRVEIMENAWYYPEESFQAGAKSFELLVNSSSM
LYDTGLVFEWNFGTFTIGYSAEHEYISIDRGGPDGVRRAYWSPTNHIFLRIFVDVSSIEIFCGEGEVVFSSRFYPKS
MRIKVIGKSQLHITQHRLRRTIGYDKELVNRLKVS

Papilio polytes

>Ppol_GH32_1, XP_013141532
MLAITVFLFILFCYRATAVVDDRYYPRYHLAPPY**GWMNDPNGF**SEFKNEYHLFYQFNPNSSLVPGIAHWGHAKSKDF
FRWEHLPIAMYPDQWYDKGGVFSGSALVENDKLYLYYTGNVNKPGQYPDHQQHQVLAISTNGVDVTKEKINPIINGS
NYQ**PDFRDPKVW**KYRNTYYMVIGNSFQDEETNSTLGRALLYTSKDKIQWDFASILDESDGSL**GYMWECPDF**FELNGR
FVLLFSPQGIPPSGYKYRNLYQTGYIVGDFDYKTNMFTPKTEFIELDYGHDLYATQTILDKSKRRIVIAWMDMWDQN
YAEAEDGFTGQMTIPRELSLDKYNRLIQRPVKEIASIRGRTRFIGKGRAGAEINLRDKSGEIHIKSSANKDLEIWIQ
STNESSNVLISYNYKRGIVTLDRGGKDGVRRAKWRPTDKLHLKAYIDASSIELFFGNGEVTFSSRFFPVGPIKVRIG
ESNDVESISVIDLQRSIDATYKIESED
>Ppol_GH32_2, XP_013141674
MAYSLTTLASVSSSAESRDKAFMTSKSQITQYGTQFLRIGCFNNSWYICYFNMKSLTQTFRSWTFCCANKNLTTKDR
QRATAVVDDRYYPRYHLAPPY**GWMNDPNGF**SEFKNEYHLFYQFNPSSSLVPGIAHWGHAKSKDFFRWEHLPIAMYPD
QWYDKGGVFSGSALVENDKLYLYYTGNVNKPGQYPDHQQHQVLAISTNGVDVKKEKINPIINGSNYQ**PDFRDPKVW**K
YRNTYYMVVGNSFQDEETDAILGRALLYTSKDKVKWNFASVLAESDGSL**GYMWECPDF**FELNGRFVLLFSPQGIPPS
GYKYRNLYQTGYIVGDFDYQTNMFTPKTEFVELDYGHDLYATQTILDKARRRIVIAWMDMWDQNYAEAEDGFTGQMT
IPRELSLDNNNRLIQRPVKEIASIRGRTRFSGKGRAGAEIRLRDKSGEIHIKSSGTKDLDIWIQSTNESSNVLISYN
YKRGIVTLDRGGKDGVRRAKWRPSEQLDLKAYIDASSIELFFGNGEVTFSSRFFPAGPIKVRIGESNDVESISVTDL
QRSIDASYKIESED
>Ppol_GH32_3, XP_013143210
MTLSRNLILICLAVSVAAKCIREEDPKAELERYIENKIPDINQRWRPHYHVAPPV**GWMNDPNGF**SYYNGEYHLFYQF
YPYDSVWGPMHWGHVKSPDLVNWHQLPTALLPDEEQCFSGSAVQDGDTMVLMYTAHIISDREPFYNETQYLAYSDDG
VNFNKYNGNPVLPSAPNGS**PDFRDPKVW**RHGDHWYVILGSKTTDLRGRVLLYRSTDLKEWEFLKVLAESQGDM**GYMW**
ECPDFFELDGKFILLMSPQGLSPQGDRYKNTFQNGYIIGTFDYDTFEFVQEVPFQEIDFGHDFYAAQTTEVDGKRYL
MSWFSMWEVPHPEDVDGWAGMMTIARELKLVGNKIIMKPVDGMADIRQSVPILNVLEYNEEVDFEQAVEINIEADFD
EIVEIKLEGRNGGGYVVLSWDPLVGKVSVDRGAEDVRQVEWEPNNETSWRIFLDSSSIELFCGIGDVVFSSRVYPLG
GWKMINLSSQDIEIEAYNLKKSFPV

Caddisflies (Trichoptera, Insecta, Arthropoda)

>Dann_GH32_1, ON703150
MNIYIKTIACSFATLMISCASGDIVIDDFESGTFDKWTVTGDAFGASPTMGAYPGQQEVTGFGGKYLANSFYNGDDS
FGTLSSSEFTIERDFINFLIGGGKHVNTYIELLIDGKSIYQARPVDESESLQWMAWDVKAYKNQKATIRIVDNQQGG
WGHTLVDDIVMSDESKSSFLTNYELSFDIDKKYLLIPIEDKSPEYHLHLDKDGKTISPQLDIRIAQSQIDYWMPINV
EEYKGKKISLIFDLMRKENIGYSQIKQSDDFHFDYNEKYRPDYHFSPPY**GWTNDPNGM**VYHNGEYHLYYQHNPYGSM
WGNMSWGHTVSKDLKKWEHLPVAIVGDSLGAIFSGSTVIDKDNTAGFGKDAMIAIYPSAGQAQTQSIAYSTDNGRTF
TKYEKNPVLTDPNY**VDFRDPKVF**WHEARKQWIMSLTTSQVITFYSSPNLKEWNKLSEFGQGLGAH**SGVWECPDL**FPM
SYNGQSKWVLFVSINPERPNGGSATQYFIGNFDGKTFKADSLPYPLWLDYGRDNYAGVTWNSAPDNRRIFIGWMSNW
DYTNQVPTLNFRNAMTIARELKLVHNGKHLVVANPPVKEIYDLRADTKDLPDILVNKTHTIDKLLDNNDGSYEIEMT
FVPNQSTKFDFKLLNRKGEELSFTFDIKNESLIVDRSKSGIIDFSTNFGSTAIKAPIHKSTIYTIRLLMDKSSSELF
VNNGEITLTNTLFPTEPYNTLMINSEDGILNVKDLKTYDLK

Micropterna sequax

>Mseq_GH32_1, ON703152
MNIYIKTIACSFATLMISCASGDIVIDDFESGTFDKWTVTGDAFGASPTMGAYPGQQEVTGFGGKYLANSFYNGDDS
FGTLSSSEFTIERDFINFLIGGGKHVNTYIELLIDGKSIYQARPVDESESLQWMAWDVKAYKNQKATIRIVDNQQGG
WGHTLVDDIVMSNETKSSFLTNYELSFDIDKKYLLIPIEDKGPEYHLHLDKDGKTISPQLDIRIAQSQIDYWMPINV
EEYKGKKISLIFDLMRKENIGYSQIKQSDDFHFDYNEKYRPDYHFSPPY**GWTNDPNGM**VYHNGEYHLYYQHNPYGSM
WGNMSWGHTVSKDLKKWEHLPVAIVGDSLGAIFSGSTVIDKDNTAGFGKDAMIAIYTSAGQAQTQSIAYSTDNGRTF
TKYEKNPVLTDPNY**VDFRDPKVF**WHEASKQWIMSLATSQVITFYSSPNLKEWNKLSEFGQGLGAH**SGVWECPDL**FPM
SYNGQSKWVLFVSINPGGPNGGSATQYFIGNFDGKTFKADSLPYPLWLDYGRENYAGVTWNSAPDNRRIFIGWMSNW

DYTNQVPTLNFRNAMTIARELKLVHNGKHLVVANPPVKEIYDLRADTKDLPDILVNKTHTIDKLLDNNDGSYEIEMT
FVPNQSTKFDFKLLNRKGEELSFTFDIKNESLIVDRSKSGIIDFSTNFGSTAIKAPIHKSATYTIRLLMDKASSELF
VNNGEITLTNTLFPTEPYNTLMINSEDGILNVKDLKTYDLK

Halesus radiatus

>Hrad_GH32_1, ON703226 (1)
MNCFAFGLALPCATHAVRSAIRTSADCEAIIHNSLKPSKMNTLKHTALFLLMSISLFKLEAQQIWSFDQANPLQANN
SVQLTLHSIREPELTDGIVGKALRTDGYTTWLSTQFDTPRHINGISAWFALESFPTDTAAFFGLRNKQNKETLTLGT
NMFGKLLIGTVVNGNFIYLPVTYTVKRFEWLNLMLTIHKGRLALYVNGNEINIDQSAKVDAVVFDELIVGKDFRDKK
LGIHDLTAINGLIDDITIWDTPIDIKTRQTEIGQLSHKVPILAIPKSRFANDFSRPKYHLLPSA**NWTNETHGL**IYYR
NSYHIFNQKNASNLFLGQINWGHFSSPDLINWTEHKPALTPEPQYDAMGIWSGHVVIDHNIPTIIYTTGDAKMGIGI
AFPKDSTLIDWVKYENNPVVHGQPKAYTR**TDLRDPYVW**KEANNWYMAVGFGIKDHQSERGALLLYRSVDLRKWDFLH
TLYEGTPEIDNS**GIFWEMPVF**KKIGDQYVLLINKVPHKGVPAKAMYWVGKFIDERFIPNNAIPQNLEIVNRLLSPSI
TEDKDGLITAIAIIPDEIGSQAAYQQGWTHLYSIPRVWNLKDGSIKQSPHPALATLRGKHEAIGLSKVTESKPLLIN
KDQHQLEVKVNIKPNGSKKFGFVMAKNQNLSEYTRIYYDVDQKEFIVDQTHSSLKQGIPLRVRKGAYNLDLTQPIEL
RVFIDGSVVEVFVNNEDAFTTRIFPLKETSTMLEFFVEGGSLDVDGELWILNSANMKADF
>Hrad_GH32_2, ON703226 (2)
MKHIITIVLLAFLACGGKKEPISIEKKTATETLINIQANSKYLLIPIEDQGKEIIVDLVADGKQINQYTIRLANTKI
DYWVPLDISLYKDVQLSIANLSDSALVFKSIKQSDTFDYNYHETYRPLYHFSPPV**GWMNDPNGM**VFYNGEYHLCYQS
NPYGSKWQNMSWGHAISTNMIDWKDQPTAIYPDKLGTIFSGSSVVDWNNTAGLQDGNEKTLLAFYTNFLPNEQYQSL
AYSNDKGRTWIKYSKNPILKHPTA**KDFRDPKVF**WHTPTSKWIMILAVGQVMEIYSSNNALDWVKESEFGLGMGAH**DG**
VWECPDLFELRLDGTANRKWVLVCNINPGGPQGGSATQYFVGSFDGKQFVNENPADQTLWMDWGKDHYAAVTWADMA
NMDHESIDNPRRVSIAWMSNWEYSNNVPTINFRNAMTLPRQLSLATVSGKIIMKNYPIVEAKDLRTQETKIPNFKIA
ETMVDHRIPSNTGAFEMELEIHKQSAKTIGFKLQNNKGEFVDFVINVSEQSIAMDRTHSGISNFSPSFPTSTSVAMD
LADKYKLRIFIDKASIELFVNEGELSMTNTVFPTEPYNQISFYAKGGKCEVSNLVMYDLRSIH
>Hrad_GH32_3, ON703153
MMKRARAFALSGVLCFTPVAPGFQHLVVSAEADVVNSETVLYDGLAITKGDSDTVRYDSGKYYIECNGDAFAVSDIE
LTNFVYEADVEFGRIGGAVGIIFGVENPNEPTEQAVVANLNPSNGEARMFTFGQSGGSDLASSVRREDWIGVNHFRL
KIEAVDGNIIYYINDEMIMNVSNQSTSGYIGIMGWNTDITFHSASVSELAGLDANHIGIGETFNTNLTNWRADKGIW
QLTEDGIQGKGLGNSPIFSRDRASNFTLEADMNIEGNVGGSIIFRSNASHTEYYAADIAYWDTETGHKARLIKYYLE
NGVMREAEIGEVYVLPDQNTNQYHLKVEALGDQLKFYVDDILAVSVNDDTHMTGFFGLNVCSIIGTFQNVIYEELDI
GDVATLTGLSFNEEVSLDETFDSQVSDYTFTVPYDMDRIQITPEIINEGTIKIKGESNEVVVLSEEESGAIDLHCGT
NLVKILVKTLENGYTKTTTLHIFRNNDPSEVYDVPYRPQYHYSPQE**HWVNDPNGM**VYFEGEYHLFYQYYPGANVWGP
MHWGHAVSEDLIHWKELPIALYPDGNGNIFSGSAVIDVNNTTGFFDGIEGGGLVAIFTHDNPAAGQQYQSLAYSKDK
GRTWIKYEGNPVIPFSQDPLHN**TAFRDPKVF**WHEEGNQWLMIAAGGPVRLFSSNDLKEWKPEGMYP**NLHTECPDL**FP
LEVEETGETKWVLNCGGRDYIVGDLEKDENDIWQFIPLTDRLTLNFAPDSYAAQTYDSTPDGRRIMINWMNNWDYCI
DVEKVTGDSGFNGNFTLQLELKLKLNQDGQIRLYQEAIEEYQDLRDSDLATIITGQVIEPDGNNLLEGFRGSQYEIV
AEFTPDSTATEVGFKLCKGLGETIVKYDINTETLTLDRGNSGSSPSARFLGSYSHKTTKTADGKIKLQIFVDTASIE
IFGNDGESTASALIFPKFGDDGVEVYSKGGNVTADITIYPLKSIWEDVRTESVSNQPSKVFLDAQKTNLYVGDRLTI
NAKVLPQIAGQDVIWSITSEDGRENVVSVSVSQNSNSIVIEAQNRGIITLSATASEGISNSISIKVKPEQIGFLSNLN
NWVIQSGDWDMDTTGYTGNYNDNGFVVAEYDNDDFIYEVDVTYHSGQAIGLLFRSQTQIPRGYVANIDGIHRGGSVR
IFTYGGADVGQFAPLRFELTEGTTYHLKLEVEGNHFKFYINGELYLDVIDTLNNFPTGEYAGLYIYRSEATYQNAMF
TAMGTDIAPEPTPEPTPEPTPEPTPEPTPEPTPEPTPEPTPEPAPTPSTGKTKPSNSGSATVAHKNPQTSNNASVGN
SADWRKEAGGWRMLIGTNTYAKSEWKLVNNLWYFFNSNGFMETGWVLVNNQWYYFNKDGAMITGWLFNNNKWYFLKS
SGAMATGWILPEGVWYYLNENGEMTILPITYKGNVYEFSQNGACINP
>Hrad_GH32_4, ON703154
MKKIVKSVSYLIMAAILAGCSPGDIAIDDFESGTFDKWAVEGDAFGAKPAQGAYPGQQEVKGWEGKYLANSYNGGDD
ARGTLTSAPFTIERDCINFLIGGGMHDSLYIELIVDGKNVCTSRSLAESETLEWESWNVKAYKDKKAVIRIVDNQQG
GWGHILVDEIAMSNQSKSKIEKDYRLSLRIDKKYLLVPIEDKGTISHLHLEAEGKVVSPQLDIRVAQSKIDYWMPID
VEQYKGKELTLLFDQLAKDNIGYSQIKQSDDYHFDYNEKYRPHYHFTPKY**GWMNDPNGM**VYVDGEYHLFYQYNPYGS
AWGNMNWGHTVSKDLKKWEHLPVAISPDSLGAIFSGSAIIDKDNTAGFGKGAMVVIYTSAGKVQTQSIAYSTDKGRT
FHKYDHNPVLSDANH**VDFRDPKVA**WHEATKQWIMTLATGQTITFYASPNLKEWTKLSDFGTGIGGH**GGVWECPDL**FP

MTYNGQTKWVLFVSINPGGPNGGSATQYFIGGFDGKTFKADNLPYPLWLDYGRDNYAGITWSNAPENRHIFIGWMSN
WQYAQDVPTINFRSAMTVPRELKLAHNGKHLIVANPPVKEIYDLRSETKEVQDLLVEKTSTIDKLLDNNHGAYEIEM
TVQLNKASAFDFKLINKKGEEMSFVFDVQNGNLAVDRSKSGAIDFSKDFAANGSKAPLTKKGEYKIRLLVDKASTEL
FVDNGELVQTNTIFPSEPLNTLQLNSQGGAIRVKDIKIYNLK
>Hrad_GH32_5, ON703155
MKGRFNMSDKTHKQNLIKAQNAVIEKESFAQKGSYRQQYHFMALT**GWINDPNGL**IFWHGQYHLFYQYNPYASVWGSM
HWGHAVSNDLINWEYLPIALAPSETYDDNFHGGCFSGSAVSNDDTLMLIYTAATKCGDVLLQTQCLATSKDGITFEK
YENNPVIKELPPQAS**SDFRDPKVL**KYKDTWYMVVGSSLGKGARLGGDGCAQMYKSTDLINWEYCGVIAKSNGKL**GTM**
WECPDLFPIGDKWVLMFSPMFYGKRKTVYLLGDMDFDNYSFSWDYEGEIDYGFEYYAPQSFLDNKGRRILIAWANGW
EWMPWWKDFGPTASEGWCGHFAIPREVRIAKDKGLQFVPINELESLRTDCKSYPSFILGSEEKKEIHAGDGIHCELL
INIDVGASTARQVILDVRQGENERVRIVLSLKDKEVLFSRINSIGQVEELKQQIFADFGKSLLTMHLFLDTCSVELF
VMDYKMSFSGNFYFNENSNLIFLESKCGYTKINSVHTYALSIH

Micrasema longulum

>Mlon_GH32_1, ON703151
MINNLFFAFFILVICACGTQKNAIITEQHRPQFHFTPPK**MWMNDPNGL**LYHEGEYHLCYQYYPDSTVWGPMHWGHAV
SKDMLHWKHLPIALYPDQYGYIFSGSVVVDENNTSGFQKGAEKPLVAIFTYHSMAGEKAGTNDFQTQGIAYSLDKGR
TWAKYTENPVIKNTGI**KDFRDPKVQ**WHSPTQQWIMTLAVADHVEFYASKNLKNWQKVGEFGKTEGAH**GGVWECPDL**F
PLKIDGESTEKWVLLLSIGNGAPNGGSGTQYFIGDFDGKSFKNDNTPDNFLWFDYGRDNYAGVTWHNAPNNRRLFLG
WMSNWQYAQKVPTQTWRSAMTLPRELSLKNTPNGIRLFQKAVNEQITLKSNETPLASQTIEAMYPLKNTSKCNEITL
NFDISRATSQDFGVVLSNTKGEKVWVGYEKSTNRFYIDRTDAGKKDFEAGFAGRHYAPRSATSDVLKMNLHIDVSSV
ELFADDGAVAMTDVFFPNEDFNALAIFSKGGEARLLDGRIWKLK

Wasps (Hymenoptera, Insecta, Arthropoda)

Trichogramma pretiosum

>Tpre_GH32_1, XP_014223192

MSWSKHVAVSGLLLLALLLEASKSQPTKAPYSWYPKYHLASVK**GWMNDPNGL**IYHKGYYHAFWQHYPDAPSWGLMHW
GHARSRDMLNWEHLPIALAPSLPDDIDGIFSGSAIVKDDKLHLMYTGVSLDNKRQRQMLAVSDSEEDDRFRKLGTVI
VRDGSE**LNFRDPKVW**QEADGTYWVIVGTQTPDGRGEVLLYNSTDLMSWTYDRVLARSEKKY**GFMWECPDM**ISLNGKR
LLLVNPQGMEPAGYDYQNLYQTGYFVGEWHPGSDYKIEREFREIDHGHDFYAAQTFLAADGRRILIGWLDMWESEFA
EKREGWAGQFSLPRELTLNDAGDLEIRPIKEVKEARKKRKAATLESSEPAKSSEQADAAGERVFLSGEQLNRNEIVV
DFNLKETTCEEFGIQLKGNDEQFEGVKIYVDREKSRLFLERFYPKYNIERSSRSVPIDLSKPELSLDIFIDSSSIEV
FVNDGQAVMSSRIYPNEDQRQFSMYGTKGSLILKEFNVYDL

Trichogramma brassicae

>Tbra_GH32_1, ON703209
MSWSKQVAFSGLLLLALLIEASKSQPTKAPYSWYPKYHLASVK**GWMNDPNGL**IYHKGYYHAFWQHYPHAPSWGLMHW
GHARSRDMLNWEHLPIALAPSLPDDIDGIFSGSAIVKDDQLHLMYTGVSLGNKRQRQMLAVSDSEEDDRFRKLGTVI
VRNGSE**LNFRDPKVW**READGTYWVIVGTQTPDSRGEVLLYNSTDLMSWTYDRVLARSEKKY**GFMWECPDM**ISLNGKR
LLLVNPQGMEPAGYDYQNLYQTGYFVGEWHPGSDYKIEREFREIDHGHDFYAAQTFLAADGRRILIGWLDMWESEFA
EKREGWAGQFSLPRELTLNDAGDLEIRPIREVKEARKKRKAATLESSEPAKSSEQADAAGERVFLSGEQLSRNEIVV
DFNLKETTCEEFGIQLKGNDEQFEGVKIYVDREKSRLFLERFYPKYNIERSSRSVPIDLSKPELSLDIFIDSSSIEV
FVNDGQAAMSSRIYPNEDQRQFSMYGTKGSLILKKFNVYDL

Trichogramma evanescens

>Teva_GH32_1, ON703210
MSWSKQVAFSGLLLLALLIEASKSQPTKAPYSWYPKYHLASVK**GWMNDPNGL**IYHKGYYHAFWQHYPHAPSWGLMHW
GHARSRDMLNWEHLPIALAPSLPDDIDGIFSGSAIVKDDQLHLMYTGVSLGNKRQRQMLAVSDSEEDDRFRKLGTVI
VRNGSE**LNFRDPKVW**READGTYWVIVGTQTPDSRGEVLLYNSTDLMSWTYDRVLARSEKKY**GFMWECPDM**ISLNGKR
LLLVNPQGMEPAGYDYQNLYQTGYFVGEWHPGSDYKIEREFREIDHGHDFYAAQTFLAADGRRILIGWLDMWESEFA
EKREGWAGQFSLPRELTLNDAGDLEIRPIREVKEARKKRKAATLESSEPAKSSEQADAAGERVFLSGEQLSRNEIVV
DFNLKETTCEEFGIQLKGNDEQFEGVKIYVDREKSRLFLERFYPKYNIERSSRSVPIDLSKPELSLDIFIDSSSIEV
FVNDGQAAMSSRIYPNEDQRQFSMYGTKGSLILKKFNVYDL

Eupelmus annulatus

>Eann_GH32_1, ON703203 (**active site region 1 not detected**)
MFTSFYSEPTTLPDGTKVEKGTQSQSIAYSNDKGNTWHFYDKNPVIRSPPGKYASE**FKEFRDPKVFW**YAPHNKWVMI
NMVSQKKVALIWSSKNLKDWRLMSEFSSRFTP**NEIWECPDI**FELKVIDQNESKWVLLVSTNPGGVARGSGMHYYIGH
FDGYKFTEDTDIRYNHIKWLDYGSDFYAGITWNNVDTGRYIVAWVNNWDYAQARHDEYKGAIGFVREMSLITVDGTI
KVSQKPIANLSNYIKETDEYGLEKIRNGVEILKNKAYELRVQLTDVGESGFIFVLEDEKNNVEAEIKYDDKNKTLSI
NKINRYPGETEKYVTHYAPYECTKDQETFRIFVDSNTLTLFTTKGDVVFTELLISYAENRKFYLKEGGEIKVDLTRY
LLEF
>Eann_GH32_2, ON703204 (**active site region 1 not detected**)
MSWGHAVCKDLVRWESKGIGIHYKEDTKEFLFSGSAVYDVNNTSGFGTAENPPVVAIFTSFYSEPTTHPDGTKVEQG
TQSQSIANSTDKGNTWQFYDKNPVIRSPPEWYATE**FREFRDPKVFW**YAPHNKWVMVTMLAQKKKALFWTSQNLKDWT
VMSEFSSRFTP**DGVWECPDI**FKLNISGQNESKWVLLVSANPGGVAKGFGMHYYIGDFDGYRFTEETDEKYHHIKWLD
YGSDFYAAISWNNVDTGRYLVAWIDNWEYAYATHDEYKGAIGFVRELSLITVDGTITVSQKPVGTLANYIKEKKQYS
LEDVRTGVEILMNKAYELIIELSDIGESGFIFVLQDDKNNVEAEIKYGDEGKTLSIRKKNRDPKVPEKYVMHYAPYK
KNILENRETLRIFIDSNTLALFSTRGDVAFTELLISYAEKRKFYLKEGGKINVNLTRQLLEF

>Eann_GH32_3, ON703205 (**active site region 1 not detected**)
MFTSFYSEPTTLPDGTKVEKGTQSQSIAYSNDKGNTWHFYDKNPVIRSPPGKYASE**FKEFRDPKVFW**YAPHNKWVMI
NMVSQKKVALIWSSKNLKDWRLMSEFSSRFTP**NEIWECPDIF**ELKVSDQNESKWVLLVSTNPGGVARGSGMHYYIGH
FDGYKFTEDTDIRYNHIKWLDYGSDFYAGITWNNVDTGRYIVAWVNNWDYAQARHDEYKGAIGFVREMSLITVDGTI
KVSQKPIANLSNYIKETDEYGLEKIRNGVEILKNKAYELRVQLTDVGESGFIFVLEDEKNNVEAEIKYDDKNKTLSI
KKINRYPEETEKYVTHYAPYECTKDQETFRIFIDSNTLTLFTTKGDVVFTELLISYAENRKFYLKEGGEIKVDLTRY
LLEF

>Eann_GH32_4, ON703206
MRFYFNKYIRVDMYKLCDLNYDYTFSALYIFSLFLLATAYGKDRALNSPLLFTPPK**GWVNDPNGL**VYVDGTYHLFYQ
HNPYANSWGNMSWGHAVSKDLVRWESKGTGIHYKEDTKEFLFFGSAVYDVNNTSGFGTAENPPVVAIFTSFYSEPTT
HPDGTKVEQGTQSQSIAYSTDKGNTWQFYDKNPVIRSPPEWYA**TEFRDPKVFW**YAPHNKWVMVTMLAQKKKALFWTS
QNLKNWTVMSEFSSRFTPD**GVWECPDIF**ELNISGQNESKWVLLVSANPGGVAKGSGMHYYIGDFDGYRFTEETDEKY
HHIKWLHYGSDFYAGISWNNVDTGRYLVAWIDNWEYAYATHNEYKGAIGFVRELSLITVDGTITVSQKPVGILANYI
KEKKQYSLEDV

Tetragonula mellipes

>Tmel_GH32_1, ON703200
MTATWSDKSPLLDIVNIEQGLGFSQFRPLLHITPNPYANKDYVGDYWQTFPADNAAIANVKAENTPWIR**PWYNDPSR**
LIWDEKQNIWRCFTITSSTAELNGDWHSTWMEMASPDLCNFVNNRIPFYTKGMEYPDLWGGSIVIDEHGVTPFGKGS
VLYYLTMPGVPGQTSSQQSTSLWVAPDLGMTPVFYDIVLENPGAGDIVHAPG**MDFRDPRVD**WDDDHKKFVMKLTVGY
GITFYESTDGISWTNLSTIDLSD**WQQIETPDL**VPMYTPDGIKKWVLFFSLKRWEGKDASSIGYMVGSWDGKTFHPDQ
NTPKRVNWGHDYYAQAISQHKGDTYCWAWMGCWTNGDLPTQGFAGNHSIITKLTLDKDSDGTYGLRFNLMPKSINMY

NNHTDDWSAPTLSAAGQQTWTPPLKAPGICWRVDLELLRKDTTPLPDEIQFDFCRNGATYTRLALRPKDGTFYLDRS
NSGLTPLNKGSQYTQDSWKAIQEGTLPKEAFYTVTVMFDVSTVEIIINNQSYLSTLIFPPEDAFSMVLSAIGDGEVV
LRRATMRY
>Tmel_GH32_2, ON703201
MDTKYWNIDRLGRLYKDWDPKVLKELETKIAKSHWRPGYHITTKT**GLLNDPNGF**SYFNNQWHLFYQAFPYGAVHGLK
SWQHMVSDDLVHWKFIDKPMVPDLYSDRNGCYSGSAINVDGKLFLMYTGNTFEGEEMARHPYQLGAWMDDDNNISKL
DHPLIESEPAGVT**GHFRDPQVI**KNGDYYYAFLGAQTVDNAGEILTYRTKDIENGWKLYKKLDLGTDNL**GYMAECPNV**
GFIDGKVVVIFCPQGADKHEFHYDNVHPNAYIVADDLDFEEMKLVNPSEMKQLDEGFDYYATQVLNAPDGRLLSVGW
IGLPDTEYLSDQEGWEGELSMVRELHIENGDLTQRPVQEFKQLIEEDEASDTDLQKHIEAAPFDEHFNLNLESEDSY
LRLSYDEIDQEWTLKRKNVGQEVSIRTFKGEASSVDFYLDNTVFEIYILGGKITMTGQFFHDGNEVMVNSENMSVKQ
NKMSNM
>Tmel_GH32_3, ON703228 (1)
MADLAPIQLTNERYRLDYHLQAPS**GWINDPNGF**CYFRGYYHMFYQYYPYGAEWGPMHWGHARSKDLINWQTLPIALT
PGDEEDRSGCFSGSAIVKDDTLYLIYTGHNCYDDSDPDHYWQNQNLAYSTDGIHFTKYEHNPIIATPPTDNT**QEFRD**
PKVWEHDGAYYVVLGSQTKEHLGRVLLYKSTDLRSWDYLGPLTQSQQAELE**GYMWECPDI**FRLNGHDILLTSPQGIT
AQKEQYLNLHQTGYFVGQLDYQTPKLTRGDFHELDAGHDFYAPQTMLAPDGRRIMMGWLAMWESEMPEQADGWAGAL
TIPRELVWRNNQIYMQPIQEMKQLRQKQLAKQTWNLADTPVICQGQATAEINLTLDLKQFAGQEFTIQFQDDQSTAQ
VELTYIRSDNKLVLRRSDRSDKRFATIAEQDTLQLQIFVDKSSLEIFINEGATVFTERYYFDGQPTVKLVGDAGQIG
GTVHQLANDVVQYH
>Tmel_GH32_4, ON703228 (2) (**Active site region 1 not detected**)
MYYQYNPKGTQWGNMSWGHATSKDLIHWQEQSVAIPMLQNQPWEDFTYTNQNGDLANQRVRYVGSPTADWGNTPNSK
AIFSGSIMVDKQNVSGLGKNAVLAFYTSSYQLPERKDDHLDNGWGTWVGLSDIQEQHLAYSLDNGKTFKQYSPDGNS
KQPQPIVPVTANPAGDA**ANFRDPNVV**YDRQHQQYIMTIVSGQQALLYKSKDLLHWDYASNIKRQKDVG**LGVWECSSL**
VPMKVKNSSATKWILFMSVQQGSHATGSGMQYFVGNLDAKGTWHPDSGQTLAYPHTLDYGEDFYAGIPFANLPNDRT
IMLAWANNWSYNSESPATQWNGNMTLPRDLQLVNDAQATDGYTVQNSAIAETQKITQANALPTGKQSLNLSSNEEQL
PFAGKNYKLTATLKWDPEKKPQSVGFNLRQTPDQKYYVTIGYDVANSKVYVQRLNTGEASMGAPRDQMNAVVKADNG
QVKITAYIDETMAEVFANNGAASITQNFYLQSDLINQQDTTGISAFANGGSAQITDLQLQPMQSIW

Macrocentrus cingulum

>Mcin_GH32_1, ON703208
MLQTLIIIILCLSAAITLAQTDKQSSSELQKLEKYIQDKKPQINQRYRLQYHVAGPV**GWINDPNGF**SYYKGEYQLFFQ
FYPYDSVPGKIHWGHVSSRDLINWKQLPTALLPDDEQCFSGSAVVLNNTLVLMYTGHRTLSSTIYNESQYLATSDDG
LVFTKYKGNPVLPRSPNGS**PDFRDPKAW**RHGDHWYVILGSKNNANHGRVLLYRSEDMITWEFLRVLAESNGTL**GYMW**
ECPDFFELDGKFILLMSPQGIAAQGDRFKNLYQTGYIVGNFNYETFEFIPEVEFQELDFGHDFYAAQTNEVDGKRYV
VAWFNMWEQPHPEAIDGWAGAMTIVRELKLIGNQIIMKPLDGMVSLRNKTVLQGELKVNQTINFNKTAEIIVDGDLY
QKIELLIDGHDGGEKVSIRWDPNVRKVVVDRGSQDIRQVEWNPLNSLTWRIFLDSSSLELFCGEGEVVFSSRVFPTG
NWRLTNLSPQILNVHAFTLKQSVPI

Flies (Diptera, Insecta, Arthropoda)

Mayetiola destructor

>Mdes_GH32_1, QIX12404
MSRANILLCTTLLVLISLTSAVYDELYRPQIHLSPPS**GWMSDPNGL**VYHDGVYHVFYQHNPNDTKPNDFPQMYWGHA
ISTDLVHWKNLDIALSPPTDKSFFSGSGIIDQHNVTGFQIDDDKKPIILLFTSNNMSTAEQEQWIAYSNDAPEYKTF
EYYKNNPVIKKLNLEGEKS**INFRDPQII**EWETGQYVTLITQDNKSMIYNSRDMINWELVSQFGEYEGKH**GGTWECPS**
LFTLNVTINGTMVEKQALLITLTDAVIPAIQYFIGSFDGKTFKNENPPETILWYDYGPDSFAGSTFNHVPSNRRIFL
SWMSRWEYAKRVGPGPWSGNMGLPRELNLKQVGNDIRFASLPVSELKVLRMSQFREKNVTISSKYVSKMVEDDGEDK

HTVDIEMILDLSNLKKGDKFDIVFFDKQDRLNVTLKGNEFTLDRSRAGKTDFNKFGIPWKAPRFIDSPELKLRIIID
RSSIEFFADDGLTVMTALFFSKEDIASKMAIHVHSSSLKSTVHLRELNAYKMKSIWN
>Mdes_GH32_2, QIX12405
MLQNRILFGTSLMVLVSFGSALYDEPYRPQLHFSPPS**GWMNDPNGL**VYHDGVFHLFCQYNPAAPLHGNIHWFHAISS
DLIHWKNLGIALAPQEGNLIFSGGAIIDHDNVTGLQTENDKKTLILVFTAHNIVEEKQWLAYSNDGPEYEHFQYYNH
NPIIPNPNPKTY**KDFRDPSVF**KYEDHFVMVLAAYDHIMIYNSPDLLEWNLVSEFGIDEGSH**IGTWECPSL**FPINVTI
DGVEIEKYVLIISLTDNAIPSMQYYIGSFDGQHFTNENSKETELWLDYGPDSYAGITYNQLPDGRRTFISWLFRWEY
ATHMNFSIWNGQAGIARKLMLNMIGDRIQLSSLPVREFKSLRIKQLANKQRSIPIEDKLSFEFSKNGTKGRKLLLDL
EMIFDLTNLKGDDQFDIVFFDTNDNLNISFNGNEFTLDRSNAGKTDFPNFGRLWKAPRFVKSLELKLRIIIDQSSIE
FFADDGLTVMIAFFVSDEDIASKMAIHVHSSSVTSMVYLKKLNAYQLKSIWN
>Mdes_GH32_3, QIX12406
MSRTNILLCTTLLVLISLTSAVYDELYRPQIHLSPPN**GWMSDPNGL**VYHDGVYHVFYQHNPVDTEPDKFPQMYWGHA
ISTDLVHWKNLGIALSPPTGRSFFSGGGIIDHHNVTGFQIDDDKKPLILLFTSYNISTEEQEQWIAYSNDAPEYNTF
EYYKNNPVINQLNLEGEKS**INFRDPQIY**EWETGQYVTFISQDNKSMIYNSRDMINWELVSQFGEYEGKH**GGIWECPS**
LFTLNVTINGTMVEKEALLITLTDFVIPAIQYFIGSFDGKTFKNENPPETILWYDYGPDSFAGSTFNHVPSNRRIFL
SWMSRWEYAMSVGLGPWNGNLGLPRELHLKQVGNDIRLASLPVYELKDLRTSQFRKKNVRISSKYVSKIVEDDGEDQ
HTVDIEMILDLSNLKKGDKFDIVFFDKQDRLNVTLKGNEFTLDRSRAGKTDFNKFGIPWKAPRFIDSPELKLRIIID
RSSIEFFADDGLTVMTALFFSKEDIASKMAIHVHSSSLKSTVHLRELNAYKMKSIWN
>Mdes_GH32_4, QIX12407
MVKNNILPVVFLIVLSSFSSALYDEPYRPQFHFSPPS**GWMNDPNGL**VYYDGLFHLFYQHNPVAPIHDNIHWGHAVSP
DMVHWKNLAVAIAPYGKELIYSGSSIIDHNNVTGLQFDDHIQPLIAIFTAYNNDTGEQKQWLAYSNEGPEYEHFQYY
NYNPIIPNPNPSVQ**KDFRDPAVF**QFNNHYVQVVAAHDHIMIYNSLDLLEWKLVSEFGMDQGSH**IGTWECPSL**FPINV
TINGLDVEKWVLIVGLTDFAIPTTQYYIGSFDGQTFINENSEETVLWLEYGPDSFAGITYNQLADGRRPFISWMNRW
QYSKSFNFTAWNGQMGLARELKLTKIGNEIRLSSLPVREVQTLRINPVRRQNVTITNAFVFNIVEHDDEQSQHQVDV
EMTLDVTNLKAGDSFNIVFFDKKDSIKISFKANAFILDRSNAGRTDFPNFGLLWKAPRFIKSCELKLRIVVDRSSIE
FFAMMACLL
>Mdes_GH32_5, QIX12408
MLRINIQLIISIVVLDSYSSELYDELYRPQLTFSPPS**GWINDPNGL**VYYDGVFHLFCQYNPNSTLHGNLHWYHAISP
DLVHWENLGIALAPTNGNLIFSGSAIIDHGNVTGLQANDDKKTLIAIFTAHDLSTNEENQWLAYSNDGPEYRQFEMY
KNNPILPNPNPSEQ**KDFRDPAVF**KYNDHFVLVLAAYNRILIYNSPDLLKWTFVSEFGVDEGSH**TGTWECPSL**FPINV
TIDGVQVEKYVLIVGLTDNSIPTTQYYVGSFDGQTFTNENSKETILWLDYGPDSYAGITYNQLPDGRRIFISWENRW
QYAQQLNFNVWNGQMGLARELTLNKIDNRILISSLPVRETKMLRIDHVRKQNIPIENYLSFEIAKFDDENRKQSVDI
EMMLNIANLKAGDSFNIVFFGVNDALNISFNGNEFILDRSKAGRIDFPNFGRLWNAPRMIESSILKLRIIIDRSSIE
FFADDGLTVMTALFYSEEDIASKMVIQVHSASLDSMIAVKEFNVYKMKSIWK
>Mdes_GH32_6, QIX12409
MVHINILLIALIMVLNSFSSGLYDEPYRPQLHFSPPV**GWMNDPNGL**VYHDGIFHLFYQHDPKTTIQGQMHWGHAIST
DMIYWTNLDIALTPPKGIAYFSGGAIIDYNNVTGFQMSANVKPLIAIFTAHNRATYEQEQWLAYSNDGPEYKHFELY
ENNPIIGKLNTDPSKP**IDFRDPAIT**KWGDHFVLFLAQYNKSLIYNSLDLKNWELVGDFGEYDGSH**SEVWECPSL**FPL
NVTINGVNVEKHVLIVGLTGSVLPTTQYFIGSFDGKKFTNENSPETTLWVDYGPDSYAGITYNQLPDGRRVFVSWMN
KWEYAEQLNFNVWNGQMGLMRELKLKQVGDQTRLVSYPVREEEKLRTYVVRKENIKISTNRCVYKITPDGKTKHTVD
MEITLDVTDLKKGDSIEFTFFDKNDNLTISLTENEFTLERNNTGRTNFSNFTRPLKAPRLIDSPELKLRIIIDRSSI
EFFADDGLTVMSALFFSDEDIASKIAIQVYSSLADSIVHLKNFNAYQMKSIWN
>Mdes_GH32_7, QIX12410
MFRVDILLFALSIVPISNAVYDELYRPQLHYSPAK**GWLSDPNGL**IYKEGIYHLFFQCRPDHIAQGSVHWGHSISSDL
IHWKALDTALYPPVGHEMFSGGAIFDYKNVTKLQTNENVPALILLPSAAVWSTREQNIWLAYSNDGPEYNKFTYYEK
NPVIRGPSSYGKLI**TAFRDLTVF**KYHDNYVSILVQYNRTQFYSSHDLIDWELISEFGEYEGSH**AGRWECPSL**FPFNV
SIDGKQVEKYVMIITLTDYVHPVHQYFIGSFDGKKFTNENTKETILWLEYGPDSFAGVTYNELPDGRRIFIHWMGRW
EYVANLNFSPWLGQLGIPRELNLIKVGDQIRLTSEPVREMESLRINHVRRQNINITNEFSYQIADSAKKNHLADIEL
MLDLQNLDSRDAFQIVFSGKSDEFKIIFKEKEFILDRTRAGKIIPNFEGVKISQGNFDNPNFIRPTKERNFEDLWKA
PRLIDSSNLKLRIIIDTNAIEMFADDGLTSMCALFFSKDGIASKMTIQVHSSTKKSHIYLRDMNVYEMKSIWHKDES
TKSKFNFSDNKKQKIVK
>Mdes_GH32_8, QIX12411

MAPERISSESYDEQYRPQLHYSPPS**GWINDPNGL**VYHDGIYHMFNQNNPNDVIPGRISWGHAISTDLVHWKTLPCAI
PSTDENSIFSGSAIIDDDNVTGLRTDDQIKTLIAIFTAHNLSTNEENQWLAYSNDGPAYEKFHFYEKNPIIPNPNPK
KQ**IDFRDPSVF**KYKDRFVILLAAHNHIKIYNSLDLLQWKFVNEFGLNDGSH**AGTWECPSLF**PIKVTIDGVEVEKYVL
IVGLTDGAIQTTQYFIGSFDGEKFINDNFKETELWLDFGPDSYAGITYNKLPDDRRIFISWMNNWLYAQHLNFNVWN
GQMGLARELKLIQVQDRILLSSLPVHELEMLRINPVSIQNVLIENDFIYKIDRNDSIAKKHLVDIEMNLDLSKFKKG
DSFDIVFSDENDEIKISYKRNEFILDRSKAGRTDFPNFGRLWKAQRFIDNSNLKLRIIIDRSSIEFFADDGLTVMTA
LFYSKEDIASQMAIYVHSSANGSIIDLKKLNVYQMKSIWSQ
>Mdes_GH32_9, QIX12412
MLKINILIVAFSMIPGSFSLYDEKYRPQIHFSPPN**GWINDPNGL**VYFNGIYHMFFQNNPYNTVPANNIHWGHAISRD
LIHWKSLSTAIYPKDGNLIFSGGAIIDEHNVTGLRTNDNRATLIAVYAAHHLSTNDESQWISYSHDGPFYEKFQYYK
KNPIIRNPNRRQ**KDFRDPFVF**KYKDHYVVTIAAHDRIMIFNSRNLLNWKLVSEFGVDTH**GRVWECPSL**FPINATING
AKVEKWVLTISLTGNELPNQQYFIGSFDGKNFKNDHSKDVKLWLHYGPDSYAGIVYNQLPDGRRIFISWMNKWEYAQ
QLNFNRWNGQMGIAQELRLIQVKDQIRLASLPVRELKKLRTKQLEQKKNIKITNDYVYKLTKNGCHKAEDKLDIEMT
VDLKNLKAGDTCNFVFSGSNEYLNISLKGNEFTLDRSHSGRTNFTNFAKPTKCKRQSDDSKLEMRFVFDLSSIEVFI
DGGMTTMTALFYSKEDIASKMQIRTHAKDSSIILKDLKVYRLKSIWNEFSIQPSLDKFPCSE
>Mdes_GH32_10, QIX12413
MLQNRILFGTSLMVLVSFGSALYDEPYRPQLHFSPPS**GWMNDPNGL**IYHDGVFHLFCQYNPAAPLHGNIHWFHAISS
DLIHWRNLGIALAPREGNLIFSGGAIIDHDNVTGLQTKNDKKTLILVFTAHNIVEEKQWLAYSNDGPEYEHFQYYNH
NPIIPNPNPKTY**KDFRDPSVF**KYEDHFVMVLAAYDHVMIYNSPDLLEWKLVSEFGIDEGSH**IGTWECPSL**FPINVTI
DGVEIEKYVLIISLTDNAIPSMQYYIGSFDGQHFTNENSKETELWLDYGPDSYAGITYNQLPDGRRTFISWLFRWEY
ATHMNFSIWNGQAGIARKLMLNMIGDRIQLSSLPVREFKSLRIKQLANKQRSIPIEDKLSFEFSKNGAKGRKLLLDL
EMIFDLTNLKGDDQFDIVFFDTNDNLNISFNGNEFTLDRSNAGKTDFPNFGRLWKAPRFVKSPELKLRIIIDQSSIE
FFADDGLTVMIAFFISDEDIASKMAIHVHSSSVTSMVYLKKLNAYQLKSIWN

Contarinia nasturtii

>Cnas_GH32_1, XP_031633595
MKRIVTLFLSALLCGALAEYQAQQVLSSRPTAVNKNFTNEKPHNNEGDSVPAEQPLTKVAELESSESTTTFVSNTFN
GTVEYIQTTDELQEVDPIEKQPYISIKGKKPSYKEKYRLQVHFSVPS**GWMNDPNGL**VYAGDYYHLFYQYNPKSTTFG
SPHWGHARSKDLIHWENLPIALKPIDLPEGVGDIFSGCCVSDKNNVAGFTPKSMTDTNALIAVYTINSNVSQTQAIS
YSLDNGITWTAYKNNPVIQNPGV**ADFRDPNII**ERDGKFYMTLAVHDRISFYSSKDLKEWRWLSDFGIKPNEGDK**SGV**
WECPSLFSINDEQGNVHDILIVSENGPSRGSLLQYFIGKFDGNKFNSYDKSKLLWLENGFDNYAAIPYHNDPLKRLI
IIGWLSNWLYTQGIPTSTWRGQMTIPRELSLKHINGNLYLAQRPVDELKNIKDSSKSWSLSKNLDIKNNQTFDITSI
IPLKIGSVLSLEYVFDIGNVTNGKVGLKFGNSLGEFVTFLYNIEKNEYEFDRRNSGNVSFDIRFADIVPTATRISND
SLLSGQIILDTASIEIFADDGLNTFSALFFPTEPFEHIQVTNGIEDYGKSMTIQKLNVSALNSIWKH
>Cnas_GH32_2, XP_031621237
MKSLCTLFVVLFLTEYSITYKIITGEYAEMYRPQLHYSPPK**GWLNDPNGL**IYENGLFHLFYQCHPNDTNVIREQHWC
HAISSDLVHWKTLPTALYPPNKGFAMFSGGAVFDHNNITGLQTNSNTPTLLLMPAAAEWSSREQNIWLAYSNDGPEY
ATFKYYKNNPIIAGPPNSEIV**TAFRDPIFF**QHKDYYVAIVASYNRTKFYNSRNLLNWEFVSEFGEFDGSH**TGRWECP**
SLFPFNVTINGKQVEKYVMIITITDYQNPVHQYFIGSYDGKKFTNENSRDTILWLDHGPDSMAGITYNALPDGRRIL
ISWMGRWEFAGNLNFNAWNGQMGIPRELNLIKVGGRIRLTSQPIREMESLRINPVRKQNINITNDYTYKIADAEKKN
HLADIEMTLDVKQLKTGDSFDIVFSGKKDQLKISFKGNEFILDRSRAGKIIPNFIDTSLEKGNLDNPNDELSVPKLT
FKDLCNAPRLINSSNLKLRIIIDTNAFEMFADDGLTCMNALFYSEYGIASKMTIQVQSPSKKSKIQLKELNVYEMKS
IWQKDASSSRTGKKRADPSPKNKGKKSKY
>Cnas_GH32_3, XP_031631695
MFRIGSFVVVAWMVSISDAIYDEVYRPQLHYSPPK**GWLSDPNGL**IYGNGIYHMFHQCQPDDANTISDIHWGHAISTD
LIHWKTLEPALYPPDKGTALFSGGAIFDYKNITGLQTNSNIPTLILMPAPAVWKTRKQNVWLAYSNDAPEYVKFKYY
EKNPIIRGPPDTEII**TAFRDLTVF**KHQDYYVSIIATYNRMQFYHSRNLLKWELVSEFGEFDGAH**TGRWECPSL**FEFT
VPLGNGKQIKKHVLLVTITDYAQPAHQYFVGSFDGRKFTNDNSKDTILWVEYGPDCFAEIIYYDLPDGRCISMNWMG
RWEYAENLNFKNWNGQMGLPRELKLIKVGNQFRLTSLPVREINSLRINPVRKKNINIMNVYQHEIADSGKKRHLADI
EMTMDLKKLKAGDTFDIVFSGKRDELKISLKNNVFYLDRSRSGKIVSNYRNPNVKKGNIDNPDIELAKMDHSFGGLW

EAPRLTNSTNLKLRIIIDTNGIEMFADDGLTSMSALFFSEDGIASKMTIQVHSATKKSLIQLKELNVYEMKSIWHNE
EGIKKKIELSAHTKKLKSKKGGK
>Cnas_GH32_4, XP_031631694
MFRIGSFVVVAWMVSISGAIYDEVYRPQLHYSPPK**GWLSDPNGL**IYGNGIYHMFYQCQPDDANSISNVHWGHSISTD
LIHWKTLDTALYPPNNGFAMFSGGAIFDHNNITGLQTNRNTPALILMPSLFNRKNHRQNVWLAYSNDAPEYTKFKYY
EKNPIIPGPPETEI**AAFRDQTVF**KYQDYYVSIVVTYNRTQFYHSRNLLKWELVSEFGEFDGAH**TGRWECPSL**FEFTV
PLGNGKQIKKHVFLITITDFAQPAHQYFVGTFDGRKFINDNSKDTVLWVDHGPDCFAEIIYYDLPDGRCISINWMGR
WEYAENLNFKNWNGQMGLPRELKLIKVGNQFRLTSLPVREINSLRINPVRKKNINIMNVYQHEIADSGKKRHLADIE
MTMDLKKLKAGDTFDIVFSGKRDELKISLKNNVFYLDRSRSGKIVSNYVNPHFSKLGKGNIDNPDEVLPKMDHSFGG
LWEAPRLTNSTNLKLRIIIDTNGIEMFADDGLTSMSALFFSEDGIASKMTIQVHSATKKSLIQLKELNVYEMKSIWH
NEEGIKKKIELSAHTKKLKSKKGGK
>Cnas_GH32_5, XP_031633597
MLNSNVSIPLFFIIFLLAVNGHSYHDKYRLQVHYSIES**GWGNDPNGL**IYAGGYYHLFYQHDPNETVFIDRIRWGHAR
SKDLIHWETMPIAIDMYNDNSIYSGCCISDTNNVTGFAKNQTEPPLIAIYTLNKSNEKISQRQAISYSLDDGMTWTQ
YVNNPIIPNPGV**ADFRDPNVF**HRDGKFYMTLTVGDRIRFFSSVDLKIWHFESDFGVNPDAGDK**SGVWECPSI**ITLKD
EQGYVHDILIVSENGLSSGSMTQYFVGKFNGTHFINYNQSKLLWFDDGFDNYATISFNNNPFDDTIMIGWMSNWLYA
NNTPTSTWRGQYTIPRKVTLKTVDEKLYLAQRPIDELNNIIDSSKCWSLSQPTKLSGYQILDLTERIPFELGPAYTL
EFEYDIEHVTMGELGIKFSNSFDEFISYSFDLKNKTFEFDRRKSGNVSFSPRFANTTVFKRISNSNILSAKLILDTT
SIEKFIDDGLNTFTAIFFPTEPYNKIQLFAAFENARESAIISTLSVSKLKSIWSES
>Cnas_GH32_6, XP_031617397
MLNSLVFCIIFLVAVNGQNYDEQYRLQVHYSVKS**AWSNDPNGL**IYAGGYYHLFYQFYPNDIVWGPMHWGHARSKDLI
HWENMPIALEPYDKWMIFSGCCVIDKSNVAGFLSDRSNNTDQTEDTLIAIFTLKELNGTEQSEGMAYSFDAGTTWTQ
YVNNPIIRIPGI**VDFRDPMVF**QRNGQFYMTLDLGDRIRFYSSTDLKTWKDEFDFGVNPDEGDK**SGVWECPSM**VTLKD
EQGNEHDVLIVSVLAPEGCFAQYFVGKFTESHFNSYNQSKVALLDNGFDNYASVPYINDPLGRIILIGWMSNWLYSD
KIPTSTWRGQYTIPRELTLKTIHGQIYLVQQPIRELNNLIDPLRTWSLSKPMKVSGFKTIDLTEEIPFVLASAYILD
FEYDIGNVTQGNLSIRFSNSFDEFIAFRYDFSDNSIEFDRTNSGNFSFSSKLVKKEPRISNSNIFSGKVILDTSSVE
KFVDGGLNVFTNLVFPAEPYNKIQLFTAFENAEESVTVSKLNVTCLNSIWK
>Cnas_GH32_7, XP_031616780
MSKLFVKFLLFCTVSLLAVVADKATYNEKHRLQLHYSVPT**QWSNDPNGL**IYFNGYYHFYYQYNPYDTKMGSAIHWGH
ARTSDLIHWENLPIAIEPYDEGQIFSGCCVLDKDNVTNFAPNSTEDHTDIPMVALFSLVDGDKQSQGMAYSFDNGTT
WKQYHANPVIDNPGI**TDFRDPNVF**EHEGEHYMTLAVKDRVSFYKSKDFKNWEWVGDFGIDPEEGDT**SGVWECPAL**VT
LKDDQNNQHDVLLVSENGEAKGSLFQYFIGKFNGSRFNSYNTSKMYWAEDAIDNYAAVPYHNDPLGRVILIGWMSNW
LYADKIPTSTWRGQYTIPRELGIKFVGGDLYLTQRPVEELNKQISQTWSMPAPFNINANQINLTEQIPFKTGSMLKL
EYAIDIANLTTGKCGLQFSNNRGEFVSFAFDVEKNVYEFNRMHSGNVSFHERFANKVMTSDRVSTSDTLFGTIVLDV
ASIEIFADDGVNLITAIFFPTEVFENIELISTNNDASGQSPRVQSLSVSSLKSIWSN
>Cnas_GH32_8, XP_031630288
MSKLIVRLLLCCVFILATSINGQSYHEKYRLQLHFSMPS**GWANDPNGL**IYSDEYYHLFYQHDPSGALNKLLHWGHAR
SRDLIHWENLPVALYPYEKGDIFSGCCVIDKDNVTGLISAENQDQSNNTIVAVYTLHKDIEQSQALAYSFDDGITWT
QYDANPVLPNPGI**PDFRDPNIF**ERDGRFYMALAVLDRISFYTSSNLLEWTRLGDFGLEPNEGDK**SGVWECPSV**VRLK
DEQNNEHDILIVSENGDTRGSLTQYFVGTFNGTHFNSYDQSKILWVDNGFDNYAAIPYQNDPSGRVLIIGWMSNWLY
AQYTPTTTWRGQMTIPRELALQTIDGNIHLVQRPIDELMNLVDATRKWSLSTPLTLSGNQIVDLTPQIPFKTGSMFT
LEYVFNIENVVNGKVGLRFSNFFGEFITFFFDLSEGIYYLDRSNSGDVSFSPRFANRMADAKRIRSTNSLSGQVILD
TASIEIFADDGLNTFSAIFFPSELFENIQLLCAIEDNEKPITVEKLSVAALHSIWAES
>Cnas_GH32_9, XP_031628779
MAKAIISLLIGCTIFLLPINGQNYNEKYRLQVHYSVPS**GWMNDPNGL**IYEDGYYHLFYQYNPASTTFGPPHWGHAQS
LDLVHWENLPIAIYPYNEQWIFSGCCVLDKDNVTGFGSHLPGQSEQPLIAIYTLHEQHGETHKETQGLSYSLDKGMT
WTYYDANPIIPSLGI**PDFRDPNVF**ERNGTFYMVLAEFDRIEFFKSKNLINWEPISKFGVDPMEGSK**DGVWECPSL**FS
LKDEQNNEHDVLVVSENIPNSAIEYFIGKFDGVKFNTYNKSRSLRLDFGHDNFAAVPYVNDPFGRIIMVSWMSNWNY
SLDTPTSTWRGQMTIPRELKLKTVDGSLYLVQRLVDDLYSLIDGSRQWSLSTPYHLLGNQNINLSSLMPFKTGSMLI
LEYAINIENIRNGEIGIKFGNNLGESVLFHYNVSSDTYVFDRRKSGNVTFNTKFADQIAKAKRISTSKCLSGQILLD
TASIEVFADDGLSVFTSLFFPTEPYENIELIFDTADKSATVEKLSVSALHSIWT
>Cnas_GH32_10, XP_031622607

MSKIICSLLFCSIFLLSINGQNYTEKYRLQVHYSIQS**AWMNDPNGL**IYADGYYHLFFQYNPAETTFGMIYWGHARSI
DLIHWENLPIAIYPYEKGMIFSGCCVIDKNNVTGFGSHLPGQTEQPLIAIYSLHNATSNSQDQGLSYSHDRGLTWKY
YDDNPIIPNPGIS**NDFRDPNII**ERYGKFYMVLSVFEQISFYSSDNLIKWDYISQFGMDPEEGDR**DGVWECPAL**FTLK
DEQNNEHDILIVSENIPKRGSAMQYFIGLFNETHYNTYDKSRVLWLEYGFDNFAAVPYHNDPFGRLIIIGYMSNWNY
AEDIPTSTWRGQLTIPRQLGLKTIDGNLYLIQHPVDEFYSLVDRSRQWSLSTPYYIFSNEYINLTSQMTFSTGPMFT
LDYDINIENVLNGDIALKFSNNHGESVLFHYNVSSDTYVFDRQNSGNVTFNPEFINRIPKAKRINTGNRLWGHIVLD
TSAIEIFADDGILVFTALFFPTEPFENVELIFSSEDTEKTVTVEKLSVSALNSIWT
>Cnas_GH32_11, XP_031622608
MTSIIITSLIICSIFLLPINGQNYTEKYRLQLHYSVPS**GWMNDPNGL**IYADGYYNLYFQYDPNSMAEDLRPHWGHAK
STDLIHWENLPIAIYPYEKGTIYSGCCVLDENNVTGFGSHLPGQAEEPLIAIYSLHNVTTDSQTQGLSYSLDQGLTW
TYYDDNPIIPNPGL**EHFRDPNIF**KRYGKFYMILAVFDRVSFYVSENLIKWEHFSDFGEGDK**NGIWECPAL**FTLKDEQ
NNEHDVLIVSEYIYGGTPKIGGVTQYFIGQFNGTQFNTYNQSRQLWLENGQDNYAAVPYHNDPLGRNAILIGWMSNW
KYAQTTPTSTWRGQLTIPRELGLKTVDGNLYLIQHPVDEFYSLIDRSRQWSLSTPYYMFGNQSINLTSQMTFKTGSM
FTLDYDINIENIENGEIGLKFSNNLGEFVLFRYNGSSDGYVFDRQNSGNVTFSPNFANRISKAKRINTDNRLWGHIV
LDTSAIEIFADDGLNVFTALFFPTEPFENIELIFDAEDTEKTMTVGKLSVSALNSIWI
>Cnas_GH32_12, XP_031622609
MSKTAISLFIGCMIFLLPINGLNYHEKHRLQVHYSVPS**GFMNDPNGL**IFEDGYYHLYFQYDPDAMTNTNCHWGHARS
IDLIHWENLPIAIYPYEKGMIFSGCCVKDKNNVTGLGSNLPGKDVEPLIAIYSLHNVTSNSQDQGLSYSHDRGLTWT
YYDGNPIIPNPGVS**NDFRDPNII**ERYGKFYMSLAVFDRVTFYSSDNLIKWDFVSDFGVDPDEGEK**EGVWECPAL**FTL
KDEQSNEHDILIVSENVPKQGSAMQYFIGKFNGTHYNTYDKSRQLWLEYGHDNFAAVPYHNDPLGRLIIIGWMANWN
YASDVPTSTWLGQMTIPREVTLRNVDGRIHLVQRPIDEFNSIIDRSRQWSLSTPYYMFGSQNLNLTSQMTFKTGSML
TLDYDINIENVLKGDIALKFSNNLGEFVLFHYNVSSDTYVFDRRNSGNVTFSEDFITRIPTAKRISTGNRLSGQIIL
DKSAIEIFADDGIMVFTALFFPTEPFENIDFIFDVEDTEKTVTVEKLSVSALKSIWM

Sitodiplosis mosellana

>Smos_GH32_1, ON703217
MSKSIIVRFLLLFSIFYCELSVNGQSYKEKHRLQVHYSIPS**GWSNDPNGL**IYLDGYYHLYFQHNPVDTRFGPMHWGH
ARSRDLIHWETLPIALKPYEKGVIFSGCCIHDENNVTGIRPNVLYNPNASSTTLIALYTLTKDEDQSQALAYSFDNG
TTWTQYDANPVIPNPGI**SDFRDPNVF**ERNGKFYMTLAVRDRISFYVSTDLLSWEKLSDFGVSPDEGYK**IGVWECPSL**
FTLTDEEGKAHDILIVSLNGDLEGSKSQYFIGQFNGSRFNTYDKSRILWIDNGFDNYAAIPYHNDPAGRIVLIGWLS
NWLYAQDIPTSTWRGQMTIPREMGLRTIDDDLHIVQRPVVELKGIIDPARNWSTSEPLTLTGRQTIEVTSEIPFKTG
SQLILDYAFDIGQVKNGQIEVRFSNTKDEFVSFNYNIEKRTYGFDRRYSGDVTFNPRFADPLPRIDRISGSNLLSGQ
IILDTASIEIFADDGLNTISALFFPSELFEKIHLRSSIDAGKSVTVPKLSVAALNSIWPK
>Smos_GH32_2, ON703218
MSKSIIVRLLLLFSICYCELSVNGQSYKEKHRLQVHISMPS**GWSNDPNGL**IYLDGYYHLYFQHNPVDTKFGPMHWGH
ARSRDLIHWETLPIALKPYEKGVIFSGCCIHDKDNVTGIVPNVLINGDASSTALIAVYTLTKDEDQSQALAYSFDNG
TTWTQYDANPVIPNPGI**SDFRDPNVF**ERNGKFYMTLAVRDRISFYVSTDLLSWEKLTDFGVSPDEGDK**NGVWECPTL**
FTLTDEEGKPHDILIVSLNGDLEGSKSQYFIGQFNGSRFNTYDKSKILWIDNGFDNYAAIPYHNDPDGRVVLIGWLS
NWLYAQDIPTSTWRGQMTIPREMGLQTIDHDLHIVQRPVDTMYGIIDPARNWSISEPLTLTGRQTIEVTSEIPFTTG
SQLILDYAFDIGQVKNGQIEVRFSNTKDEFVSFDYNIEKRTYGFDRRYSGDVTFHPRFVDPLPRIDRISGSNLLSGQ
IILDTASIEIFADDGLNTISALFFPSELFEKIHLRSSIDDGKSVTVPNLSVGALYSIW
>Smos_GH32_3, ON703219
MFKSNIVRLFLLFSICYCELPVNGQSYMEKNRLQLHYSIPS**GWSNDPNGL**IYAGGYYQLYFQYNPVDTVFGPMHWGH
ARSPDLIHWENMPIALKPYEKGVIYSGCAILDKNNVTGFAALKNSDASSTALIALYTLDDNKNQSQALAYSFDNGTT
WTQYEANPVIPNQGI**LDFRDPNVF**ERDGKFYVTLAVLDHIEFYVSSNLLSWEKVTDFGVSPDEGLK**IGVWECPAL**LT
LADEHGNKHDILIVSMNDEVHGSRTQYFIGQFNGSRFNTYDKSRILWIDNGFDNYAAVPYPNDPAERVVLIGWLSNW
LYAQNVTASTWRGQMTIPREMGLRTIDGALHVIQQPVVELNNIIDPARNWTLSERFNLTGNHIMDVTSKIPFNTGSQ
LILNYEFDIEQVENGQIDLRFGNTKGEFVSFNYNVKERTYGFDRSNSGDVAFHPGFAGPLQRIKRISSSQLLSGKIL
LDTASIEIFADDGLNTISALFFPSELLEKIHLRSSVDAGKSVKVSKLRVAALKSIWSNNTMSSHGNNMNYSPQHIFL
LLLIIYVCSFFSFVSNNSQ
>Smos_GH32_4, ON703220

MFKSNIVRLLLLFSICYCELPVNGQSYKEKNRLQLHYSIPS**GWSNDPNGL**IYAGGYYQLYFQYNPVDTVFGPMHWGH
ARSPDLIHWENMPIALKPYEKGVIYSGCAILDKNNVTGFAALNNSDASSTALIALYTLDDNKNQSQALAYSFDNGTT
WTQYEANPVIPNQGI**LDFRDPNVF**ERDGKFYVTLAVLDHIEFYVSSNLLSWEKVTDFGVSPDEGLK**IGVWECPAL**LT
LADEHGNKHDILIVSMNDEVHGSRTQYFIGQFNGSRFNTYDKSRILWIDNGFDNYAAVPYPNDPAERVVLIGWLSNW
LYAQNVTASTWRGQMTIPREMGLRTIDGALHVIQQPVVELNNIIDPARNWTLSEPFNLTGNHIMDVTSKIPFNTGSQ
LILNYEFDIEQVENGQIDLRFGNTKGEFVSFNYNVKERTYGFDRSNSGDVAFHPGFAGPLQRIKRISSSQLLSGKIL
LDTASIEIFADDGLNTISALFFPSELLEKIHLRSSIDAGKSVKVSKLRVAALKSIWSNNTMSSHGNNMNYSPQHIFL
LLLIIYVCSFFSFVSNNSQ

Bradysia coprophila

>Bcop_GH32_1, XP_037030094
MFNRSADAIRSSTSILYLLFSLQFLHLCYTLCLDDKYRPQLHYSPSK**HWMNDPNGL**IYLDGEYHLFYQYNPNATVSG
QVYWGHAISTDLVHWTELPIALSPNDIIDVEPVSLFSGCAVLDAKNSSGLQTGSITPMILFFTQATTKKQQQSIAVS
NDRGRTFEMYSKNPVIPNDDEKV**PDFRDPKIF**NWKDKWIMMVAAGDKIQFLESDNLLNWNSISEFGARPLQGSHG**GV**
WECPDLLPFTVNGQRVYVLLVSINPGGPNQGSATQYFVGSFNGKQFLKSVLYQPLWLDWGPDNYAGVSWFNEPNNRS
LLIAWMSNWDYGKFLPTSAWRGQMTLPRVLNVQRLDGRLRLQSQPAAEVDSLRISSQFFETTQPLSIRTGHDFTNDL
GFRNSLLEVDVLIDTQLMFSDSLAYLRLCLFNDLAEEVCVGYSFGSNEIHLDRSMSGDTSFYSEFAAVATANRESKN
RYLQMKVYLDVSSIEVFVDGGFTTMTGLFFPTEAFTRVKVEFNSAKGINQLTLLSATIRGLKSIYDC
>Bcop_GH32_2, XP_037025415
MTSNLRVIHFVVTVCFIGFASSICLNDKHRPQFHFSPAK**YWINDPNGL**IYVDGEYHMFYQYYPNGTIHGTMYWGHAI
STDLLYWEELPIALSPDGLGEIFSGSAVLDVRNTSGLQTGTNLPIILIYTQEDGAAERQSIAVSNDKGRTFQKYSGN
PVLTDPSY**RDFRDPKVY**EINGKWVMSLAVKDSIDFYSSSDLIHWTALSKFGGDPIEGAH**GGVWECPDL**LSFDLDGNT
FWVLLVSINPGGPNLGSVTQYFLGDFDGKVFRKYGINQNLWMDWGPDNYAGVTFANDPLNRKVLIAWMSNWLYGEKV
PTEAWRGQMTIPRVLELKRVDKTRVRLASSPSEELKKLRSPSDFYEKTAPLIIQSGNVYDFTADLAFVNPLLEVDAL
IDTNLVTADSSASFQLCFYNRLNEQLCVGYSYGQNSIYLDRSNSGSVSFDTNFGQRATAQRETKNKIIQLKLFLDTS
AIEVFADEGVTAMTGLFYPTEPLSSIRIGFTSANSANQLSVLSITVQGLKSIYSC

Bradysia odoriphaga

>Bodo_GH32_1, KAG4069715
MLIFGLMLLSSTIFLELVSGQNYSERYRLQVHYSLPS**GWLNDPNGL**IVHGGVYHLFYQNYPYNKIWGPMHWGHAVSS
DLIHWETLPAAIKPEDDGDIFSGCCVIDNRNVTGFSKDGIEPLVALFTLSKNGHQSQAMAYSYDNGLTFTHYDWNPI
IENPSI**PDFRDPNVI**ELDGIYYMALAANDRIIFYSSKDLLSWTKLSEFGASPSQGDK**SGVWECPAI**VTLTDENGTEY
HVLIVSENGDAAGSVIQYFIGRFDGNQFVNQNDAATTLWLDHGPDNYAAVPFHNDPHGRVILIGWLSNWLYAELIPT
SNWRGQMTIPRELGLKTVNGNVHVVQQPIDDLQSIADTSKIWKIAESFSVGTTFTYNLSKESSIESSLLLLEYVFDI
QNAQSGTVEFRFSNSYNEFVSFAYVAQENIYQLDRTKSGQTSFSHRFANEKRSFDRIDNSNNITGRIILDVASIEIF
TDDGLNTFSALFFPTEPFNEVTINFAFDGNDGTNAVVINELSLTPLKSIWSSSTAIRASAGTIFIFSILYFLSQIEI
>Bodo_GH32_2, XP_037032900
MLIFGLMLLSSTIFLELVSGENYSERYRLQVHYSLPS**GWLNDPNGL**IFHGGVYHLFYQNYPYNKLWGPMHWGHAVST
DLIHWETLPVAIKPEDDGDIFSGCCVVDNKNVTGFSKNGIEPLVALFTLSKNGYQSQAMAYSYDNGVSFTRYDWNPV
IENPSL**VDFRDPNVI**ERNGIYYMAVAANDRIIFYSSTDLLSWTKLSEFGASPSQGDK**SGVWECPAI**VALTDENGTEY
HVLIVSENGDAAGSVMQYFIGRFDGSYFVNLNGAATTLWLDHGPDNYAAVPFHNDPHGRVILIGWMSNWLYAQEIPT
STWRGQMTIPRALGLKTVNGNVYVVQQPIDDLLSIADTSKNWKIAESISVGTSFTYNLSKESSIESSLLLLEYVFDI
ENAQSGIVEFRFSNSHNEFVSFAYIAQEKVYQLDRTKSGKTSFSNRFANEKLTFDRIDNSDNITGRIILDVASIEIF
ADDGLNTFSALYFPTEPFNEVTINFAFDGDDETNSVVINELSLTPLKSIWSSSTAVRASAGTIFVFSFFLRSFMN
>Bodo_GH32_3, ON703213
MPSNLHVLIYFVVTVFSIGSVSSICLNDKHRPQLHFSPSK**YWINDPNGL**IYVDGEYHMFYQYYPNGTIHGTMYWGHA
ISTDLLHWEELPIALSPDGLGEIFSGSAVLDVRNTSGLQTGTNLPMILIYTQENGAAQRQSIAFSNDNGRSFQKYSG
NPVLTNPSY**RDFRDPKVY**EINGKWVMSLAVNDSIDFYSSSDLKQWTALSKFGGNPLEGAH**GGVWECPDL**LSFDLDGN
TFWVLLVSINPGGPNLGSVTQYFLGDFDGKVFRKYGINQNLWMDWGPDNYAGVTFANDPKNRKILIAWMSNWLYGEK
VPTEAWRGQMTIPRVLELKRVDKTRVRLASSPAEELKQLRSPSEFYEKTAPLNIQSGNVYDFTADLAFVNPLLEVDA

LIDTNLVTADPSASFQLCFYNRLNEQLCVGYTYGQNSIYLDRCNSGSVSFDTNFGQRATAQRETKNKIIQLKLFLDT
SAIEVFADEGVTSMTGLFYPTEPLSSIRIGFTSANSANQLSVLSITVQGLKSIYSC
>Bodo_GH32_4, ON703214
MTSTTTSDAQALMRETALSDELRPAYHFVAPA**GWLNDPNGV**CQRDGVYHLFYQYNPTGPCHHSIQWGHATSTDLMQW
TDRPIALRPSDGHDEEGCWSGVLVDDGTRPVLVYSGHRDDRQTAMLAYGDESLDHWTKEPSNPVIAAPPAGTDI**TAF**
RDHCVWREGGVWRQIIGSGIRGRGGAAFLYESDDLVHWRELGPLVVGSADDFSETNPLWT**ATMWECVDF**FRLDADGR
TAAPDCSSGDAHVLIFSAWDDGRTMHTISARGHYAGDRFEVTDYHRLDLGGRHAYAPQSFIDEQGRRMLWAWMQEGR
DDAAQLRHGWSGAMTLPRVLRLGAAGDVRQTPAPEVEAARGEALEWHGLASRAGAAEFGFEVDLPVGSVVEVDLFAT
PDPTSTDAEFTTLRLSGVADGSIAVELDRSRSSLDTTNASSAHTGTVVRTDGRVEVRGFLDRSSIELFIDGVPLTTR
VYPTRPDADAIRIRAVDTTIEHLCGWMMRNTEQAHRVLDLTETSTG
>Bodo_GH32_5, ON703215
MNDPNGLVFHEGRYHLFYQCNPEGWRHAHLSWGHASSADLVQWEHHAVAIRDDEDGEIYSGSTVIDVHNSSGLGTAD
AAPLVAVYTQASPRRQRQSIASSVDGGRTWRKYDGNPVLDRGT**SDFRDPKVF**RYTGAAGSYWVMVAVEAQDRQVLLH
RSDDLVDWEFLSSFGPEGAV**GGVWECPDL**FPLAVDGDADDVRWVLLISLNPGGIAGGSGTQYVIGDFDGVRFVPDPD
PTDHEVDWLDFGRDCYAGVTFDGLAQEHRTLIAWMSNWDYSNGMQGGEGSLGWGMMTLPRTLSLVRLGDRIRLRQIP
VPPALAAASTVTSTVITEPARIGALPEGGCVRLRVDVDNAAGFALRLRADETGNGGVELAYDREDGVVTLDRRNGAD
ALPTGFGSVQRMPVERGAVVEWEIWLDRASIELFADAGTRTLTDLIPEQQGDQFWIEGRGGAVRIERLEIAAVTGR

Bactrocera tryoni

>Btry_GH32_1, ON703198 ((**D in active site region 1 substituted to S, E in active site region 3 substituted to A, but there is an adjacent E**)
MHKKDHKMTDETLLTPEEKAHIAVLSEAIAEIAEVNRLQKPNSTSMCPRYHLVAPC**GWMGSAPSV**VWFNGQYHAYYS
FSPFALTAGDLYIGHSVSNDLLRWEHKDPILAPEAKFDNHGCRGPFAVVFNNMVFLYYTGMIELEHKGETHYLENVC
LASSGDGETFQKLGAVISPFGGT**TYIGDPIVW**KEGGFWYMTLAIQRQDQVGDHVRSSVALYRSYTLYEWDYMTTLLT
EKKADA**EQWTAPQIF**SVGDATFMALTQLTPLETTVYPDTKHITPDGQESVIPGDTVTALPPHENLHRTVTFAGEISL
TDGFKRTGDAVKLDIGSDMLLPRIFDAPDGRKILLGWANMWGDAQPTIFFGFAGSWTLPREVELVNGQLHQKPIVEL
EQYRVTGTMYDALTLTDSHTALTDDNPSFDFELVFDLDATSAETFGFSIGAGYKLEFEVSENNELELTVFSQRNYYQ
EGLDDTRALTITRGSQLKIYGVFDRSLVELFIDGLAMPLTARIMPGISDRRIYLYSMGGTLQLASAHHNALSSF

Phormia regina

>Preg_GH32_1, ON703196
MELQSILYRPAIHYTPAL**NFMNDPNGL**IYEPQSETYHMYYQYNPYENVAGNQHWGHTTSKDXXLLHWVDQPVALSPQ
NSTDHAFTGSVVIDQNNTSKLFPGKNGTNFVAIYTQSPGNGVQQQSVMYSTDGGKSYQPYSENPVLNLNS**TNFRDPQ**
VQWLPEHEKWLMSVVLAREHKVQFYESDNLLEWTYLSDFQAGIF**GVDYECPNL**VPVKDEDGNDKYALFVSINPGMPQ
GGSGTQYFIGDFNGTHYIADDYATRIIDFGKDNYALQYVNPGSNPNELSKVPQASYIGWFSNWQYCQEVPSGDWRSA
MTLARFLSIKKDWYGVPRVAQTPVDLTHIRMNNVTTSHNFTINGTTNNYNSAVKLSNASTAYEIEVKGKMTSKVGRF
NLALXALTNFNSESLEIGLYISSQGGQVFIDRGSLKGFKSPFFTNDFSSVTDIGDDNSFDWRIIVDASLLEFYSDSG
LTVGSVQYFPESPLTHLSVSAQNGTTADFTATIHPLKKTVDRETYN

Beetles (Coleoptera, Insecta, Arthropoda)

Ignelater luminosus

>Ilum_GH32_1, KAF2893415

MLLLKMFSISIITFCLLCLLFISKTSSASIDSDLTVDKANEYIQQHKDTIDQTYRPKYHFAAPI**GWINDPNGV**IFFR
GEYHLFYQYNPYNTYSQRIHWGHAKSKDLIHWEHLPVALAPDQYYDWDGVFSGSAIVKDDKLYLIYTGHTWQNEVQC
VAVSEDGIHFEKVPQNPVLDANDLPPNAQR**QDFRDPKVI**QRGDTYYLVVASKTNSNTGQLLLYQSSDLIDWQFKSIL
LEGSREE**GEMWECPDL**FELEGKDVLIMSPISYPRSGNDYYNSHSVLEFVGEIDWEQGKLKVESLKEIDHGMDYYATQ
TILGDQNRRIAIAWMSMWGRNYPTDTLGHKWAGSMTLPRELHLKDNKLIQVPISVSAISQSPIEINNVVLNNEAKQF
EGVAGEVALLELVVNLEEARLLKLHLRKNSKEETIVSFDSNSKEVTLDRSNSGISIIGSENPPFSSRKVEVSLKQNK
LSLQIFLDQSSIEVFVNGGEETLTATVYPTELPSNGILFEAEGKVVIESLSFALLNP
>Ilum_GH32_2, KAF2899194 (**active site region 1 not detected**)
MHWGHAKSKDLIHWEHLPVALAPDKYYDRNGVFSGSAIVKDDKLYLVYTGNTWVNQVQCVAISEDGIHFEKISQNPV
LDANDLPPNAKP**QEFRDPKVF**RYGDSYYLVVASKTHSNTGQILLYQSSDLIDWRFKSILLEGTTEQ**GIMWECPDL**FE
LEGKDVLIMSPINYPKSGNHYHNGQSVLGFIGKVDWEQGKLIVENWKEIDHGMDYYATQTLLDNQNRRIAIAWMSIW
GRNYPTDTLKHGWTGSMTLPRQLHLKDNKLIQVPVSVSEISQEPIVLENIVLNDEAKQFEGVVGDVALLELVINLEE
ARLLKLHLRTNSKQKTIVSYNTISKEVKLDRTESGIPIIGSENPPFSSRKVEVPLEQNKLNLQIFLDQSSIEVFVNG
GTDTLTATIYPTQVPSNGILFEAEGKVIVESLCFALLKP

Limonius californicus

>Lcal_GH32_1, ON703131
MAESSLFKTPCYLFLFLIIGCSVNASSETKSNGTQKPIFPKKAVIPSKEEWYPTYHLSPPS**GWMNDPNGL**IWFDGYY
HAFYQHNPDAAAFGLMCWGHARSKDMITWEHLPVALRPSIEADSNGCFSGSAVDNNGTLTLIYTGNGFDRQTQCLAS
SQDTINFTNNSKVLDAPGNN**PDFRDPKVW**RESNTWYMVAGIRVDDGGQVLLYKSDDLHDWQSQGVLAKGDDSM**GFMW**
ECPDFFLLENKHILLMSPQGIQAQGYNNRNLFQSGYYLGTWQPGGQFNIEQDFIEIDNGHDFYATQTFLAPDGRRVM
IAWASMWESPLPEREQEWAGMLSLPRELTLQGNGRLRVRPIAEIENYRLFQTQINNRVLEREYVELENTTKAMEIIT
VWDLKQSDAEKYGLRLGDGNLAEAGVYLYVDTQAQRLVLDRRYPQYGISGYRSVALPSENLLTLRVFFDSSSLEVFV
NDGEASLTSRIYPLENQRHLALFAENGRATLQNSSRWWLGRE
>Lcal_GH32_2, ON703132
MAESSLFKTPCYLFLFLIIGCSVNASSETKSNGTQKPIFPKKAVIPSKEEWYPTYHLSPPS**GWMNDPNGL**IWFDGYY
HAFYQHNPDAAAFGLMCWGHARSKDMITWEHLPVALRPSIEADSNGCFSGSAVDNNGTLTLIYTGNGFDRQTQCLAS
SQDTINFTNNSKVLDAPGNN**PDFRDPKVW**RESNTWYMVAGIRVDDGGQVLLYKSDDLHDWQSQGVLAKGDDSM**GFMW**
ECPDFFLLENKHILLMSPQGIQAQGYNNRNLFQSGYYLGTWQPGGQFNIEQDFIEIDNGHDFYATQTFLAPDGRRIM
IAWASMWESPLPEREQKWAGMLSLPRELTLQGNGRVRVRPIAEIENYRLFQTQINNRVLEREYVELENTTKAMEIIT
VWDLKQSDAEKYGLRLGDENLAEAGVYLYVDTQAQRLVLDRRYPQYGISGYRSVALPYENLLTLRVFFDSSSLEVFV
NDGEASLTSRIYPLENQRHLALFAENGRATLQNSSRWRLGRE
>Lcal_GH32_3, ON703133
MAESSLFKTPCYLFLFLIIGCSVNASSETKSNGIQKPIFPKKAVIPSKEEWYPTYHLSPPF**GWMNDPNGL**IWFDGYY
HAFYQHNPDAAAFGLMCWGHARSKDMITWEHLPVALRPSIEADSNGCFSGSAVDNNGTLTLIYTGNGFDRQTQCLAS
SQDTINFTNNSKVLDAPGNN**PDFRDPKVW**REDNKWYMVVGIRVDDGGQVLLYQSDDLHDWQSQGVLAKGDDSM**GFMW**
ECPDFFLLENKHILLMSPQGIQAQGYNNRNLFQSGYYLGTWQPGGQFNIEQDFIEIDNGHDFYATQTFLATDGRRIM
IAWASMWESPIPEQEQEWAGMLSLPRELTLQENGRIRVRPVGEIENYRLFQTQINNRVLEREYVELENTTKAMEIIT
VWDLKQSDAERYGLRLGDANLAEAGVYLYVDTQAQRLVLDRRYPQYGISGYRSVALPSENLLTLRVFFDSSSLEVFV
NDGEASLTSRIYPLENQRHLALFAENGRATLQYSSRWWLGRE
>Lcal_GH32_4, ON703134
MAESSLFKTPCYLFLFLIIGCSVNASSETESNGIHKPIFPKKAVIPSKEEWYPTYHLSPPS**GWMNDPNGL**IWFDGYY
HAFYQHNPDAPEFGLMCWGHARSKDMITWEHLPVALRPSIEADSNGCFSGSAVDNNGTLTLVYTGNGFDRQTQCLAS
SQDTINFTNISKVLDAPGNN**PDFRDPKVW**REGNTWYMVVGIRVDDGGQVLLYKSDDLHDWQSEGVLAKGDDSM**GFMW**
ECPDFFLLENKHILLMSPQGIQAQGYNNRNLFQSGYYLGTWQPGGQFNIEQDFIEIDNGHDFYATQTFLAPDGRRIM
IAWASMWESPLPEREQEWAGMLSLPRELTLQGNGRIRIRPIAEIENYRLFQTQINNRVLEREYVELENTTKAMEIIT
VWDLKQSDAEKYGLRLGDGNLAEAGVYLYVDTQAQRLVLDRRYPQYGISGYRSVALPSENLLTLRVFFDSSSLEVFV
NDGEASLTSRIYPLENQRHLALFAENGRATLQNSSRWWLGRE

Sitophilus oryzae

>Sory_GH32_1, XP_030753212
MRKSLVVLIMILEPLCYGANTYDWFPTFHLAPPK**GWMSDPNGL**QYFNGYYHFYFQHNPNSPVWGSMHWGHSKSTNML
KWEHLPFALEPSLPEDIDGIFSGSAVINDGNLTVMYTGASGNLTHQTQCLARSSDGLIFEKLGVVLKQDGND**SNFRD**
PRVWRQGNSWFVVIGSITENNRGEVLLYQSDDLISWEYHGVLADANETL**GYMWECPDF**FALGDKQILLINPQGMEQN
GYDFQNLHQTGYFVGTWQPREEYIIEKGFREIDHGHDFYASQTFLAPDGRRILVSWMGMWESPFPEQGDGWAGMLTL
PRELTLSEDGTIKIKPIKEVKDYRTEDVSYENSVNLNDNSIYVLKEQVSSNEIIIELDPVKSNASTYGVRLGDEGQG
VSVYVDATKERLFLERSYPQFNISRSDRSVAVDLSIPIYLDIFIDNSSIEVFVNDGQSVLTSRIYPTELQRTLVVFH
SEGCVTLNKYSIWNI
>Sory_GH32_2, XP_030753449
MNSLMLIIVILLPLCFGADNYSWYPTFHFSPPK**GWMNDPNGL**SYYNGYYQAFYQYNPYDTESGWMHWGHARSTNLLK
WEHLPIALTPSLPEDIDGIFSGSAVVSDGNLILMYTGVSGNSTHQAQCLANSTDGINFDKLGLVLKKNGSD**LNFRDP**
KVWKQGDSWFVIIGSKTDDDRGEVLLYQSLDLKSWDYQGVLAQADETL**GYMWECPDF**FTLGDKQILLINPQGMEQNG
YDYQNLYQTGYFVGTWQPGEQYIIEKGFKEIDHGHDFYASQTFLAPDGRRILIGWLDMWESVFPERQEGWAGMLTLP
RELTLSLNGTIEIRPIREVEDYRTEDVVSNNSVTLNDDSIFVLKEQVTSNEIIIELDPVKSNASTYGVRLGDEDQGV
FVYVDTEKQRLFVERSYPQFNISRSDRSVAVDLSNPIYLDIFVDSSSIEVFVNDGESVLSSRIYPTELQRSLAVFHS
EGSVTVNKYSIWNI

Agrilus planipennis

>Apla_GH32_1, XP_018321007
MSLLKGKDLLLTILLTIIFHYLRVVNCISVTNERYRLNYHIMAPA**GWINDPNGF**SYYNGEYHLFYQHNPDNATSDNM
HWGHVKSEDLVHWKHLPIALSPDKLYDDGGVFSGSGIVXLVLMYTGNVNHSNGTYFSSQSQELALSSDGESFIKYTN
NPVIAFPPINGG**NAFRDPKIW**QYNDTWYVILGNQNNLTQQGRALIYSSYDLYIWNYVGVLAEPTGNFG**YMWECPDF**F
ELNGRHILLMSPQGIEGNGDDYKNLYQTGYFVGSYNYQTNNFSHNNVFIEMDHGHDFYAAQTMEAPDGQRIMIAWMD
MWESAYLEQEDGWVGALTIPRALTLSPSGKILQNPVDELRSLRTSTLLRSTINVEGVSKLLDARSDEILIKATFKST
VARVGIQIDIGNCSNVLAYLDKSDNKFILDRGDNDPRKTVIDVVNRDNFETLKIRIFLDKSSIEIFLDDGGTTFSSR
IYPSSTPAVYALSEGAVTQMEVVAYELKNIWKTSS
>Apla_GH32_2, XP_018320176
MRLIQFQFILIFISLKKNCFGNEEIVTLANDENLKYRLHYHIMASS**GWMNDPNGF**SFFNGDYHIFYQHNPYEVTPGN
VHWGHVKSKDLVHWKYLPNALTPGEPYDRNGCFSGTAIVDGNNLILFYTGNVDENNSTNYSSQNQNMALSGDGVKFE
KFSRNPIIPFPPEDGG**SAFRDPKVW**KHEDNWYLLMGNQAKENGRVVLYESKDLKNWNYIGVLASSWNGSL**GYMWECP**
DFFKLNESYVLIFSPQGLERDGDKYANLYQTGYVVGGYCYEYNHFSYGHFTEMDFGHDFYAMQTMETPDKRRVGVAW
MGMWESNFPESEEGWAGALTIPRELYLEEGKLIQKPIRETKLLRKSLLFSKLITVNGMEEICELKAAEIDIDTKLCA
DIDEFGFVLDDQEGLVVKFYLNETDKTFNLDRGDEDPRKTGFEELLYLKMQIFLDASSLEVFLNDGEITFTSRIYPN
QSPLLSIFSRGSCALDVDVKIFELENIWEKCN
>Apla_GH32_3, AIR93898
MSLVTFKVNKPFSIIILVIIFNLFETVSCISVTNERYRLNYHIMSPA**GWINDPNGF**SYYNGEYHVFYQYNPDDATHA
NMHWGHVKSEDLVHWKHLPIALYQDQPYDVDGVYSGSGIVVNESLVLMYTGNVNQIDGRDFSTQSQALAISSDGESF
IKYINNPVIPFPPQNGG**NDFRDPKIW**KYNDTLYVVLGNQNNLTMQGRAVIYSSYDLFDWTYVGVLAESTGSF**GYMWE**
CPDFFELNGFHILLLSPQGIEANGDDYQNIYQTGYFVGSYNYRTNNFSHSNTFIELDHGHDFYAAQTMETPDGRRIM
IAWMDMWERDYPEQEDGWVGALTIPRELTLSSSGRILQNPVQELIALRTSTLLNTTLNVEGISKLFDVHSAEILIKA
TFNDAITQVGIRIELGNNSCVLAYLDTSNNKFVLDRGDNDPRRTLINVTNANNPEALEMRVFLDKSSIEIFLGDGEI
TFSSRIYPSSTPAVYALSNGTVTQMEVVAYELENIWSGSWCIRQSSFVQAFYFAAYLMAKLYIQ
>Apla_GH32_4, XP_025830341
MSLLKEKDLLLIILLTIIFHYLRAVNCISVTNERYRLNYHIMAPA**GWINDPNGF**SYYHGEYHLFYQHNPDNATPGNI
HWGHVKSEDLVHWKHLPIALSPDKLYDNGGVFSGSGIVFKNSLVLMYTGNANQSNGTYFSSQSQELAFSSDGERFIK
YTNNPVIAFPPIDGG**NAFRDPSIW**QYKDMWYVILGNQNNLTQQGRALIYSSYNLHTWNYVGVLAESTGNF**GYMWECP**
DFFELKGRYILLMSPQGIKANGDDYKNLYQTGYFVGSYNYQTNNFSHNNVFIEMDHGHDFYAAQTMEAPDGQRIMIA
WMDMWESAYLEQEDGWVGALTIPRALTLSPSGKILQNPVDELRSLRTSTLLRSTINVEGVSKLLDARSDEILIKATF

KSTVARVGIQIDIGNCSNVLAYLDKSDNKFILDRGDNDPRKTVIDVVNRDNFETLKIRIFLDKSSIEIFLDDGGTTF
SSRIYPSSTPAVYALSEGAVTQMEVVAYELKNIWKTSS

Sphenophorus levis

>Slev_GH32_1, AIL92341
MNKIPILICLLIPIHCIGENISWYPKFHLAPPK**GWMNDPNGL**SFFDGYYHAFYQHYPDKPEWGLMHWGHARSINMLD
WEHLPIALSPSIPEDIDGIFSGSAVVNDGNLTLMYTGVSGNSTHQAQCIAYGSDGINFKKVGVVLKKDGND**LNFRDP**
KLWKQDDSWFVVVGSKTYNNRGEVLLYSSPDLYSWDYQGVLAQADKNL**GYMWECPDF**FTLNGKQILIINPQGISAKG
YDYLNLYQTGYFVGAWEPGKEYQIEKGFREIDHGHDFYASQTFLAPDGRRILIAWLDMWESEFPEQSEAWAGMFTLP
RELTLSECGDLQIRPIREVQDKRRAIPINHSPLFVDNKFIYITLKDAPGHEVYLEFDKHHKNATVYGAKMGDADHGF
NVYYDVRAGRLFLERSYVNFNISKSSRNLKIDLKQPISLDIFVDSSSVEVFVNNGFGVLSSRIYPTDKDRELVVYSD
SKLLKLNTYKIWRICE

Listronotus oregonensis

>Lore_GH32_1, ON703127 (**D in active site region 1 substituted to S, but there is an adjacent D. D in active site 2 substituted to Q, but there is an adjacent D**)
MREFSDDYYSRIEAVDQAELFKRMEAVSDSPWRQGYHVQPVM**GFMASPAA**FFFHDGVYHLFYNWHPLSSKNNINYWY
HTTSFDLVTFQNKGLKLRPDDLYDAHGLTSGSAVRFKDALYVFYTGFFKKGTDSIAPVQLAAELKPDDKLYKHSVPL
IETVPED**IKSIGQPFV**FKLNDTYYMFLGIEKDNGYGGIAVYEAVEDYRFEYTGILETNLDTFGDIWEYPGLFSLDGF
DILMFSPKGIDKFGYNFWNTYQSGFVIGELNIETLSMAHGTFFEFDYGFDFYAPQITVDKDGRRILTGLLGMHDTGY
PADEYHWVNCMSLPRVLTIENYKLKQRPHPNLTALRGEEIKAEGYFNHRPKRMRDFYGDRYEFIIDFIEYDATEIYL
KLRVSKREETVITYNTEQQEITLDTAFSGAQPEGVDGTKRYLKLNHELRQLRIFMDISSIEIFINDGDRVMSSRIFP
GSHATGVELS**TEMGECFV**EMRQYKLKETEDEKVIYSWRGTD
>Lore_GH32_2, ON703128
MTYYEKHRPRIHYTPKK**NWMNDPNGL**VYFKGEYHLFYQYNPEANHHDNMHWGHAVSTDLYHWEERSVKLYPDELGTI
FSGSVVVDDKNTSGLFNTDEGGLVAVFTHDGASQQQSIAYSGDRGRTWIKYKGNPVIPNTTI**IDFRDPKV**FWHKESS
QWVMSLACGSCIRFYGSNNLIEWQFLSDFGSSYPSY**EGVWECPDL**IQLEVEDTGETEWVLIISINEGAPNGGSGIFY
FTGQFDGKSFHPNESETDALKWADTGRDFYAAVSFDNTGTDTYWIGWMNNWQYAGHVPVSPWRSAMSLARQLSLVNT
GGSYLLRQIPVINDDNEKNTEQNMTVTPEQPLSLHTGDAFLFDIRLKGNKDQSVWGVDFITNQDEVYRLSIISRENR
YTFDRTTGSISFSEQFPRVIEGPLNGSGHALTTVVDVSSLELFFQSGLSVSTNLIFPEGQVRCINVWNDSGVLPVES
LSMKPLRTVMAAGVSSDTHDTET
>Lore_GH32_3, ON703129
MGQVMEWTRSLSYKPYHEWSKSYHSELFSVIQNSKWKLGFHILPET**GLLNDPNGF**SYYNGKWHLFYQAYPFGPVHGV
KSWYHMTSENLVDWKQNDYVVLPDSSYDSHGAYSGSALAVDDKLFIMYTGNVRNSDWERHSYQLGAWMNHEMKLEKF
DKPLISNPPEGYT**HEFRDPQVF**RYQDKYLMVIGGQKNSIEGAVLTYQSEDLTNWDYLGELDYTSDKM**GFMVECPNL**I
FIDQKPIFLFCPQGLDENIKFYQNIYPNTYVTGSSFNKETLKIEGVSELNHLDDGFDLYATQAFNAPDGRALSVGWV
GMPEVNYPSFDEKWAHCLSLVKELSIKDNHLYQNPVNEMKSLRKYSRKIEGTASPERSLIYQADENIYELELTLTTA
ESGMLKLFADDSGIDGLKIIFDAETRTLTMDRSEAGLAFAEEYGLTRSVVLPKDETLNLQFFVDRSVCELFINDGYK
VMTGRVFPKSSADTNIYIEGFDGDFTGEIFKLREMNT
>Lore_GH32_4, ON703130
MRIITIAFTVLGITFSLSQTTIAASEHQWVPKYHFAPEK**GWINDPNGL**SYYKGYYHIFYQYNPNDAVWGLMHWGHAR
SKDMLSWEHLPIALAPSLPEDIDGIFSGSAVNNDGNLSLIYTGVSGTHQVQCLATSSDGEFFVKEGVVLRKDGDD**AD**
FRDPKAWYQNGSWYVIIGSKTTDNKGEALLFKSPNLHTWESQGVFAQADDKL**GYMWECPDV**FMLGNKHILIVNPQGM
QQDGYDYQNLYQTGYFVGTWVPGHKYKIEKEFREIDHGPDFYASQTFLAPDGRRIQIAWMAMWESVFPEAASGWSGM
FTLPRELTLSSSGDLEVRPIREIKDRRMGNPKKVTAFVVNNTYAIVDEGVYSKEIIIDFNLPDTTAEKFGLRIGDSA
VSDAGVQVYVNYTESRLFLERYYPDYNIIASSRSVPINLNKTLSLDIFVDSSSIEVFVNDGQAVLSSRIYPNDDERF
LSLFSLSGLFIFSNYSSWDL

Anoplophora glabripennis

>Agla_GH32_1, XP_023309906
MVFIGIPILFTLCLYIQKSACDVDPNLTVENANAFIAENKDTINQTYRLKYHAMAPI**GWINDPNGF**IFFQDEYHLFY
QYNPYNAYPNKIHWGHMKSKDLVHWEDLPVALAPDQDYDADGVFSGSAIVKDGKLYVMYTGNSGDRQVQCIAVSEDG
INFQKIDQNPVLDANSLPSNAQP**QDFRDPKV**FQRGDLYYVVTVSRTVNETGQVLLYQSTDLINWEFKSILLEGTTEQ
GNMWECPDLFELDGKDVLLLSAIEMARSGNDYSNMDSVVEFIGEMNWGEGKFVVESMKELDHGMDFYATQSTTDNQG
RRIVIAWMNMWARTYPTADLGHGWVGAMTLPRELHIKHGSLVQKPISEIARFYKPVAKYVNIRLTNQTRRFRNVRGV
VGEMELQVNLRNTKTFTIELRANDDEKTILSYNTESSELTLDRTNSGITITGNENPQVFARKVQASLNHNRLKLQVF
LDRSSVEVFVNDGRESLTATVYPTEVPSDSIRFTAEGTALIERLTFSKINLH
>Agla_GH32_2, XP_023309905
MDKLELIVTEEDEDITRVVNMVFIGIPILFTLCLYIQKSACDVDPNLTVENANAFIAENKDTINQTYRLKYHAMAPI
GWINDPNGFIFFQDEYHLFYQYNPYNAYPNKIHWGHMKSKDLVHWEDLPVALAPDQDYDADGVFSGSAIVKDGKLYV
MYTGNSGDRQVQCIAVSEDGINFQKIDQNPVLDANSLPSNAQP**QDFRDPKV**FQRGDLYYVVTVSRTVNETGQVLLYQ
STDLINWEFKSILLEGTTEQ**GNMWECPDL**FELDGKDVLLLSAIEMARSGNDYSNMDSVVEFVGEMNWDEGKFIVESM
KELDHGMDFYATQSTTDNQGRRIVIAWMNMWGRTYPTADLGHGWVGAMTLPRELHIKHGSLVQKPISEIALFCKPVA
NYANVILTNQTHRFQNVSGEVGEMELQVNLRNTKTFTIELRANDVEKTILSYNTESSELTLDRTNSGITITGNENPQ
VFARKVQASLNDNRLKLQVFLDRSSVEVFVNDGRESLTATVYPTEVPSDSIRFTAEGTAKEMK
>Agla_GH32_3, XP_018563655
MVFIGIPILLTLCLYIQKSACDVDPNLTVENANAFIAENKDTINQTYRLKYHAMAPI**GWINDPNGF**IFFQDEYHLFY
QYNPYNAYPNKIHWGHMKSKDLIHWEDLPVALAPDQDYDSDGVFSGSAIVKDGKLYAMYTGNSGDRQVQCIAVSEDG
ISFQKIDQNPVLDANSLPSNAKP**QDFRDPKV**FQRGDLYYVVTVSKTVDETGQVLLYQSTDLINWEFKSILLEGTKEQ
GIMWECPDLFELDGKDVLLLSIIQIARSGNDYSNIDSVVEFIGEMNWDEGKFAVESIKELDHGMDLYATQTATDDKG
RRIVTAWMNMWGRTYPTADLGDGWVGAMILPRELHIEDGFLVQSPVSEIASFYEQVAEYTNVTLTDQTSRFHLVSGE
VGEMELQANLENTNTFTIELRANDDEKTILSYDTESSELTLDRTNSGISITGNENPQVFARKVLAPLNDNRLKLQVF
LDQSSVEVFVNDGRESLTANIYPTKVPSDSIRFTAEGTALIERLTFSNINLD

Dendroctonus ponderosae

>Dpon_GH32_1, XP_019772489
MLRPVLYAVFISSAIALPETGWYPKYHIAPQH**GWMNDPNGL**IYFQGFYHVFWQYNPAAPQWGLMHWGHARSPDLLTW
EHLPIALAPSLPEDIDGAFSGSAVLLNETLTLIYTGVSENGTRQVQMVATSQDGVAFEKLGVVIGENE**TNFRDPKAW**
WQDGSWYVVIGAQTADERGQVSLYSSPDFFNWTPQGVLAQADPSL**GYMWECPDF**FSLEGKQVLVVNPQGIQSKGEDF
QNLFQTGYFVGSWAPGGNFAVERGFRELDHGHDFYASQTFDSPDGRRLEIGWLGMWESQFPENASGWAGMLSLPREL
TLSDQGDLEVRPLREIQSLRTERLEVPQTLHITPGEGVAILQNISHSEMALDFNLRNSSSNAFAIQLTKESFERDGG
AQVHVDRNCSRVFLERHYPAYNITRSSRSVAVNLTGNLSLDIFIDGFSMEVFVNDGQAVMSSRIYPDAELRTFLITA
QNGSVAVDRLKVWDLYLSIINAI
>Dpon_GH32_2, XP_019766056
MLRPVLYAVFISSAIALPETGWYPKYHIAPQH**GWMNDPNGL**IYFQGFYHVFWQYNPAAPQWGLMHWGHARSPDLLTW
EHLPIALAPSLPGDIDGAFSGSAVLLNETLTLIYTGVSENGTRQVQMVATSQDGVAFEKLGVVIGGNE**TNFRDPKAW**
WQDGSWYVVIGAQTADERGQVSLYSSPDFFNWTPQGVLAQADPSL**GYMWECPDF**FSLEGKQVLVVNPQGIQSKGEDF
QNLFQTGYFVGSWAPGGNFAVERGFRELDHGHDFYASQTFEAPDGRRLEIGWLGMWESQFPENASGWAGMLSLPREL
TLSDQGDLEVRPLREIQSLRTERLEVPQTLHIAPGGGVAILQNISHSEVALDFKLHNSSSNSFAIQLTKESFERDGG
AQVHVDRNCSRVFLERHYPAYNITRSSRSVAVNLTGNLSLDIFIDGSSMEVFVNDGQAVMSSRIYPDAELRTFLITA
QNGSVAVDRLKVWDL
>Dpon_GH32_3, AEE61467
MLRPVLYAVFISSAIALPETGWYPKYHIAPQH**GWMNDPNGL**IYFQGFYHVFWQYNPAAPQWGLMHWGHARSPDLLTW
EHLPIALAPSLPGDIDGAFSGSAVLLNETLTLIYTGVSENGTRQVQMVATSQDGVAFEKLGVVIGGNE**SNFRDPKAW**
WQDGSWYVVIGAQTADERGQVSLYSSPDFFNWTPQGVLAQADPSL**GYMWECPDF**FSLEGKQVLVVNPQGIQSKGEDF
QNLFQTGYFVGSWAPGGNFAVERGFRELDHGHDFYASQTFEAPDGRRLEIGWLGMWESQFPENASGWAGMLSLPREL
TLSDQGDLEVRPLREIQSLRTERLEVPQTLHIAPGGGVAILQNISHSEVALDFKLHNSSSNSFAIQLTKESFERDGG

AQVHVDRNCSRVFLERHYPAYNITRSSRSVAVNLTGNLSLDIFIDGSSMEVFVNDGQAVMSSRIYPDAELRTFLITA
QNGSVAVDRLKVWDL

Rhynchophorus ferrugineus

>Rfer_GH32_1, KAF7287881
MSKLLLTIAALLPILCGADHGWYPVYHVAPPQ**GWMNDPNGL**SYFDGYYHAFYQHYPYAPEWGLMHWGHARSKNMLNW
EHLPIALTPSLPEDIDGIFSGSGVVNEGNLTLIYTGVSGNSTHQEQCLAYSSDGVNFTKEGVVLKKGGND**ENFRDPK
LW**WQDDSWFVVIGSKTDDNRGEVLLYRSPDLKDWSYEGVLASADDKL**GYMWECPDF**FTLNNKQILLINPQGVEQDGY
DYQNLYQTGYFVGSWAPGGNYTVEEGFKEIDHGHDFYASQTFLAPDGRRILIGWLAMWESEFPEGADGWAAMLTLPR
ELTLSDSNVIEVRPIREVQKYREEISSNSDPITISGGAPYGLLADAKANEVTVRFDTASTTAKTYGLFFGNEAGSLN
ISVNVDLGRLFVTRYYPNYNITESERSVAVDLSGPLNLDVFLDSSSIEIFVNDGAGVLSSRIYPVDGQRDLVAYQTD
GVAVLSQYKSPILRKVEVPIVENKKCEEMLRKTRLGDDFNLHESFICAGGEAGRDTCHGDGGSPLLCSNDNYNTMIQ
VGVTSWGIDCGQKDVPAAYADVRHSYNWLITELNKRNINITARVN

Cockroaches (Blattodea, Insecta, Arthropoda)

Blattella germanica

>Bger_GH32_1, PSN47193
MFLHKVFALTLVMVCSISADQVPRAAPPAYTVEYANQYIEENKDTVIQTYRLNYHFMAPI**GWINDPNGF**IYYQGEYH
LFYQFNPYDTFSQKIHWGHAKTKDLIHWEHLPVALAPDEWYDADGVFSGSAIEKDGKLYVMYTGNSAEGQVQCIAVS
EDGIHFEKVPENPVLDANDLPSNAQA**PDFRDPKVF**KRGDLYYVVLVTKTTDNRGEVLLYQSADLINWEFKSILLEGT
SEQ**GIMWECPDL**YQLDGKDVLVLSPIQIPRVGNEYWNIDSVVEFVGTMDWESGKMAVETVKELDHGMDFYATQSLED
QQGRRIVIAWMAMWGRNFPTDTLGHHWAGSMTLPRELHVKDGLLTQVPVAEAFNITQNPVEYFGVYLTDEVREFNQV
SGETGLLNIVVNLNVASVFTIDLRANENEKTVLTYNTESQELILDRTSSGITIIGGENPTVYSRIVNVPLVRSRLIL
QIFLDVSSVEVFVNEGVETMTSTIYPTEATSSAIRFGVQGTAAIDSLSFSSISV
>Bger_GH32_2, ON703221
MTFWRKSAIVLVAACLAAQQVSCDSSALTVQSANAFIKQNEGRVDTTYRLKYHFMAPY**GWINDPNGF**IYFRDEYHLF
YQYNPYNSTPNKIHWGHAKSKDLIKWEHLPVALAPDQAYDQDGVFSGSSIEKDGKLYLMYTGYANNDQVQCIAVSED
GVNFNKVSQNPVIDSSALPPNAVA**GEFRDPKVF**KRGNMYYVVLVSETKNQTGQVLLYESKDLINWNFKSILLEGNPG
Q**GVMWECPDL**YKLQGRDVLVLSPIQWPKTGNDYENTDSVVEFIGHMDWNKGTLSVENYKELDHGLDFYATQSLLDNK
NRRVFIAWMAMWGRNFPTSDLHQGWAGAMTLPRELHLKNGKLVQKPVDEIKNYYGNPVVLKQHTIENEVKSFKHVSG
EVAVLELTVDLRQARHFWIDVRANKHNKTVLSYDTSTQEVKLDRTNSGISMTGREDPPVFFRKVKAPLFHTGLLHLQ
IFLDVCSVEVFINKGEESMTDTIYPEGGNASEIRFGAEGAGVIKELYFAPIVF

>Bger_GH32_3, ON703222
MTFWRKSAIVLVAACLAAQQVSCDSSALTVQSANAFIKQNEGRVDTTYRLKYHFMAPY**GWINDPNGF**IYFRDEYHLF
YQYNPYNSTPNKIHWGHAKSKDLIKWEHLPVALAPDQAYDQDGVFSGSSIEKDGKLYLMYTGYANNDQVQCIAVSED
GVNFNKVSQNPVIDSSALPPNAVA**GEFRDPKVF**KRGNMYYVVLVSETKNQTGQVLLYESKDLINWNFKSILLEGNPG
Q**GVMWECPDL**YKLQGRDVLVLSPIQWPKTGNDYENTDSVVEFIGHMDWNKGTLSVENYKELDHGLDFYATQSLLDNK
NRRVFIAWMAMWGRNFPTSDLHQGWAGAMTLPRELHLKNGKLVQKPVDEIKNYYGNPVVLKQHTIENEVKSFKHVSG
EVAVLELTVDLRQARHFWIDVRANKHNKTVLSYDTSTQEVKLDRTNSGISMTGREDPPVFFRKVKAPLFHTGLLHLQ
IFLDVCSVEVFINKGEESMTDTIYPEGGNASEIRFGAEGAGVIKELYFAPIVF

Spring Tails (Collembola, Collembola, Arthropoda)

Cyphoderus albinus

>Calb_GH32_1, ON703109
MMIGVRVEMCHLFFVFSLFTSASTFNVNQEKKEPLALIKQSTSCTTQTYRPRYHFSPSE**NWMNDPNGL**VYYDGLYHL
FFQYHPGSSLWGPMNWGHAISRNLVTWEELDIALMPDELGDIFSGSAVVDHLDRAGFKDQNSTVDPIVAIYTSASGE
SYEIQRQSLAYSIDNGITFQKYEHNPILEAENS**SDFRDPKVF**FHSASSKWIMSLAVGHKIEFYSSTNLLNWTKESEF
GADPLQGEH**GGVWECPDL**IQLRAQVNSSTIIDIWVLLVSINPGYFIGNFSANRDGTLSFTAFEWNNAQWLDWGPDNY
AGVTFSNEPSGRQIYIAWMSNWNYANKTPTRPWRGQMTIPRQLGVRVLNSSSGDLRLTSNPVPELTSLRHPLLQILN
RIEFEMLPQTITVLTNDAKFKSPLMELEIDLEIVNNPQFSICAHNTLKEETCFGYNGTHWYIDRSKSGNFHKVNKLY
AASAQAFATRETTDTEVTIRIFFDVSSIEVFADGGLTSMTALHFPTEPFDKLYIEHWSKGATNASLRVKRFKLWALR
CWYSGSASTKPTPVFTLVTVFIVLLCVLPQ
>Calb_GH32_2, ON703110
MKFEVFCCALLASWANCSPLLAKATNVQCTSESHRPRYHFSPRE**KWLNDPNGM**VYYEGIYHLFFQYHPFSSEWGPMH
WGHAISTDMIHWKELDIALYPDELGFIFSGSAVVDFKNTTGFQPSNSDVPPIVAVFTHAGGENNVIQRQSLAYSLDK
AMTFAKYDGNPVLEVPTE**PDFRDPKVH**WNEEGQRWIMALAVRNRIEFYSSTDLKSWVKDSEFGANPQEGAH**GGVWEC**
PDVFPLKVTNSAGEEIELWALIVSINPGGPNRGSIAQYFIGNFKKNERDQMVFETFPWKDTQWLDWGPDNYAGVTFS
NQPNNRFSYIGWMNNWIYANNLPTPDWRGQMTIPRELGLYVLNEGERQYRLTSNPIPEMVILRKLNELVENTHSFDL
PPQTVVNLMENATFRTLLLELEITIEIENNPRVSICAFNEATREESCFGYNETKWVLDRSKSGNVGFHGEYAATLVG
TAAREVQDQQTTINMVRV
>Calb_GH32_3, ON703111
MLRPRPFHSFILVAFFFGVALAQDEQRDCTTQKWRPRFHYSSLV**NWINDPNGM**VYNDGVYHLYYQYHPNSTIWGPMH
WGHATSTDLVSWVERGIALAPDANGMIFSGSSVVDPTNTTGFKQGDLDPIIAIYTSHKEKTTPTGKNIETQSIAYSL
DNGLTFQMYNSNPVLKDHNTTR**EDFRDPNVL**RHQGMWVMSLAAQDRIEFFSSTNLKSWKFLSAFGEKEGSH**GGVWEC**
PDLIPLRVVISDKKVIEKWLLLVSINPGGPNGGSATQYFIGDFNDATGEFKTDPWSDTQWLDWGPDNYAGVTFANEP
TGRAVYTGWMSNWNYANVTPTEGWRGAMTIPRLIELVYLPNPDPLVGDFRVKTTPIPELKNLRVPGEEFKSDTFDLP
IQSKRNINLPFRNPLMEVEVKISNIDVDNAQFTICAANSRFEEACFGFDRNEWILDRQASGTVKYTKANTGVLSLEA
RAKREINAEKTTIRLFFDTSSVEAFADDGLTAMTAIVFPTVPYDRLYINHWAQEN

Pogonognathellus flavescens

>Pfla_GH32_1, ON703112
MANNFWTLLFTVLVLSSYVECGEKACLNDRYRPQFHFSQAA**NWINDPNGM**VYYKGEYHLFYQYHPYSTVWGPMHWGH
AVSSDLVHWEELPIALYPDELGQIFSGSAVVDSRNTSGLQQSVDEDVIVAIFTHEGGSQVQSLAYSNDRGRNFQKFV
RNPVLPNPPGV**IDFRDPKVI**DINGKWVMALAVGNKISFYGCEDLISWSPMSEFGADPPQGGH**GGVWECPDL**FPLEFD
GQTVWVLLVSINPGGPNLGSVTQYFLGDFDGLTFRSFYPGEEPVLWMDWGPDNYAGVTFSNEPNNKRILMGWMQNWI
YSADIPTAAWRGQMTIPRELVIKMVEGRLRLASVPVPEMSLLRNPDEIYSPPNIGVISSGSILVLTSGMPFTNHLLE
LDLTLQFDGFAAFGVCFYNGVAQELCFGYEHANSQFFLDRSRSGNVNFHPDFSRRAIAARESRSRTITFKVFLDDSS
IEIFADGGTTVMTALFYPDQPFTVAHIHHYEGESVLEIQSATLRGLHSIHDCN
>Pfla_GH32_2, ON703114
MSWKSRVVLCMVVMNILTVKCEETRISGAALQKGGIKDETFRPQLHYSVRE**NWSNDPNGM**VYLDGEYHLYYQYHPYD
TVWGPMHWGHAVSPDMVSWTDLPIALYPDELGTIYSGSAVPDFENTAGFQTGNNTPIIAIFTHAGASQQQSIAFSND
KGRTYTKFEGNPVISNTDI**QDFRDPKVI**RYRDSWVLVLAAGNRVVFYGSQDLKTWEHLSEFGNDPSQGAH**GGTWECP**
LIMETTIANETVWVLIVSIGGGGPNGGSVTQYFIGDFDGRTFHSSQVDPLWMDWGLDNYASMSFSNDPQNRNIVIGW
MSNLQYSGSTPSVGWRGQFTLPRVLEVKLASGQLRLQSTPVPELNILSSFYIIHMDTRVVGADEVIDLKSEDFLRNP
LMHAVLHFDTENMSVGASLSICFLNSLRQEICTGFDKGRERSIFLNRELSGRSDFHPEFARRASALRQISSPIIQFE
IYLDVSAIEVFVDSGLTCMTALFYPDEPFQTVEIRHHANGNPSSTFTILPNGQVEGLKSIYEI
>Pfla_GH32_3, ON703115
MSWKYGVVVCTVVMCILTAKSEGRISGAGLQNGGIKDETYRPQLHYSVRE**NWLNDPNGM**VYLDGEYHLYYQYHPYGT
GDGPKHWGHAVSADMVSWTDLPIALYPDELGAIWSGSAVPDFENTAGFQTGNNTPIIAIFTHAGASQQQSIAFSNDK
GRTYTKFEGNPVIPNTVL**QDFRDPKVV**RYNRDSWVLILAAGNRVIFYGSQDLKTWEHLSEFGNDPPQGSH**GGTWECP**
IFMETTIANETVWVVIVSVSGGSPNGGSGTQYFIGDFDGRTFHSSQVDPLWMDWGLDNYASMSFANDPHNRNIVIGW
MTNLQYAGSTPSVGWRGQMTLPRVLEVKLASGQLRLASTPVPELNILSSFYIIHMDTRVVGADEVIDLKAEDFLQNP

LMHAVLNFDTENMAVGASISICFLNSLRQEICTGFDKGRERSIFLNRELSGRSDFHPEFARRASALRQISSPLIKFE
IYVDVAAIEVFVDSGLTVMTALFYPDEPFQQVEIRHHANGNPSSTFTILPNGQVEGLKSIYEV
>Pfla_GH32_4, ON703116
MANNFWTLLFTVLVLSSYVECGEKACLNDRYRPQFHFSQAA**NWINDPNGM**VYYKGEYHLFYQYHPYSTVWGPMHWGH
AVSSDLVHWEELPIALYPDELGQIFSGSAVVDSRNTSGLQQSVDEDVIVAIFTHEGGSQVQSLAYSNDRGRNFQKFV
RNPVLPNPPGV**IDFRDPKVI**DINGKWVMALAVGNKISFYGCEDLISWSPMSEFGADPPQGGH**GGVWECPDL**FPLEFD
GQTVWVLLVSINPGGPNLGSVTQYFLGDFDGLTFRSFYPGEEPVLWMDWGPDNYAGVTFSNEPNNKRILMGWMQNWI
YSADIPTAAWRGQMTIPRELVIKMVEGRLRLASVPVPEMSLLRNPDEIYSPPNIGVISSGSILVLTSGMPFTNHLLE
LDLTLQFDGFAAFGVCFYNGVAQELCFGYEHANSQFFLDRSRSGNVNFHPDFSRRAIAARESRSRTITFKVFLDDSS
IEIFADGGTTVMTALFYPDQPFTVAHIHHYEGESVLEIQSATLRGLHSIHDCN

Yoshiicerus persimilis

>Yper_GH32_1, ON703117
MDKMKSLFAAFLVLNIVVLEGTSLPHPKNERFRPQIHFSVPE**NWSNDPNGM**VYLDGEYHLYYQYNPFDTVGNGNLMH
WGHAVSLNMVKWSNLPVALSPDELGAIWSGSSVVDFDNSTGFQSGSTPPIIAMYTSFGDTQQQSIAFSNDRGRTFTK
YEGNPVIPNPNRI**PDYRDPKVI**RYQDSWVMVLAVGPKVEFYGSADLKTWEYLSEFGADPSQGAH**AGPWECPDF**LEMK
FGDETVWVLTVSLFGAHPNGGSGTQYFIGDFDGRRYTSSQFDPLWMDWGLDNYASVTFSNDPRGRWIMMGWMTNLEY
ASQTPTEGWRGQFTLPRILGLQLANGQLRLTSTFVPELSVLLKDGLYHPENITIAADEVLDMSSVNNPLLHLDLTFD
TSGMANGAAIAICFVNSIGQELCTEKQYDIEIYLDVSAIEVFVDSGLTAMTGLFYPDEPYTGVEVRHHADGHPESNL
ILLNGFVEGLLSMNEI
>Yper_GH32_2, ON703118
MKYAYTIFLLVLLSQNSLIESRKKACLNDQHRPQIHFSPAN**NWINDPNGM**VYYGGEYHLFYQYYPYGTQWGPMHWGH
TISSDLVNWEELPIALYPDELGDIFSGSAVVDQRNSSGLQESVDEDVIVAIFTHAGASQQQSIAFSNNRARNFSKFA
GNPVLPNPGL**PDFRDPKVI**EYNDKWIMVLAVGNRIIFYQSDDLKSWGILSEFGADPPQGSH**GGVWECPDL**LPIDYNG
ETIWVLLVSINPGGPNGGSSTQYFIGDFDGTTFRNFNPGENTVLWMDWGPDNYAGVTFANEESNRHVMMAWMNNWQY
GNDLPTVAWRGQMTLPKELIVKHVEGRLRLASVPVRELSHLRNPGQVYVPPSVGVISSGSIASLTAGAPFQTPLMEV
ELNLDFDGFVSFGVCFYNSLGQELCFGYEHANHQFFLDRTRSGNVAFHPEFSKRATAFRESQSKSMDLRIYLDVSAI
EIIVDGGLTTMTSLFYPEEPFTFAHIHHYSGESETRLELKSATVQGLNSIYDC

Tomocerus vulgaris

>Tvul_GH32_1, ON703119
MNMKIVALVLVLISQSYLIEGRKKACLDDKHRPQIHFSQER**NWINDPNGM**VYYKGEYHLYYQYHPGSTVWGPMHWGQ
ALSTDLVHWEEQPIALYPDEHGAIFSGSAVVDQRNSSGLQLSNDEDVIVAIFTHAGAVQAQSIAFSNDRGRNYTKFA
GNPVLPNPGV**PDYRDPKV**VEVDGKWVMALAVGNKIMFYGSDNLKSWRVLSEFGADPPQGSH**GGVWECPDL**LRMDFDG
ETLWVLLVSINPGGPNGGSVTQYFIGDFDGTTFRSFYPGENALWMDWGPDNYAGVTFSNVAENRHLLMGWMNNWDYA
NEVPTVAWRGQMTLARELHLKHVEGKLRLASVPASEVSLLRNPDQVYSPPNIGLISSGSILLLTSGSPFQNPLMEIE
LSLQFDGFVSFGICLFNTLAQELCFGYEHANNQFFLDRTKSGNVSFHPQFSRRALAARESRSKSMNLRIFVDVSAVE
IFADDGLTLFTSLVYPDEPFTFAHIHHYDSGEAETRLELKTAKIQGLRSIHDC
>Tvul_GH32_2, ON703120
MGKTPFIALLFLINFLVREGVSLSNQGRKIQLKNERFRPQIHYSVPE**NWSNDPNGM**VYLDGEYHLYYQYNPYDTVGD
GNRMHWGHAVSTNMVKWTDLPVALYPDELGAIWSGSAVVDHDNTTGFQTGDTPPIVAIFTHFGPLQQQSIAYSNDKG
RTFTKYAGNPVIPNTDGL**PDFRDPKVI**RYGNSWIMVLAVGPVVHFYSSTDLKSWEFLSEFGREQGAH**AGPWECPDL**L
EMKFGDETVWVLTVSLHGAHPNGGSGTQYFVGDFDGLHYSSSQMDALWMDWGLDNYAGVTFANDPKGKWIMMGWMTN
LDYAGSTPTEGWRGQFTLPRQLGLRLVNGQLRLQSTPVPELNLLLKNGLYNPENITILADDVLDLSTLNNPLLHLDL
TFDISRLSNGASLAICFVNSQSQEVCTGYDKGRERPLFLNRELSGLADFHPNFPRRASAIREATTDTIRFEIYVDVS
AVEVFVDSGLECMTALFYPDEPDTGVEIRHHAVGNDDSVIVLLNGYVEGLLSMTEI

Tomocerus qinae

>Tqin_GH32_1, ON703121
MKMKYAVLIFALIFQSSLTESRKKACLNDKYRPQIHFSQER**NWINDPNGM**VYYKGEYHLYYQYHPGSTQWGPMHWGQ
ALTTDLIHWEEYPIALYPDELGTIFSGSAVVDQRNSSGLQQNSNEDVIVAIFTHAGSAQAQSLAFSNDRGRNYTKFP
GNPVLPNPGI**PDFRDPKVV**DINEKWVMSLAVGNKINFYESDDLKSWRLLSEFGADPPQGSH**GGVWECPDL**LRIDFNG
ETLWVLLVSINPGGPNGGSVTQYFIGDFDGTTFRSFYPGENALWMDWGPDNYAGVSFSNAPDNRVLLMGWMNNWNYG
NELPTVAWRGQMTLPRQLVLRHVEGRLRLASVPAVELSQLRNPEQVYSPPNIGVISSGGIFLLTSGSPFQNPLMEID
LTVEFEGYVSFGICFFNSLAQELCFGYEHANNQFFLDRTKSGNVGFHPDFSKRALATRESRSKTINLKIFLDVSAVE
IFADDGLTVFTSLFYPEEPFTFAHIHHYIGEAETRLELKSAKLQGLRSIYDC
>Tqin_GH32_2, ON703122
MGKMSFLSLFLVVSVLLREGTSLARPNIPKDDRFRPQIHYSVPA**NWANDPNGM**VYLDGEYHLYYQYNPFSSVADGNT
MHWGHAVSTNLVKWTDLPTALSPDHLGAIWSGSAVVDYENTAGFQTGATPPIVAIFTHFGPLQQQSLAYSNDKGRTF
TKYEGNPVIPNPVNSFSVMLLIGT**PDFRDPKVI**RYGDSWIMVLAAGPKVDFFRSRDLKSWEFLSDFGSDPPQGAH**GG**
PWECPDFLEMKVGDDTVWVLIVSLDGGPNGHSATQYFVGDFDGTTFSSSQMDPLWMDWGLDNYASVSFSNEPRGRWV
MMGWMANWEYAGSTPTEGWRGQFTLPRMLGLELANGQLRLQSTPVPELNILLKNGLYHPENITIAADEVFDMSTLNN
PLLHIDLTFDIHEMKNGASIAICLVNSLGQELCTGLDKGRERPIFLNRELSGLGDFHPAFARRASAAREVSRETIRF
EIYVDVSAIEVFVDSGLTCMTGLFYPGEPYTGVEIRHHASGNPDSSIVLSTGFVEGLLSMGEI

Orchesella cincta

>Ocin_GH32_1, ON703123
MAFGTMGEVSRTSNDTLYKIKRSIASHSKRDKLSRRVLRSMPPVRMALGSVSYFDELFAASNIDFVIRQTSLKFIAI
GLVALVAIHVTESKSVFHTEQNQCTTEQYRLRYHFSPAE**HWINDPNGM**VYYDGVYHLFFQYHPYSTVWGPMHWGHAI
SRDLATWEELPVALAPDEIGDIFSGSSVVDFTNSSGFQTSPDIAPIVAIYTSAGGAGQRQSIAYSNDKATTFTKFEG
NPVLSDANS**PDFRDPKVR**KLSNGKWAMALAVRNKIEFYGSDDLKSWNKLSEFGADPEQGSH**GGVWECPDL**FPLNVTQ
VDGTTLEMWVLIVSINPGGPNRGSAAHYFVGTFDGTFTALPWQNTQWLDWGPDNYAAVTFSNEPQDRFIAMGWMNNW
MYANNLPTAAWRGQMTIPRTLGLRDLNKSENRYRLTSVPTAELEALRNPQEYVGNNAEFMVPPQTVIQLTEQATFKN
PAMELEITLEISQQPRFSLCAHNSGGEEICFGLNETRWYQDRSKTGNTGFNGEYAATLYASAPREIDDQEMKIRIFL
DVSSFEVFADDGLTSMTSLFYTNEPLDMFYVNHWSDTGSSATVKVKNFKVWGLVCWFQERAQKLKLS
>Ocin_GH32_2, ON703124
MIMNKLQLCVAIFLVLHLDAVASMPKQQTQCTTEEFRPKFHFSPAQ**HWINDPNGL**IYYDGVYHMLYQYHPNSTIWGP
MHWGHAISTNLITWEELDIALAPDQLGDIFSGSSVVDSTNTTGLKTDNGNDPIVAIYTSASGDNFLTQVQSLAYSLD
KGASFQKYENNPVLKDENS**PNFRDPKVF**YHGGKWIMSLAVGNKIEFYGSPDLKNWTKLSEFGADPLVGNH**DGVWECP**
DLIPLVVDVSGNGTSIVELWVLLVSINPGGPNTGSATQYFVGNFDGTTFTTYPWSDSQWMDWGPDNYAGVTFSNEPS
GRALVISWMSNWNYANATPSTAWRGQMTIPRTLDLIVLNRTADQYRLVSTPAEELVVLRNPLQYLAYNAELNVLPQS
VINLTEQAAFSNPLMELEITLEISKNPEPSFAICASNSKGEEVCFGYNNTEYYLDRSKTNNTAFNQHYTGALRSKAV
RQITDETITVRMFLDASSIEVFADGGLTTMTAIHFPSEPLDKLYINHWSKAGSPATLKVKEYKVYGLHCWFAEKEIP
ATTEALNSGASQNQYKSKNFKVWFIFCRQILQQ

Sinella Curviseta

>Scur_GH32_1, ON703105
MTLCTRLLAASLFVAIISDLTLANPAFSKQNEQCTSDKYRPRYHFSPAA**NWINDPNGM**IYYDGLYHLFYQYHPYSMQ
WGPMHWGHAVSKNLVTWTELDIALEPDDIGDVFSGSAVVDTLNTTGFKTNDSDPIVAIYTSAAGNNYETQRQSLAYS
LDKGMTFKKFEKNPVLESTS**KDFRDPKVF**FHDGRWIMSLAAGNKIEFYSSPNLKDWKKESEFG**TSEGEHGSA**TQYFI
GDFSKNDSNPIAYKTNTWKKSQWLDWGPDNYAGVTFSNEPGGRFIYVGWMNNWLYANFTPTSTWRGQMTIPRQLSLR
VLNHEEKQYRLVSTPIKELETLRNHLQYIEQNQEIDLGKASTVNLTEKADFATPSMEVDVTLEIENDPHFVICASNS
VNEEVCFGLNKTSWYLDRSKSGNTGFHSQYATTLHATAEREFTDKQTSIKLFLDASSMEVFADGGVTTMTALHYPSQ
PLDKVYMKYLSPANDKSSVKIKNFKVWGLQCWFTEPAPKSGARISNSANVGLALIFTLALASIF
>Scur_GH32_2, ON703106
MILNSKAVFSALCVLLTFTSNGEGRPSSSSRALQCSTEQYRPSYHFSPAT**NWLNDPNGM**VWYDGVYHLFFQYHPYST
IWGPMHWGHAISSNMVTWEELPIALYPDEFGDIFSGSAVVDFTNTSGFQPPQGGDPPIVAIYTSAAGPVQRQSIAYS

LDKGMTFQKYEHNPVLADETR**PDFRDPKVA**LIDGRWIMSLAVGDKIEFFSSTNLIDWTLDSEFGANPPEGNH**GGVWE**
CPDLFSFDVQDPDGSTRTLWVLIVSINPGGPNRGSASQYFVGNFTRNGDTYSFSTYPWSSNQWLDWGPDNYAGVTFS
NEPQGRHIYMGWMNNWIYANNLPTVTWRGQMTLPRQMNLKALDPNNVNDPRYRVTSTPVPELDSLRNPDQHLEITEP
INIEPQTVITVTDLANFSTPLMELEVTVEIEGQIQDFSICAFNSAEEESCFGYNNTRHGWYLDRSKSGNVGFHNEFQ
QTLNALATREVNTEQVVIRMFVDVSSLEVFADEGLTSMTSLHYPSQPFDKIYINNWSSGESNAVLRISKLNLWGLNC
WYEGTA
>Scur_GH32_3, ON703107
MEITTKYLLALSILCFQTKFSEAWQPDAQCSTQQHRLRYHFSPRE**NWSNDPNGM**VYYEGVYHLFFQYHPESTQWGTM
HWGHAVSSNMLNWEQVDIALYPDEHGTIYSGSSVIDFGNTTGFQVNPDVHPLVAIFTSAGSSQTQSIAFSNDNGGSF
AKFEGNPVLSGENT**PDFRDPKVF**MDANTGNWVMVLAVGNRVEFYGSENLKSWTKLSEFGRDPEEGSH**VGTWECPDM**V
ELPVGEGVEKLWALIVSVGGGGPNGGSGTQYFVGNWDGTKFTTFPWQPTQWVDWGPDNYAGVTWSNDPFNRSIGLGW
MSNLAYAGAIPTSTWRGQMTMPRQLSLRALNATANQYRVQSQPPQEMESLRNPLQHVEHSQEFTVLPQTTVTLTEQA
VFRNPSMEIEISLEMLNEPQFSLCAYNSLGEEVCFGYNATKWFLDRTKSGNTGFNDQFQRSSFATAQREIGEEQVTI
KMFLDTSSIEVFVDDGLTAMTGLFFPTEVLDQVYIHHWSGATSGATLKVKKLDIWGLVCTVQDTK

Cyphoderus albinus

>Calb_GH32_1, ON703109
MMIGVRVEMCHLFFVFSLFTSASTFNVNQEKKEPLALIKQSTSCTTQTYRPRYHFSPSE**NWMNDPNGL**VYYDGLYHL
FFQYHPGSSLWGPMNWGHAISRNLVTWEELDIALMPDELGDIFSGSAVVDHLDRAGFKDQNSTVDPIVAIYTSASGE
SYEIQRQSLAYSIDNGITFQKYEHNPILEAENS**SDFRDPKVF**FHSASSKWIMSLAVGHKIEFYSSTNLLNWTKESEF
GADPLQGEH**GGVWECPDL**IQLRAQVNSSTIIDIWVLLVSINPGYFIGNFSANRDGTLSFTAFEWNNAQWLDWGPDNY
AGVTFSNEPSGRQIYIAWMSNWNYANKTPTRPWRGQMTIPRQLGVRVLNSSSGDLRLTSNPVPELTSLRHPLLQILN
RIEFEMLPQTITVLTNDAKFKSPLMELEIDLEIVNNPQFSICAHNTLKEETCFGYNGTHWYIDRSKSGNFHKVNKLY
AASAQAFATRETTDTEVTIRIFFDVSSIEVFADGGLTSMTALHFPTEPFDKLYIEHWSKGATNASLRVKRFKLWALR
CWYSGSASTKPTPVFTLVTVFIVLLCVLPQ
>Calb_GH32_2, ON703110
MKFEVFCCALLASWANCSPLLAKATNVQCTSESHRPRYHFSPRE**KWLNDPNGM**VYYEGIYHLFFQYHPFSSEWGPMH
WGHAISTDMIHWKELDIALYPDELGFIFSGSAVVDFKNTTGFQPSNSDVPPIVAVFTHAGGENNVIQRQSLAYSLDK
AMTFAKYDGNPVLEVPTE**PDFRDPKVH**WNEEGQRWIMALAVRNRIEFYSSTDLKSWVKDSEFGANPQEGAH**GGVWEC**
PDVFPLKVTNSAGEEIELWALIVSINPGGPNRGSIAQYFIGNFKKNERDQMVFETFPWKDTQWLDWGPDNYAGVTFS
NQPNNRFSYIGWMNNWIYANNLPTPDWRGQMTIPRELGLYVLNEGERQYRLTSNPIPEMVILRKLNELVENTHSFDL
PPQTVVNLMENATFRTLLLELEITIEIENNPRVSICAFNEATREESCFGYNETKWVLDRSKSGNVGFHGEYAATLVG
TAAREVQDQQTTINMVRV
>Calb_GH32_3, ON703111
MLRPRPFHSFILVAFFFGVALAQDEQRDCTTQKWRPRFHYSSLV**NWINDPNGM**VYNDGVYHLYYQYHPNSTIWGPMH
WGHATSTDLVSWVERGIALAPDANGMIFSGSSVVDPTNTTGFKQGDLDPIIAIYTSHKEKTTPTGKNIETQSIAYSL
DNGLTFQMYNSNPVLKDHNTTR**EDFRDPNVL**RHQGMWVMSLAAQDRIEFFSSTNLKSWKFLSAFGEKEGSH**GGVWEC**
PDLIPLRVVISDKKVIEKWLLLVSINPGGPNGGSATQYFIGDFNDATGEFKTDPWSDTQWLDWGPDNYAGVTFANEP
TGRAVYTGWMSNWNYANVTPTEGWRGAMTIPRLIELVYLPNPDPLVGDFRVKTTPIPELKNLRVPGEEFKSDTFDLP
IQSKRNINLPFRNPLMEVEVKISNIDVDNAQFTICAANSRFEEACFGFDRNEWILDRQASGTVKYTKANTGVLSLEA
RAKREINAEKTTIRLFFDTSSVEAFADDGLTAMTAIVFPTVPYDRLYINHWAQEN

Folsomia candida

>Fcan_GH32_1, XP_035710378
MLAKFTPIVAAILVVAVATVTHGQVDDRYRPKIHFSIAA**NWINDPNGM**VYMDGEYHLHYQYNPADSVWGPMHWGHSV
STDLVTWTDLPIAMFPDHLGQIFSGSAVVDFNNSTGFQQDDVNVPIVAIFTHAGDSQQQSIGYSLDKGRTYAKFEGN
PVIPNPGI**PDFRDPKVI**QYDDKWILLLAAGNKVMFYSSTDLKTWEYTSEFGADPEQGEH**SGVWECPDL**FPLQFGGET
VWILIVSINPGGPNGGSATQYFVGGFDGVNFNSPQMTPLWMDWGVDNYAAISFFNEPSGRAVNMGWMNNWDYANGLP
TIGWRGQMTLPRVMDLVLVNGQLRLASNPAPELRTLFKNETYQTVPNQVVEADQVLEIPTGNSSLLHTEFTFDTRTM

GIGSSLAICMTNPAGQEVCTGFDKGRERPFFLNRSDSGFVDFAPNFSRRITATRERSSTEAIKFEIYWDVTAVEVFI
DGGLTCMTALFYPDEPYTILEVRHHGAGNPGGTLVISSGSIRSLYAMDELINKK
>Fcan_GH32_2, XP_021946443
MAASLAGIKPLLNWLAYCCIALGLVTSVQGAQEWDNFRPKLHYSVQL**NWLNDPNGL**VFVNGEYHMYYQYHPGSSVPG
PKSWAHTISRDFVTWSDLPTALEPDELGDIWSGSSIVDFTNSSGFQTNVDVYPIVAVFTHAGQSQQQSIAYSNDWGR
TFTKFAQNPVIPNPGK**QDFRDPKVI**WYNDESWILVLAAGDRVEFYISQDLKNWSLSSDFGADPDQGL**KGVWECPDI**F
PLEFDGETVWVLLVSNGGGGPNGGSNTQYFVGTFDGTTFSSPQIDPLFMDSGVDNYASISFFNEPRGRRVIIGWMTN
LMYANDIPTGDWRGQMTMAREVRLAKNSGNALRLRSLVVDEIVEKLVDPNQEISVGITGIAPDTTMYLTDSVPWNNS
LMHLNLFIDVGGLCQGCSLSICFLNSKEQEICSGYDHGRERPLFLDRERSGRTDFNPSFSRRLSAIREIPLDGANNI
LQFEAFLDLVAIEVFFDGGMTPMTSLFYPEEPYTFVEIRHHALGNINSRLSIASGSVFQGMKSMYEV

Allacma fusca

>Afus_GH32_1, ON703125
MRVVLVVVLALGMVASERPLECRQERHRPQIHFSVPS**NWLNDPNGM**VYYDGEYHLHYQYHPYSTQWGPMHWGHAVST
DLVTWTDLPISLYPDELGMIFSGSAVVDLDNSTGLQKDSVQPIVAIFTHANAGPQQQSIAYSLNKARDFVKYENNPV
VPNPGV**GDFRDPKVI**RYNDKWVMVLAVGNKVDFFGSSDLKTWEFLSDFGIDPSQGSH**AGVWECPDL**VRIQHNGFYHW
VLLVSINPGGPNGGSVTQYFIGSFDGKEFHSNQMDALWMDWGEDNYAAVSFSNEPKGRTLVMGWMNNWAYANELPTR
SEGFNGQMTLPRELNLATVDGALRLISSLPEEFGKLRDPSQVFQLPLLKEIPPNGFYNVSEEIGFKSGLVEVDLAFN
MVGIEGTSSIAICFVNRQRQEVCTGYDHERPVDKELFMNRELSGDLRAIGGLRRATAGRVKKDNIITFKIVMDLSAA
EVFIDNGLTVFTALFFPDEPLDTLEIRHHAGSAESRVTLINGSVQGLRSMYNC
>Afus_GH32_2, ON703126
MKQLFLFLTLIFSIAASDVLVCREEKFRPQIHFSVPE**NWANDPNGL**VYYDGEYHFHYQYHPGNPNNGPKSWAHAVST
NLVIWTDLPVAIEPDHLGDIWSGSAVVDHENTTGFQQDPDIAPIVAIFTHSSSSQQSIAYSLNKARDYVKYEGNPV
LPNNIH**RDFRDPKVF**RYENKWVMVLAAGDRVMFYSSLDLKSWEYMSEFGADPPQGSH**SGVWECPDM**LEIKYNGFTYW
VLLVSVSGGAHNGGSFTQYYVGLFNGREFQSSQIDTKYLEWGEDNYASVSFSNEPRGRQIIIGWMTNAIYCEQLPTG
IFRGEMTLPRVLTLENVAGHLVLKTNLVEEFANLRIPSQEHPLEMRRLEPNGYWNVSEELGFKSQLVEVDLAFNIAG
MKGLSALAICFLNRLRQEICVGYDHERPENQEIFVNRELTGDLRSISDHFVGRQTGGRVFKDGIITFKIVLDTTALE
LYVDGGLTVMTSLFFPDEPMDTLEIRHHAPVDGSEVLLLNGKIQGLRSMYDC

Dicyrtomina minuta

>Dmin_GH32_1, ON703108
MYQKPLATAVLLVLGLAINSCASFSIGIPNAENAPAECRNEKHRPQLHFTPKA**NWINDPNGM**VYYDGEYHLYFQYTP
NSAHSGAKYWGHAVSTDLSTWTELDIALYPDEMGDMWSGSAVVDFKNASGFQTDPNIQPIVAFFTHAAGPAPQQQSI
AYSNDKGRTFTKFAGNPVIANPGR**GDFRDPKVI**QFAENSWVLVLATGDRVQFYTSTDLKKWDLASEFGAEPLVGNH**G**
GTWECPELLRIETQGYESWVLLVSNFGGYHNPGSGTQYFVGSFDGTTFHSDQVAPLYLDWGFDNYASVSFSNEPKNR
HILMGWMTNLMYAEEIPTGDYRGQITLPRDISLVAVDGHLRLKSVIPEEFFNSLRNPSQSFAISSMTDIPPNQQLNV
SDAMPFSNSLLEVDLAFNIHEIRGPSSIAICFLNSLNQELCVGYDHEREAGTEIFLNREKTGNLGAISGSFAGRATA
GRETGDAIITFKIILDVSAVEMFVDGGLTVMTALFYPDEPLTRVEIRHHAFHPESKVTLISGSVRGINSMYDC

Spiders/Mites (Arachnida, Arthropda)

Tetranychus urticae

>Turt_GH32_1, XP_015792351

MVNKLVYSWITLIILLSDFSLDTIQLIQGMPMQGNTESKVYGEKWRPQYHFSPPT**SWINDPNGL**TYSNGKYHLFYQY
HPDSLVWGPMHWGHATSSDLLRWENEPIALYPAPGGAAFSGSAVIDSTNITGLQKTANSNVLVAIFTRSNDSSPTPQ
TQHLAYSYDGITFNNYENNPILVDPSK**RDFRDPALL**KYGDGYIMSLAAGDAAMFYKSTNLIDWTYLSSFGEKPSQGS
H**GGVWECPAL**IRFEVDGRELWVLLISINPGGPTVGSATQYFVGTFDGTRFINENPADRVLWLDWGPDNYAAAMYTNI
PSPKPTMVAWMNNWNYAANEPTTPWRGQMTLPRSMELVKTSDNDYHVQTFPIDDVKNLRESLLYEFKGEPVTESLAI
QHNSDRYHLECSFKSNRTSFGAVKIKLMNQASSEQVTMNLDNLRIEVDRSQSGLTNFSSDFTKLIRGSRILPSPQLS
VDLFFDSSAVELFYDKGLSSFTYLVFPTQPYDLISIECTDCTISSLSMYQMKSIWKNDS

>Turt_GH32_2, XP_015792426
MVNNLVYSWITLIVLLSDCSLDTIQLIQGMPMQGNTESKVYGEKWRPQYHFSPPT**SWINDPSGL**TYSDGKYHLFYQY
HPDSLVWGPMHWGHATSSDLLRWENEPIALYPAPGGAAFSGSAVIDSTNITGLQKTANTNVFVAIFTRSNDSSPTPQ
TQHLAYSYDGITFNNYENNPILVDPSK**RDFRDPSLV**KYGDGYIMSLAAGDAAMFYKSTNLIDWTYLSSFGEKPSQGS
H**MGFWECPAL**IRFEVDNSELWVLITSINPGGLNVGSATQYFVGTFDGTRFINENPADRVLWLDYGPDYYAAAKYTNI
PSPKPTMIAWMSNWNYATNQPTTPWRGQMTLPRSMELVKTSDNDYHVQTFPIDEVKNLRESFLYEFKGEPVTESLAI
QHNSDRYHLECSFKSNRTSFGAVKIKLMNQASGEQVTMNLDNLRIEVDRSQSGLTNFSSDFTKLIRGSRILPSPQLS
VDLFFDSSTVELFYDKGLSSFTYLVFPTQPYDLISIECTDCTISSLSMYQMKSIWKNDS

Tetranychus cinnabarinus

>Tcin_GH32_1, ON703231 (1)
MVNNLVYSWITLIVLLSGCSIDTIQLIQGMPMQGNTESKVYGEKWRPQYHFSPPT**SWINDPSGL**TYSNGKYHLFYQY
HPDSLVWGPMHWGHATSSDLLRWENEPIALYPDQGGAAFSGSAVIDSTNITGLQKTANTNVFVAIFTRSNDSSPTPQ
TQHLAYSYDGITFNNYEKNPILVDPSK**RDFRDPSLV**KYGDGYIMSLAAGDAAMFYKSTNLIDWTYLSSFGEKPSQGS
H**MGFWECPAL**IRFEVEGRELWVLISSINPGGLNVGSATQYFVGTFDGTRFINENPADRVLWLDYGPDNYAAAKYTNI
PSPKPTMIAWMSNWNYATNQPTTPWRGQMTLPRSMELVKTSDNDYHVQTFPIDDVKNLRESLLYEFRGEPVTESLAI
QHNSDRYHLECSFKSNRTSLGAVKIKLMNQASGEQVTMNLDNLRIEVDRSQSGLTNFSSDFTKLIRGSRILPSPQLS
VDLFFDSSAVELFYDKGLSSFTYLVFPTQPYDLISIECTDCTISSLSMYQMKSIWKNDS
>Tcin_GH32_2, ON703231 (2)
MVNKLVYSWITLIILLSDCSLDTIQLSQGMPMQGNIESKVYGEKWRPQYHFSPPT**SWINDPNGL**TYSNGKYHLFYQY
HPDSLVWGPMHWGHATSSDLLRWENEPIALYPDQVGAAFSGSAVIDSTNITGLQKTANSNVLVAIFTRSNDSSPTPQ
TQHLAYSYDGITFNNYEKNPILVDPSK**PNFRDPSLL**KYGDGYIMSLAAGDAAMFYKSTNLIDWTYLSSFGEKPSQGS
H**GGVWECPAL**IRFEVDGRELWVLLISINPGGPTVGSATQYFVGTFDGTRFINENPADRVLWLDWGPDNYAAAMYTNI
PSPKPTMVAWMNNWNYAANEPTTPWRGQMTLPRSMELVKTSDNDYHVQTFPIDDVKNLRESLLYEFKGEPVTESLAI
QHNSDRYHLECSFKSNRTSFGAVKIKLMNQASGEQVTMNLDNLRIEVDRSQSGVANFSSDFTKLIRGSRILPSPQLS
VDLFFDSSAVELFYDKGLSSFTYLVFPTQPYDLISIECTDCTISSLSMYQMKSIWKNDS

Oedothorax gibbosus
>Ogib_GH32_1, KAG8199638
MFLLAFSLLLAANSIPGLTSRSIIAEDSNQDDYLLIKINGLVNERSIDKFLENIAADVRKFDPSLCAGPTPYTPPKS
EWYPTYHLAPPS**GWMNDPNGL**IHFGGYFHAFYQHNPYAPEPEQIHWGHARSTDMLEWEHLPPALAPSIEEDKNGCFS
GSAVNDGGTLKLMYTGNGDVQVQCLATSNDSSGIHFTKQGKVLDNPEGV**INFRDPKVL**PLNGEWFVVIGTEFQGEGQ
IRLYKSGDLQQWEEQGVLTDRQSGLG**GYMWECPDF**FPLEDKYLLMFSPQGIKPEGYLYRNLYDNGYFLGTWSPDTPF
DIEKEFTLLDHGHDFYAPQTFQAPDERRIVIAWMDMWESPMPEKKQGWAGSLTLPREMTLNDDGDLLINPIREVEGL
RTEPTTNSETILHSEKLLLSDEVYAAEVDITWNLTSSVAETYGLHLGHPNSSEGLFLYVDNQAQRLTLDRRYPQYDL
VGYRSVSLSNKDTVSLRVFYDHSSVEVFVDGGRSCMSSRIYPTMKKRQLSLYAENGDATLLSSTLWMLQK

Caerostris darwini

>Cdar_GH32_1, GIX93812
MYFRIIFSLFFICLLHCSLGAKLQSDGQHLRLKKSAVQSSDTPETLFQTQDQTSETVSAFEDPQAEDECNASVPEKE
AWYPTYHLAPPQ**GWMNDPNGL**IEFEDYFHAFFQYNPSAPFWGDIHWGHARSKNLIQWEHLPVALAPSIPEDRDGVFS

GSAVNDNGVLTAIYTGNNFLENDQLRQVTCLATSNDTINFTKLGEVLEPPNGV**QNFRDPKAW**WGDDRWYVVMGTQDG
AVGEAHLYESEDLHHWDFVEVLATGAGGL**GYMWECPDF**FPLDDKYLLVLSPQGIEPEGYKYRNIYQSGYFVGTWEHG
SAYHIEKNFTEIDNGHDFYAPQTFLTSDQRRIVIGWMSMWESPFPEKAQGWSGMLSLPRELVLAKDGNVLIEPVREI
EAFRGPENHLNKTVLEKSSHILSRNVKAMEILMSWDLSSSNAEKYGLRLGDENCSDGGLYIYIDTQAQTLTLDRRYA
RYSVSGYRSIPLLQEDQISLRVFLDHSSVEVFVDGGRNCMSSRIYPADDQRQLSIFADNGIGVLEKCSWWPIVVKEV
K

Araneus ventricosus

>Aven_GH32_1, ON703157
MLNFIQFYVLFTFFAMAVIRIAAILKPSKMADIWRLFGWLARFPSLARNRTINDSPSSTNGDWYPTYHLSPPK**GWMN
DPNGL**IEFQGYYHAFYQHNPSEPVWGDMHWGHARSKNLVEWEHLPEALAPSIDEDRDGCFSGSAVDDRGTLTLIYTG
NIFLEKDQLHQVTCLATSNDTINFAKLGKVMDPPEGV**QHFRDPKAW**RADNRWYVVMGTRDGDHGEAHLYQSDDLREW
EFVQVLAKASDGQ**GYMWECPDF**FPLGDKHLLVLSPQGMPKIGYSYRNLYQSGYFMGKWKPGDDFQIEQNFTEMDNGH
DFYAPQTFFTSDGRRIVIGWMDMWESPFPENVYGWSGMFSLPRELTFAEDGTFLIEPVREIEDFRGKEDHLNKTVLT
KSFLKLHDDAKAMEVIMTWDLKSSDAEMYGLRLGDANHTDGGLYLYVDTQARRLTLDRRYLQHSLSGYRSIPLSGAD
KLSLRVFLDHSSVEVFVDGGRKCMSSRIYPANDERELSVFANNGQAVLENCSWWPIKANSVD

>Aven_GH32_1, WEI57568

MLNFIQFYVLFTFFAMAVIRIAAILKPSKMADIWRLFGWLARFPSLARNRTINDSPSSTNGDWYPTYHLSPPK**GWMN
DPNGL**IEFQGYYHAFYQHNPSEPVWGDMHWGHARSKNLVEWEHLPEALAPSIDEDRDGCFSGSAVDDRGTLTLIYTG
NIFLEKDQLHQVTCLATSNDTINFAKLGKVMDPPEGV**QHFRDPKAW**RADNRWYVVMGTRDGDHGEAHLYQSDDLREW
EFVQVLAKASDGQ**GYMWECPDF**FPLGDKHLLVLSPQGMPKIGYSYRNLYQSGYFMGKWKPGDDFQIEQNFTEMDNGH
DFYAPQTFFTSDGRRIVIGWMDMWESPFPENVYGWSGMFSLPRELTFAEDGTFLIEPVREIEDFRGKEDHLNKTVLT
KSFLKLHDDAKAMEVIMTWDLKSSDAEMYGLRLGDANHTDGGLYLYVDTQARRLTLDRRYLQHSLSGYRSIPLSGAD
KLSLRVFLDHSSVEVFVDGGRKCMSSRIYPANDERELSVFANNGQAVLENCSWWPIKANSVD

Argiope bruennichi

>Abru_GH32_1, KAF8789872
MLIFTLLLLFFVNSLDYSYGSQKRDDFTYLRKGPFQVIEEISKNNDESKNRSTEDRDNYDSSVSTKGDWYPTYHLSP
PK**GWMNDPNGL**IEFKGYYHAFYQHNPSEPVWGDMHWGHARSKNLIEWEHLPVALGPSIDEDRDGCFSGSAVDDRGTL
TLIYTGNVFLENDQLHQVTCLATSNDTINFTKLGKVMDPPKGV**QHFRDPKAW**RADNRWYVVMGTRDGDLGEAHLYQS
EDLRQWEFVQVLAKADIGQ**GYMWECPDF**FPIDDKYLLVLSPQGMPKIGYKNRNLYQSGYFLGKWNSGDYFQIEQNFT
EMDNGHDFYAPQTFLTSDGRRIVIGWMDMWESPFPENVFGWSGMFSLPRELTLAKDGTLLIEPVREIEDFRGKEDHL
NRTVLNKSFLKIHDDATAMEIVMTWDVKSSDAETYGLRLGDANLTDGGLYLYVDTQARRLTLDRRYLQHSLSGYRSI
PLTGIDTLSLRVFLDHSSVEVFVDGGRKCMSSRIYPEKDQRELSVFANNGQAVLENCSWWPIKVDNIN

Sea spider (Pycnogonida, Chelicerata, Arthropoda)

Nymphon striatum

>Nstr_GH32_1, KAG1650202 (**active site region 1 not detected**)
MHWGHATSTDLLHWEHKPIALFPDEHGLIFSGSAVVDFENTSGFGTKENPPLVAIFTYHLMDGEKAGRKDFQTQGIA
YSLDNGDTWTKYDGNPVIGNDGI**KDFRDPKVL**WDEVNHVWVMALVAGDHLQLWNSKDLKNG**PNEVEGTDE**EKWVLLI

SINPGAPNGGSGTQYFIGDFDGEKFTTDQTETKWLDWGTDNYAGVTYNVLPKGYYGDTYKNDPKQDRIFIGWMSNWA
YARDTPTEKWRSAMTVPRKLSLKKVNDNYSLFNYPVSEMESIIDNATKKNITVSAGTKESISFVDLNQSEMLFKTAS
KNFQLKVSNSLNEEVVLTMFGDEKQFFLDRTGSGKVDFQENFSGVQQMPVPDLPADGI

Millipede (Julida, Diplopoda, Arthropoda)

Julidae sp.

>WWPL01038432
MNNLKDLRSLFGRIATYVIVGVAGLLISCKVESPSYVVTHLCDKQSKVQIGKPEKYLLLPVEESSNESHINVVVDNE
IVQSLNVRLAVRKTDYYVPLDLSGFKDKSISLSFNNIDSSAICWTSIKLSNEFDSTNREKFRPVYHFTPLY**GWMNDP**
NGMVYKDGEYHLFYQYNPYGSMWGNMHWGHAISRDLIHWEHLPVALTPDSLGAVFSGSAVVDHNNIAGFGAGAIVAF
YTSAGERQTQSMAYSLDNGRTFLKYRNNPVLTSTI**PDFRDPKVF**WHEQTQKWIMLIAAGQEMQIYSSLNLKDWQMES
RFGEGNGAH**GGVWECPDL**IELSVDNTAEKKWVLICNLNPGGIFGGSATQYFVGTFDGKTFVNESPAQTKWMDWGKDH
YATVTWSDAPDNRHIAIAWMSNWEYANQVPTTQFRSANSLPRDLSLYMRGGETYIKCTPSAELQALRTNKQEEASFK
VDNTYNQDKILAGNQGAYEMEVTITNQDAEVIGFRLFNAIGEEVNCYFNLVENKFYMDRTRSGKVDFSPSFSAVTAA
PTEQGKEYKLRIFVDKASIEVFGQDGRFVMTNLIFPTEPYDRVSFYSKGGTYNVNPLTVYTF

Crab (Decapoda, Malacostraca, Arthropoda)

Portunus trituberculatus

>Ptri_GH32_1, JACMIE010001374
MSIEKPSNYQELHRPQFHFTPAA**NWMNDPNGM**VYYEGEYHLFYQYYPDSTVWGPMHWGHAVSKDMVRWTHLPIALYP
DSLGYIFSGSAVIDWNNTSGLGKDGPPMIAIFTHHDIKGEKAGENQFQYQSIAYSNDRGRSWTKYAGNPVLPNPGI**K**
DFRDPKVIWHEASQKWIMVFAAYDKAMFYHSPDLINWAYVSEFGIPGD**TRLWECPDL**FPLKVKDSEETKWILIVSIQ
KDAPNGGTATGYFIGDFDGETFKGNNQEQHWLDYGRDNYALVSWSDIPESDGRRLGMGWMSNWEYAQVVPTEKWRSA
MTLPRELVLKNTAQGFRLTSQPVEELKRLRTAEVLIPLEATVGEISLEDKIPFPINQLELRLHWLLPEEGTTEVGIE
WSNELGEYYRFGYDGKTNTFYSDRRKSGKTEFSEEFAKQIHLAPRFTKERGLSLHFFMDAASVEVFTDDGKVVMTEI
LFPTQAYQHLKVFNTGAVARLERAQVFSLQSIW

Water flea (Decapoda, Malacostraca, Arthropoda)

Daphnia similis

>Dsim_GH32_1, WJBH01000282
MYKNIIFIAILGILASCQSSPEVVFTPSESEAYRPDYHFTPPS**GWMNDPNGL**VYLDGEYHLFYQHYPDSTVWGPMHW
GHAISKDLVHWEHYPIALEPDSLGYIFSGSAVYDAQNTSGLGTTKNPPLVAIYTYHDMAGEKSGRTDYQTQGIAFSV
DKGRTWTKYSGNPVLPNQGI**KDFRDPKVSQ**IKDADGSASWLMTLAAQDRIQFFSSKDLKKWELLSEFGQEVGAH**GGV**
WECPDLLPFKTPSGQQKYVLLVSINPGGPQKGSATQYFIGDFKNGEFVPDDTMIRWLDYGPDNYAGVTWSNVPEEQN
RTLFIGWMSNWLYSQVVPTSSWRSALTLARELSLIEVDGTLLLKSAPVSELKNLRGEEISIKNGSSNLPSQAVEITA
ELANSESFSIILTNELGEKLGIIKDQGLISIDRSLAGKHDFHEDFASVHSAPMSWEAKNVRLFLDAHSVELFVNDGE
LVMTSIVFPNRPWKKIEVSETFHLQKSIP

Wood lace (Isopoda, Malacostraca, Arthropoda)

Idotea báltica

>Ibal_GH32_1, JAACYD010068060
MKIAGYKVLLLVFLISIFSCKNQEEKVQTISNIKTSDHYAERYRPQFHFSPQE**KWMNDPNGL**VHYKNTYHLFYQYYP
DSTVWGPMHWGHAISKDMIKWEHKPVALYPDEHGLIFSGSAVIDSKNTSGFGTLENPPMVAIFTYHLMAGEKAGRTD
FQTQGIAYSLDEGNSWIKYVGNPVIGNPGI**KDFRDPKVF**WNEKLQLWSMLLVAGDHMQVWNSQDLKSWEQVSEFGKD
KGAH**CGVWECPDL**FPLKIEGTEEEKWVLLISINPGAPNGGSGTQYFIGDFDGNTFTTEQKSSRWIDLGRDNYAGVTY
NNAPNGDRIFIGWMSNWDYARDTPTVKWRSAMTLPRKLSLKKINESYELINYPLESLNAIVVTEQKPQEIIIKADEI
KTIELAYGNTSEIQFKTTARDFMLNLNNSLGDSLIFGMEGQNKEFIFDRTRSGRIDFKDNFALGIQKMPISNLPDGP
IEVRILLDHSSVEIFINKGQYVMTNQIFPNEMYKTLEIFNPADQNLEIIDFVENKVERIWD

Shrimp (Spinicaudata, Branchiopoda, Arthropoda)

Eulimnadia texana

>Etex_GH32_1, NKDA01000016
MRKVCFFLLLSLLSFCSFSQPAFQEKYRPRYHFTPEK**NWINDPNGL**IYYGGEYHMFYQYNPFGNTWGHMSWGHAVSP
DLVRWKELPVALAEEGETMIFSGSASDGSAEYVRFCEKAGASAHGSHLYRTYRGQEPEPAHRPFPGFRQNIYQVPVQ
SGPDLDL**KDFRDPKVF**WHDATKRWVMLVVVPDKKKIQLYHSKSMKAWTLLSEFGPAGDT**TGIWECPDL**FRVPVVGGA
GGYKWVMMHSPSPYMQYWVGEFDGTKFVNENRADKILPARLWPGLLCRHNYNNSLPTMGPVSIGWVNNWEYAKEIPA
GEWRGAMSLPRSLAVKKVGNEWILVQNPVDQLGTLAGPAKSFDPAGPLAIPSASFLLEWSWPDNNTGVSEMVLDNNE
LVVRFNKQTREVEIDRSGGSKVFNNAAFRRLSTYKAKVPSPANGNLKFRLYFDQSIAELYVGDGELVMTAQVFPDQV
RQVRFTNTTGQGTLREIAPYR

Water bears (Parachela, Eutardigrada, Tardigrada)

Ramazzottius varieornatus

>Rvar_GH32_1, GAV03986

MASFNSLSIVLCVSFFLQAVSSKLYSEPYRPQFHFSPKA**NWMNDPNGL**VFHNGTYHMFFQHNPTGIGFGPMHWGHAT
SQDLVHWEEQPLALFPDPLGHIFSGSAVVDINNTSGLAPAGLTAIIAIYTSHNPEFAAQNRTDVERQSIAFSVDNGK
TFVKYEKNPVLPNPGI**RDFRDPKVF**WYALQEKWVMALAAQDRIHFYSSKNLLQWEKESEFGGPSIGAH**GGVWECPDL**
FLIEQKDISKWVLLVSINPGGPNGGSATQYFTGAFDGKTFTADSKDINWVDYGTDNYAGVTWSNVGDRHLFLGWMSN
WNYGQSVPTEPWRGAMTVPRDLSLKVANENAYLVSQPIPELNSLIEAELVSLNETTVKGELKLVDTKRENSTGLLMV
NFELAEVAEWSFVLENDLGEQVVVGYEASSRQLYVDRSSSGKLDFEIQFADKMKAPRISQDGSMDVTLLIDVASIEM
FADGGLTCMTAIFFPHKPLNLLSVRSGGVGSLVLGSVRVWSLNSTVWSGQVDPVSSGLRRAPSTTNSFFIVFSLGLL
FLCVLGEL

Hypsibius dujardini

>Hduj_GH32_1, ON703162

MVTESAASIRQRLAVDPNRPRYHFLPPS**NWMNDPNGF**IQWQGTYHLFYQHNPNSPLWGDMHWGHATSDDLVHWTDWP
IALAPTPGGPDEAGCFSGSAVNNNGVPTLIYTGTRGARNEVQTQCVATSHDGLQTWQKFMGNPVLPDMPALTKQS**SD**

FRDPYVWREPDAWYMVVGSRILGVGGVVFLYRSPDLLKWEYLHPLLINDDKQQA**DGIWECPNF**FKLGDRWVLIISSH
TGTTTDTVVYHVGSFENLQFKPAYRGVLDNAQLYAPLTTRDDQNRMILIGWVRETRSRDEMLAAGWSGVQSIPRVLT
LDQHDRLCMQPVSALDGLHSAHHHLDARDVSAPTKLAVHGSVLDIQASFTPAADGSCGLAVGCSVDGSERLEIVYEA
AAQRLFVRIVSAAGIQDAANHGREVRHELAPQEPLQLRILLDGSVVEIIANERTSLTSRLYLARAASDGVQLLGHKA
RLNTLDIWEMSSIWQ

>Hduj_GH32_2, ON703165

MYCSLVNRVPSHQHRPLVHFSPSK**NWINDPNGL**VFYAGEWHLFYQHNPHTPMSVNMHWGHSVSEDLIAWTELPIAIE
PEDDVVGIWSGSVVIDWKNTTGFQKNTSVHPMIAIYTWQKQRWQEQHMAYSLDRGKVFIVIKFQTCCCLGRTWTKYS
SNPIIPMYNNISAADRLSDRIVR**RPFRDPKVS**YHIPTSSWILVLTVGNHVQFYRSSDLIHWSLMSRFGLTDGSH**GGT**
WECPDLIEFPQRTLKNRTSLWVLLVSVQSNAPAGGSGMQYFIGTFDGKEFHNIQPPETINWLDYGPDFYAGITFHNV
PGYDRRQILISWMSNWQYANSLPTAPLWRGQMTIPRQLQLDLNSYTNKYHLRQTPVQELTRYSKRLITYHHEKLSKM
ILNMSRDVYMFNIELHKITKATTIRLKFRENSDKSEYTQVSYFGEKNQIELDRSRSGLTDFHSSYPVQFNMNLDRET
LITGRLQLQLIIDRCSIELFVNEGKYTMTSLIFPKDSGYQIEISVDGHELFINYLELMLFSL

>Hduj_GH32_3, ON703166

MHSATKIGFVGLLVLLLNASASISSAQDNSWKYTQKFRPQFHFSPAE**GWIGDPDGM**IRFDNLYHVFWWGHAVSDDLV
HWKELAYPMKGDNGSFLYFSGSVVVDKQNSSGFGTGEKPPMIAIYTMHDQYTKDETQGLSISQDYKTFTYYDKNPVL
DAEQ**AAFRDPTVF**FHEPSKRWIMVITLPEERKVSFYASADLKQWTHLSDFGPMGAT**SQVWEVPDL**VEMPLDGDAKHT
KWVLFCGMGPNRVQYFVGDFDGTRFTPDPATTTGQQVHWLDYGPDYYAARTYRDYDGVEQRTVMMAWMGNWEYADKV
PTTWGKGTLALPRELTLKTSPEGVRIVQKPIPALEMLRQDEVQTKQMRLEGTMQLTAFAPRRNTYELDASFTISDPA
ARFGLRLATNGNAGIAVGYDAKTGELFIDRMHPENGDFNPNFPKLVTAPLLPNEGTIRLHIYVDQSSVEVFANEGEV
TMTALYFPDPASTGIEAFSDGGQTTLTQLSAWELESIWGVKPK

>Hduj_GH32_4, ON703168

MQTLLLLLAALGGTFANEDIPIAPFPDMNYGAWKVTGTAFTWGPANPQAQVKLEITNASSDRIISSEMEDDRPVGTMI
SPQFRIERHYISFRVGGGDYERDTCLNLIVDGKVIRMATGRRSDRLVPTSWDVRRYLGKNARLQVVDNASGDWGHIN
VDRLLQTDKPEQLPVADQPLYKETLRPQFHFTARQWTTDHLNPGMRQE**GWVNDLNGL**IYYEGEYHLFAQRWNKCWIH
AVSRDLIHWTELEPAFWEKELDSGVQSGTCVIDYHNTSGLGKDPKHPPMIAFWSGNDNRSQCIHYSLDKGRTWIPYA
GNPILD**FPERDPKVF**WYEPGKHWVMMLYGDGKYHVLTSPDLLHWTDEKKPI**DNCFECPDL**FQLPIDGDKTKMQWVLI
QGNGNYSLGKFDGKSFEETSGRRPCDVGPNFYATQTWENISRRVQTAWMRGSDFPNMPFNQQISFPCELTLRTTSAG
PRLFREPVSELSKLHSGQKSWRNQELSAGQSMALEASRDMLRIKADVIIPPKSKLVFKLRGIPVVLTSKTVESGSKP
VEVLEEVQSVEILLDRASIETFVNHGEVSSTRFILPQEDGLTINAEDGPVTIRSLTVYRLKSAWGKVK

>Hduj_GH32_5, ON703169

MEGEAMNLHTIEKAPMQYFKPIGDH**FFAGDCMPF**FHDGVFHLFYLHDENHHAALGGLGGHQWAHATTTDLVNWTHHP
LAIAITNDWEFSICTGSTFYHAGTYYGFYATRNQDWSQHLSLATSADGLTFHKTEPNPYASPLQGYHR**DHYRDPFVF**
WDEPTAQFHMLVTAMLEDYPLHDLGGCLAHLTSTDLHHWTVEEPFYIPGL**RGAPECADH**FYWRGWYYLLFGNDLVTR
YRMSQSPFGPWQRPPSDVLDAPAMRVMKTAPFTGDRRLGAAWIGTREGDKDAGRFQWGGHVVLREIIQHADGTLGTR
FVPELMPAAKQALTVSAVPLTPNVSLTGSQARLDASYSLEAARIGVATPRATIKMRITPSPNSAVFGLRLRSSNNFE
GGYDLRFLPYEQRVELCDQAITGVTGLDQPFDLEIVLRDGIIDVCIDHRRCLINRLPEADGMRVHLFAHNAVVTFDN
MTITADH

Nematodes (Rhabditida, Chromadorea, Nematoda)

Heterodera schachtii

>Hsch_GH32_1, ON736442
MTSDDRMLTLTLTNESIVHFWVHPVTPSDIAKVFLCPSSNDGLQQQQQQVAECAKYSSDRWYEFFEINGAAAGDYFL
RWTEHTKLSLVYVYEPETVLEEGIRIIFVNENAKLPSLGGSYHFRAPF**GWTSDPSGF**VRSKDGLFHLFYQHYPHAKQ
YYLIHWGHAVSKDLVNWVHLPIFMIPPNVLHLDFEVGYFVGSVVNLPSDDFGIFWTQRIPLVLRGTESENPWRENQN
FVILSIPDWNTHKIVLDQCPSAEFPPLG**NGFRDPVVS**LGPGGTNYKCYYYMSVGGERKDGSAGVVLLYRNESKERDR
LDKDWAYQGILWEDSR**DRMCELPML**IALGDPHDPNTKWLLSYMLDKGSYTMCARDAEGRERLNPLLVGHFDGKNFQK
LFEQEMDFARASFAYQGFYDKQSARNFLIGWIFDYDMRTITNDALISVGRAGILSLTELVLSENEQFLLVKPLPELN
SLRMAKEPQNLKKGQKIHLEMGQAELFFQFKWANDGTEEKFELDLTPTHLKGEKLEFRVDHNGIELKKTWEKTDKKR
MLVHKVKPTKIHVFIDLDSVEYFADDGRWSGTVRLTNETSEEKRIGTVELKSAPLLLEESSLWYLKYELHKSALF
>Hsch_GH32_2, ON736443 (**active site region 2 not detected**)
MAKKHTIVLTVVGVCIVVAVIAWGIIRLIIYVIGKTKKSRFYSSQLGGSNTSGIVTVPVQLYSSSILHIWLKTRSAA
QEVPARIRLRHPAMDNVTLAKTDQFIEYEFHELALLLEGPANLEWAKEGGFQSKEGAKDHLKNYFLLPIDRRRNPCL
STSEPVRALLVFCCFFRDVEIKFYLSFSVKNFWHFLSINLIFKFKSDTRVSYIYVFEPDTVLERGIRPAFISNTARF
PDVREGYHFRPPL**GWMNDPNGF**SKFGDHFHLFYQHYPHALHWSAMYWGHAVSRDLINWVHQPIFLLPDPAVALEHDG
IGGIYSGSAVVVIDAVSGRPKMNVYYTDSLNGILMPDYVYQESGCMTDLNHTAMTNWRFLNVLHRDSRVN**TSVVECA**
ALLPLPAPVGSRTSSTVPLWVLLYGQLDAFDPRTGRRNMSPAFVGTFDGRQFRALFEQGFYEQSLDQTLMIGWLANW
RDYDAETKSDFPTSMTVPRQMLLTRDGTTETAPGVLIAPEGLEILPGKSHSTPQRLIALGARPTEVHILLDTGSIEV
FADGGRWAGEFFGTRSLSLSNFRFFLLSSFLSA

Heterodera glycines

>Hgly_GH32_1, AJR19781
MNSVIKLKLITTAFVLLFVCKSDGHTARELNVLLHQDSIVHLWIQPKTRNTVANVYLCPITAGATPNVETELAEQRN
VAECKQYSTNLWYDFYEINGVDKAGEYVLKLTESTKPSLIYVYEQKTVLQEGIRILFINDEAKLPNLEGSYHFRAPF
GWNSDLTGQKMGFSICSINIIPMPCNADFPRGTITEFPWREVQQYVSTKNLIRPDWNTKKTIVEGFPTDVPPLG**KSF**
HDPVVFFGPEGYYYMTIGGERKDGSAGVVLLYRNKNKSADQLDHDWEYQGVVYEDNRFE**LRLCECPML**VAMGDPLDR
NTKWVLSYVSDKGSYPVLGKDAEGRERMNALIVGHFDGHTFEKLFEQQMDFVGGSFAYQGFHDQQSGRSFTIGWICD
IGWIGDNTGDANFDGRGGEFVLKDDHLIVRPLPELAQLRQSKQPHQIRKGEKYSLEKGHAELLFQFKWSNNDDGSAE
EKFVLDLTPTQLKDGKLEFTIDSKGIELKRTWVKPNKRLVVYNVKPTQIHVFIDLDTVEYFADNGRWSGAVRVPNAS
QENRIGTVELKSTPLVLEQSSLWYLKYGSHKSARLQPNGIPFAMNAETSSFKQDEA

Globodera rostochiensis

>Gros_GH32_1, ON703232
MPSSSAKNMLLLAFLFATSLFLIHGTGTEGVSRVNVLLLDDSTLHVWVRPVKKGEVAKVYLCPPTEAATIEQSGQLI
AECKQYSSDLWHDFIEINGKTAGAYTLTWTETTHASLVYVFKPKTVLEEGIRILFIGEEAKLPNLEGSYHFRAPF**GW**
NSDTCGFNRSNDGLFHLFYQHFPHAVQWYSMHWGHAVSKDLINWVHLPIFMIPPNLLHLDFENGHFTGSSINLPNGE
LGVFWSQRISHVPRGTMAEYPWRELQQYVTTKNLIRPDWTTHKTIIERFPTVDPPLG**NNFHDPVVF**LGPGGYYYMTV
GAERKNGSAGLVLLYKNKNKHADQLDRDWEYQGVLYEDKRNQE**LRMCECPML**IALGDPLNANTEWVLSYMSDGGSHP
APIRGTDAEGRDRMNPLFVGRFDGRKFEHRFVQKMDFAGGSFAYQGFYDKQSGRSYLIGWICDISWISENTGDPNFD
GRGGATSSFYCFIAGICSIRKWTIFDGQTIAGVGFSSRKKAATRHNVGQEMHLEKGQAELVFEFSWDNGREETFELH
LTPTHTNGKKLEFRIDNNGLQLKRTWVTSNKRLVVYNAMPTKIHIFIDLDTVEYFADEGRWSGSIRLPEASQEKRIG
TVELKSAPLMLKESSMWYLNYKAHKSAYLQQTANIIPIKKHIFEKQKSSPKSVLDAEFGESPKKGELPALHTIGLRK
>Gros_GH32_2, ON703233
MPSSSAKNMLLLAFLFATSLFLIHGTGTEGVSRVNVLLLDDSTLHVWVRPVKKGEVAKVYLCPPTEAATIEQSGQLI
AECKQYSSDLWHDFIEINGKTAGAYTLTWTETTHASLVYVFKPKTVLEEGIRILYIGEEAKLPNLEGSYHFRAPF**GW**
NSDTCGFNRSNDGLFHLFYQHFPHAVQWYSMHWGHAVSKDLINWVHLPIFMIPPNLLHLDFENGHFTGSSINLPNGE
LGVFWSQRISDVPRGTVAEYPWRELQQYVTTKNLIRPDWTTHKTIIERFPTVDPPLG**NNFHDPVVF**LGPGGYYYMTV
GAERKNGSAGLVLLYKNKNKNADQLDRDWEYQGVLYEDKRNEE**LRMCECPML**IALGDPLNANTEWVLSYMSDGGSHP
APIRGTDAEGRDRMNPLFVGRFDGRKFEHRFVQKMDFAGGSFAYQGFYDKQSGRSYLIGWICDIRWISENTGDPNFD
GRGGATSSFYCFIAGICSIRKWTIFDGQTIAGVGFSPRKKAATRHNVGQEMHLEKGQAELVFEFSWDNGREETFELH

LTPTHTNGKKLEFRIDNNGIQLKRTWVTSNKRLVVYNAMPTKIHIFIDLDTVEYFADEGRWSGSIRLPEASQEKRIG
TVELKRAPLMLKEASMWYLNYKAHKSAFLQQTANIIPIKKHIFEKQKSSPKSVLDAEFGESPKKGELPALHTIGLRK
>Gros_GH32_3, ON703234
MPSSSAKNMLLLAFLFATSLFLIHGTGTEGVSRVNVLLLDDSTLHVWVRPVKKGEVAKVYLCPPTEAATIEQSGQLI
AECKQYSSDLWHDFIEINGKTAGAYTLTWTETTHASLVYVFKPKTVLEEGIRILFIGEEAKLPNLEGSYHFRAPF**GW**
NSDTCGFNRSNDGLFHLFYQHFPHAVQWYSMHWGHAVSKDLINWVHLPIFMIPPNLLHLDFENGHFTGSSINLPNGE
LGVFWSQRISDVPRGTVAEYPWRELQQYVTTKNLIRPDWTTHKTIIERFPTVDPPLG**NNFHDPVVF**LGPGGYYYMTV
GAERKNGSAGLVLLYKNKNKNADQLDRDWEYQGVLYEDKRNEE**LRMCECPML**IALGDPLNANTEWVLSYMSDGGSHP
APIRGTDAEGRDRMNPLFVGRFDGRKFEHRFVQKMDFAGGSFAYQGFYDKQSGRSYLIGWICDIRWISENTGDPNFD
GRGGATSSFYCFIAGICSIRKWTIFDGQTIAGVGFSPRKKAATRHNVGQQMHLEKGQAELVFEFSWDNGREETFELH
LTPTHTNGKKLEFRIDNNGLQLKRTWVTSNKRLVVYNAMPTKIHIFIDLDTVEYFADEGRWSGSIRLPEASQEKRIG
TVELKRAPLMLKEASMWYLNYKAHKSAFLQQTANIIKMEPIKKHIFEKQKSSPKSVLDAEFGESPKKGELPALHTIG
LRK
>Gros_GH32_4, ON703235
MPSSSAKNMLLLAFLFATSLFLIHGTGTEGVSRVNVLLLDDSTLHVWVRPVKKGEVAKVYLCPPTEAGTIEQSGQLI
AECKQYSSDLWHDFIEINGKTAGAYTLTWTETTHASLVYVFKPKTVLEEGIRILFIGEEAKLPNLEGSYHFRAPF**GW**
NSDTCGFNRSKDGLFHLFYQHFPHAVQWYSMHWGHAVSKDLINWVHLPIFMIPPNLLHLDFENGHFTGSSINLPNGE
LGVFWSQRISDVPRGTVAEYPWRELQQYVTTKNLIRPDWTTHKTIIERFPTVDPPLG**NNFHDPVVF**LGPGGYYYMTV
GAERKNGSAGLVLLYKNKNKNADQLDRDWEYQGILYEDKRNEE**LRMCECPML**IALGDPLNANTEWVLSYMSDGGSHP
APIRGTDAEGRDRMNPLFVGRFDGRKFEHRFVQKMDFAGGSFAYQGFYDKQSGRSYLIGWICDISWISENTGDPNFD
GRGGATSSFYCFIAGICSIRKWTIFDGQTIAGVGFSSRKKAATRHNVGQQMHLEKGQAELVFEFSWDNGREETFELH
LTPTHTNGKKLEFRIDNNGLQLKRTWVTSNKRLVVYNAMPTKIHIFIDLDTVEYFADEGRWSGSIRLPEASQEKRIG
TVELKRAPLMLKEASMWYLNYKAHKSAFLQQTANIIKMEPIKKHIFEKQKSSPKSVLDAEFGESPKKGELPALHTIG
LRK

Acrobeloides nanus

>Anan_GH32_1, ON703160
MFLTFFWSPNVKNVVFLVKKYVNFGKKTCRLGPKYAINVTNSRYRQNYHIIAPT**GWLNDPNGL**SYFNGNYHVFFQYD
PTSSNGGGPKQWGHVTSTDLVNWKYTQPTIALAPDQSYDVGGVWTGSAVNNNGELTAIYTGLHNNTGEQLVNIATST
DGVTFTKYSGNPVVPTQPIDGT**NQFRDPKVW**KYNGVWYMVVGNQSKKNHGRTVLYQSDDLRQWTYLGPLADEPDNRV
GSMWECPNFFYINGKHVLFMGTGLNVYFIGDFNYETKVFSHAVDFYELDNGHDFYAAQMFQDPKNRTILFAWMNSFG
TNFEEMQDGWAHTLTVPRQLRLSSDGSRVQMIPVDELTLLRGTEYINSPIEIEGTYDTGIPTSSTEILLNFSLAETT
AQKIGLSFPLNTGHDLRLYYDKQTGKLVLERTKDVRQVDMGSIMQLNLRIFLDQSSIEVFANDGNVTMSSRYYKDPI
PTITLFAEYGVANVEFQAFRLNNIW
>Anan_GH32_2, ON703161
MNHDHNKNYKNCLIFSYTINVTNQRYRLNYHIMPPA**GWSNDPNGLS**YFNGQYHAFFQYCPDNPNGCSTSWGHSISND
LVHWHYAQPTIAISPNQPYDRDGCWSGSAVVNDGQLTLIYAGNVQNPFNQTINIATSTDGVNFTKYSGNPVIAIPPF
DGT**TGFKDPKVW**RSNGTWYVVISNPTQQPNFPRAVLYKSDDLKQWTYLGSLAAEVDDSENL**GSMWDDPNF**FYLNGKF
ILLTSAEGVLRDGNRFANLFQVGYFIGDFDYQTNFFNRSDFYELDLGYDFFAAQVFEDPKGRMILMAWMYTMGSPLP
EQADGWAGALTLPRQLILSEDGTRVKMVPIEELKSLRGNQYVNSPIFINGQFDPKIPCDSTEVIMNISLLQAPGKRI
GMRFGYMQLYYDRQYKKLVLARNKDIRQVDLGNITQVNLRVFFDKSSVEVFANDGAATMTSRFYSDPAPGLILFAES
SNEMPGTANVNLQAFHLNSIYGNN

Meloidogyne enterolobii

>Ment_GH32_1, CAD2191864
MAITYISTNSEKLNNSIERGLSAKVEGKSNNRFSNKKIKIIALTFIGICLVLAVIIWGVCRFNSSSSPNKNKTISVP
INLYKTSILHIWLKTREENQPKKANIKIKNEQINSTIAETNKFKSFVFFELSPQIEGNAFLEWFNGDTWVSYIYVYE
PESVLELGIRPVFISNAARNPEVPEGYHFRPPT**GWMNDPNGF**SKFGQHFHLFYQNYPHALKWSPIHWGHAVSQDLVN
WVHLPIFLLPDPSLDVNTTGGIFSGSAISFPNELRIFYTDSLIGRIPMEFQKMVTTINGIHPNELSKTVIPEGPSKL

NLT**QDFRDPNVI**LGPDGKWKMILGSRDSQGGVILLYGTDDPTAQSGWHFLNVLYRDNRLG**MTVAECSGL**APIDGNPQ
DPNTLWALIYAQLNSTDPATGRRDITTVHVGKFDGITFTPMFEQEMDFGTHAYAFQALYSQELGTLTIAWLANWKDW
NWDTKPDFPTSMTLPRKLVLSADRKALLTPPIDWVVSKLRNQQLDLDKQLLKGQFVDLPNGTAEISISLQNFQSNIL
EPITSDQRSSNNSNNENLKFIRLEIEHPKITNVGVEISFEGIEILYGLPKQPQQRLIALGAKPSQVQILLDTGSIEV
FADGGRWTGTVRIPGTNKFSKIRLIGDLSMVTRSEIWGLKPAEFKGKLQIKK

>Ment_GH32_2, CAD2179544
MTITYISTNSEKLNNSLERGLSAKVEGKSNNRFSNKKIKIIALTFIGICLVLAVIIWGVCRFNSSPSPNKNKTMSVP
INLFKTSILHIWLKTREENQTNKANIKIKNEQINSIIAETNKFKSFVFFELSPQIEGNAFLEWSNGDTWVSYIYVYE
PESVLELGIRPAFVANAARDPEVPEGYHFRPPT**GWMNDPNGF**SKFGQHFHLFYQNYPHALKWSPIHWGHAVSQDLVN
WVHLPIFLLPDPSLDVNTTGGIFSGSAISFPNELKIFYTDSLIGRIPMEFQKMVTTINGIHPNELSKTIIPEGPPKF
NLT**QDFRDPNVIL**GPDGKWKMILGSRDSQGGVILLYGTDDPTAQSGWHFLNVLYRDNRLG**MTVAECSGL**APIDGNPQ
DPNTLWALIYAQLNSTDPATGRRDITTVHVGKFDGITFTPMFEQEMDFGTHAYAFQALYSQELGTLTIAWLANWKDW
NWDTKPDFPTSMTLPRKLVLSADRKSLLTPPIDWVISKLRNQQLDLDKQLLKGQFVDLPNGTAEISISLQNFQSNIL
EPISSDQRSSNNSNNENLKFIRLEIEHPKITNVGVEISFEGIEILYGPPKQPQQRLIALGAKPSQVQILLDTGSIEV
FADGGRWTGKFKFKNKYKFIYKIGTVRIPGTNKFTKIRLIGDLSLVTRSEIWGLKPAEFKGKLQIKK
>Ment_GH32_3, CAD2180476
MAITYISTNSEKLNNSIERGLSAKIEGNKNRFSNKKIKIIALTFIGICLVLAVIIWGVCREENQPKKANIKIKNEQI
NSTIAETNKFKSFVFFELSPQIEGNAFLEWFNGDTWVSYIYVYEPESVLELGIRPAFIANAARNPEVPEGYHFRPPT
GWMNDPNGFSKFGQHFHLFYQNYPHALKWFPMHWGHAVSRDLVNWVHLPIFLLPDPSFDVNTTGGIFSGSAISFPNE
LKIFYTDSLIGRIPMEFQKMVSTINGIHPNELSKTVIPEGPSKLNLT**QDFRDPNVI**LGPDGKWKMILGSRDSQGGVI
LLYGTDDPTAQSGWHFLNVLYRDNRLG**MTVAECSGL**APIDGNPQDPNTLWALIYAQLNSTDPATGRRDITTVHVGKF
DGTTFTPMFEQEMDFGTHAYAFQALYSQELGTLTIAWLANWKDWNWDTKPDFPTSTILPRKLFLSADRKSLLTPPID
WVISKLRNQQLDLDKQLLKGQFVDLPNGTAEISISLQNFQSNILEPISSDQRSSNNSNNENLKFIRLEIEHPKITNV
GVEISFEGIEILYGLPKQPQQRLIALGAKPSQVQILLDTGSIEVFADGGRWTGTVRIPGTNKFTKIRLIGDLSLVTR
SEIWGLKPAEFKGKLQIKK
>Ment_GH32_4, KAF7632376
MDSPTPQSHLKPSFRKKPLIIIFIAFCAFVIVSSITFGLLHLIKNHNSQEFDATTISSIISSTQYYTTDNPPFKKYV
YVHVNLINTSVLHIWLKPKLENTTATLYANVSKTNSNESFPTALFYSAKIGPPTWFDFVELELNITGIIELSWSEMT
TIVSYIYTYEPEEVKFQGIRPAYISKQAMNPPMDQSFHFRPPL**GWINDPNGF**SRTRDGLYHLYYQHYPHSKRWSSMY
WGHAISYDLVNWIHQPIFLRPNDKECGENCGCYSGSAVPNGNDGLQVFFTDNNNTRNHQQIQRTIYTKDTIAPSNES
EIILNDLPDLSEKELKLS**SDFRDPNVF**ISPDGYHYMTLGSGDTLGWQFQNVIKTDNLFG**TKVCECSQI**FQLGDKNRR
ETLYALLYARMGSKDDDGRSNLTPIILGTFNGTHFEKIYEQELDFAAGSFGVQAFYDPIADEALLLDG
>Ment_GH32_5, CAD2180252
MDSPTPKSFSKPNSCLKTFLLVFAGLCAILIIASITFGLLQLIKSHDITEPETSTIPSVNTETSYSSTTDAGCVQNS
YSVYVDLINTSIIHVWMKPKRLGSTAILYANISNTNQTTPTPIFYTSENGEPTWFDFAQLELNFNGKVELSWSEKDT
NVSYIYAYETENVKRNGIRPAFVSKLAMGPNMEQSFHFRPPL**GWTNDPNGF**SRTKDGLYHLYYQHYPHSKQWNSMYW
GHAISTDLVNWIHQPIFLRPDDKECGTDCGLFSGSAVPIHDGDGLQVFFTDNNNTRINPEIQRTVYTKDTILPYNES
EIIIHELPDLEEKGLKLG**ADFRDPNVF**LGPEGYYYMTLGSSDTLGAGGVVLLYRSKEKHSVHSGWIFQNVLRTDNAF
G**TKVCECSQI**FQLGGRRRLSQTLYALLYARTGSKDGDGRANLSPIILGNFDGSNFNPIEEQELDFGAGSFGFQAFYD
TIDDEALLIGWLANWPDWDRISDWSTSLTLPWVIRWNNRQLLIGPHPNVLGQLRDIELDGKALLRGNAVVLPNGTAY
IRLAIEYYYFYNGIFLQLNHPDYIQLGVKVSKDGLEIVKVKHNDPPNRRFLAADARPDIVHIYIDRDSLEVFGEMKN
DGMPRWAGTSRLTGLNTFVESVQLSTTDRYSYLLQGCQIWSMKPAKFLGRL
>Ment_GH32_6, CAD2182172
MDSPTPKSFSKPNSCLKTFLIVLAGLCAILIVASITFGLLQLIKSHDITEPETSTMPSVNTETSYSSTTDSGCVQNS
YSVYTAILYANISKTNTNQTTPTPIFYTSENGEPTWFDFAQLELNFDGRVELSWSEEDTNVSYIYAYETENVKRNGI
RPAFVSKLAMGPNMEQSFHFRPPL**GWTNDPNGF**SRTDDGLYHLYYQHYPHSKQWNSMYWGHAISTDLVNWIHQPIFL
RPDDKECGTDCGLFSGSAVPLHDGLQVFFTDNNNTRINPEIQRTVYTKDTILPFNESEIIITDLPDLDEKGLKLG**AD**
FRDPNVFLGPEGYYYMTLGSSDTLGAGGVVLLYRSKEKHSVHSGWIFQNVLKTDNAFG**TKVCECSQI**FQLGGRRRLS
QTLYALLYARTGSKDGDGRANLSPIILGNFDGSNFNPIEEQELDFGAGSFGFQAFYDTIDDEALLIGWLANWPDWDR
ISDWSTSLTLPWIIRWNNRQLLIGPHPNVLGQLRDIELDGKALLRGNAVVLPNGTAYIRLALEYYYFYNGIFLQLNH
PDYIQLGVKVSKDGLEIVKVKHNDPPNRRFLAADARPDIVHIYIDRDSLEVFGEMKNDGMPRWAGTSRLTGLNTFVD
SVQLSTTDRYSYLLQGCQIWSMKPAKFLGRL

>Ment_GH32_7, CAD2157872
MDSPTPKSFSKPNSCLKTFLIVIAGLCAILIVASITFGLLQLIKSHDIIEPETSTIPSVNTETSYSSTTDAGCVQNS
YSVYVDLINTSIIHVWLKPKRLGSTAILYANISKTNTNQSTPTPIFYTSENGEPTWFDFAQLELNFNGKVELSWSDE
DTNVSYIYVYETENVKRNGIRPAFVSKLAMGPNMEQSFHFRPPL**GWINDPNGF**SRTDDGLYHLYYQHYPHSKQWNSM
YWGHAISTDLVNWIHQPIFLRPDDKECGTDCGLFSGSAVPIHDGLQVFFTDNNNTRINPEIQRTVYTKDTILPFNES
EIIISDLPDLKEKGLKLG**ADFRDPNVF**LGPEGYYYMTLGSSDTLGAGGVVLLYRSKEKHSVHSGWIFQNVLRTDNAF
G**TKVCECSQI**FQLGGRSRRSQTLYALLYARTGSKDGDGRANLSPIILGNFDGSNFNPIEEQELDFGAGSFGFQAFYD
TIDDEALLIGWLANWPDWDRISDWSTSLTLPWVIRWNNRQLLIGPHPNVLGQLRDIELDGKALLRGNAVVLPNGTAY
IRLAIEYYYFYNGIFLQLNHPDYIQLGVKVSKDGLEIVKVKHNDPPNRRFLAADARPDIVHIYIDRDSLEVFGEMKN
DGMPRWAGTSRLTGLNTFVESVQLSTTDRYSYLLQGCQIWSMKPAKFLGRL

Rotifers (Philodinida, Bdelloida, Rotifera)

Adineta ricciae

>Aric_GH32_1, CAF0964479

MTNQTYSICAKLIARNQLVSVNATGALLLSLTFGKRTTMTDSQQSYFMHREKRHLDTEKIFTDGKILVIVNKTLWFS
FGKSCGLWYMVGNSNYVVNNLSQATIVKIVLLCMTVFILRVATAHYKTRDTFKPSSSNDLKTLANRVPSHQHRPLVH
FSPSK**NWINDPNGL**VFYDGEWHLFYQHNPHTPMSVNMHWGHSVSEDLIAWTELPIAIEPEDDVVGIWSGSVVIDWKN
TTGFQKNMSTHPMIAIYTWQKQRWQEQHMAYSLDRGRTWTKYSSNPIIPMYNNVSAADRLSDQVVR**RPFRDPKVF**YH
ILTSSWILVLTGGDHVQFYRSSDLIHWSLMSRFGLTDGSH**GGTWECPDL**IEFPQRTLKNRTSLWVLLVSVQRNAPAG
GSGMQYFIGTFDGNEFHNIQPPETINWLDYGPDFYAGITFHNIPGYDRRQILISWMSNWQYANSLPTAPLWRGQMTI
PRQLQLDLNSYTNRYHLRQTPVQELIRYSKRLITYYHEKLSNMVLNMSRDVYMFNIELHKITKATTIHLKFRENSDK
SEYTQVSYIGEKNQIELDRSRSGLTDFYSSYPVQFNMNLDRETLITGRLQLQLIIDRCSIELFVNEGKYTMTSLIFP
KDSGYQIDISVDGHEIFINYLELMLFSL

>Aric_GH32_2, CAF1050785

MVGNSNYMVNNLSQATIVKIVLLCMTVFILRVATVHYKTRDTIKPSSSNDLKTLTNRVPSHQHRPLVHFSPSK**NWIN
DPNGL**VFYDGEWHLFYQHNPHTPTSVNMHWGHSVSEDLIAWTELPIAIEPEDDIVGIWSGSVVIDWKNTTGFQKNMS
THPMIAIYTWQKQRWQEQHMAYSLDRGRTWTKYSSNPIIPMYNNVSTADRLSDQIVR**RPFRDPKVF**YHIRTSSWILV
LTGGDHVQFYRSSDLIHWSLVSQFGLTDGSH**GGTWECPDL**IEFPQRTLKNRTSLWVLLVSVQRNAPAGGSGMQYFIG
TFDGNEFHNIQPPETINWLDYGPDFYAGITFHNIPGYDRRQILISWMSNWQYANNLPTAPLWRGQMTIPRQLQLDLN
SYTNKYHLRQTPVQELTRYSKRLITYHHEKLSNMVLNMSRDVYMFNIELHKITKATTIHLKFRENSNKSEYTQVSYI
GEKNQIELDRTRSGLTDFHSSYPVQFNMNLDRETLITGRLQLQLIIDRCSIELFVNEGKYTMTSLIFPKDSGYQIEI
TVDGHEIFINYLELMLFSL

Adineta vaga

>Avag_GH32_1, UJR14897

MIHNYKYMIDNLSSATIGKLIGLCITIFILRFATTHYKTRNSSIALSSNDLESLNNSISVYQYRPLVHFSPSK**NWIN
DPNGL**VYYHDEWHMFYQHYPLAPMAINMHWGHSISRDLITWTELPIAIEPEDDIVGIWSGSVVIDWKNISGLQKNSN
IHPMIAIYTWQKQRWQEQHMAYSLDRGRTWTKYSSNPIIPLYNNVSTADRFSDKIVR**RPFRDPKVS**FHSPTASWILV
LTGGNHIQFYRSVDLIHWSLISRFGFTDGSH**GGTWECPDF**IEFPQTTLKNQTSLWILIVSVQNGAPAGGSGMQYFIG
TFDGITFRNLQSSSTINWLDYGSDFYAGITFHNVPRYDNRQILISWMNNWKYARDLPTAPLWRGQMTIPRQLQLDWN
SFTNMYHLRQIPVHELYSYSKRFITFYRQKLSSNILLNISRDVYMFNSEFHNITKATNIHLNLRQNFNKSEYTQINY

IGEKNQIEFDRSNSGLVDFHASYSLHFNMILDDETLTTGKLKLQLIVDRCSVELFINDGKYTMTALIFPKFSGYQIE
LSVDGHEIFINFLELMLLSQ

Adineta steineri

>Aste_GH32_1, CAF1103651

MYRHHNNSINNLSRTIIGKILILGIIIYLVKYVMSDHTNHNSISESNSLPEISNTIIPHHQHRPLIHFSPKK**NWIND
PNGL**VYYDTEWHLFYQYYPKAPISINMHWGHAVSKDLITWNELSIAIPPEDDIVGIWSGSVVIDWKNITGFQKNTNI
HPMIAIYTWQYQRWQEQHMAYSLDKGRTWTKYESNPVIPLYNNVSKADRLSDKITR**NAFRDPKVM**FHVPTNSWILVL
TGGDHVQFYKSVDLIHWSLISHFGHNEGSH**GGTWECPDL**IEFPQVKLKNQTNLWVLLVSVQRGSPAGGSGMQYFIGT
FDGYIFHNIQSSQTINWLDYGSDFYAGITFHNIPQYDGRHILISWMNNWQYARELPTAPLWRGQMTIPRQLQLDFNS
FTKTYHLRQLPAHELYLYSKQLLTFHRRKLSSKSANLILNSSHDVYMLNTEFYNITKTTNIHIRLRQTIDKPEYTEI
KYIGNKNQIEFDRSHSGNINFHNSFYPQFNMSLDKETLTTGILKLQIIVDRCSIELFINGGKYTMTALIFPKHQGYQ
IELSIDGEDILLNYLELMLFSM

>Aste_GH32_2, CAF3659239

MYRHQNNSINNLSRTIIGKILILGIIIYLVKYVMSDHTNHNSISESNSLPEVIKEIPNTIITDHQHRPLIHFSPKK**N
WINDPNGL**VYYDTEWHLFYQYYPKAPMSINMHWGHAVSKDLITWNELLIAIPPEDDIAGIWSGSVVIDWKNITGFQK
NTNIHPMIAIYTWQYQRWQEQHMAYSLDKGRTWTKYESNPVIPLYNNVSKADRLSDKITR**NAFRDPKVM**FHVATNSW
ILVLTGGDHVQFYRSIDLIHWSLISHFGHNDGSH**GGTWECPDL**IEFPQVKLKNQTNLWILLVSVQRGSPAGGSGMQY
FIGTFDGYIFHNIQSSQTINWLDYGSDFYAGITFHNIPQYDGRHILISWMNNWQYARELPTAPLWRGQMTIPRQLQL
DFNSFTKTYHLRQLPAHELYLYSKQLLTFYRRKLSSKSANMILNSSHDVYMLNTEFHNITKTTNIHIRLRQTIDKLE
YTEIKYIGNKNQIEFDRSHSGNINFHNSFYPQFNMSLDKETLTTGILKLQIIVDRCSIELFINGGKYTMTALIFPKH
QGYQIELSIDGEDILLNYLELMLFSM

>Aste_GH32_3, CAF3483435

MYRHQNNSINNLSRTIIGKILILGIIIYLVKYVMSDHTNHNSISESNSLPEVIKEIPNTIITDHQHRPLIHFSPKK**N
WINDPNGL**VYYDTEWHLFYQYYPKAPMSINMHWGHTVSKDLITWNELLIAIPPEDDIAGIWSGSVVIDWKNITGFQK
NTNIHPMIAIYTWQYQRWQEQHMAYSLDKGRTWTKYESNPVIPLYNNVSKADRLSDKITR**NAFRDPKVM**FHVPTNSW
ILVLTGGDHVQFYKSVDLIHWSLISHFGHNEGSH**GGTWECPDL**IEFPQVKLKNQTNLWVLLVSVQRGSPAGGSGMQY
FIGTFDGYIFHNIQSSQTINWLDYGSDFYAGITFHNIPQYDGRHILISWMNNWQYARELPTAPLWRGQMTIPRQLQL
DFNSFTKTYHLRQLPAHELYLYSKQLLTFHRRKLSSKSANLILNSSHDVYMLNTEFYNITKTTNIHIRLRQTIDKPE
YTEIKYIGNKNQIEFDRSHSGNINFHNSFYPQFNMSLDKETLTTGILKLQIIVDRCSIELFINGGKYTMTALIFPKH
QGYQIELSIDGEDILLNYLELMLFSM

>Aste_GH32_4, CAF1512156

MYRHHNNSINNLSRTIIGKILILGIIIYLVKYVMSDHTNHNSISESNSLPEISNTIIPHHQHRPLIHFSPKK**NWIND
PNGL**VYYDTEWHLFYQYYPKAPMSINMHWGHAVSKDLITWNELSIAIPPEDDIAGIWSGSVVIDWKNITGFQKNTNI
HPMIAIYTWQYQRWQEQHMAYSLDKGRTWTKYESNPVIPLYNNISKADRLSDKITR**NAFRDPKVM**FHVPTNSWILVL
TGGDHVQFYKSIDLIRWSLISHFGHNHGSH**GGTWECPDL**IEFPQVKLKNQTSLWVLLVSVQRGSPAGGSGMQYFIGT
FDGYIFHNIQSSQTINWLDYGSDFYAGITFHNIPQYDGRHILISWMNNWQYARELPTAPLWRGQMTIPRQLQLDFNS
FTKTYHLRQLPAHELYLYSKQLLTFHRRKLSSKSANMILNSSHDVYMLNTEFHNITKTTNIHIRLRQTVDKLEYTEI
KYIGNKNQIEFDRSHSGNVKFDDSFFPQFNMSLDKETLTTGILKLQVIVDRCSTELFINGGKYTMTALIFPKYQGYQ
IELSIDGEDILLNYLELMLFSM

>Aste_GH32_5, CAF0887994

MYRHHNNSINNLSRTIIGKIMILGIIIYLVKYVMSDHTNHNSISESNSLPEVVKEISNTIIPHHQHRPLIHFSPKK**N
WINDPNGL**VYYDTEWHLFYQYYPKAPMSINMHWGHAVSKDLITWNELSIAIPPEDDIAGIWSGSVVIDWKNITGFQK

NTNIHPMIAIYTWQYQRWQEQHMAYSLDKGRTWTKYESNPVIPLYNNISKADRLSDKITR**NAFRDPKVM**FHVPTNSW
ILVLTGGDHVQFYKSIDLIHWSLISHFGHNEGSH**GGTWECPDL**IEFSQVKLKNQTNLWVLLVSVQRGSPAGGSGMQY
FIGTFDGYIFRNIQSSQTINWLDYGSDFYAGITFHNIPQYDGRHILISWMNNWQYARELPTAPLWRGQMTIPRQLQL
DFNSFTKTYHLRQLPAHELYLYSKQLLTFHRRKLSSKSANLILNSSHDVYMLNTEFHNITKTTNIHIRLRQTIDKLE
YTEIKYIGNKNQIEFDRSHSGNIKFDDSFFPQFNMSLDKETLTTGILKLQVIVDRCSTELFINGGKYTMTALIFPKF
QGYQIELSIDGEDILLNYLELMLFSM

Macrotrachela quadricornifera

>Mqua_GH32_1, ON703170

MKVHLLLLILIKFVYSINLNSISLNTLKLPEDLSQVNLSTLPDNSLFTKWRPTYHFASPN**SWMNDPCGP**LYNPTTQN
YHLYYQVQPGYVQWGNISWGHAKSKDMIFWEDVISWEGYNYIALAPGVGNNQSIRGVFSGSALPVSINGDTENGTIT
VIYTSVKQLPISWNGFYSLGSETQSLAVSYDDGVTFQHYDNNPILRSSPIEMNV**TGWRNPKLK**KWPEMDILLYGSNK
GNYYMTISSGIQGIGPRVLLYEAPPNNLTRWTYLGPLLAVNGNYTINEIWSGSL**GYNFEASNA**CSLLEKAVDGGDNR
TIYTVALLGTEGGNTTLHQSTRWSVWIHGNLIQTSNGSPTMNILAGGVLDWGDTYALHSFHDLKGDRRILYGWVLEA
NNNYGERAFGYNGQITLPREVFVQIYHNVVDVDGSLVQPGPWTVIPNSNGTYTFKTLGIKPIDEITKLRQNNSVVHI
ISQTFNQPTLFTSLNISSSNFELQTSINISSTTSAGLVFRRSSNGLEYTTLIYDPVSQYLILNRTYSSLVTMFANST
IYAKHALLTTKINNRTTQMELLSLRIFVDNSLVEVYANNRTAIATHIYPTLSDSIGIGYIVGEQGGSITFGETSFWF
DLQNAFPKRPANSSINLVDSTNTISGVSFQNSFISLVFLFVLFFLYYIY

>Mqua_GH32_2, ON703171

MIMQVSILFLILNQILLFIYCISIENSKFNDNNLNKNDLICNKPSDDNNYIIYS**LSIDDNSPI**GDVMPYFDYELNQL
YILYLKDIWNDFNHQRHPWYGLKTNNFYSYSLINQDELLNSNTNECSQDYAIGTGSIIKKDNIYYAFYTGHNPNSPS
YCIKTKEGIMLATSLNLNNKFIKDLNFKTIYPPIEKNFDEE**NNFRDPFVF**YDINLKKYTLIISARTTRGVIIYYTSY
DLYNWLYQGILYDGDSTY**FYMMETPDL**FQINNIYYLIFSDIDTKNVYYRKSLSLYGPWEKPTEHLIDRFDAYGLYGA
KVIRDNYNDYYIFGWTNQYENNNDYGQWKWGGNLIIHKLYQLEYSKDLAITIPHTIKNYFEIFNQPIIKHSQWGIVQ
DLSTDILSYSLSSLLNSEITNVLFNPINLIRYKISTIISYTRSIKDFGFMINVCDGYNQFYSLRFIPSQHRFSFDKI
NRSSLTSTTISTIDIQYKFIPNTDYIIHIIIENSMVITYIDHKVALTARIYNAGKNSWGIFADNSNVIFKNLTVTRP

>Mqua_GH32_3, ON703172 (**D in active site region 2 substituted to N, but there is an adjacent D**)

MKVHLLLLILIKFVYSINLNSISLNTLKLPEDLSQVNLSTLPDNSLFIKWRPTYHFASPN**SWMNDPCGP**LYNPTTQN
YHLYYQVQPGYVQWGNISWGHAKSEDMIFWEDVISWEGYNYIALAPCVGNNQSIKGVFSGSALPVSINGDTENGTIT
AIYTSVKQLPIIWNGFYSLGSETQSLAVSYDDGVTFQHYDNNPILRSPPIEMNI**TGWRNPKLK**KWPEMDIVLYGSNK
GNYYMTISSGIQGVGPRVLLYEAPSNNLTRWTYLGPLLAVNGNYTINELWSGSL**GYNFEASNA**FSLLEKAADSGDNR
TIYTVALLGTEGGNTTLHQSTRWSVWIHGNLTQTSNGNPMMNILTGGVLDWGDTYTLNSFHDPKGDRRIFYGWVMEA
NNNYGERAFGYNGQFSLPREVFVQIYHNVFDVDGSLVQPGPWTVIPNSDGTYTFKTLGIKPIDEITKLRQNNNVVYI
ISQTFNQPTSFTSLNVSSSNFELQTSINIFSNTSAGFVFRRSSNGLEYTSLIYDPVSQYLILNRTYSSLVTMFANST
IYAKHALLTTKINNHTTQMELLSLRIFVDNSLVEVYANNRTAIVTHIYPTLSDSIGIEYVVGGQGGSITFGETSFWF
DLQNAFPKRLANSSINLVDPTNIISGVSF

>Mqua_GH32_4, ON703173 (**D in active site region 2 substituted to N, but there is an adjacent D**)

MKVHLLLLILIKFVYSINLYSISLNTLKLPEDLSQVNLSTLPDNSLFTKWRPTYHFASPN**SWMNDPCGP**LYNPTTQN
YHLYYQVQPGYVQWGNISWGHAKSEDMIFWEDVISWEGYNYIALAPGVGNNQSIKGVFSGSALPVSINGDTENGTIT
AIYKSVKQLPIIWNGFYSLGSETQSLAVSYDDGVTFQHYDNNPILRSPPIEMNI**TGWRNPKLK**KWPEMDIVLYGSNK
GNYYMTISSGIQGIGPRVLLYEAPSNNLTRWTYLGPLLAVNGNYTINELWSGSL**GYNFEASNA**FSLLEKAADSGDNR
TIYTVALLGTEGGNTTLHQSTRWSVWIHGNLTQTSNGRPIMNILAGGVLDWGDTYALNSFHDPKGDRRILYGWVMEA

NNNYGERAFGYNGQITLPREVFVQTYHNVVDVDGSLVKPGPWTVIPNSNGTYTFKTLGIKPIDEITKLRQNNNVVHI
ISQIFNQPTSFTSLNVSSSNFELQTNINISSNTSAGFVFRRSSNGLEYTSLIYDPVSQYLILNRTYSSLVMMFANST
IYAKHALLTTKINNRTTQMELLSLRIFVDNSLVEVYANNRTAIATHIYPTLSDSIGIGYVVGGQGGSITFGETSFLF
DLQNAFPKRLANSSINLVDPTNIISGVSF

>Mqua_GH32_5, ON703174

MKMRLPFFVLIVITLLYSANTTKILSNIFKQFQNLSQINLTALPDNSLFTKWRPTYHFASPN**SWMNDPCGP**LYDPAT
ETYHLYYQVQPGHVEWGNISWGHAKSKDMIIWEDVTSWRGYDYITLTPGIGNNQSILGVFTGSTLPVTITGDLTKGT
ITTIYTSVKYLPISWNGPYHKGSETQSLAISYDGGVTYQQYANNPILAAPPEDMNV**TGWRDPKFE**QWPELDIVLYGS
NQEHYYMTISSGIRGVGPRLLLYQASINDLTDWTYLGPLISVSGNYTFNEIWSGSF**GYNFEVSNT**FSLLEKAADGGD
NYTIHTFAALGTEGGNTTLHQSLQWSIWIKGNLIKTGNGSPTMNIVAAGVADWGDAYALNSFYDPKGDRRIFYGWIK
EAHKNYGQRAFGYNGQITLPREVFVQVYKNVVNISGSLSKQGPWILISNTNGTYTCKTLGIRPINEVTKLRYNSTFI
NLASQTFNQSTKFTSFNIFSSNFELKANINISSNAAAGFVFRRSSDGLEYTTLIYDPVSEYLILNRTYSSLITQFDH
SPVYAKHTLLTRITNKNTIQKELLSLHIFLDNSLLEVYANNRTVMATHIYPTLSDATGLGYIVDRDDGPVTFNNVFI
WSNLQNAFPYRPANSSITLVADSDNITSTSSPLPYFYTSLFFLLFFVQ

>Mqua_GH32_6, ON703175

MFLFTFIIIIFEVTLADDPLRPQYHLMPSQ**NWLNDPNGP**VYYNGYYHMFYQYYPNPVPSDQKHWGHCYSKDMVHWIR
LPIALSPDQPYDINGVWTGSTSIVNDIPIIIYTGISDAHVQVQCQARPSNLSDITLTNWTKSPLNPLITS**PNGRDPA**
TSFKDNQNNHYLIYGYGTEDLGGQAVLFTSKDFLNWTYLHPIHSNHY**DQFWECPDI**FNLSNHMVLKASLLGRDFWTL
GSIDPVKLIFTPINHDLGEFTQLIDYGKFYASKTFYDPLNNQQVVVGWTAEDDDQGEQRGWQGLLTLPRAIFLSNDG
LQLRTRPIEAVKTLRDPNSHQQYHDITIGSEIPFELIPNINGNQIEILINWQFPMNQV

>Mqua_GH32_7, ON703176

MFLFTFIIIIFEVTLADDPLRPQYHLMPSQ**NWLNDPNGP**VYYNGYYHMFYQYYPNPVPSDQKHWGHCYSKDMVHWIR
LPIALSPDQPYDINGVWTGSTSIVNGIPIIIYTGISDAHVQVQCQARPSNLSDITLTNWTKSPLNPLITS**PNGRDPA**
TSFKDNQNNHYLIYGYGTEDLGGQAVLFTSKDFLNWTYLHPIHSNHY**DQFWECPDI**FNLSNHMVLKASLLGRDFWTL
GSIDPVKLIFTPINHDLGEFTQLIDYGKFYASKTFYDPLNNQQVVVGWTAEDDDQGEQRGWQGLLTLPRAIFLSNDG
LQLRTRPIEAVKTLRDPNSHQQYHDITIGSEIPFELIPNINGNQIEILINWQFPMNQV

>Mqua_GH32_8, ON703177

MFLFTSLIIIFGVIYADDPLRPQYHLMPEK**NWLNDPNGP**VYYNGYYHMFFQYNPNAAVWGDMHWGHCYSEDMVHWIH
LPVALAPDQSYDKNGIFTGSTTIVNGIPTIIYTGITSDNTQVQCQAQPVDISDPTLTNWKKSLLNPLITN**PNGRDPS**
TAFQDDENNYYLIYGYGTKDLGGQAVLFTSKDFLNWTYLHPIHSNQY**DTFWECPDI**FNITNRIVLKASLLGHDFWTI
GDIDPKELIFKPINHDLGEFIQLIDHGKFYASKTFYDPIHDQQIIMGWTSEDDNLGPQRGWQGFHTLPRMIFLSEDG
LELRTRPIDALKTLRNSQSHRHYTDVILPSYLPFELIPDVNGNQIEIEIDWQFPMNQVKY

Didymodactylos carnosus

>Dcar_GH32_1, CAF0755962

MAMKDEALPLSLDGRSRNPTTQVPTINYTGRAEADIRRSPNEGIGACGWALVILSYFLMIITLPFSLFLTIKVVQEY
ERAVILRLGRILPGGAKGPGLFFILPCVDTIVKVDLRTATFNVPPQEVLTRDSVTVSVDAVVYSRVFNPIISVTNVK
NTQYATQLLAQTSLRNILGTKTLQEILSDRETIAHSMQVHLDEGTDPWGVKVERVEIKDVRLPVSMQRSMAAEAEAS
REARAKVIAAEGEQKASRSLKEAADIISESPIALQLRYLQTLTHISAEKNSTIIFPIPVLLRNQNFELSYDALSDWI
TNNSNIFHIESKNDQCCTSINGMYHLVSPVNGTGSIQTDNFIVSGLGWMDFLIAGSANPDLCRLELVLTENNIVIYS
ESGPGLWPSSSGNSKPSWRHTRKFWNVSSYLGKTVYLRLIDYSTDGFIAIDDIHTEVSHLDILFNIRYLNYRPLYHF
SPDT**MWMNDPNGL**VYYDQEYHLFFQHYPYDKKAMNIHWGHAVSKDLITWTQLPIALPPEDAVAGIWSGSAFVDWNNA
SGFQKNHNTKPLIAIYTHQKHGHQEHMAYSNDRGRTWTKYVNNPILPMHSIDSKSTEENDNILTLVH**PVFRDPKVF**

YHYETESWILVVSGGTHVKFYRSDNLVNWKLMSEWGKEHYSKTH**PGVWECPDL**FKMETEQDKNVWVLIISINRNSVS
GGSGMQYFVGTFDGVQFTLNSKSLNTSLWLDYGPDYYAGITYSDIPHYDNRRIMISWMNNWLYGRDLPTSVWCGQMG
IPRSLKLYIDHYTNSYRLSQQPVIELYHYQRKLISFHRQMIHQGSNLLENILGVTYIITASFNLNTSIFNCQTCELG
FQFRKSSENMKEYKEYTTIRYVPYNNTIYFDRSQSGETRFHSGFRTNYNITLDDEVFDKGLLKFQILVDRLSVELFI
NDGKYAFTALIFPDFNSQNMALFTNGADVMIDYADILLL

>Dcar_GH32_2, ON703180

MILLLLFVNLFPNVLVQSAKNSTTISTIPTGASLAQTNLNTLPNNILFNQWRSQYHFLAPN**SWMNDPCGP**VYHSPSQ
TYHLFYQWNPQHIKWGNISWGHATSKDLIKWTDVPLSNNNYMTPGISTEANSTYDNLGIFTGTAQTLSNGSILIIYT
SVRHLPISWALPYINNSESQSLILSNDNGTTWQRYANNPVIATPPPNVNI**TGFRDPFLQ**KWREMDTLLNNSNNWYVT
VSSGTKTKGTNLFLYTASDNTIQNLTYLGNLISLTENFTFNENWSGTW**GFNLEVATV**FSLNETVADGGEEGVMRTYV
TAGSEGGNTTLHTSAHWSVWAEGLVINDKNASAYMTLKSGGVADWGATYAMLSFLDSQERRVFFGWVPDDFNDYALT
QIGYNGQFILPRQAFIQVTPDITPGSLPSSLLTSGPFTVQAQPNGLWRVHTLGFRVLPEFTTWFNSLSSVVTFLNNQ
MVTNSSTHLLSNFLTSNRYELNVTVMNYTTTFPTITIYLATDPQYQEYTMLTYNFMTYDLTIIRTHSSLITQFDSST
VYAKHFLFSNKESLQLRIFLDNSLLEVHANERTSLATHIYPVLNTSTQVGYAVSNGAGVQVTVIGRDLSKANVWPQR
PINTSVPLVYDTAAETNNYTYWTGN

>Dcar_GH32_3, ON703181

MILLLLFVNLFPNVLVQSAKNSTTISTIPTGASLAQTNLNTLPNNILFNQWRSQYHFLAPN**SWMNDPCGP**VYHSPSQ
TYHLFYQWNPQHIKWGNISWGHATSKDLIKWTDVPLSNNNYMTPGISTEANSTYDNLGIFTGTAQTLSNGSILIIYT
SVRHLPISWALPYINNSESQSLILSNDNGTTWQRYANNPVIATPPPNVNI**TGFRDPFL**QKWREMDTLLNNSNNWYVT
VSSGTKTKGTNLFLYTASDNTIQNLTYLGNLISLTENFTFNENWSGTW**GFNLEVATV**FSLNETVADGGEEGVMRTYV
TAGSEGGNTTLHTSAHWSVWAEGLVINDKNASAYMTLKSGGVADWGATYAMLSFLDSQERRVFFGWVPDDFNDYALT
QIGYNGQFILPRQAFIQVTPDITPGSLPSSLLTSGPFTVQAQPNGLWRVHTLGFRVLPEFTTWFNSLSSVVTFLNNQ
MVTNSSTHLLSNFLTSNRYELNVTVMNYTTTFPTITIYLATDPQYQEYTMLTYNFMTYDLTIIRTHSSLITQFDSST
VYAKHFLFSNKESLQLRIFLDNSLLEVHANERTSLATHIYPVLNTSTQVGYAVSNGAGVQVTVIGRDLSKANVWPQR
PINTSVPLVYDTAAETNNYTYWTGN

>Dcar_GH32_4, ON703182

MKMRFLSLLLIVVTSLNPTYASTISLNILTRIQDLSQVNLTTLLDNSLFTMWRPTYHFASPN**SWMNDPCGP**LYDSAT
QTYHLYYQVQPGHVQWGNISWGHAKSKDMIFWEDVTSWRGYDYITLAPGVGNNQSVLGVFTGSTLPVTITGDSTNRT
ITAIYTSVKYLPISWNGPYLKGSETQSLAVSYDGGITYQQYANNPILASPPEGMDV**TGWRDPKFK**QWPEIDNVLYGS
NQGHYYMTVSSGVRGVGPRLLLYRAFANDLTNWTYLGPLVSVSGNFTLNEIWSGSL**GYNFEVSNA**FSILEKAADGGD
NKTMHSFAMLGTEGGNTTLHPSTHWSVWINGNISKTGNDSVTMNIIASGVADWGDTYALNSFHDPKGNRRIFYGWVM
EDNNNYGQRAFGYNGQITLPREVFVQVYQNIVNINQSFSRQGAWILVPNNGGTYTFKTLGVRPINEVTNLRQGSNFI
SVMSQTFNQTTEFASLNVFSSNFELKANIDISSNTTAGFVFRRSSNGLEYTTLIYDPASEYLILNRTCSSLITAFGH
NTIYAKHALLTTQIDENNIQKEFLSLHIFLDNSFLEIYANNRTVLATHIYPALSDSTGLGYTVGGQGRSVTFSNISI
WSNLKNAFPNRPINSSIALAADPNNVTAASSVLSCNFGSFNSFFFFAFFSLYVS

>Dcar_GH32_5, ON703183

MLCTKTYTIFGVTFTLKKKMFVKLIFCSLLLSLTLCSVLLRNQNFELSYDALSDWITNNSNIFHIESKNDQCCTSIN
GMYHLVSPVNGTGSIQTDNFIVSGLGWMDFLIAGSANPDLCRLELVLTENNIVIYSESGPGLWPSSSGNSKPSWRHT
RKFWNVSSYLGKTVYLRLIDYSTDGFIAIDDIHTEVSHLDILFNIRYLNYRPLYHFSPDT**MWMNDPNGL**VYYDQEYH
LFFQHYPYDKKAMNIHWGHAVSKDLITWTQLPIALPPEDAVAGIWSGSAFIADKINSVLSFYLGRTWTKYVNNPILP
MHSIDSKSTEENDNIPTLVH**PVFRDPKVF**YHYETESWILVVSGGTHVKFYRSDNLVNWKLMSEWGKEHYSKTH**PGVW
ECPDL**FKMETEQDKNVWVLIISINRNSVSGGSGMQYFVGTFDGVQFTLNSKSLNTSLWLDYGPDYYAGITYSDIPHY
DNRRIMISWMNNWLYGRDLPTSVWCGQMGIPRSLKLYIDHYTNSYRLSQQPVIELYHYQRKLISFHRQMIHQGSNLL
ENILGVTYIITASFNLNTSIFNCQTCELGFQFRKSSENMKEYKEYTTIRYVPYNNTIYFDRSQSGETRFHSGFRTNY
NITLDDEVFDKGFLKFQILVDRLSVELFINDGKYAFTALIFPDFNSQNMALFTNGADVMIDYTDILLL

>Dcar_GH32_6, ON703185

MLLFVSFMIIVGISQADDRFHPQYHLMPAN**DWLNDPNGP**VFYKGYYHMFFQYTPNSSTSGLKYWGHGYSEDMVHWTR
LPIAISPDQPYDINGIWTGSTTIVDDVPVIIYTGINENNQEVQCQARPADATDPTMTEWIKFPMNPLITNP**NGRDPA**
TAFQDDQNNYYLIYGFGTDELGGQAVLFTSRDFTNWTYLHPIHSNHY**DNFWECPDI**FNVSNRVVMKASLRGQDFWAV
GELDPVKKVFVPLAGDLGEYTQLVDQGKFYASKSFYDPIHDQQVIVGWISEDDNQGEQRGWQGLLSLPRSIFLSDDG
LQLRSHPIETLQILRDEKSHRSFHNIVLPSVVPFELVPDIKGNQIELIINWQFPGNQVNSYSYILYK

>Dcar_GH32_7, ON703189

MLLFATIIVSLGVSQADDPLRPQYHMMPPK**NWLNDPNGP**VYYNGYYHMFFQHNPNAAVWGDMHWGHCYSEDMVHWIH
LPVAIAPDQPYDKNGIFTGSTTLVDGIPVIIYTGITETNEQVQCQARPANLSDPTLTSWIKSPLNPIITHP**NGRDPA**
TAFEDDYNNYYLIYGFGTDELGGQAVLFTSKDFSNWTYLHPIHSNHY**DNFWECPDI**FNVSNRIVLKASLRGQDFWAI
GDFDPTNLIFVPVGNDLGEYTQLIDQGKFYASKSFYDPLNDQQVIMGWTAEDDNQGEQRGWQGLHTLPRAIFLSDDG
LQLRTRPIEALQSLRNEKTHRHFQNVILPTVIPFVLVPDVSGNQIEIIINWQFSMNQVDRLLSLTFISLFLLTEFRF
WHECSFNIRW

>Dcar_GH32_8, ON703192

MIILAGLLSVLCASYVLTGDQFRPQFHLMPEK**NWLNDPNGP**MYFNGYYHMFYQYNPNAPVPDTMYWGHCFSTDMVHW
IRLPVALAPDHSYDQGGCWTGSATIVNGVPTIIYTGINSNGQQVQNQAFPADLTDPTLTIWTKSSLNPLITS**PNGRD**
PSTGFQLHDGYYYLIYGFGTEDLGGQAVLFRSQNFKNWTYMHPFHSNHY**DSFWECPDI**FNISSSNQLVLKASLLGHD
FWATGSFDFETQTFVPTGTGDVGEFSQLIDQGRFYASKTFYDPIDDQQVIFGWVAEEDNHGADRGWQGCHSLPRRIE
LSDDGQEIRSHPIDNLKSLRDKSSHKKFINIPLSSSTPFRLIDGLIGNQIELLINWQFPMYQVIFLLFDNQTS

Rotaria sordida

>Rsor_GH32_1, CAF1504698 (E in active site region 3 substituted to K, but there is an adjacent D)

MNDPNGLVYYDGEWHLFYQHYPYESKPLNIHWGHAISKDLITWIELPIAIKPENKDVGIWSGSVIIDWKNITGFQNE
TTIHPMIAIYTWQKLKWQEQHMAYSLDRGRTWTKYHSNPIVSLNDSISKPNNMNIQSSE**IVFRDPKVI**FHTSTASWI
LVLTGGNHIQFYKSIDLIHWSLVSRFGYEYNSH**SSIWKCPDL**IEFPLTKAKKKLNLWILIVSRQTSASIGDSAMQYF
IGTFDGTTFRNLQLTQTINRLDYGPDFYAGIIYHNISQYDGRQIIISWMNNWQYAQDVPTGPLWRGQMTIPRQLKLD
FNSFTNNYHLRQLPVHELYSYSKKLLTFHHQILTSNTTNILSNISSHVFMLSTEFHNITKTTVIRLYVRQSLDKSEY
TEIKYVGKNNQIEFDRSHSGNINFHSGFFTKFNITLDDETLTMGILQFQIIVDRCSVEIFVNGGKYTMTTLIFPKFE
SQRMELSVEGQHILLNYLELMFL

Rotaria sp. Silwood1

>CAF3679761

MNDPNGLVHYDGEWHLFYQHYPHQPRPMNIHWGHAVSKDLITWIDLPIAIKPEDEKVGIWSGSVVIDLKNVTGYQRE
TTIHTMIAIYTCAKLQWQEQHMAYSLDRGRTWTKYYLNPIITLNSSISKSDIVNIESMKTDFRDPKVMFHEATSSWI
LVLTGGNHVQFYKSTDLIHWTLTSRFGYEHSIESAFFHPHRPLIHFSPKR**NWMNDPNGL**VHYDGEWHLFYQHYPHQP
RPMNIHWGHAVSKDLITWIDLPIAIKPEDEKVGIWSGSVVIDLKNVTGYQRETTIHTMVAIYTCAKLQWQEQHMAYS
LDRGRTWTKYHLNPIITLNSSISKSDIVNIESMK**TDFRDPKVI**FYEATSSWILVLTGGNHVQFYKSTDLIHWTLTSR
FGYEHVSH**DGLWECPDL**IEFSSTRVKNQTSLWVLLVSVGNNAIAGGTGMRYFIGTFNGNTFQNLQSSDTINWLDYGP
DFYAGITYHNVPRYDGRQIIISWMNNWRYAQDLPTAPLWRGEMSIPRQLKLDYSSFTKKYFLRQLPVHEVYSYSRKV
FIFHRQVLNSSTSNILRNISSHAFIMLTEFHNITKDTVIRLHVRQSLDKREYTEIIYLGTTNEVEFDRTHSGNIKFH
SSFFKRFRAPLDDETLITGILKLQILVDRCSVEVFINGGTYTITALVFPQFASQQLELSVEGQPVILNYIELVLL

>CAF1243227

MNDPNGLVHYDGEWHLFYQHYPHQPRPMNIHWGHAVSKDLITWIDLPIAIKPEDEKVGIWSGSVVIDLKNVTGYQRE
TTIHTMIAIYTCAKLQWQEQHMAYSLDRGRTWTKYYLNPIITLNSSISKSDIVNIESMK**TDFRDPKVM**FHEATSSWI
LVLTGGNHVQFYKSTDLIHWTLTSRFGYEHVSH**DGLWECPDL**IEFSSTRVKNQTSLWVLLVSVGNNAIAGGTGMRYF
IGTFNGNTFQNLQSSDTINWLDYGPDFYAGITYHNVPRYDGRQIIISWMNNWRYAQDLPTAPLWRGEMSIPRQLKLD
YSSFTKKYFLRQLPVHEVYSYSRKVFIFHRQVLNSSTSNILRNISSHAFIMLTEFHNITKDTVIRLHVRQSLDKREY
TEIIYLGTTNEVEFDRTHSGNIKFHSSFFKRFRAPLDDETLITGILKLQILVDRCSVEVFINGGKYTITALVFPQFA
SQQLELSVEGQPVILNYIELVLL

>CAF3722074

MNDPNGLVHYDGEWHLFYQHYPHQPRPMNIHWGHAVSKDLITWIDLPIAIKPEDEKVGIWSGSVVIDLKNVTGYQRE
TTIHTMIAIYTCAKLQWQEQHMAYSLDRGRTWTKYYLNPIITLNSSISKSDIVNIESMK**TDFRDPKVM**FHEATSSWI
LVLTGGNHVQFYKSTDLIHWTLTSRFGYEHVSH**DGLWECPDL**IEFSSTRVKNQTSLWVLLVSVGNNAIAGGTGMRYF
IGTFNGNTFQNLQSSDTINWLDYGPDFYAGITYHNVPRYDGRQIIISWMNNWRYAQDLPTAPLWRGEMSIPRQLKLD
YSSFTKKYFLRQLPVHEVYSYSRKVFIFHRQVLNSSTSNILRNISSHAFIMLTEFHNITKDTVIRLHVRQSFDKREY
TEIIYLGTTNEVEFDRTHSGNIKFHSSFFKRFRAPLDDETLITGILKLQILVDRCSVEVFINGGKYTITALVFPQFA
SQQLELSVEGQPVILNYIELVLL

>CAF5081337

MNDPNGLVHYDGEWHLFYQHYPHQPRPMNIHWGHAVSKDLITWIDLPIAIKPEDEKVGIWSGSVVIDLKNVTGYQRE
TTIHTMIAIYTCAKLQWQEQHMAYSLDRGRTWTKYYLNPIITLNSSISKSDIVNIESMK**TDFRDPKVM**FHEATSSWI
LVLTGGNHVQFYKSTDLIHWTLTSRFGYEHVSH**DGLWECPDL**IEFSSTRVKNQTSLWVLLVSVGNNAIAGGTGMRYF
IGTFNGNTFQNLQSSDTINWLDYGPDFYAGITYHNVPRYDGRQIIISWMNNWRYAQDLPTAPLWRGEMSIPRQLKLD
YSSFTKKYFLRQLPVHEVYSYSRKVFIFHRQVLNSSTSNILRNISSHAFIMLTEFHNITKDTVIRLHVRQSFDKREY
TEIIYLGTTNEVEFDRTHSGNIKFHSSFFKRFRAPLDDETLITGILKLQILVDRCSVEVFINGGTYTITALVFPQFA
SQQLELSVEGQPVILNYIELVLL

>CAF3365478

MQAQTKQEYRPLVHYSPRM**NWMNDPNGL**IYYDGEWHLFYQYYPLEPQAKNIHWGHAISKDLIAWTELPIALTPDDED
VGIWSGSVVIDWNNVTGLQQTSTTHPMIAIYTWQKRGWQEQHMAYSLDRGRTWIKYPLNPIIPLDASIMREDNVKLN
PSE**VVFRDPKVL**FHVPTGSWILVISGGDHVQFYTSNDLIHWLLSSRFGYKDGCH**AGVWECPDL**VEFSSTTQDNQKLW
LLIVSIDRNAVAEGSGMQYFVGTFDGHTFKNLQTANTICWLDYGPDFYAGITYFNIPRFDARQIMIAWMNSWQYAQS
LPTAPVWRGQMAIPRRIELDCDVFTNVYYAAA

Rotaria sp. Silwood2

>CAF2743690

MNDPNGLIYYDGEWHLFYQHYPQQPKPMNIHWGHAVSKDLITWIDLPIAIKPEDEEVGIWSGSVVIDSKNVTGCQKE
TTIHTMIAIYTWKKLQCQEQHMAYSLDRGRTWTKYHLNPIITLNSSISKSDIVNIQSMK**ADFRDPKVI**FHEATSSWI
LVLTAGNHIQFYKSTDLIHWILTSRFGYEHVSH**GGVWECPDL**IEFLPTTVQNQTSLWILLVSVGNNAIAGGTGMRYF
IGTFNGNTFQNLQSSNTINWLDYGPDFYAGITYHNVPRYDGRQILISWMNNWQYAQDLPTAPLWRGQMSIPRQLNLA
YNSFTKKYLLQQLPVYEVYSYSRKFFKFHRQILTSSTSNILRNISSHAFIMLTEFQNITKDTVIRLHVRQSFDKREY
TEIIYLGVTNQVEFDRTHSGNINFNSNFFKKFRASLDDETLITGILKLQLIVDRCSVEVFINGGKYTITALVFPKFA
SQQLELSVEGQPIALNYIELMIL

>CAF3250172

MNDPNGLIYYDGEWHLFYQHYPQQPKPMNIHWGHAVSKDLITWIDLPIAIKPEDEEVGIWSGSVVIDSKNVTGCQKE
TTIHTMIAIYTWKKLQCQEQHMAYSLDRGRTWTKYHLNPIITLNSSISKSDIVNIQSMK**ADFRDPKVI**FHEATSSWI
LVLTAGNHIQFYKSTDLIHWILTSRFGYEHVSH**GGLWECPDL**IEFSPTTVQNQTSLWILLVSVGNNAIAGGTGMRYF
IGTFNGNTFQNLQSSNTINWLDYGPDFYAGITYHNVPRYDGRQILISWMNNWQYAQDLPTAPLWRGQMSIPRQLNLT
YNSFTKKYLLQQLPVHEVYSYSRKFFKFHRQILTSSTSNILRNISSHTFIMLTEFQNITKDTVIRLHVRQSFDKREY
TEIMYLGATNQVEFDRTHSGNINFNSNFLKKFRASLDDETLITGILKLQLIVDLCSVEIFINGGKYTITALVFPKFS
SQQLELSVEGQPVALNYIELVIL

>CAF4582490

MNDPNGLIYYENEWHLFYQHYPLEPQAKNIHWGHAVSRDMIAWTDLPIALTPDDENTGIWSGSAVIDWNNVTGLQQI
STVHPMIAIYTWQKRGWQEQHMAYSLDRGRTWTKYPLNPIIPLVADITQQDNVELNPSE**IVFRDPKVL**FHRPTGSWV
LVISGGNHVQFYTSNDLIHWSLSSRFGHEDGSH**AGVWECPDL**IEFSATTSDNQKLWLLIVSINRNAVAGGSGMQYFV
GTFDGRTFKNLQTANTIRWLDYGPDFYAGVTYHNVPRFDARQIMVAWMNNWQYAQSLPTAPIWRGQMTVPRQIELSF
DALTNAYHVRQSPVHELYSHSKKIITLSRQNLTSETPNILATMCSDTFMIWAEFHRVTPTTVVILQVRKSSDGSEYT
EIKYMADKNRLELDRSHS

>CAF3200955

MNDPNGLIYYENEWHLFYQHYPLEPQAKNIHWGHAVSRDMIAWTDLPIALTPDDENTGIWSGSAVIDWNNVTGLQQI
STVHPMIAIYTWQKRGWQEQHMAYSLDRGRTWTKYPLNPIIPLVADITQQDNVELNPSE**IVFRDPKVL**FHRPTGSWV
LVISGGNHVQFYTSNDLIHWSLSSRFGHEDGSH**AGVWECPDL**IEFSATTSDNQKLWLLIVSINRNAVAGGSGMQYFV
GTFDGRTFKNLQTANTIRWLDYGPDFYAGVTYHNVPRFDARQIMVAWMNNWQYAQSLPTAPIWRGQMTVPRQIELSF
DALTNAYHVRQSPVHELYSHSKKIITLSRQNLTSETPNILATMCSDTFMIWAEFHRVTPTTVVILQVRKSSDGSEYT
EIKYMADKNRLELDRSHSEQRPWSEWSRSDESYLQHTGHDVEQVQHLYSMCEQPFVNYCRHRNQEDPNSNITPYLSP
MNLLVVTLWYLKHYHSERYIAAEFDLSRSRVNYFLSTVVDILHCCVYPELVSLPADMSNRTAAHGPEEHHKLIVDSN
FIAIPQPDDINQRKAYYHAKSPTNYALKIQISCDFRHRIVHVFERYHGSVHDITILRESGLLEHVNDSVQIIADKGY
IGEEYVVTPRKKPHGRELTDEDKNFNRDINSARAAIENIDQRLKTYSILGSVYRGAIDDLHKITKIAQVVSALCNMN
LNKHPIRK

>CAF2941950

MNDPNGLIYYENEWHLFYQHYPLEPQAKNIHWGHAVSRDMIAWTDLPIALTPDDENTGIWSGSAVIDWNNVTGLQQI
STVHPMIAIYTWQKRGWQEQHMAYSLDRGRTWTKYPLNPIIPLVADITQQDNVELNPSE**IVFRDPKVL**FHRPTGSWV
LVISGGNHVQFYTSNDLIHWSLSSRFGHEDGSH**AGVWECPDL**IEFSATTSDNQKLWLLIVSINRNAVAGGSGMQYFV
GTFDGRTFKNLQTANTIRWLDYGPDFYAGVTYHNVPRFDARQIMVAWMNNWQYAQSLPTAPIWRGQMTVPRQIELSF
DALTNAYHVRQSPVHELYSHSKKIITLSRQNLTSETPNILATMCSDTFMIWAEFHRVTPTTVVILQVRKSSDGSEYT
EIKYMADKNRLELDRSHSEQRPWSEWSRSDESYLQHTGHDVEQVQHLYSMCEQPFVNYCRHRNQEDPNSNITPYLSP
MNLLVVTLWYLKHYHSERYIAAEFDLSRSRVNYFLSTVVDILHSCVYPELVSLPADMSNRTAAHGPEEHHKLIVDSN
FIAIPQPDDINQRKAYYHAKSPTNYALKIQISCDFRHRIVHVFERYHGSVHDITILRESGLLEHVNDSVQIIADKGY
IGEEYVVTPRKKPHGRELTDEDKNFNRDINSARAAIENIDQRLKTYSILGSVYRGAIDDLHKITKIAQVVSALCNMN
LNKHPIRK

Flatworm (Seriata, Rhabditophora, Platyhelminthes)

Schmidtea mediterranea

>Smed_GH32_1, AUVC01002567

MNFKLFLTLIIVITIYSCNNNTQNSTKEEHRLGFHFTPKA**NWMNDPNGL**VYYNGEYHLFYQYYPDSTVWGPMHWGHA
VSTNLNTWQDLPIALFPDSIGYIFSGSIVIDSNNTSGFKNGNELPMVAIYAYHNPILEKQNSLNTQTQGIAYSNDKG
RSWKKYNLNPVLKNGKY**KDFRDPKVF**WYELEKKWVMSLAVGNRIQFYSSKNLKEWQLTGEFGEDNQGSH**NGVWECPD**
LIKIKIKNTNQTKWVLLVSINPGGLRGGSGTQYFIGDFNGKTFKNENKKDKIMWLDYGADNYAGVTWNNTPNDTILF
LGWMSNWLYAQKVPTEKWRSAMTIPRILTLQNTNNGLILSNNPSSQINNLRINKNNILENKLFKTQTALNEINLSIN
LKETTSNEFGVEISNDLNEKLIVGFDKTNNQLFIDRTNAGKNKFDTNFASKHYATRIINDSILNLKIFIDVASVEVF
CDEGTLTMTEIFFPNEEMKNIKLFSKNGKIDLLQNEIYSLKNTLNKN

Segmented worms (Canalipalpata, Polychaeta, Annelida)

Streblospio benedicti

>Sben_GH32_1, JAGTTF010000008
MSSYKEQYRPQFHFSPER**NWINDPNGL**VYHKGEYHLFYQYNPYGSTWGHMSWGHAVSNDLLHWKHLDVALLEEDEIM
IFSGSAIIDRENSSGLGSAQCPPMIAFYTGHKIEKDRGYGIGNHISYATILGSIPIDSTVNMFCKFNSIKCHYFSQI
QDQRLAFSLDDGRSWTKYSGNPVLDLGK**ADFRDPWIM**WHEPSKKWNMLVVDALKKKVMFFSSKNLIEWKFMSDFGPA
GITE**TREWECPVL**LEMTTDDSKKKWVLMISCTPNKMQYFVGTFDHNFTFMLTAVYYYIWRYANDIPTNPWRGSMSIP
REITLVKKENQYILRQKPVEEINSLRKKSQTFSKIPTKQVRKSEICF

Plants

>Taes_GH32_1
MEARDGGSAPLPCSYAPLPEDAEAATTVGRAHRTAGPLCAALLLATAVVLLVVAALAGVRVNGQLPAGGIVVMSDHQ
TTVDAAPMSTSSRGPDSGVSEKTSGAGAHGGMLGADAGGNAFPWSNAMLQWQRTGFHFQPEK**NWMNDPNGP**VYYKGW
YHLFYQYNPDSAIWGNKIAWGHAASRDLVRWRHLPVAMSPDQWYDINGVWSGSATVLPDGRIVMLYTGSTNASVQVQ
CLAFPTDPSDPLLINWTKYENNPVMYPPPGVGE**KDFRDPTTA**WFDGSDDTWRLVIGSKDDRHAGMVMTYKTNDFINY
ELVPGLLHRVPG**TGMWECIDL**YPVGGKRGIDMTEVVAAASTNGGDDVLHVMKESSDDDRHDYYALGRYDAAKNTWTP
LDADADVGIGLRYDWGKFYASKTFYDPAKKRRVLWGWVGETDSEHADVAKGWASLQSIPRTVVLDTKTGSNLLQWPV
EEVETLRTNSTNLGGVTVDHGSVFPLSLHRATQLDIEASFRLDQLDVAASKEADVGYNCSTSGGTTGRGTLGPFGLL
VLADARRHGGDMERTGVYFYVARSLDGGLRTHFCHDEMRSSHANDIVKRVVGNIVPVLNGEEFTMRVLVDHSIVESF
AMGGRLTATSRVYPTEAIYANAGVYLFNNATSARVNVTRLVVHEMDSSYNQAYMASL
>Taes_GH32_2
MDTGDSGSVPCSYAQLPDDAEAGRTRPGRTGPLCAAVLLTSAAVLLAVAALAGVRLAGQLPAAGVIMSGHPTTVDMA
PMSTSSRGPESGVSEKTSGAATVDVHGSMLAADAGGNAFPWSNAMLQWQRTGFHFQPEK**NWMNDPNGP**VYYKGWYHL
FYQYNPDGAIWGNKIAWGHAASRDLLRWRHLPVAMSPDQWYDINGVWSGSATVLPDGRIVILYTGSTNASVQVQCLA
FPTDPSDPLLINWTKYENNPVMYPPPGVGE**KDFRDPTTA**WFDGSDDTWRLIIGSKDDRHAGMVMTYKTKDFMEYELV
HGLLHQVPG**TGMWECIDL**YPVGGVRGIDMTDAVAASSTNGGDDVLHVMKESSDDDRHDYYALGRYDGAKNTWTPLDV
DADLGIGLRYDWGKFYASKTFYDPAKKRRVLWGWVGETDSERADVAKGWASLQSAPRMVVLDTKTGSNLLQWPVEEV
ETLRTNSTDLGGVTIDCGSVFPLSLHRATQLDIEATFRLDPLDIAVANEADVGYNCSTSGGAAGRGMLGPFGLLVLA
DARRHGGDAERTAVYFYVARGLDGGLNTHFCHDETRSSCANDIVKRVVGHTVPVLDGEELSVRVLVDHSIVESFAMG
GRLTATSRVYPTEAIYANAGVYLFNNATGARVTTTSLVIHEMDSSYNQAYMASL
>Taes_GH32_3
METRDRGSMPALVPCFYVQLPLDDDEAQAGRARPGRTGPVCAAMLLTLAAVLLAVAALAGVKLASELPASGIVMSGH
PTEVDAAPMSTSSRGPESGVSEKTSGAGAHGGMLGADAGGNAFPWSNAMLQWQRTGFHFQPEK**NWMNDPNGP**VYYKG
WYHLFYQYNPDGAIWGNKIAWGHAASRDLLHWRHLPVAMSPDQWYDMSGVWSGSATVLPDGRIVMLYTGSTNASVQV
QCLAFPTDPSDPLLINWTKYENNPVMYPPPGIGE**KDFRDPTTA**WFDGSDDTWRLVIGSKDDRHAGMVMTYKTKDFID
YELVPGLLHRVPG**TGMWECIDL**YPVGGARGIDMTDAVEAGSTNGGDDVLHVMKESSDDDRHDYYALGRYDAAKNIWT
PLDSDADVGVGLRYDWGKFYASKTFYDPAKKRRVLWGWVGETDSERADVAKGWASLQSIPRTVVLDTKTGSNLLQWP

VQEVETLRTNSTDLGRVTIDHGSVFPLSLHRATQLDIEASFRIDPLDIAATKEADIGYNCSTSGGAAGRGALGPFGL
LVLADARHHGGDAERTAVYFYVARGLDGGLRTHFCHDETRSSRANDIVKRVVGNTVPVLNGEDLSVRVLVDHSIVES
FAMEGRSTVTSRVYPTEAIYANAGVYLFNNATGAQVTATSLVVHEMDSSYNQAYMASM
>Atha_GH32_1
MADVMEQNLLQTAVLNRTSFHFQPQR**NWLNDPNAP**MYYKGFYHLFYQNNPLAPEFSRTRIIWGHSVSQDMVNWIQLE
PALVPSESFDINSCWSGSATILPDGRPVILYTGLDVNNKQQVTVVAEPKDVSDPLLREWLKPKYNPVMVPPSNVPF**N**
CFRDPTEAWKGQDGKWRVLIGAKEKDTEKGMAILYGSDDFVQWTKYPVPLLESEG**TGMWECPDF**FPVSITGKEGVDT
SVNNASVRHVLKASFGGNDCYVIGKYSSETEDFSADYEFTNTSADLRYDHGTFYASKAFFDSVKNRRINWGWVIETD
SKEDDFKKGWAGLMTLPREIWLDTSGKKLMQWPIEEINNLRTKSVSLDDCYEFKTGSTFEISGITAAQADVEVTFNL
PFLEDNPEILDADQVDDATLFDRDSSVGCVYGPFGLLALASSDLSEQTAIFFKVIRRGNGYAVVMCSSEKRSSLRDN
IKKSSHGAFLDIDTRHEKISLRCLIDHSIIESYGVGGKTVITSRVYPKLAIGEAAKLYVFNDGENGVIMTSLEAWSM
RNAQINSNPTY
>Atha_GH32_2 (**D in active site region 1 substituted to G**)
MAKLNRSNIGLSLLLSMFLANFITDLEASSHQDLNQPYRTGYHFQPLK**NWMNGPMIY**KGIYHLFYQYNPYGAVWDVR
IVWGHSTSVDLVNWISQPPAFNPSQPSDINGCWSGSVTILPNGKPVILYTGIDQNKGQVQNVAVPVNISDPYLREWS
KPPQNPLMTTNAVNGINP**DRFRDPTTAW**LGRDGEWRVIVGSSTDDRRGLAILYKSRDFFNWTQSMKPLHYEDL**TGMW**
ECPDFFPVSITGSDGVETSSVGENGIKHVLKVSLIETLHDYYTIGSYDREKDVYVPDLGFVQNESAPRLDYGKYYAS
KTFYDDVKKRRILWGWVNESSPAKDDIEKGWSGLQSFPRKIWLDESGKELLQWPIEEIETLRGQQVNWQKKVLKAGS
TLQVHGVTAAQADVEVSFKVKELEKADVIEPSWTDPQKICSQGDLSVMSGLGPFGLMVLASNDMEEYTSVYFRIFKS
NDDTNKKTKYVVLMCSDQSRSSLNDENDKSTFGAFVAIDPSHQTISLRTLIDHSIVESYGGGGRTCITSRVYPKLAI
GENANLFVFNKGTQSVDILTLSAWSLKSAQINGDLMSPFIEREESRSPNHQF
>Atha_GH32_3
MAKLNRSNIGLSLLLSMFLANFITDLEASSHQDLNQPYRTGYHFQPLK**NWMNDPNGP**MIYKGIYHLFYQYNPYGAVW
DVRIVWGHSTSVDLVNWISQPPAFNPSQPSDINGCWSGSVTILPNGKPVILYTGIDQNKGQVQNVAVPVNISDPYLR
EWSKPPQNPLMTTNAVNGINP**DRFRDPTTAW**LGRDGEWRVIVGSSTDDRRGLAILYKSRDFFNWTQSMKPLHYEDL**T**
GMWECPDFFPVSITGSDGVETSSVGENGIKHVLKVSLIETLHDYYTIGSYDREKDVYVPDLGFVQNESAPRLDYGKY
YASKTFYDDVKKRRILWGWVNESSPAKDDIEKGWSGLQSFPRKIWLDESGKELLQWPIEEIETLRGQQVNWQKKVLK
AGSTLQVHGVTAAQADVEVSFKVKELEKADVIEPSWTDPQKICSQGDLSVMSGLGPFGLMVLASNDMEEYTSVYFRI
FKSNDDTNKKTKYVVLMCSDQSRSSLNDENDKSTFGAFVAIDPSHQTISLRTLIDHSIVESYGGGGRTCITSRVYPK
LAIGENANLFVFNKGTQSVDILTLSAWSLKSAQINGDLMSPFIEREESRSPNHQF
>Atha_GH32_4 (**D in active site region 1 substituted to G**)
MTKEVCSNIGLWLLLTLLIGNYVVNLEASHHVYKRLTQSTNTKSPSGNQPYRTGFHFQPPK**NWMNGPMIY**KGIYHLF
YQWNPKGAVWGNIVWAHSTSTDLINWDPHPPAIFPSAPFDINGCWSGSATILPNGKPVILYTGIDPKNQQVQNIAEP
KNLSDPYLREWKKSPLNPLMAPDAVNGINA**SSFRDPTTA**WLGQDKKWRVIIGSKIHRRGLAITYTSKDFLKWEKSPE
PLHYDDG**SGMWECPDF**FPVTRFGSNGVETSSFGEPNEILKHVLKISLDDTKHDYYTIGTYDRVKDKFVPDNGFKMDG
TAPRYDYGKYYASKTFFDSAKNRRILWGWTNESSSVEDDVEKGWSGIQTIPRKIWLDRSGKQLIQWPVREVERLRTK
QVKNLRNKVLKSGSRLEVYGVTAAQADVEVLFKVRDLEKADVIEPSWTDPQLICSKMNVSVKSGLGPFGLMVLASKN
LEEYTSVYFRIFKARQNSNKYVVLMCSDQSRSSLKEDNDKTTYGAFVDINPHQPLSLRALIDHSVVESFGGKGRACI
TSRVYPKLAIGKSSHLFAFNYGYQSVDVLNLNAWSMNSAQIS

Fungi

>Kmar_GH32_1
MKLAYSLLLPLAGVSASVINYKRDGDSKAITNTTFSLNRPSVHFTPSH**GWMNDPNGL**WYDAKEEDWHLYYQYNPAAT
IWGTPLYWGHAVSKDLTSWTDYGASLGPGSDDAGAFSGSMVIDYNNTSGFFNSSVDPRQRAVAVWTLSKGPSQAQHI
SYSLDGGYTFQHYSDNAVLDINS**SNFRDPKVFW**HEGENGEDGRWIMAVAESQVFSVLFYSSPNLKNWTLESNFTHHG
WT**GTQYECPGL**VKVPYDSVADSSSNSSDSKPDSAWVLFVSINPGGPLGGSVTQYFVGDFNGTHFTPIDDQTRFLDMG
KDYYALQTFFNTPNEKDVYGIAWASNWQYAQQAPTDPWRSSMSLVRQFTLKDFSTNPNSADVVLNSQPVLNYDALRK
NGTTYSITNYTVTSENGKKIKLDNPSGSLEFHLEYVFNGSPDIKSNVFADLSLYFKGNNDDNEYLRLGYETNGGAFF
LDRGHTKIPFVKENLFFNHQLAVTNPVSNYTTNVFDVYGVIDKNIIELYFDNGNVVSTNTFFFSTNNVIGEIDIKSP
YDKAYTINSFNVTQFNV
>Fsar_GH32_1
MLWKHSLKSMLPNFHTKLSGLALVFLLACLAQAEDFRPLYHFVPDQ**NWMNEPNGL**IKIGSKWHLFFQHNPTGNFWGN
LSWGHATSTDLIDWTHLPVAISSADGVEAFTGTAYLDEDNTSGLGTSDSPPYLAFYTGYFPDSGVQDQRLAYSLDQG

ETWTKYSENPIISQAQEKPHDTTGG**LETRDPKVF**FHTPTRTWVMVLAHGGQDKLSFWTSPDAKTWTWNNDLTSSDIS
GLSSD**ITGWEVPDL**FELTVEGTFDTKWVLLVTPAQGSPAGGNGVFAVTGSFDGTTFSPDAIDSTNMWLDFGRDWDGA
YSWENVPSSDGRKVLASVMNSYGADPPTETWKGMLSFPRTLELHEIDNELRFVQQPVKELDSASTSLVAVANKTLET
GDTLFSDQSGVALDIRVSFIPTEGSVLSLAVRKGDLEQTVIKYSQSDNTLSVDRTKSGNISYNSAAGGVHKATFSAD
SNGVVHLRVLVDVCSVEVFGGAGEVVISDLIFPSESSDGLELSATGGSVMLKSAEVRSVE

>Gste_GH32_1

MWSAIPFLGVALLTFSAAYAQGLDDTTIEAMGNNSLFTRWRPRSHFIAPA**GWMNDPCGS**MYDPHRDIYHLFYQWHPN
HINWGNISWGHATSKDLITWTDVGGWQDQQAQALGTGSVGTYNGLGIFSGTAQPVNIHGEQDGTLLAFYTSVSELPT
GYAIPYMKGTESQSLAFSTDGGITWEEYQDNPIITNPPDGWNI**TGWRDPFFE**PWPAMDALLGQSTPHYYAVFGSGIK
SVGPRIPFYSAPASDLTKWTFLGALWEPADNTSLGTVLETGSY**GFNFEVSGF**FSLEDSKGDLHYFVNMGTEGGNVSF
HESSHWALWNEGIVTRRANGSAQFTPISGGAGDWGLLYAMTSFNDTKHNRRVQWGWAPEDEGNFALTQQGYQGCFAL
PRELFVHTTTGLINADGQLITLGNSHVTEQSGGTFTATTLGNRPLPDVVVGIREGSTSKTYSMGKCSSSETLAAGSS
HMELVATFSNFSGAAGLTVAASPDGEEYTHIYFDPSSYTINVDRAHSSTITEFPNSTVVGYFYPYTSKTGGTESITM
NVFLDGSLLEVYVNDRFALTTRIYPSRTDSTSFGVFVASSANVNVVEVSTWVGLMNVFPDRPLNSSSKLVFDTAAET
NNYTWWTGS

>Dque_GH32_1

MGSMFTLGKVVRSVLIAALSLVSESKASAQDDVPTSLAGVDLSSLQNNTLFTRWRPTSHFIAPH**SWMNDPCGP**SYDH
SSGLYHLFYQFHPAHIAWGNISWGHATSEDLATWTDVEGWEAYSAVAIPPGPNGSIDHLGVFTGSAQFLTVTANQTW
GLPIQCDNEPLSDCGTQTVMLVFHTAVKHLPIGWSIPYTEGAETQALAISYDGGFTWSKFENTSINPVIATPPPDLD
V**TGFRDPFFE**LWPEMDSIIEADEPHFYAAFGSGIQHVGPRLQFYTAPVANLTHWSYLGPLFSVADNSSWSETYSGSY
GNNFEVAGTFSLPELVDNGGDGVTAHHFVMMGTEGEKNWPLWSEVDIVRSANGSAESIIRSSGVIDWGASYAWNSFY
DEPHDRRITFGWISEDLNTPALLPQGWAGATSLPREIYVQVYQNLANTNGVLSQPGSWTAVQDQDGRWNMTTLAMRP
APDVVAALRQNATMTHVDNIVQSASVAATSQFYSLGVSSDSVNLHAEIDIPVNTTASVGFVLRRSPDGEEQTVVIYD
PVHETLTINLTSSTLMNATFVNNADHIAPLFLLDTYNSTDASAVQTTRESLTLDIFLDNSIIEVFANSRAAMIGRVY
PARVDSLGIGYIVGPGSGQVVFKQIVVWEGLEKAWPGRPDNTSTQLVYDTSEETNNYTWWVGY

>Bbru_GH32_1

MTIKQDVTNLILSSDPLRPQAHFLAPY**GWMNDPCGP**IYAGGKYHLFYQWNKDAAKWGNIHWGHAISSDLIHWKHEST
ALFPQKNGADKDGVFTGDVVDVDGKTAIALYTGFRLDKPLKQVQCIATSDIEMIKWKQEPKPFLDVAPDGLEI**DGFR
DPRVW**RENGKYVMILGSSIKGKGGVVFRYEGTDLKHWTYKGVLYGPSKLRGTD**DDALECPDF**FPLGNRHVLIFSLNS
VVYAVIGEYKNAKFTPLSIEKYGHGNIYAARTFLDSAGQRVLFGWITERISQENEALARGWSGTMSFPRILYLTEDG
HVGSRIYPTVDSIEGELLIKETPLRIFSLFLDVPCTSRFHLKHGILEKFFFLELILLFV

Bacteria

>Bthe_GH32_1

MMKNMILPIAFTALIASMTACSDETDPILTQKNWDGTATYFQSSDEHGFSMYYKPQVGFVGDPMPFYDPVAKDFKVM
YLQDYRPNPEATYHPIFGVATKDGATYESLGELISCGGRDEQDAAIGTGGTIYNPADKLYYTFYTGNKFKPSSDQNA
QVVMVATSPDFKTWTKNRTFYLKGDTYGYDKNDFRDPFLFQTEDGVYHMLIATRKNGKGHIAEFTSADLKEWESAGT
FMTMMWDRFYECPDVFKMGDWWYLIYSEQASFMRKVQYFKGRTLEDLKATTANDAGIWPDNREGMLDSRAFYAGKTA
SDGTNRYIWGWCPTRAGNDNGNVGDVEPEWAGNLVAQRLIQHEDGTLTLGVPDAIDRKYTSAQEVKVMAKDGNMIES
GKTYTLGEGASVIFNRLKVHNKISFTVKTASNTDRFGISFVRGTDSASWYSIHVNADEGKANFEKDGDDAKYLFDNK
FNIPADNEYRVTIYSDQSVCVTYINDQLSFTNRIYQMQKNPWSLCCYKGEITVSDVQVSTY

>Blon_GH32_1

MTDFTPETPVLTPIRDHAAELAKAEAGVAEMAAKRNNRWYPKYHIASNGGWINDPNGLCFYKGRWHVFYQLHPYGTQ
WGPMHWGHVSSTDMLNWKREPIMFAPSLEQEKDGVFSGSAVIDDNGDLRFYYTGHRWANGHDNTGGDWQVQMTALPD
NDELTSATKQGMIIDCPTDKVDHHYRDPKVWKTGDTWYMTFGVSSADKRGQMWLFSSKDMVRWEYERVLFQHPDPDV
FMLECPDFFPIKDKDGNEKWVIGFSAMGSKPSGFMNRNVSNAGYMIGTWEPGGEFKPETEFRLWDCGHNYYAPQSFN
VDGRQIVYGWMSPFVQPIPMEDDGWCGQLTLPREITLGDDGDVVTAPVAEMEGLREDTLDHGSVTLDMDGEQIIADD
AEAVEIEMTIDLAASTAERAGLKIHATEDGAYTYVAYDGQIGRVVVDRQAMANGDRGYRAAPLTDAELASGKLDLRV
FVDRGSVEVYVNGGHQVLSSYSYASEGPRAIKLVAESGSLKVDSLKLHHMKSIGLE

>Tmar_GH32_1

MFKPNYHFFPITGWMNDPNGLIFWKGKYHMFYQYNPRKPEWGNICWGHAVSDDLVHWRHLPVALYPDDETHGVFSGS
AVEKDGKMFLVYTYYRDPTHNKGEKETQCVAMSENGLDFVKYDGNPVISKPPEEGTHAFRDPKVNRSNGEWRMVLGS

GKDEKIGRVLLYTSDDLFHWKYEGVIFEDETTKEIECPDLVRIGEKDILIYSITSTNSVLFSMGELKEGKLNVEKRG
LLDHGTDFYAAQTFFGTDRVVVIGWLQSWLRTGLYPTKREGWNGVMSLPRELYVENNELKVKPVDELLALRKRKVFE
TAKSGTFLLDVKENSYEIVCEFSGEIELRMGNESEEVVITKSRDELIVDTTRSGVSGGEVRKSTVEDEATNRIRAFL
DSCSVEFFFNDSIAFSFRIHPENVYNILSVKSNQVKLEVFELENIWL
>Mabs_GH32_1
MTPTPTWAAAGGFPWSNEMLQWQRSGYHFQTAKNYMSDPNGLMYYRGWYHMFYQYNPVGTDWDDGMEWGHAVSRNLV
QWRTLPIAMVADQWYDILGVLSGSMTVLPNGTVIMIYTGATNASAVEVQCIATPADPNDPLLRRWTKHPANPVIWSP
PGVGTKDFRDPMTAWYDESDDTWRTLLGSKDDHDGHHDGIAMMYKTKDFLNYELIPGILHRVVRTGEWECIDFYPVG
RRSSDNSSEMLHVLKASMDDERHDYYSLGTYDSAANTWTPIDPELDLGIGLRYDWGKFYASTSFYDPAKKRRVLMGY
VGEVDSKRADVVKGWASIQSVPRTVALDEKTRTNLLLWPVEEIETLRLNATELTDVTINTGSVIHIPLRQGTQLDIE
ASFHLDASAVAALNEADVGYNCSSSGGAVNRGALGPFGLLVLAAGDRRGEQTAVYFYVSRGLDGGLHTSFCQDELRS
SRAKDVTKRVIGSTVPVLDGEALSMRVLVDHSIVQGFAMGGRTTMTSRVYPMESYQEARVYLFNNATGASVTAERLV
VHEMDSAHNQLSNEDDGMYLQSSS
>Aalb_GH32_1
MQNHQENIERANKAIDKIKDQVSQHEWRLQYHVMPPANWMNDPNGFSFFRGEYHLFYQHHPYDPKWGPMHWGHVKSK
DLVNWEHLPIALAPSEPYDKDGCFSGSAIEKDDKLYLIYTGNVWIEDGPLDEDLTQVQAVAVSEDGIHFEKLKENPV
IAEIPDGDINIFHFRDPKVWKRGDTYYMVLGSKTKKNTGQVLLYRSEDLIRWDFVSIMAKGEGNFGFMWECPDLFEL
DGKDVLVMSPQGLEPEGDKYHNLHQAGYIMGQLNYNTGKFNHGALHLLDHGFDFYAPQTMVDDQGRRILVAWMDMWE
SEMPTQTHGYAGAMTIPRLVTRDKDNLRTVPVPELQTLRQHQVSYENVQVNGASSLNGVEGDCLELEIDIDPNEATT
FGLKLRVDDEVNQETKLLFDQQAGTVTLDRNKSGKGPGGFRRTTVNMAKNVHLQIFIDKSSVEIFINRGEKVMTSRI
FPTENATGISFFADQEMTIERLSKWELKQAIK
>Unia_GH32_1
MLTTNKLQLAQEAIEKAKQQMNSRYRLGYHIMAPANWINDPNGLVQYKGEYHVFYQHHPFDENWGPMHWGHVKSKDL
VHWEHLPIALAPTEAYEKDGCFSGSAVDDNGILTLIYTGNIFIDKEKDILDQSQCIATSTDGIHFTKDTANPVISKH
PEEGSGHFRDPKVWKYEDFWYMILGTRKEDTGKVVLYKSSDLREWQYIGVLAESDGSLGYMWECPDFFELGGKHVLL
FSPQGMEAQGELYNNIFQTGYIVGDYSYETNSFEADDFTELDNGHDFYAVQTFLDDKGRRIAIGWMDMWESIMPTKE
DGWCGALTLPRELTLDQNNKILMKPVEELSLLRTTEQKVCTNKHLAESYLVEANNDLMELKLDIDLTKTAAQTVGVK
IRGINQEETLIKYETKEQKLVLDVSKAGKSEDGVRGTTLKADTQLSLRVFIDRSSIEVFANDGQVTMTSRIYPNEAI
LGFELFAENGAAEVIDFTYWELKDIWE
>BRHF_GH32_1
MLTINRIKQAEEALKHAKNKINNRYRLGYHIMAPANWMNDPNGFAQYKGEYHAFYQCHPYDANWGPMHWGHVKSKDL
VHWEHLPVALAPGDACDTDGCFSGSAVDNNGELTLIYTGHHYTDKEKDLFHQNQNIAVSTDGISFEKVGENPVIAKP
PVDSAHHFRDPKVWRHNDTWYMVLGNSTKDNVGRVILYRSSDLLNWEYIGVLAQSDGSLGYMWECPDFFELDGKYIL
MISPQGIKSNGDLYNNLFQTGYLVGDYNYETNEFVHGSFTELDNGHDFYAVQTLLDDKGRRIAIGWMDMWESNMPTK
ADGWCGALTLPREITLGDHNKILMNPVEELILLRDSEHIECTNQSISESYMIETKEDLLEVKAVFDLTNCSAQSVGL
KIRGVNQEETVLTYHLDKQQLTLDCSKSGKVEDGVRSTMLETNKELSLRVFIDRSSIEIFANDGQTTMTSRIYPNED
RLGIELFTEKGDVQVKEITYWNLQDIWK
>Rban_GH32_1
MTSQASSPSDEARLETISAVLPAGTTIHAWLKASHGGAPARLTAHVDGGLAGAVETSNPDEFAFRPLTLERGGEAAF
SYNPATTQVSVLYAFLEPRVLEEGIRLLHARPANATPEVPGSYHFRPPFGWMNDPNGFGRFGGKLHLFYQHYPHSLR
WNNMHWGHAVSKDYLRWTHLPIFLFPSDELAARADGRGGAFSGSAIALPGDEAGLRIFFTEHMKDREPEEQVQFTAT
SRDLVNVEPASLILPARPTGLGLTTDFRDPYVFSGPDGKWKMLLGTRDREGGVILLYETDDPAAAAGWTFLGTLHRE
NRFGMTAAECPCMLPLDGPANDPATRWALIFGLLTSRDPATGRRNMTLATVGRFDGRGFSVEFEQELDFGTDAYAFQ
AFVDEGGPVGIAWLANWTDISKEIDMPTAMTLPRRLALQSDALITPPVSGVESLRRRQLDAKALLDGRTVDLANGSV
EILLTLRQAGNAFRLDLEHPEATVAVQLNDDGLSIPFSVANTKASPRYIAAGARPSTIRIFLDAGSIEVFADDGRWT
GTKRLPGFKGVSAVRLTAPEGNIIAAEIWQLGL
>Sval_GH32_1
MKKLRLSIILLAFVVQYSFGQASQADAQSERYRPQFHFSPKAHWINDPNGMVYYKGTYHLFCQYHPESSVWGPMHWI
HATSKDMVHWQEQPIALYPDSLGYIFSGSAVVDVNNTSGFGKNGQVPLVAIFTHHNSKLEKTQPEQVQYQSLAYSLD
EGKTWTKYAGNPVLKNPGITDFRDPKVRWYEPQKKWIMTLATKDRVTFYSSPDLKKWARESDFGSDAGAHGGVWECP
DLFPLKHNGKDVWVLIVNINPGGPNKGSAAQYFLGDFDGKNFNAYSKQTKWMDYGTDNYAGVTFSNTGNRVVLMGWM
NNWQYARDVPTPKWRGATTIPRELGLKEANKELYLTSTPIKELDVLDGKITSLTNVDVKGSSDLTAKTGGKYDTFKL

TLSAPATNDFSVVLANEQGNELVIGYDKSANTYYIDRTKSGKTDFEKDFGKRMTAPRLSTDKTISLTLFIDVASAEL
FADDGLSVMTGIFFPNQPLTKLSIKSATGTSISKLTYTKLSPSVQ
>Cfre_GH32_1
MTALLAAANEALNQQKDRINPRWYPRYHLAPPAGWMNDPNGLSWFDGYYHAFYQHYPWQPVWGPMHWGHARSRDMVN
WEHLPIALAPEGPEDKDGCFSGSAVVDGNKLALIYTGHKFDGEAKEENLYQVQCLATSTDGIHFERQGMILNTPRGV
HHFRDPKVWQEGDSWYMVVGARVDDVGEVQLYRSQDLQHWQFASTLAQADDGMGYMWECPDLFPLNDKLVLMFSPQG
IAADGYDYRNLFQSGYMVGEWQDNHQFHVTQPFQEMDHGHDFYAPQSFITPDGRRVVIGWLSMWESPMPEQADGWAG
MLTLPREVTLDADLRLRMNPVKELEALRGQLHVWPVSDLNNRTLMVEEQAHALEVELSLDIARSHAEQYGIALGDGL
RVFVDTQAQRLVLERRYPQHGLSGCRSIPLPANDVLDLHLFIDSSSVEVFVNHGEYTLSSRIYPEPDDRQLTLFSQN
GHAIFNNGHAWPLAAN
>Socc_GH32_1
MVQVLSVLVIPLLTLFFGYVASSSIDLSVDTSEYNRPLIHFTPEKGWMNDPNGLFYDKTAKLWHLYFQYNPNATAWG
QPLYWGHATSNDLVHWDEHEIAIGPEHDNEGIFSGSIVVDHNNTSGFFNSSIDPNQRIVAIYTNNIPDLQTQDIAFS
LDGGYTFTKYENNPVIDVSSNQFRDPKVFWHEDSNQWIMVVSKSQEYKIQIFGSANLKNWVLNSNFSSGYYGNQYEC
PGLIEVPIENSDKSKWVMFLAINPGSPLGGSINQYFVGDFDGFQFVPDDSQTRFVDIGKDFYAFQTFSEVEHGVLGL
AWASNWQYADQVPTNPWRSSTSLARNYTLRYVHTNAETKQLTLIQNPVLPDSINVVDKLKKKNVKLTNKKPIKTNFK
GSTGLFDFNITFKVLNLNVSPGKTHFDILINSQELNSSVDSIKIGFDSSQSSFYIDRHIPNVEFPRKQFFTDKLAAY
LEPLDYDQDLRVFSLYGIVDKNIIELYFNDGTVAMTNTFFMGEGKYPHDIQIVTDTEEPLFELESVIIRELNK
> Stel_GH32_1
MRKIPLSSLLLVVLSHLAYGQVKQTDTKPELYRPQYHFTPKAHWMNDPNGMVYYKGTYHLFYQYYPGGTVWGPMHWG
HTTSKDMVHWQEQPIALYPDSLGYIFSGSAVVDINNTSGFGKNGQVPLVAIFTHHNPKLEKLKPDKAQYQSLAYSLD
EGKTWTKYAGNPVLPNPGITDFRDPKVRWYEAQKKWIMTLATKDHITFYSSPNLKSWSKESEFGGDAGAHGGVWECP
DLIPLNHKGKTVWVLIVNINPGGPNGGSAAQYFVGDFDGKNFNAYAKETKWMDYGADNYAGVTFANTGNRTILMGWM
SNWQYATVVPTVAWRSATTVPRELGLKEVGRELLLTSKPIKELDALNEQRFSLQNLAVKDSYELTAKTNNNTGLFKL
ELSTQNTNDFSIVLTNEQGNEVLIGYDKRANQYYIDRSHSGKVDFEKGFGKRHTAPRLSTDKTISLTLMADVASVEL
FADNGLTIMTDIFFPDKPLNKLFIKSATGITIPKLTYTKLAASVF
>Ppsy_GH32_1
MKNKFIKPAIFLLCSLFSIGASAQSTESEKYRPAFHFSPKAHWMNDPNGMVYYKGVYHLFFQYHPGGTTWGPMHWGH
AVTRDLFNWEEKPIALYPDSLGMIFSGSTVIDKDNTAGFGKNAMVAIFTHHNQKIEEAKTGLHQYQSIAYSTDEGAH
WTKYKGNPVLPNPGITDFRDPKVMWFEQGKKWIMTLATKDRITFYSSPNLKTWKRESEFGANLGAHGGVWECPDLFP
LKSNGKTVWVLLVSINPGGPNGGSATQYFTGSFDGNKFTPNATKEKWVDYGTDNYAGVTWSNTGDRKIFLGWMNNWQ
YANQVPTKAWRGAMTIARELSLENVGNDYYLRSVPVKEIGPLLKSVYNRTNFSIGTELNLSSYIKSLNGKFKLDLGI
AKLEKVDIVLENRDKEQLIIGYDEKNDNYFIDRRSAGNTSFEKGFAKKHVAPRSSKSKNLKLSLILDETSVELFSDN
GLTVMTDIFFPHSYWDTLKIRTSSGVKLSDISVWAIDLKKSVDNRHKK
>Aarb_GH32_1
MKKLLFSIVLGAFLYSCNSKPSATENTRVSTEKTGETADSANVNDKYTAEQHRPQFHFSPPQMWMNDPNGMVYYAGE
YHLFYQHYPDSTIWGPMHWGHAVSKDMVHWQNLPIALYPDSLGYIFSGSAVVDENNTAGFQKGNEKTLVAIFTHHNP
KTQKQVQSLAYSNDKGRTWTKYANNPVLPNPGISDFRDPKVSWYAPEKKWVMTLAVKDRVHFYGSPDLKKWQLLSEF
GKGNVGAHGGVWECPDLFPLTVNGQQKWVLFVSINPGGPNGGSATQYFIGDWNGKEFKNSNPPQTTLWVDQGSDNYA
GVTWSNIPASDGRRLFMGWMSNWFYANQVPTQNWRSATTVARELTLQNTPAGIRLASTPVKELQQLWQETKTIKAQE
ISSTLDLSKEYNLTSPLIELNLNFDVAKSSELLLTFSNTKGEYVNIGYSVPAKQLFIDRTHAGKTNFEPRFAKKHVA
PLILQNGKLNLHLFLDVASVEVFANNGQLVMTDIFFPNEDFTQVKVSTKGAAQLLESQAYPLKSIWKEALKE
>Salb_GH32_1
MEEKRIRNAYRKLDDMSHKDLAELIDTVQESPWRQNYHVQPVTGLLSHPSGFVYHDGLYHLFYQWSPLDSHHGIEYW
YHVTSEDLAVFTNRGVKIRPDSIYDSHGASSGSAFVIDEQVHIFYTGRRILGKGAAVPYQMHAVMDDRLKVRKDVFP
LIDSVPEGFTESFKDPAIWEEADVFYMILGAQTITDYGRAAVYSTEASSAFEYRGMIRTDLEAFGFLWESPDFFTID
GVDVLAFCPHGIDRYQYSYWNAYQSGYIMGRLYRGTLVMDHGEFHEFDHGFDFYAPRTTVGKNGEQVLIGMMGIAGT
AYPTDTYHWANCMTLPRVVTLGNGRLRQNPISALEKLRHNEISAVGYITHHPRKMKDFYGESYELLVDIKENNATEI
YINLRTSRKEATSLIYNTEKRLLTLDIGFSGEQPLNVDGTKRSVVLEEDLNDLRVFVDVSSIEIFVNQGEAVMSSRI
FPSDRATGVELSTEIGDCFLSLTKYDLTSFEHEKIIYNP
>OCFH_GH32_1
MLYQEKHRPQLHFTPKENWINDPNGLVYYEGEYHLFYQRNPHSNDHADMHWGHATSTDLYHWEEHGIALAPDELGTI
FSGSAVVDKNNTSGLFESASGGLVAIFTHDGESQQQSIAYSEDKGRTWIKYEGNPVIPNTKIKDFRDPKVSWHEDSK

KWVMALACGDHIQFYNSPNLIDWTYLSSFGKEYPSHIGVWECPDLLYLPVEGSSKKEWVLIVSINADGPNGGSAVYY
FTGSFDGKVFHPNEKPEDALKWADAGKDFYAAVSWDNTEDTYWIGWMSNWQYAGVMPVSPWRSAMSLARKISLYEKD
GNLLLKQTPVIADNRKEAASYQDVTIQPDTAESYAISDTAQIDLRISEASSSTKWGVKFVTDQEEVFQLAIDTQENT
YTFYRTEGMIDFSEEFPEEIKGTLNGLDVQNLTAIVDRSSVELFLNNGLTVSTNLIYPQGTVTQLEVFAVNDVLQID
NVSWTQLKSIWN
>Oonc_GH32_1
MELITEWTTPLRYKPYHQWSKSYQDMLSSIIQKSDWKLAFHIQPKTGLLNDPNGFSFYNEKWHLFYQAYPFGPVHGV
KSWYHMVSDNLVDWEQHDYVLLPDSPYDSHGVYSGSAMTVEDKLFLMYTGNVRNEEWERHSYQLGAWMNTEMEVEKI
TEPLIDSPPAGYTHEFRDPQVFRYQNHYLMMIGAQTKNEKGAVLAYQSSNLTDWELLGELDYTTEEMGFMVECPNFV
LVDGQPVLLFCPQGLDKDISSYQNIYPNMYVIGSSFNKETVSIENTSDLINLDEGFDIYATQAFQAPDGRVLSVGWI
GLPEIDYPTFEEGWAHCLSIVKELSIKENHLYQKPVAEMQELRQNHQELQGSAQASDTLLSSPDSNVYELKLELNAG
TSGSLYLFADKQNKEGLKISFDAKHDTISMDRSKAGIPFGESYGSTRTAALKRQDKLTLHIFADRSVCEVFVNDGYR
VITSRVFVKDPQQTHIFLQGFEGDYTGDFWTLREMNE
>Yfre_GH32_1
MMTLRIQANNAVKTLKESINPRWYPRFHLAPQAGWMNDPNGLIWFDGYYHAFYQHHPYAPVWGPMHWGHARSRDMVH
WEHLPIALAPEGPEDKDGCFSGSAVSDGDKLALIYTGHKFHGTPCDENLYQVQCLATSHDGVNFERKGIVIETPEGV
HHFRDPKVWQEKEGWYMVVGARVADIGEVQLYQSDDLHHWHYVNTLGQSDEKMGYMWECPDFFPLADKHILMFSPQG
MAAEGYNYRNLFQSGYLVGKWQEDDAYQIEQRFREMDHGHDFYAPQSFVAPDGRRIVMGWLAMWESPMPEKADGWAG
MLSLPRELTLDKKGQLRMNPVKELELLRGDCRRWQVSELTHQTIVVEQGGQPIEIQLDLNIPLSTAEQFGLNIGQGA
RIYVDSQSRRLVVERHYPQHELSGYRSVPLPDEERLALRIYIDSSSVEVFVNHGEYTLSSRIYPEKDDRDLSLFSHN
GRAVVHEAHAWYLNA
>Ubre_GH32_1
MNELLDKANSHIAATRPLMNMQWYPTWHLAPAVGWMNDPNGLIWFGGMWHAFYQHHPYDATWGPMHWGHARSSDLIH
WEHLPVALAPVGDADKDGCFSGSAVDNDGELSLIYTGHVHDGDRASDKGLRQVQCLATSRDGVNFTAQGKILDAPDG
IQHFRDPKVWKMDDYWYMVVGLRVADRGEVRLWRSDDLHNWQPQGVLARSAPGESYMWECPDFFPLGDKWVLMFSPQ
GMKADGYHNRNLFQSGYLVGTWQPGGEFVIETPFIEIDAGHDFYAPQTLSGPDGRRLMLGWLDMWESPMPEKDHLWA
GMLSMPRELTLKPDNTLRVVPAAEVDASCREPHRQQDIELKNASMPLMQDCEAHYVTLDIDLRASDAEKYGLSLGGQ
ENIEQGLFIYVDNQSQRVVLERRYPQYGISGYRSVPVTSLDTLQLRVFFDRSSVEIFVNDGESCLSSRIYPLERQRM
LQLFAQNGGARFKRVSNGAIG
>Cmob_GH32_1
MKTANYTLENANEFIRQKEEDVITTYSPSYHLTAPVGWINDPNGFVYYEGEYHLFYQYYPYKTVWGPMHWGHAKSKD
LVTWENLPVALSPDQYYDEGGCFSGSAIEKDGKLYLMYTGHLPQAEEEQSRQVQCMAVSEDGIHFEKISQNPVLDEK
DLPDNARVQDFRDPKVFERNGVYYSIIASQTKELTGQILLYQSTDLIEWEFKSILLEGTKGQGIMWECPDLFELDGK
DVLITSPIQIPKSGNEFHNVSSCVAFIGKVDWEKGTFEVESMEEIDHGLDFYAPQTTIDEQGRRVMVAWMQMWGRNM
PTHTENHGWVGAMTLPRELRIKNGKLIQLPISEIANYYQKSAEIKEVVLVDETKEFEGVAGKVGVLELVVDLKEATR
FRIELRASATERTLLTYHTETNELEFDRFQSGIPISGEEAEPLNKRKVLCSLDEAKLKLKIFLDNSSIETFVNDGKE
TITSTIYPLSDESEKIYLEAIGKVRIDSLDMFEIDMV
>Cmob_GH32_2
MKTANYTLENANEFIRQKEKDVITTYSPGYHLTAPVGWINDPNGFVYYEGEYHLFYQYYPYKTVWGPMHWGHAKSKD
LVTWENLPVALSPDQYYDEGGCFSGSAIEKDGKLYLMYTGHLPQSDEEQSRQIQCMAVSEDGIHFEKINQNPVLDEK
DLPDNARVQDFRDPKVFERNGVYYSIIASQTKELTGQILLYQSTDLIEWEFKSILLEGTKDQGIMWECPDLFELDGK
DVLITSPIQIPKSGNEFHNVSSCVAFIGKVDWEKGTFEVESMEEIDHGLDFYAPQTTIDEQGRRVMVAWMQMWGRNM
PTHTENHGWVGAMTLPRELRIKDGKLIQLPISEIANYYQKSAEIKEVVLVDETKEFEGVAGKVGVLELIVDLKEATR
FRIELRASATERTLLTYHTETNELEFDRFQSGIPISGEEAEPLNKRKVLCSLDEGKLKLKILLDNSSIETFVNDGKE
TITSTIYPLSDESEKIYLEATGKVRIDSLDMYDIDML
>Ymas_GH32_1
MMTLRLQANNAVKTLKESINPRWYPRFHLAPQAGWMNDPNGLIWFDGYYHAFYQHHPYTPVWGPMHWGHARSRDMVH
WEHLPIALAPEGPEDKDGCFSGSAVSDGDKLALIYTGHKFHGTPCDENLYQVQCLATSHDGVNFERKGIVIDTPKGV
HHFRDPKVWQEKEGWYMVVGARVADIGEVQLYQSDDLHHWNYVNTLGQSDEKMGYMWECPDFFPLADKHILMFSPQG
IEAEGYDYRNLFQSGYLVGKWQEDDSYQIEQGFREMDHGHDFYAPQSFVAPDGRRIVMGWLAMWESPMPEKADGWAG
MLSLPRELTLDKKGQLRMNPVKELELLRGDCRRWQVSELSHQTIVVEQGGQPIEIQLDLNIPLSTAEQFGLNIGQGA
RIYVDSQSRRLVVERHYPQHELSGYRSVSLPDEERLTLRIFIDSSSVEVFVNHGEYTLSSRIYPEKDDRDLSLFSHN
GRAVVHEAHAWHLNA

>Halv_GH32_1
MTLHIADAEQELAQKSDQLNTRWYPYYHLAARAGWMNDPNGLVWFDGWFHAFYQHHPYSTEWGPMHWGHARSRDLMR
WEHLPVALAPEGPEDKDGCFSGSAVVNENELALIYTGHKFHGDPSSEDNLYQVQCLATSRDGINFTRKGQVLDTPSG
LHHFRDPKVWKEGDMWYMVVGARVEDTGQVRLYRSEDLHRWDEQGILAEAPHGMGYMWECPDFFTLDDKQVLMFSPQ
GIAAEGYANRNLFQSGYIVGRWQPGEKFIQESKFQELDHGHDFYAPQSFLAPDGRRIVIGWMDMWESPLPEQQDGWA
GMFTLPRELVITSNNQLQMRPVHEVESLREEWYSWPVSTLKNKQLCVMHNCEAAEVIITWDTALSEAEQYGIALDDG
MRLYIDNQAKRLILERNYPQYNLCGQRSIPMPTGDELNLRVFFDRSSVEVFVNDGEACLSSRIYPQQRQLNVFAWSG
NAVLKECGAWNLR
>Cfre_GH32_2
MTALLAAANEALNQQKDRINPRWYPRYHLAPPAGWMNDPNGLSWFDGYYHAFYQHYPWQPVWGPMHWGHARSRDMVN
WEHLPIALAPEGPEDKDGCFSGSAVVDGNKLALIYTGHKFDGEAKEENLYQVQCLATSTDGIHFERQGMILNTPRGV
HHFRDPKVWQEGDSWYMVVGARVDDVGEVQLYRSQDLQHWQFASTLAQADDGMGYMWECPDLFPLNDKLVLMFSPQG
IAADGYDYRNLFQSGYMVGEWQDNHQFHVTQPFQEMDHGHDFYAPQSFITPDGRRVVIGWLSMWESPMPEQADGWAG
MLTLPREVTLDADLRLRMNPVKELEALRGQLHVWPVSDLNNRTLMVEEQAHALEVELSLDIARSHAEQYGIALGDGL
RVFVDTQAQRLVLERRYPQHGLSGCRSIPLPANDVLDLHLFIDSSSVEVFVNHGEYTLSSRIYPEPDDRQLTLFSQN
GHAIFNNGHAWPLAAN
>Bbre_GH32_1
MTLLIANAEQELNAKRERMNMRWYPHYHLAARAGWMNDPNGLVWFDGWFHAFYQHHPYSTQWGPMHWGHARSRDLLH
WEHLPVALAPEGPEDKDGCFSGSAVVNGDELALVYTGHKYHGDPASDDNLYQVQCLATSKDGIGFTRHGQVIDTPAG
LHHFRDPKVWREGETWYMVVGARVGDTGQVRLYRSDDLHHWEDAGLLAEAQEGMGFMWECPDFFTLNGKQVLMFSPQ
GMEARGYDNRNLFQSGYLVGQWQPGEAFSQQSAFQEMDHGHDFYAPQSFLTPDGRRIVIGWMAMWESPLPEQQDGWA
GMLTLPRELTLTADNRLQMRPARELESLRRSWQNWPVNTLKNKALRVMDECETTEVILEWDLSASNAEQFGIALEEG
MRLYVDAQAQRLVLERHYPQLGLCGQRSVPLPQSTTLSLRVFFDSSSVEVFVNDGEACLSSRIYPESRKLDIFAWSG
EAVLKKCGAWHLE
>Bsia_GH32_1
MDRIQQAEEALKEAEGKVKQRYRLGYHIMPRANWINDPNGLIQFKGEYHVFFQHHPYDENWGPMHWGHVKSKDLIHW
EHLPVALAPGDAFDQSGCFSGSAADDNGRLTLIYTGHNMIDQEKDLFYQTQNIAVSQDGIVFEKLQENPVIAAPPED
SARHFRDPKVWKHRDDWYMVIGNSSKENVGRVVLYRSSDLREWEYAGVLAQSDGHLGYMWECPDFFELGGKHVLLIS
PQGIEADGDSYNNLHQTGYLIGDYNYETNKFTHGAFTELDYGHDFYAVQTLLDDKGRRIAIGWMDMWESEMPTKADG
WCGALTLPRELTLRDDHKLLMNPAEETKQLRTKEYRECAGRQVSGSYLAKTSEDLLEVRVVFDINDCDAESAGFKIR
GLDEEELVLKYNLTDKKLTLDCTKMGKAKDGVRRVRLDASGKLALRIFIDRSSIEVFANHGEATMTSRIYPKEGRLG
LELFSEKGAVKVEEFTYWTLKDIWKKS
>DGN2_GH32_1
MISRRTFTSTALLSLAGLPAARGAWAQAPAPAPVPSADERYRPVLHFTPPQGFMNDPNGLVFADGEWHLFYQHNPFE
AKMGRVHWGHAVSRDLLHWQVLPLALRETADGMAFSGSAVIDRANSSGLLAQRGEGNGRGGMVAVYTRASATRQVQA
LAYSSDGGRSFADYRGNPVLDVGSDQFRDPKVFWHAPGKVWVMCVVRSREHRVMFYRSRNLKDWRAAGEFAHAGVYG
IDYECPDLVELPVAGGGTRWVLFLSINPGAPQGGSTVQYFVGRFDGRRFVLDDGATRFADFGKDFYALQTFAGVDGA
PVGIAWMNNWQYANDLPTGRWRGAMTLPRRLSLRRVADDWKLVQEPLGLEAARRQVIAQGPGRASTALPPDTALEIR
LACAPDTVTSLRLANGTGEYLEVGYDPGPAVGSVWVDRGNARGFRHRFFTDKMSWAPPPDGKPLDLRLVFDRCSLEL
FAAGGTGCATLLHFFAAPPDRLELAVAGGPLPDIAVHALLPTGGR
>Salb_GH32_2
MADRTTGVLMRRQRPFRHAPLRALLALAAGLALVLPLGGASPQAAAAPPRAAGSASAEEPPGAEPYRPQFHFSPARN
WMNDPNGLVHYDGEYHLFFQYNPEGNTWGNMSWGHAVSRDLVHWEELPLALPHDEQEMVFSGSVVVDRENTSGFGSP
ENPAMVAVYTSHAKDGSRQAQSLAYSTDRGRTWTKYAGNPVLDIGAKDFRDPKVQWHEPSRSWLMTVALPLEHKVRF
YRSPDLKQWTELSDFGPAGAVDGVWECPDLFPMQVDGDPERTKWVLVVNLNPGGIAGGSGAQYFVGDFDGTRFTADP
DQGTRWLDHGKDYYAAISWDGDPEGRRHMIGWMNNWQYGEKIPTDPWRSAMSVPRTMGLSTVDGRPTLTQQPVAALD
SLRLPDPVTVREVDVPEGSQPLAEGRGDGRTLDIEARFSPGEAERFGLELRAGDGQKTVVGYDAAAGEVYVDRTHSG
DTGFSPDFPGTQRAPLAVRDGEVRLRILLDASSVEVFGGAGESVLTDQIFPGAPEGVRLFAEGGTARLKSLDLHRLG
TYRP
>GT265_GH32_1
MTSRTARIRVLAAFAAPLAARALAAQAGSPPAPSTMSGTTDPPPTYTERYRPQYHFTPAANWMNDPNGLVYYDGEWH
LFYQHNPFGNRHGHISWGHAVSTDLVHWQNLPVAIPHTGAEGVWSGSAVVDWQNTSGFGQGGKPPMVAIYTGFDEAA
KRQAQHIAYSTDRGRTWTRYAGNPVLTLDLGLPDFRDPKVFWHAPTGRWVMVVSLPTEHKVRFYGSPNLKQWSLLGD

FGPAGATGGVWECPDLFEVPVDGDPRHTRWVLVVNVNPGGIAGGSATQYFVGRFDGNRFAADEPSPRTANPGAPTVR
WADYGKDLYATVSWSDVPRADGRRVWIGWMSNWQYAFEIPTSPWRSAMSVPRTVALTTTRDGLRLVQQPVVELERLR
GERRHVGPQTIPVGSTSLAARGVTGKSMEIAATFEVNSAEPGRASEFGLKVRTGNGEETVIGIDPKARQLIVDRTRS
GQVGFHADFPSREAAPLPVEDGRVRLRVLVDWSSVEVFAGDGRVVVTDQIFPAPESEGVQLYAKGGTARLVSLDAWP
LASARTTAAAAPQRAATPGR

>Sphy_GH32_1

MRRQRPFRHAPLRALLALAAGLALVLPLGGASPGAAAAPSEAAGSAAAAGPAAAEEPPGAEPYRPQFHFSPARNWMN
DPNGLVHYDGEYHLFFQYNPEGNTWGNMSWGHAVSRDLVHWEELPLALPHDEREMVFSGSVVVDRENTSGFGSPENP
AMVAVYTSHAKDGSRQAQSLAYSTDRGRTWTKYAGNPVLDIGARDFRDPKVQWHEPSRSWLMTVALPLEHKVRFYRS
PDLRQWTELSDFGPAGAVDGVWECPDLFPMQVDGDPERTKWVLVVNLNPGGIAGGSGAQYFVGDFDGTRFTADPDQG
TRWLDHGKDYYAAVSWDGDPEGRRHMIGWMNNWQYGEQIPTDPWRSAMSVPRTMGLGTVDGRPTLTQQPVAALDSLR
LPDPVAVREVDVPEGSQPLAEGRGDGRTLDIEARFSPGDAEHFGLELRAGDGQKTVVGYDAAAGEVYVDRTHSGDTG
FSPDFPGTQRAPLAVRDGEVRLRILLDASSVEVFGGAGESVLTDQIFPGAPEGVRLFAEGGTARLKSLDLHRLGTYR
P

>Salb_GH32_3

MRRQRPFRHAPLRALLALAAGLALVLPLGGASPQAAAAPPRAAGSASAEEPPGAEPYRPQFHFSPARNWMNDPNGLV
HYDGEYHLFFQYNPEGNTWGNMSWGHAVSRDLVHWEELPLALPHDEQEMVFSGSVVVDRENTSGFGSPENPAMVAVY
TSHAKDGSRQAQSLAYSTDRGRTWTKYAGNPVLDIGAKDFRDPKVQWHEPSRSWLMTVALPLEHKVRFYRSPDLKQW
TELSDFGPAGAVDGVWECPDLFPMQVDGDPERTKWVLVVNLNPGGIAGGSGAQYFVGDFDGTRFTADPDQGTRWLDH
GKDYYAAISWDGDPEGRRHMIGWMNNWQYGEKIPTDPWRSAMSVPRTMGLSTVDGRPTLTQQPVAALDSLRLPDPVT
VREVDVPEGSQPLAEGRGDGRTLDIEARFSPGEAERFGLELRAGDGQKTVVGYDAAAGEVYVDRTHSGDTGFSPDFP
GTQRAPLAVRDGEVRLRILLDASSVEVFGGAGESVLTDQIFPGAPEGVRLFAEGGTARLKSLDLHRLGTYRP

>Pgor_GH32_1

MKVRTRNVLILASALMTCISTVEAQDLTMKITKKYLNLPVSHQTDRAVMTFDVGGKQERAFDIRLAPENPDYWVFCD
MSALKNKEIKISYNGNKAGINKIYQADEIAGQDSMYTETNRPQIHYTQRRGWNNDPNGLLHYDGEYHLFYQHNPYER
DWGNMHWGHAVSKDLIHWEELPIALYPDEHGTMFSGSAVIDYDNTSGFGKKGTPAMVAIYTADNPEKQVQCIAYSLD
KGRTWTKYQGNPVIDSKAKWNSKDTRDPKVFWHKPSNKWVLVLNERDGHSIYNSDNLKDWTFESHITGFWECPELFE
LPVDGNKNNTKWVMYGASGTYMIGSFDGKKFTPEAGKYYYSTGSIYAAQTFTNIPESDGRRIQIGWGRISHPGMPFN
GMMLLPTELSLRTTKDGVRLFSKPIKEFEQLQTKANQWSSLTADKANDLLQQYNDAGTLRIRTTIKLSHATNAGLNL
FGQSLLNYDMNFNLVNGVFYSPEDMTGMEITADIILDKTSVEVFIDNGAYSYSMERRPDLKNKEGFHFWGNNIEVKN
LEVYTLRSIWK

>Ostr_GH32_1

MKNLTLLFFLSIFLISCENRQHNGTELSDELYYKASVEQLLIPKADKKYLLLPIEDTGEELKTSILVDGQEIATYII
RLAKAKIDYWMPLDLSEWRGKDIRIHISGLNESAVCLKEIKLSDTFDFESNEKYRPAYHFTPPYGWMNDPNGMVYYA
GEYHLFYQYNPFGTRWQNMSWGHAVSTDLVHWEHLPIALKPDSLGTIFSGSAVVDENNTAGFQTGSEKTLLAFFTHS
ERQGQFQSLAYSNDKGRTWTKYANNPILKHSMAKNFRDPKVFWHNATRKWIMVLAVGQIIEFYSSTDAKEWTYESNF
GKGYGSHDGVWECPDLFELPVENTNETKWALIVNINPGGPMGGSATQYFVGNFDGKTFTATNKPEEKHWLDWGKDHY
AAVTWSNAPDNRRIAIAWMNNWDYANDLPTQNFRGATTVPRELKLQKQGNSYLLANYPVKELENLRQEKKELENIAV
VDDYSIDKLLDNNQGTFELVLDIENQSAEIIGFKLFNNVGEFVDVFISLPENKFYVMDRKSSRKIDFSDRFASVTSA
PIAAKKAYKLRLLIDKASIECFEGEGEISMTNLVFPSEPYNRIGFYANGGKYTVKKFEIYNLKYIR

>Dalg_GH32_1

MKIYFKSIFCLGLLSLIGCSTKSDIVVEDFEADSYKNWTVEGEAFGTSPSEGSIPGQQEVKGFEGKRLANSYHGGDD
SRGTLISPEFTIERDYINYLIGGGMHADTYIELLVEGKSVYVSRSIVETESLLWMTWDVKNYKGKKAVIKIVDNQRG
GWGHILVDQIEMSDTPKSNIMVDHRLSFKIDKKYLLVPIEDKGPESQIYLETNGKTVSPLLNIRVAQTKIDYWVPID
VEQYKGQDLTLLFAHVKSGDIGYSQIKQSDTFDFDYNEKYRPLYHFSPQYGWTNDPNGMVYHNGEYHLFFQHNPYGS
MWGNMNWGHTVSKDLKKWEYMPVAINPDSLGTIFSGSAVIDKNNTAGFGKDAMVAIYTSAGRIQTQSIAYSLDNGRT
FTKYDKNPVLSDLNFVDFRDPKVFWHQGTNQWIMSLATTQTITFYGSKNLKEWSKLSEFGEGIGDHGGVWECPDLFP
LTYNGQTKWVLFVSINPGGPNGGSATQYFIGNFDGKTFKADNLPYPLWLDYGRDNYAGVTWSDAPDNRRVFIGWMSN
WNYTNHVPTVNFRNAMTIPRELTLVNNGKHLVVANAPVKEIAELRRATEKVSDFTVDKTYTVDRLLKDNQGAYEIEM
TVKPTANFNFKLINKKGENLKFTFDLAKGSLIVDRSNSGISDFSDNFASAEIKAPLVKKDTYKIRLLIDKASSELFI
NDGELVQTNTMFPSEPYNSLVFESEGTIAVENINIYELK

>Sbac_GH32_1

MKKQLYLFYLLLSNILFAQTSVEPHRPLFHFTPKTGWMNDPNGMVYYKGEYHLFYQHYPDSTVWGPMHWGHAVSKDL
MRWEHLPIALFPDSIGCIFSGSAVVDVKNTSGFGKKGGESPLVAIFTYHNFEGERANRNDFQSQGIAYSLDKGRTWV
KYAHNPVIKNTGKRDFRDPKVMWYEPTQQWIMTLAVANHVEFYTSKDLKNWAQTGEFGHTEGSHGGVWECPDLFQLP
VDGGKTQKWVLIVNIGNGAPNGGSGGQYFIGEFDGKTFKNDNKPSDILWLDYGTDNYATVTWADAPNQKRLSLGWMS
NWQYAQIVPTQTWRSAMTLPRELGLVNTHQGVRLIQKPVKEVEILRGDKNTLSKQVIASTLGLKTKGKGQEISLTFD
LSKTTASDFGIELVNSKGEKVWIGYEKTTQRFFIDRTEGGKKDFHKGFAKRHYAPRLSKDNLLKLSIFIDVASVELI
ADDGLTAMTDIFFPNEDFNKTAIFSKNGSTFLEKCVFWQLK
>DHDWB_GH32_1
MNIYFKSIFCLGLLSLIGCSAKSDIVIEDFESDSYKNWTVEGEAFGTSPSEGSLPGQQDVKGFEGKRLANSYHGGDD
SRGTLISPEFTIERDYINYLIGGGMHADIYIELLVEGKSVYVSRSIVETESLLWMTWDVKNYKGKKAVIKIVDNQRG
GWGHILVDQIEMSNTPKSNIMVDHRLSFKIDKKYLLVPIEDRGPESQIYLEQDGKAVSPLLSIRVAQTKIDYWVPIN
VEQYKGQDLTLLFAHVKNSDIGYSQIKQSDTFDFDYNEKYRPLYHFSPQYGWTNDPNGMVYHNGEYHLYFQHNPYGS
MWGNMNWGHTVSKDLKKWEYLPVAIAPDSLGTIFSGSAVIDKNNTAGFGKDAMVAIYTSAGRIQTQSIAYSLDNGRT
FTKYDKNPVLSDLNFVDFRDPKVFWHQSTNQWVMSLATTQTITFYGSKNLKEWSKLSEFGEGIGDHGGVWECPDLFP
LTYNGQTKWVLFVSINPGGPNGGSATQYFIGDFDGKTFKADNLPYPLWLDYGRDNYAGVTWSDAPDNRRVFIGWMSN
WDYTNHVPTVNFRNAMTIPRELTIVNNGKHLVVANAPVKEIAELRRATEKVSDFTVDKTYTIDRLLKDNQGAYEIEM
TIKPTANFNFKLVNKKGENLKFTFDLAKGNLLVDRSNSGLSDFSKNFASAEIKAPLVKKDTYKIRLLIDKASSELFI
NDGELVQTNTMFPSEPYNSLVFESEGNVTVENINIYELK
>Phie_GH32_1
MKFKLYCWFRLMVCATCFLCTGLQASAQHWSFDGENPLKADKKNDVLELKNVRNQIELTAGVIGKGLRTDGYSTSLR
TEIRPNQNVSACSGWFALESFPTDTAAFFALKDRRTGISISVGVNRFGEIVIGKGNGKDFSYLPTGHFVKKFSWLNI
ALGMGSADRGVWINGQKLKINMSLINFPAKAGEILIGKDFRENLLGQMDLTAINGIIDELRLWNKPLPIKELQNEIA
VLAKKVPVLAIPAERFKDDFSRPKYHLMPAANWTNETHGLIFYKGRYHIFNQKNASNLALRQINWGHFSSPDLVNWT
EHKPALSPERGYDENGIWSGHVILDDKGIPMISYTAGGPKMGIALAFPKDSTLNDWIKYKQNPVIPGQPDGYGRTDL
RDTYVWKEGNKWYMVVGFGVKKDDVEKGALLLYSSPDLKNWNFLHTLFEGNPDLDNSGIFWEMPVFKKIGDKYVLLV
NKVPHKGVPARALYWVGSFVNERFVPDNPMPKNLEVINRLLSPSVTEDKDGRICAIAIIPDEISGEAAYNQGWSHLY
SLPRVWNLTDGTLNQSPHPALRSLRVANSNFFDATVANHQSVKLYSGHQQYEVVAQVRPNKSKQFGFVLHKNPDGSE
YTKIYYDVDAKELVVDQRHSSTKKGIPLRIKKDAFALDTSKPVGFHVFVDGSVVEIFINDQHALTTRIFPSKEQSNQ
AEIFSEGDSIQIKAEVWAIRPAVINTDF
>Hhat_GH32_1
MNHKKSRVLAVAISSLLSFGMILQTAAPPQVYASEGIRRESSQKVSDWTFKAADNNDQTAVTEVVKSDAYLSGLEIK
GGSLDKPFSREFSSYSVVVGHKTDSLTIIPKIDGNAAVTVDGKAAGSGQAVTVNVKTGSNVFNIVVKGANGKTETTT
VNVLRNSDPDDVYSVPYRSQYHFSPQRAWCNDPNGMVYYKGEYHLFYQYYPDDMIWGPMHWGHAVSKDLVHWEELPI
ALYPESDGGAMFSGCCVVDKDNTTGFFGQGKEGLVAIYTQDNGDKGQEQCLAYSKDKGRTWTKYQGNPVLRWEDDRL
LDKAFRDPKVFWHEESGQWMMVVAGGPLRIYSSKNLKSWKLESTYGKKTNEGIADQPADSARIYTECPDLYPLKVDN
TDQTKWVLSEGGRYYRIGDLKQQNEHWTFVPDSGYSNTNDNISDPSSYKNDDTYKMNFGPQSYAAQTYSNMPDSRTV
MINWASTWENGYCNNVSEVTGQYGFNGFFNLQNELTLKQIDGKVRLVQKPVKEYESLRMQQAKTELKNAVIPEKTDS
SENLLSGFQGGEYEIIAEFTPKTGTREVGFKLRTGKNGKQETVVKYNVQDETLIINGDKAGVLPQGQIKGDIKSKIS
KSADGKIKLHIFVDDSIVEVYGNEGETVGSLAVFPDAKSTGAEVFSEGGQTEADITIYPLKSIWSDKMSGSTDPANV
YLNTETASSSVKLGETTTLNSTVTPQKAEQKVSWSIAGNSGKKVSIVKQDAGSITLKGENQGTVIVKAKTSNGIART
MEIQVDGEDEKLNSNLSGWHQVGGQWTLDANGYNSNHKDNGFLISSTKTGSDYIYEADVTYQEGNAIGLFFRGQSPD
SDKGYVVNIDDPEHKGNGSTRIFTFGGGTGDIGSRVKYTLTPGATYHLKLEVRGNKFKFYINDSLKLNVTDRNIKKN
YAEGDYVGLYAFSGTGESDKNIQASYQNIKVTPLEVRKPSVREKMELYSGQSIQLAADLGEDDYAAAYYESSDPSV
>DHDWA_GH32_1
MKIYFKSIFCLGLLSLIGCSTKSDIVIEDFESDSYKNWTVEGEAFGTSPAEGSLPGQQEVKGFEGKRLANSYHGGDD
SRGTLISPEFTIERDYINYLIGGGMHADTYIELLVEGKSVYVSRSIVETESLLWMTWDVKNYKGKKAVIKIVDNQRG
GWGHILVDQIEMSNTPKSNIMVDHRLSFKIDKKYLLVPIEDRGPESQIYLEQDGKAVSPLLSIRVAQTKIDYWAPIN
VEQYKGQELTLLFAHVKNSDIGYSQIKQSDTFDFDYNEKYRPLYHFSPQYGWTNDPNGMVYHNGEYHLYFQHNPYGS
MWGNMNWGHTVSKDLKKWEYLPVAIAPDSLGTIFSGSAVIDKNNTAGFGKNAMVAIYTSAGRIQTQSIAYSLDNGRT
FTKYDKNPVLSDLNFVDFRDPKVFWHQGTNQWVMSLATTQTITFYGSKNLKEWSKLSEFGEGIGDHGGVWECPDLFP
LTYNGQTKWVLFVSINPGGPNGGSATQYFIGDFDGKTFKADNLPYPLWLDYGRDNYAGVTWSDAPDNRRVFIGWMSN
WDYTNHVPTVNFRNAMTVPRELTLANNGKHLVVANAPVKEIAELRRATEKVLDFTVDKTYTIDRLLKDNQGAYEIEM

TIKATANFNFKLVNKKGENLKFTFDLAQDKLLVDRSNSGLSDFSKNFASAEIKAPLVKKDTYKIRLLIDKASSELFI
NDGELVQTNTMFPSEPYNSLVFESEGNVTVENINIYELK
>Lbac_GH32_1
MNNEKHEQRIKEAEKAITAGSFLAQQGKYRQAYHFMAPAGWINDPNGLIYFQKKYHLFYQYNPYQAVWGSMHWGHAV
SDDLLHWDHWPIALAPSESYDDHPEGGCFSGSAVEDNGELNLLYTAAVNTEYGSVQTQCLAVSTDGAKSFQKYDHNP
VISALPAGISEDFRDPKVIRYQNFWYLVLGASSGSGARGGGDGCALLYQSSDLKKWEYRGVIARSDGKLGSMWECPD
LFPLRDKWILLFSPMLCGEKKTIYLIGSMNFETACFLPEQQGELDFGPDYYAPASFLDPKGRRVLMAWANGWEWMPW
HTGFGKTENEGWRGHLAIPRELRRLESGQLQFIPIQELETLRTKPKKYTAFTLPAGEVFEIQAGNGIQCELIFEISL
KNTSAEYIHFNLRCGAAERVRITVNLLGKDLIWQKKESSGEITTICRPLYAEQNENFKMHIFMDTCSVEVLADNYRL
AASGNVYFSADSNRIYIEAEGGEASFNNIYTYGLKSCQQN
>Pmeg_GH32_1
MNKINQAQNSLNEAEKKVNHQYRLGYHIMAPANWINDPNGLVQYKGEYHVFYQHHPYDENWGPMHWGHVKSKDLVHW
QHLPIALAPGDTFDKDGCFSGSAVDNEGELTLIYTGHNYIDKELDTFFQNQNIAVSKDGITFEKAEANPVIAEPPAD
SSHHFRDPKVWKHEGFWYMILGNSTKKQEGRVILYRSSNLRKWEYVGVLAKSDGDLGYMWECPDFFELDGKHVLMIS
PQGIEAKGNSYHNLFQTGYLVGEYNYETNTFHHGSFTELDYGHDFYAVQTLLDDKGRRIAIGWMDMWEANMPTKDDG
WCGALTLPRELTLRGDKVLMNPVQELTSLRETQYNMLTNKALSNSYVVEVNEDLLEIQAVFDLAECQASSVGIKIRG
INNEETLMCYNLNEQKLLLDCTHSGKEDGVRKVALQAGETLALRIFVDRSSIEVFANEGQVTMTSRIYPKESRLGIE
LFTEGGDVIVKELTYWNLKDIWG
>Pmeg_GH32_2
MNKIEQAQHSLNEAEKKVNHQYRLGYHIMAPANWINDPNGLVQYKGEYHVFYQHHPYDENWGPMHWGHVKSKDLVHW
QHLPIALAPGDPFDKDGCFSGSAVDNEGELTLIYTGHNYIDKELDTFFQNQNIAVSKDGITFEKAEANPVIAEPPAD
SSHHFRDPKVWKYEDFWYMILGNSTKKKEGRVILYRSSNLRKWEYVGVLAKSDGDLGYMWECPDFFELDGKHVLMIS
PQGIEAKGDSYHNLFQTGYLVGEYNYETNTFHHGSFTELDYGHDFYAVQTLLDDKGRRIAIGWMDMWEANMPTKEDG
WCGALTLPRELTLRGDKVLMNPVQELTSLRKTQYNMLTNKALSNSYVVEVNEDLLEIQAVFDLADCQASSVGIKIRG
INNEETLMCYNLNEQKLLLDCTHSGKEDGVRKAALQAGETLALRIFMDRSSIEVFANEGQATMTSRIYPKESRLGIE
LFTEGGNVIVKELTYWNLKDIWG
>Lapi_GH32_1
MKKAVQVTNERYRLNYHISTPAGWLNDPNGFSYFKGYYHIFYQYHPYSAEWGPMHWGHYRSKDLVHWEELPIALSPD
SESDCDGCYSGSAIEKDGRLFLIYTGNRYCDNKHEQFCQTQNVAFSEDGVHFKKYDHNPVIESAPVDNSAHFRDPKV
WRHNDKFYLVIGSQSKEGLGRVLTYQSNDLYHWQYLGPIAEAADVAEEGYMWECPDLFSLNGKDILLCSPQGIESAG
KKYLNCHQTGYFVGQMDYQNNRFEHGSFTELDHGHDFYAAQTMRTPDNRQILFGWMDMWHSNFPELADGWSGALTLP
RELKLKDNRLLMTPVEELIALRQKKEVDQKLAVENEQINVSDDQHVELLLNFSLENWEGKQIIFTLKESAGNILTLT
FNSATNEVVIKRSDKEPNDAKRFGQVNTSDELKFRIFIDTSSVEFFINDGELVFSERYYTEHEPRIVLTADKPVMTQ
ITAYNLSK
>Aalb_GH32_2
MKTLQNHQENIERANKAIDKIKDQVSQHEWRLQYHVMPPANWMNDPNGFSFFRGEYHLFYQHHPYDPKWGPMHWGHV
KSKDLVNWEHLPIALAPSEPYDKDGCFSGSAIEKDDKLYLIYTGNVWIEDGPLDEDLTQVQAVAVSEDGIHFEKLKE
NPVIAEIPDGDINIFHFRDPKVWKRGDTYYMVLGSKTKKNTGQVLLYRSEDLIRWDFVSIMAKGEGNFGFMWECPDL
FELDGKDVLVMSPQGLEPEGDKYHNLHQAGYIMGQLNYNTGKFNHGALHLLDHGFDFYAPQTMVDDQGRRILVAWMD
MWESEMPTQTHGYAGAMTIPRLVTRDKDNLRTVPVPELQTLRQHQVSYENVQVNGASSLNGVEGDCLELEIDIDPNE
ATTFGLKLRVDDEVNQETKLLFDQQAGTVTLDRNKSGKGPGGFRRTTVNMAKNVHLQIFIDKSSVEIFINRGEKVMT
SRIFPTENATGISFFADQEMTIERLSKWELKQAIK
>BBac7_GH32_1
MLTTNKLQLAQEAIEKAKQQMNSRYRLGYHIMAPANWINDPNGLVQYKGEYHVFYQHHPFDENWGPMHWGHVKSKDL
VHWEHLPIALAPTEAYEKDGCFSGSAVDDNGILTLIYTGNIFIDKEKDILDQSQCIATSTDGIHFTKDTANPVISKH
PEEGSGHFRDPKVWKYEDFWYMILGTRKEDTGKVVLYKSSDLREWQYIGVLAESDGSLGYMWECPDFFELGGKHVLL
FSPQGMEAQGELYNNIFQTGYIVGDYSYETNSFEADDFTELDNGHDFYAVQTFLDDKGRRIAIGWMDMWESIMPTKE
DGWCGALTLPRELTLDQNNKILMKPVEELSLLRTTEQKVCTNKHLAESYLVEANNDLMELKLDIDLTKTAAQTVGVK
IRGINQEETLIKYETKEQKLVLDVSKAGKSEDGVRGTTLKADTQLSLRVFIDRSSIEVFANDGQVTMTSRIYPNEAK
LGFELFAENGAAEVIDFTYWELKDIWE
>Ncir_GH32_1
MISTNKLQQAEQAIQNAKKQINNRYRLGFHIMAPANWINDPNGLVQYKGEYHAFYQHHPYDENWGPMHWGHVKSKDL
VHWEHLPIALAPTELYEQDGCFSGSAVDDNGILTLIYTGNIFIDKEQDILDQSQCIATSTDGIHFTKETVNPVISKH

PAEGSGHFRDPKVWKHEDLWYMIIGTRQEDTGKVVLYKSQDLRTWQYVGVLAESDGTLGYMWECPDFFELGGKHILL
FSPQGIEAEGDLYNNLFQTGYLVGDYDYTTNTFKSGSFTELDNGHDFYAVQTLLDDKGRRIAIGWMDMWESSMPTKE
DGWCGALTLPRELTLGENNKILMNPVDELSLLRTKQQKVCTDKSLSGSYLVQATQDLIELKLDFNLTKTSAQAIGVK
IRGTNQDETKLFYDVEQQKLVLDCSKSGKGEDGVRRTALKAEQQLSLRIFIDRSSIEVFANDGEVTMTSRIYPTEER
LGFELFSENGETEVMDFTYWELKDIWE
>Vago_GH32_1
MTKKEPIELTNSRYRLGYHVAAPSGWINDPNGFCYFDGYYHVFYQHYPYSAEWGPMHWGHARSKDLVHWESLPIALT
PGDKEDEDGCFSGSAIEKDGVLYLFYTGHHYYGDGDKDHFWQNQNMAYSTDGIHFTKYENNPIIAKEPGDNTHHFRD
PKVWEKDGVYYMILGSQGEDGLGRAIVYSSKDLLDWKYEGPISKANGLKTEGFMWECPDFFNLDGKDILLLSPQGID
AQGKDYLNLFQTGYFIGDYDYKTATFTRGEFHELDKGHDFYATQTTEAPDGRRIVIAWMDMWESLFPEQEDGWAGAL
TIPRELRLKNDHLYMTPVKELEDLRIKEVSNNSSVVAKELLVAEDASSSEVLVDIPLTGTDQEEVSFSLKTSNEELV
LLTYSKATNEFILKRSDKDDLRYGTIQPCDKLSLRVFIDTSSIEIFINEGELVFTERFYTGEKTDVCVTVSEEETIS
YTVYQLDYNAVSYN
>Mlac_GH32_1
MTGTVNLQQAEEAINKAKKEVNNRFRLGYHIMAPANWINDPNGFVQFKGAFHVFYQHHPYSTDWGPMHWGHVISKDL
VNWEHLPIALAPGDYCDKDGCFSGSAVDNDGELTLIYTGHHYVDKDKDLFYQNQNIAVSSDGVHFDKVIENPVISAP
PPDSSQHFRDPKVWKHEDHWYMVVGNSSKDETGRVILYRSINLRNWDYIGVLAESDGTNGYMWECPDFFEIGGKYVL
MISPQGMDAKDDCYNNLFQTGYLVGDYSYDTNTFNHGAFKELDYGHDFYAVQTLKDDNGRRIAIGWMDMWESYMPTK
EDGWCGALTLPREITLDGQDRIRMNPVDELELLRLDENTICHKKVTSKSIAAETTEDLLEIKAVIDLASSNTKNIQM
KISYSCGQHTLITYDAEREKLILSVAGGKGLRSVALKNSGQLLLRIYLDRSSIEIFANHGEATMTSRIYPEGNRREL
TLLSNGEFTVNEFTYWKLKDIWK
>Axia_GH32_1
MEHVTEKQDHQTRVKQATEAGKAKQNPDAAFRLGYHLMAPSGWINDPNGLVFYKEQYHVFYQHHPYDETWGPMHWGH
AVSDDLVHWRHLPVALAPGDAFDQSGCFSGSAVDDDGVLTLIYTGHNVVNQETDELYQNQNIARSVDGVTFEKSEAN
PVIPAQPVGMQRDFRDPKVWKEDGSWKMVVGSTEAGKGQVLLYQSSDLENWEYQGILAKHNGGNEGYMWECPDFFKL
DGKHVLLLSPQGIEPEGNRFHNLYQTGSLIGEMRDGEFVRGAFTELDYGHDFYAVQTFLDGKGRRIAIGWMDMWESP
MPSQQEGWSGALTLPRELNLSPAGKLRMKPVEELQLLRVTEEQLKQNEISGTFLIDGQSNQLELEFDIDLTISAANI
FGVNVCCSSDGSERSTIQINRSKGTITLDRSYSGIGVSGARTAPIEIGESLHVRLFIDRSSIEIFINEGETVMTSRI
YPKESSTGIEFFTEEGTMKLRSAHLYQLKNSWLTD
>BRHFB_GH32_1
MNRIKQAEEALKHAKNKINNRYRLGYHIMAPANWMNDPNGFAQYKGEYHAFYQCHPYDANWGPMHWGHVKSKDLVHW
EHLPVALAPGDACDTDGCFSGSAVDNNGELTLIYTGHHYTDKEKDLFHQNQNIAVSTDGISFEKVGENPVIAKPPVD
SAHHFRDPKVWRHNDTWYMVLGNSTKDNVGRVILYRSSDLLNWEYIGVLAQSDGSLGYMWECPDFFELDGKYILMIS
PQGIKSNGDLYNNLFQTGYLVGDYNYETNEFVHGSFTELDNGHDFYAVQTLLDDKGRRIAIGWMDMWESNMPTKADG
WCGALTLPREITLGDHNKILMNPVEELILLRDSEHIECTNQSISESYMIETKEDLLEVKAVFDLTNCSAQSVGLKIR
GVNQEETVLTYHLDKQQLTLDCSKSGKVEDGVRSTMLETNKELSLRVFIDRSSIEIFANDGQTTMTSRIYPNEDRLG
IELFTEKGDVQVKEITYWNLQDIWK
>BAFS02_GH32_1
MNRIKQAEEALKNAKKKINDRYRLGYHIMAPANWMNDPNGFVQYKGEYHAFYQCHPYDANWGPMHWGHVKSKDLVHW
EQLPVALAPGDACDTDGCFSGSAVDNDGELTLIYTGHHYTDKEKDHFYQNQNIAVSTDGISFEKVGENPVIAKPPVD
SAHHFRDPKVWRHNDTWYMVLGNSTKDNVGRVILYRSSDLLNWEYIGVLAQSNGSLGYMWECPDFFELDGKYILMIS
PQGIKSNGDLYNNLFQTGYLVGDYNYETNEFVHGSFTELDNGHDFYAVQTLLDDKGRRIAIGWMDMWESNMPTKADG
WCGALTLPREITLGDHNKILMNPVEELILLRDSEHIECTNQSISESYLIETKEDLLEVKAVFDLTNCSAQSVGLKIR
GVNQEESVLTYHLDKQQLTLDCSKSGKVEDGVRSTMLETNKELSLRVFIDRSSIEIFANDGQTTMTSRIYPNEDRLG
IELFAEKGDVKVKEITYWNLQDIWK
>Ntax_GH32_1
MLTTNKLQQAEQAIQNAKQQLNKRYRLGFHIMAPANWINDPNGLVQYKGEYHAFYQHHPYDENWGPMHWGHVKSKDL
VHWEHMPIALAPTESYEKDGCFSGSAVDDNGVLTLIYTGNIFVDKEQDILDQSQCIATSTDGITFTKETANPVISKH
PAEGSGHFRDPKVWKHEDSWYMIIGTRKEDTGKVVLYKSQDLRAWQYVGVLAESDGTLGYMWECPDFFELGGKHILL
FSPQGVEAEGDLYNNLFQTGYFVGDYDYKTNAFKSGSFTELDNGHDFYAVQTLLDDKGRRIAIGWMDMWESSMPTKE
DGWCGALTLPRELTLNENNKILMKPVEELSLLRTQQQKVCTDKSLSGSYLVEATQDLIELKLDFDLTKTSAQAIGLK
IRGTNQDETKLVFDVEQQKLVLDCSKSGKGEDGVRRTALKADQQLSLRVFIDRSSIEVFANDGEVTMTSRIYPTEER
LGFELFSENGETEVIDFTYWELEDIWG

>Lpla_GH32_1
MQIDETKVIKVTNERYRLNYHLSPNSGWMNDPNGFVYFKGYYHIFYQYYPYDSQWGPMHWGHARSHDLIHWEQLPAA
LVPGDREDEDGCFSGSAIVKDDKLFLIYTGHHYYDKNDPDNFWQNQNLAFSSDGVHFTKYENNPIIATPPKDNTIHF
RDPKVWEHDGKYYVVIGSQNEEKLGRILMYVSNDLFEWEFKGTIAESKSSFEEGFMWECPDLFRLNGKDILLFSPQG
IKAQKQKYLNLFQTGYFIGDFNYQKANYKHHEFVELDHGHDFYATQTMLAPDGRRIVFGWMDMWEANMPEQADGWAG
ALTLPRELTLKDDHLYMNPIEETKQLRKTELASEKCTTAKYNLSLPKENGTEILLTSQIQNWKGNTITFSINSAEKN
KMALTYDKKDQKLTLTRQGQDAKRYAKLKKCSELKLHVFIDRSSVEIFINDGEAVFTERLYSEKNYEVAVEAKQTID
MNIDSYLLSRSE
>Mfor_GH32_1
MLTTNNRLQQAEEALQKAKEKLNERYRLGYHIVAPANWINDPNGLVQFKGEYHVFYQHHPYDENWGPMHWGHAKSKD
LVHWEHLPVALAPGDACDKDGCFSGSAVDNDGELTLIYTGHHYTDKEKDLFTQNQNIAVSQDGVTFEKAEENPVIAE
PPADSAHHFRDPKVWKHGDSWYMILGNATKDEVGRVILYRSADLRKWEYIGVLAQSEGTLGYMWECPDFFELDGKHV
LLISPQGLEAEGDLYNNRFQTGYLIGEYDYETNKFSHGEFIEMDNGHDFYAVQTLKDDKGRRIAIGWMDMWESNMPT
KEDGWCGALTLPRELTLDKRNKVLMNPVDELTLLRETEHKVFANQSISGNYLAKTNQDLIELKVEFDLTKTTAQAVG
FKIRGLDQEETVLRYQVKDQKLVLDCTKHGKKEDGIRRTALEAGDRLSLRVFIDRSSVEIFANEGQATMTSRIYPQH
ERLGIELFAEDGDAQVSEFTYWSLKDIWK
>BAFS09_GH32_1
MNRIKQAEEALKNAKKKINDRYRLGYHIMAPANWMNDPNGFVQYKGEYHAFYQCHPYDANWGPMHWGHVKSKDLVHW
EQLPVALAPGDACDTDGCFSGSAVDNDGELTLIYTGHHYTDKEKDHFYQNQNIAVSTDGISFEKVGENPVIAKPPVD
SAHHFRDPKVWRHNDTWYMVLGNSTKDNVGRVILYRSSDLLNWEYIGVLAQSNGSLGYMWECPDFFELDGKYILMIS
PQGIKSNGDLYNNLFQTGYLVGDYNYETNEFVHGSFTELDNGHDFYAVQTLLDDKGRRIAIGWMDMWESNMPTKADG
WCGALTLPREITLGDHNKILMNPVEELILLRDSEHIECTNQSISENYLIETKEDLLEVKAVFDLTNCSAQSVGLKIR
GVNQEESVLTYHLDKQQLTLDCSKSGKVEDGVRSTMLETNKELSLRVFIDRSSIEIFANDGQTTMTSRIYPNEDRLG
IELFAEKGDVKVKEITYWNLQDIWK
>Sele_GH32_1
MQEAMKVTNQRYRLGYHLMTKGGWMNDPNGFSWFKGYYHMFYQYYPYAAEWGPMHWGHARSKDLVHWETLPVALCPD
ENEDGCFSGSAVVYDDKLWLIYTGHHVTNPKDSEEFYQDQNIAWSEDGITFHKYEGNPVLRAPEGNTKHFRDPKVWQ
EGDTFYMVLGSQGSDELGRALLYESKDLKQWQPVSVLDKAATLEDEGYMWECPDFFRLDGRDILLMSPQGLEPQGDC
FRNLNQTGYLLGSRDEHEHLVRKNFTEIDRGHDFYATQTMLAPDGRRLMTAWMNAWDSPMQEKEDGWAGALTLPREL
RVEKGRLYQQPAREMASMRTRTLLAGSLAAGSRLPIPATSELELEFREAGDFNGSLLKITGGTQQLEISLDAKGGRI
IVQRTTKDGSRAARLLPFEDLKLHIFVDKSSAEIFVNEGELTFTERIYWQEPLELSLSGKAAEAARVYALEKETNQY
>BJCM_GH32_1
MEHVTEKQDHQTRVKQATEAGKAKQNPDASFRLGYHLMAPSGWINDPNGLVFYKEQYHVFYQHHPYDETWGPMHWGH
AVSDDLVHWRHLPVALAPGDAFDQSGCFSGSAVDDDGVLTLIYTGHNVVNQKTDELYQNQNIARSVDGVTFEKSEAN
PVIPAQPVGMQRDFRDPKVWKEDGSWKMVVGSTEAGKGQVLLYQSSDLENWEYQAPLAKHNGGNEGYMWECPDFFKL
DGKHVLLLSPQGIEPEGDRFHNLFQTGSLIGEMRDGEFVRGAFTELDYGHDFYAVQTFLDGKGRRIAIGWMDMWESP
MPSQQEGWSGALTLPRELNLSPAGKLRMKPVEELKHLRVKEEQLEQNEISGTFLVDGQSNQLELEFDIDLSNSDADI
FGVNVCCSSDGLEKTTILVNRSKWTITLDRSYSGIGVSGARTAPVELTDSLHVRLFIDRSSIEIFLNEGETVLTSRI
YPKESSTGIEFFAEEGFMRLSSAHLYQLKNSWLTD
>Mora_GH32_1
MKQEMHVTNQRYRLGYHLMTKGGWMNDPNGFSYFKGYYHIFYQYYPYAAEWGPMHWGHARSTDLVHWETLPAALAPG
DMESGCFSGSAVPHDGKLWLIYTGHHYIDPKDPEAFYEDQNLAYSEDGIHFTKYEGNPVLRVPKDNTKHFRDPKVWR
EGDTFYMVVGSQGADGCGRALLYRSPDLIDWTRCSVLAKSENEKTEGYMWECPDFFPLGDKYVLLMSPQGLEASGDD
YRNLNQTGYLVGTLDKETHVLAHGKFVEIDRGHDFYATQTMETPDGRRVMMAWMAAWDSPMHEKEDGWAGALTIPRT
LRLAGGKVYQSPIREMESLREKTLQDGAWEAGKAYALPRKAEICLRFDAAPAVLLTLTDGTHGLSLAWEAEKNRLVL
TRKTKDGVRACTLSAAPHAVRAFLDQSSVEVFVNDGETTFTERIYFAGNVTLTAAAQEGAIYALEAETNTYGTDAAE
EETK
>BJFL_GH32_1
MDRIQQAEEALKEAEGKVKQRYRLGYHIMPRANWINDPNGLIQFKGEYHVFFQHHPYDENWGPMHWGHVKSKDLIHW
EHLPVALAPGDAFDQSGCFSGSAADDNGRLTLIYTGHNMIDQEKDLFYQTQNIAVSQDGIVFEKLQENPVIAAPPED
SARHFRDPKVWKHRNEWYMVIGNSSKENVGRVVLYRSSDLRNWEYAGVLAQSDGHLGYMWECPDFFELGGKHVLLIS
PQGIEADGDSYNNLHQTGYLIGDYNYETNKFTHGAFTELDYGHDFYAVQTLLDDKGRRIAIGWMDMWESEMPTKADG
WCGALTLPRELTLRDDHKLLMNPAEETKQLRTKEYRECAGRQVSGSYLAKTSEDLLEAQVVFDINDCDAESAGFKIR

GLGEEELVLKYNLTDRKLTLDCTKMGKAKDGVRRVRLDASGKLTLRIFIDRSSIEVFANHGEATMTSRIYPKEGRLG
LELFSEKGAVKVEEFTYWTLKDIWKKS
>BSA1_GH32_1
MLKQSEIHQAGEALKKAKEKMNERYRLGYHIMAPANWINDPNGLIQYKGEYHVFYQHHPYDEHWGPMHWGHVKSKDL
VHWEHLPIALAPTEAYEKDGCFSGSAVDDNGTLTLIYTGNLFVDREKDILDQSQCIATSTDGIHFTKEAANPVIAKH
PEEGSGHFRDPKVWKHEDHWYMVLGTRKEDTGKVVLYKSKDLRQWEYLGVLAESNGNEGYMWECPDFFEINGKYVLL
FSPQGIKADGENYQNLFQTGYLIGDFSYETLEFSYGEFKELDHGHDFYAVQTFLDDKGRRIAIGWMDMWESDMPTKE
DGWCGALTLPRELTLAENNKLLMKPVEELTLLREAEHHICENKSISGSYLVETNADLLELKVEFDLAAATAEAVGLK
IRGLNDEETLLMYQIDNQKLTLDCSKHGKDKDGIRRKKIESSKRLSLRVFIDRSSIEVFANDGQTTMTSRIYPKEER
LGIELFAVNGTGQVSECTYWNLKDVWK
>Ssim_GH32_1
MTQLPAKVTNQRYRLGYHIMPKSGWINDPNGFSYYDGYYHIFYQHYPYAPEWGPMHWGHARSKDLVHWETLPIALTP
GDMEDKNGCFSGTAIVKDDQLYLFYTGHHYYEDNDPDHFWQNQNLAYSKDGIHFTKYQNNPVIAEPPSDNTHHFRDP
KVWQHNNQYYMIVGSQNDNDLGRIILYRSDDLLNWEYLGPVAQSNGLLSEGYMWECPDFFELDNQFVFLLSPQGMES
EEEQYLNLFQNGYMVGQFDYESFQFNHTQFTELDHGHDFYAPQTMLSPDGRRIVIAWMAMWESNMPEQQDGWSGALT
LPRELKLIGQKLYMQPIDELTQLRLDDGVHQQINLSNKQLLSQDTSFVELNIETVAEDFTIALNNSEHDLITIQYSH
NNNKFKLYRHDLEDYRYATIDPCSDIKLHIFIDKSSLELFINNGEAVFTERYYSECAPEVWMSSPHNIVAHVDKYHL
TSSAVDYE
>Hspor_GH32_1
MLTQSKIQQAEEALQKAKVKMNHRYRLGYHIMAPANWINDPNGLIQYKGEYHVFYQHHPFDENWGPMHWGHVKSKDL
VHWEHLPIALTPTEDYEKDGCFSGSAVDDNGVLTLIYTGNIFVDREKDIVDQSQCIATSVDGIHFTKEAANPVISKH
PKEGSGHFRDPKVWRHGEYWYMVLGTRKGNTGKVILYKSKDLRQWEYLGVLAESDETLGYMWECPDFFELDGKYVLI
FSPQGMEAKGDLYNNLFQTGYLIGEYDYETNKFIHGSFTELDHGHDFYAVQTLLDDKGRRIAIGWMDMWESNMPTKE
DGWCGALTLPRELTLGENIEILMNPVEELTFLRELKNSVCTDKSFSGTHLVETKEDLIELKLEFDLTKTAAESVGLK
IRGINQEETTLLYQIENQKLTLDCTKSGKGEDGVRRTKVESNQVLSLRVFIDRSSIEVFVNDGQATLTSRIYPKEER
LGIELFTENGDAHVIDFTYWKLKDIWR
>Bmrc_GH32_1
MFQKSRTNRAEEALSEAKGKMNDRYRLGYHIMAPANWINDPNGLVQYKGEYHVFYQHNPLDENWGPMHWGHVKSKDL
IHWEHLPIALIPGDACDTDGCFSGSAVDNDGELTLVYTGHHYTDKEKDIFYQNQNIAVSKDGITFEKVSENPVIDHP
PADSAHHFRDPKVWKHDDTWYMVLGNATQDKVGRVILYRSPDLRHWEYRGVLAQSDGALGYMWECPDFFELNGKHVL
MISPQGMEADGDSYNNLFQTGYLVGDYDYDTNEFSHGPFTELDHGHDFYAVQTLLDDEGRRIAIGWMDMWESNMPTK
ADGWCGALTLPREITLGDQLEILMNPVEEITLLRETEHNECKKRPILENYLIDTKEDLIEVKAVFDLTDATARSVGL
KVRGVEQEELTLSYDVEEGQLTLDCSKFGKMKDGVRKAPLNAKDLLSLRIFLDRSSIEVFANNGQTSMTSRIYPKEE
RLGIELFTVNGAVQIDELTYWTLQDTWK
>Pedi_GH32_1
MKIDESEIIKVTNNRYRLNYHLMPTSGWMNDPNGFVYFKGYYHIFYQYYPYDSQWGPMHWGHARSKNLIDWERLPAA
LVPGDKEDEDGCFSGSAIVKDDKLYLIYTGHHYYVKNDPDNFWQNQNLAFSEDGINFTKYENNPIIANPPKDNTIHF
RDPKVWEHNGKYYVVIGSQNQEEVGRILMYESEDLLDWVFKGVIAESKSSKIEGFMWECPDLFRLNGKDILLLSPQG
IESENKKYLNLFQTGYFVGQLDYDQVSYERDKFTELDHGHDFYATQTMLAQDGRRIVLGWMDMWEAKMPEQVDGWAG
ALTLPRELTLVDDHLYMNPVEEVKELRTDKCVDESIKNTKYLLAKLGKKEIELNIDSQLEDWEGNIFTFSMIQKGET
KLSLTFNKDNQELILYRHGEDPNRYANLEKCMRLKLQIFIDRSSIELFINDGEVVFSERIYLIDNYDISLESDSPID
MGIQGYFLKGCE
>Ncir_GH32_2
MKTIDKLLQAEEALQNAKKKINNRYRLGYHIMAPANWINDPNGLVQYKGEYHVFYQHHPYDENWGPMHWGHVKSKDL
VHWEHLPIALAPTEDYEKDGCFSGSAVDHNGILTLIYTGNIFIDQEKDILDQSQCIATSNDGIHFTKEIANPVITRH
PEEGSGHFRDPKVWKHGDYWYMVLGTRKGDIGKAVLYKSIDLRKWEYLGVLAESDGTLGYMWECPDFFELDGKYVLL
FSPQGIEAKGDLYNNLFQTGYLVGEYNYETNEFVHGAFIELDNGHDFYAVQTFLDNKGRRISIGWMDMWESNMPSKE
DGWCGALTLPRELKLGEDNKILMNPVEELTLLRETERTVCKNHILSSVYFVEIKEDLLELRVEFDLAKTSAEAVGLK
ICGINQEKTTIMYQKENKKLVLDCSKSGKEEDGIRSTMLKSDQLLSLRVFIDRSSIEIFANDGEVSMTSRIYPKEER
LGVELFLENGEAQVIDFTYWVLKDIWR
>Sput_GH32_1
MGLEQLKEANEAVQVAEKKINPRYRLGYHIMAPANWINDPNGLVQFKGEYHAFYQHHPYSVNWGPMHWGHVKSKDLV
HWEQLPVALMPGDPCDIEGCFSGSAVDNNGELNLIYTGHRFSDKEKTIPDQVQCRAFSKDGIHFEKDALNPVIPNHP

DAGSGDFRDPKVWKHGGYWYLVAGTRQDKIGKVVLYKSPDLRHWAYQGVLAESDGAQGYMWECPDFYQLDGKYVLMF
SPQGIEPSGDLCRNLYQTGYLVGDYDYKTNVFKHGGFTELDHGHDFYAVQSFEDIKGRRIVIGWMDMWESPMPEKED
GWAGALTLPRELHLSSDRKIRMTPVEELKQLREKELLHLKNERIDGNRMFANLTNDLLELKIEFDGVTSSAADFEIK
LRVGKNEETVIRYDLKEEKLIIDRSKSGLAVAGFRRAAWSKSAQMKLHIYLDRSSIELFAGDGEVVMTSRIYPTEKN
EGVQLFATDGFVKVTDIKAWKLDDIWRD
>Bpum_GH32_1
MLTTNKLQQAEQAIQNAKQQLNKRYRLGFHIMAPANWINDPNGLVQYKGEYHAFYQHHPYDENWGPMHWGHVKSKDL
VHWEHLPIALAPTESYEKDGCFSGSAVDDNGVLTLIYTGNIFVDKEQDILDQSQCIATSTDGITFTKETANPVISKH
PAEGSGHFRDPKVWKHEDSWYMIIGTRKEDTGKVVLYKSQDLRAWQYVGVLAESDGTLGYMWECPDFFELGGKHILL
FSPQGVEAEGDLYNNLFQTGYFVGDYDYKTNAFKSGSFTELDNGHDFYAVQTLLDDKGRRIAIGWMDMWESSMPTKE
DGWCGALTLPRELTLSENNKILMKPVEELSLLRTQQQKVCTDKSLSGSYLVEATQDLIELKLDFDLTKTSAQAIGLK
IRGTNQDETKLEFNVEQQKLVLDCSKSGKGEDGVRHTALKADQQLSLRVFIDRSSIEVFANDGEVTMTSRIYPTEER
LGFELFSESGETEVIDFTYWELEDIWG
>Pmur_GH32_1
MITQNKLQQAQDALNHAIGKVKNKYRLGYHIMAPANWMNDPNGLIQYKGDYHVFYQHNPYDENWGPMHWGHVKSKDL
VHWEHLPIALAPGDTCDTNGCFSGSAVDNNGELTLIYTGHHDVDKETDIIYENQNIAVSTDGITFHKIVENPVIPEP
PADSNTHFRDPKVWKHKDSWYMVIGNSTEDKIGRVILYRSLDLRKWSYVGVLAEGNETLGFMWECPDFFELDGKFVL
LISPQGMKAEKDLYNNLYQTGYLVGDYDYQTNDFTHDSFIELDHGHDFYAVQTLVDDKNRRIAFGWMDMWESDMPTK
ADGWCGALTLPRELTLGKDHKLLMNPVQELDALRNAEHNIIRQQKVADSYLVEVNEELLEIKAVFDLENCHASAIGV
KIRGVNEEETVLTYNLNQQKLTLDCSKSGRVNDGRRTATLEANELLSLRIFVDRSSIEIFANEGQTTMTSRIYPIEE
KLGIEVFTENGEVRIKELTYWSLKDIWE
>Bcru_GH32_1
MTSTPAETTRNDTSEADEWISTHKESINERYRLGYHLMPPVGWMNDPNGLVYFKGLYHAFYQHHPYTSEWGPMHWGH
ATSTDMVHWVDRPVALAPGDGSDRDGCYSGSAVVEGDTLNLIYTGQIFNTGDIEPFNSDFSESQNLAISRDGTHFEK
YAGNPVIPGPPSDNTQNFRDPKVWKHNDTWYVVIGSTSAEDDARALLYSSPDLHTWQLEGNLAQSDHKIGNMWECPD
FFELDGMHILMMSPVGLEADGHRYRNVFQSGYIAGDFDYQTLQLTHGDFEELDRGHDFYAPQTFEAPDGRRICFGWM
NMWLTPMPEQKDGWAGAFTLPRELKYVDGKVTMTPVKELESLRGSVLTDCERSVTNGDVIVSPDENRFELLFSVDNL
QVHADNLGLAFDLGNGIKASFVYDSGQELLTFDRGGKDGERSWECGTLEHLDLHIYVDNSSIEIFVNNGLATFTSRI
YPTAPARVTLLSSTDSVKAGIQSYALN
>Pmeg_GH32_3
MNKIEQAQHSLNEAEKKVNHQYRLGYHIMAPANWINDPNGLVQYKGEYHVFYQHHPYDENWGPMHWGHVKSKDLVHW
QHLPIALAPGDSFDKDGCFSGSAVDNEGELTLIYTGHNYIDKELDTFFQNQNIAVSKDGITFEKAEANPVIAEPPAD
SSHHFRDPKVWKHEGFWYMILGNSTKKQEGRVILYRSSNLQKWEYVGVLAKSDGDLGYMWECPDFFELDGKHVLMIS
PQGIEAKGDSYHNLFQTGYLVGEYNYETNTFHHGSFTELDYGHDFYAVQTLLDDKGRRIAIGWMDMWEANMPTKEDG
WCGALTLPRELTLRGDKVLMNPVQELTSLRKTQHNMLTNKALSNSYVVEVNEDLLEIRAVFDLADCQASSVGIKIRG
INNEETLMCYNLNEQKLLLDCTHSGKEDGVRKVALQAGETLALRIFVDRSSLEVFANEGQATMTTRIYPKESRFGIE
LFTEGGNVIVKELTYWNLKDIWG
>Mfor_GH32_2
MLTTNNRLQHAEEALQKAKEKLNERYRLGYHIMAPANWINDPNGLVQFKGEYHVFYQHHPYDENWGPMHWGHAKSKD
LVHWEHLPVALAPGDACDKDGCFSGSAVDNDGELTLIYTGHHYTDKEKDLFTQNQNIAVSRDGITFEKAGENPVIAE
PPADSAHHFRDPKVWKHGDSWYMILGNATKDEVGRVILYRSADLRNWEYIGVLAQSEGTLGYMWECPDFFELDGKHV
LLISPQGLEAEGDLYNNRFQTGYLIGEYDYETNKFSHGEFIEMDNGHDFYAVQTLKDDKGRRIAIGWMDMWESNMPT
KEDGWCGALTLPRELTLDKSNKVLMNPVDELTLLRETEHKVCANQSISGNYLAKTNQDLIELKVEFDLTKTTAEAVG
FKIRGLDQEETLLRYQIKDQKLVLDCTKHGKMEDGIRRTALEAGDRLSLRVFIDRSSIEIFANEGQATMTSRIYPQE
ERLGIELFAEDGDVQVSEFTYWSLKDIW
>Bhal_GH32_1
MDRIQQAEEALNKAEEKLNSRYRLGYHIMPRANWINDPNGLIHYKGEYHVFYQHHPYDENWAQMHWGHVKSKDLIHW
EHLPVALAPGDSFDESGCFSGSAVDFNGKLALIYTGHIVIDQEKDIFYQNQNIAVSQDGIVFEKIQENPVIAKPPED
SSRHFRDPKVWKHRDSWYMVVGNSTKENVGRVILYRSPNLRDWEYKGVLAQSDGDLGYMWECPDFFELGGKHVLLIS
PQGIEADGDLYKNLHQTGYLIGEYNDETNEFVYGAFTELDHGHDFYAVQTLLDDKGRRIAIGWMDMWESEMPTKADG
WCGALTLPRELTLRDDHKILMNPVEETKLLRESNHLECTNQLISGNYLAKTAEELLEIQVVYDLTDCSAKSVGLKIR
GLEEEETAIKYSITDQKLSLDCSKMGKSRDSVRNAPLEANGKLTLRIFIDRSSIEVFANHGEITMTSRIYPKEGRLG
IELFSEKGAVRVEEFTYWTLKDIWKEDESLEKNILHR

>Lani_GH32_1
MKQMIKLNNDRYRLGYHVSAPAGWINDPNGFCYYKGYYHIFYQYHPYSADWGPMHWGHARSKDLVHWESLPIALAPD
TKADEDGCFSGSAIVKDDVLYLIYTGHHYYDDGDPDHFWQNQNLAYSTDGINFTKYENNPIIASAPEDNTHHFRDPK
VWEKDGKYYMILGSQGKDGVGRAITYRSDDLKDWQYLGVIAKANGLTTEGFMWECPDFFELAGKDILLLSPQGIEAQ
GQKYLNLFQTGYFVGNFDYSTNTFEHGGFTELDHGHDFYATQTTLAPDGRRLVFGWMDMWESEFPEKADGWAGALTL
PRELELKDDQLYMRPVKEAVQLRTAEISAWNKQVTEKTLLCENEQQAEIDLTLTTDQAFELAFTDQDKQVKLTFDQA
THTFTLLNGDARYASIKPNAEFKLQIFIDTSSLEIFINDGEAVFTERFHFDNAPQVWLTAKAQCLTQVYHLDGQAIT
FE
>Pmeg_GH32_4
MLTVNKIEQAQHSLNEAEKKVNHQYRLGYHIMAPANWINDPNGLVQYKGEYHVFYQHHPYDENWGPMHWGHVKSKDL
VHWQHLPIALAPGDSFDKDGCFSGSAVDNEGELTLIYTGHNYIDKELDTFFQNQNIAVSKDGITFEKEEGNPVIAEP
PADSSHHFRDPKVWKHEDFWYMILGNSTKKKEGRVILYRSSNLRTWEYVGVLAKSDGDLGYMWECPDFFELDGKHVL
MISPQGIEAKGDSYHNLFQTGYLVGEYNYETNTFHHGSFTELDYGHDFYAVQTLLDDKGRRIAIGWMDMWEANMPTK
EAGWCGALTLPRELTLRGDKVLMNPVQELTSLRKTQHNMLTNKALSNSYVVEVNEDLLEIQAVFDLADCQASSVGIK
IRGINNEETLMCYNLNEQKLLLDCTHSGKEDGVRKVALQSGETLALRIFVDRSSIEIFANEGQATMTSRIYPKESRL
GIELFTEGGNVIVKELTYWNLKDIWG
>Ncir_GH32_2
MKTIDKLLQAEEALQNAKKKINNRYRLGYHIMAPANWINDPNGLVQYKGEYHVFYQHHPYDENWGPMHWGHVKSKDL
VHWEHLPIALAPTEDYEKDGCFSGSAVDHNGILTLIYTGNIFIDQEKDILDQSQCIATSTDGIHFTKEIANPVITKH
PEEGSGHFRDPKVWKHGDYWYMVLGTRKGDIGKAVLYKSIDLRKWEYLGVLAESDGTLGYMWECPDFFELDGKYVLL
FSPQGIEAKGDLYNNLFQTGYLVGEYNYETNEFVHGAFIELDNGHDFYAVQTFLDNKGRRISIGWMDMWESNMPSKE
DGWCGALTLPRELKLGENNKILMYPVEELTLLREAERTVCKNHILSSVYFVEIKEDLLELRVEFDLAKTSAEAVGLK
ICGINQEKTTIMYQKENKKLVLDCSKSGKEEDGIRSTMLKSDQLLSLRVFIDRSSIEIFANDGEVSMTSRIYPKEER
LGVELFLENGEAQVIDFTYWVLKDIWR
>Psim_GH32_1
MLQKSRTNRAEEALSEAKGKMNDRYRLGYHIMAPANWINDPNGLIYYKGEYHVFYQHNPLDENWGPMHWGHVKSKDL
VHWEHLPIALTPGDACDTDGCFSGSAVDNDGELTLVYTGHHYTDKEKDIFYQNQNIAVSKDGITFEKASENPVIDHP
PADSAHHFRDPKVWKHDDTWYMVLGNATQDKVGRVILYRSPDLRHWDYRGVLAQSDGTLGYMWECPDFFELNGKHIL
MISPQGIEAEGDAYNNLFQTGYLVGDYDYDTNEFSHGPFTELDHGHDFYAVQTLLDDEGRRVAIGWMDMWESNMPTK
ADGWCGALTLPREITLGDQLEILMNPVEEITLLRETEHNECKKRPILENYLIDTKEDLIEVKAVFDLTDANARSVGL
KVRGVEQEELILSYDVEEGQLTLDCSKFGKMKDGVRKAPLNAKDLLSLRIFLDRSSIEVFANNGQTSMTSRIYPKEE
RLGIELFTVNGAVQIDELTYWTLQDTWK
>Eger_GH32_1
MTYTIAQAEQELQAKCEKLNLRWYPRYHLAARAGWINDPNGLIWFDGWYHAFYQHHPYSTQWGPMHWGHARSKDLLH
WEHLPVALAPEGPEDKDGCFSGSAVIDGDTLALIYTGHKFRGDVSDDENLYQVQCLATSRDGVHFTRQGMVIDTPAD
LHHFRDPKVWREGDNWYMVVGARVGDTGQIRLYRSADLRQWQDEGILDEAEAGMGFMWECPDLFSLNGKRVLMFSPQ
GIAAQGFENRNLFQSGYLVGDWQPGEPFVRAGEFVEMDHGHDFYAPQSFLTPDGRRIVIGWLNMWESPMPEQQDGWC
GMLSLPRELTLNENNHLQMRPAKEVEALRQAWLPWPASSLKNQQTLMANKAEAMEVVLHWDCTSSDAEQYGLSLGEG
LRVYVDTQMQRLVLERRYPQYGLCGTRSVPLVPGAPLALRLFIDSSSIEVFVNDGEACLSSRIYPDADRRQLSLFAW
HGTAILSEAGAWQLE
>Bint_GH32_1
MTAIWRPGSALLDAVAASQGRTFARYLPLAHPAPTPYHTRAYASQHAGKFPADVAAFEKTQPQRWVKPWFNDPSCLV
WDEKNQIYRGFSISQSNPDSQGGDQQTWMEIITPDLVTFINNRSPFYLDNLPYPALWGGSFLIDQHNAAGYGAGAVL
YYISVPGSDTSSLQCVSLWIAPALGLAPVYHGIVLENPGVGAIVYAPGMDFRDPRVSWDDARSRFVMKLTIGRGIAF
YSSTDGLNWSFLSLIDLSDWQQIETPDLVPMEAPDGSEKWLLAFSIKQWNGQAASSVAYLIGDWDGTTFKPDFTTPK
RLNWGSDYYAQAISQHEGNTYCWGWMGNWNYMELPQQGFGGNHSLITRLSLAQDVDGSLGLRMQFMPDQLNCYAEFT
DAILSLPLSSSGTSQWQPPVQNMGVSWRLDLQLYRDAPDPWPDAISINFCVGPKNRTTLTLNPQAGTVTLLRAQSGG
GPLDTADTKKWAIWNADQVATLPNRARYFISIIVDVSTIEIIINDEVYISSLFFPPEDAFSSDVTVSGNGTARLLYF
KQSY
>Bbom_GH32_1
MTQNPIQLTNSRYRLGYHLMAPAGWINDPNGFCYFKGYYHIFYQYHPYSAEWGPMHWGHARSKDLIHWQTLPTALIP
GDSEDKDGCFSGSAIVKDDTLYLIYTGNNYYDDGDPDHYWQNQNLAYSQDGIHFTKYDKNPIIATPPTDNTQNFRDP
KVWEHDGHYYVALGSQDQAKLGRLLLYKSDDLKQWQYLGPIAHSQSVEQEGYMWECPDLFHLNGQDILVTSPQGIAA

QEQQYLNLHQTGYFVGELDYQAPKFKRGDFHELDQGHDFYAAQTMLTPDGRRVLIGWLNMWESEMPEQVDGWAGALT
LPRELIYQNNHLYQMPVAETKSLRQQKLRDEQLMVQSETELVNNQSQVEINLQLDSTKTTAQHFVVQFTDPQTHANV
QLEYDFAQQLFTLKRSDRSDARFAQLRSNEKLQIQIFVDTSSLEIFLNQGESTITERYYFDHAPQISLTSDQDLAFN
CQIYQLEKQSNNYQINE
>Luva_GH32_1
MNDPNGLFYDEKTGLYHLYYQYNPEGNEWGNMSWGHATSKDMVNWTQEQLAIPMLDNQGWEDFTYTNTTGNLAKYGE
VRYVGVPTTNWGDSNGKKAIFSGSIYVDKDNISGLGNGAILAFYTADYQIATRINDGEDNGWGTWIGLSEIQEQHLA
YSLDGGTTFIQYSSDGNSAEPKPLIPVTASEGGDAANFRDPNVVYDEQNKQFLMTVVSNQQALIYKSQDLLHWEYAS
SIQRQKNVGAGVWECPTLIPMTVSGTNITKWIFAVSVQQGAHATGSGMEYYVGNINANGEWLPESKKTMNSPMTFDY
GEDFYAGIPFANMKDKRNVLIAWQSNWSYTGDAKTSPWYGNMTLPRELQLVKSSEGTDGYLLKNTVVSEIKNNEQEN
VISQSKANLTIENNEQKIEYEGNQYKITAKFSWNAENQPESVGFKLRVSDDGKYYILVGYDLKTQKFFVRRLNTGEP
NMGDPRDQMNAFVNTDNGTITLTVYVDETSIEAFANEGEKTITQNFFLRPEYIGAQATNQLYLYAQNGTAQITDLTL
NPLASIW
>Malb_GH32_1
MTFKTALPALGLGALLLSGCATPPPDAVYREHHRPQLSFSPERNWMNDPNGLVYHDGEYHLFYQFNPSGNAWGDMSW
GHAVSPDLLNWSELPPALPVEKDAKGAITQMFFSGSAVVDHANASGLGQPGKPAMVALYTAMFPQARTLAGKTIQAG
TQAQSLAYSLDRGRSWTQYPGNPVIELPPAPYAAEYRDFRDPKLFWHEPQKKWVMVAVLPNLHKALFYSSRDLLKWE
LMSEFGPAGSVSGIWECPDLFELPVDGDPARRKWVLVMSQNPGHPAGGSGTQYFVGDFDGTRFTWDRAASDGQVQWL
DYGADFYAGVTYNGVPDGRRLLVGWMNNWLYGQQVPTTPWRGAQSVPRELSLATVDGKIRLVQQPLAELKRQRAQRV
AELASAQVAPGVLPVQSSSAAGDALEIELRLQPGSARYSGIRVQAHGGQYTEVGYDREKGTVYLDRSRAGQAGFHAD
FAARHHAPVALRSGQLPMRILVDGGSVTVFAGQGEAVLTDQVFPGRTSKDMALFSEGGTAGVTGLSAWSMKPVQLKP
ANATEGAGR
>MTW1_GH32_1
MALISRLPLLTIGAALLSAGCASGPHDPAPVHDEPFRPQLSFSPQRNWMNDPNGLVYHDGEYHLFYQYNPNKDTWGD
MSWGHAVSADLLHWTELPLALPVEKDARGEITQMFFSGSAVVDHANTSGFGQPGNPAMVAMYTAVFPQARTLNGQQI
RAGTQAQSLAVSLDRGRTWTQYAGNPVIPAPPGPYAAEYREFRDPKVFWYEPEHKWVLATVVAQQHKALFYSSRDLI
HWEWTGEFGPAGAAGGVWECPDLVELPVDGDPARRRWVLIVSINPGGPAGGSGMQYFVGDFDGKTFTRDRNVASDDV
RWLDYGADFYAGVTYNDAPGNRRLLVGWMNNWQYAGTVPTAPWRSAQSLPRELGLRTLDGQVKLVQQPLAQVQALRS
GLLYSVPARAIAPGVQAVSLNGAANAPLEVRMRLQPGTAARSGIRLGTAGTSAYTEIGYDAAQRAVYVDRTHAGDAR
FHPQFAARHAAPVVLRDAELPLRILVDRGSVTVFAGAGDTVLTDQVFPPPGPAAVSLFGVDGDAAVRDLDVWSLNSI
WKDVQ
>Obru_GH32_1
MALRSLVILLCVFAAAQPKILKQSSYAKEELEQYIANTRPELNLRYRLHYHVAPPVGWMNDPNGFSYYKGEYHLFYQ
FYPYDSVWGPMHWGHSSSPDLVNWKTLPTALLPDQEQCFSGSAIVDGDTMILMYTGHEITDKEPYYNETQFLAFSDD
GVAFTKYAGNPVLPAAPNGSPDFRDPKVWKHGKHWYVVLGSKTDDNRGRVLLYRSFDLIEWEFLRVLGESTGDLGYM
WECPDFFELGGKHVLLWSPQGMEPNGDRYKNLHQTGFIIGNFNYNNFEFVQETKFQELDYGHDFYASQTMEKDGKRY
VVAWFNMWEVPHPEEVDGWAGAMTIIRELVLVGDRVLQKPLDKMISLRDESVINGQVDENQVIELGHTGEIIISGDL
EKKIELLVEGRNGGGQALLRWDPEVGKVVVDRAGDVRQVEWSPIGSHSWRLFLDASSLELFCGEGEVVFSSRVYPDG
EWNLTNLSPQTLNVEAYKLKRSVPL
>Eger_GH32_2
MTYTIAQAEQELQAKCEKLNLRWYPRYHLAARAGWINDPNGLIWFDGWYHAFYQHHPYSTQWGPMHWGHARSKDLLH
WEHLPVALAPEGPEDKDGCFSGSAVIDGDTLALIYTGHKFRGDVSDDENLYQVQCLATSRDGVHFTRQGMVIDTPAD
LHHFRDPKVWREGDNWYMVVGARVGDTGQIRLYRSADLRQWQDEGILDEAEAGMGFMWECPDLFSLNGKRVLMFSPQ
GIAAQGFENRNLFQSGYLVGDWQPGEPFVRAGEFVEMDHGHDFYAPQSFLTPDGRRIVIGWLNMWESPMPEQQDGWC
GMLSLPRELTLNENNRLQMRPAKEVEALRQAWLPWPASSLKNQQTLMANKAEAMEVVLHWDCTSSDAEQYGLSLGEG
LRVYYVDTQMQRLVLERRYPQYGLCGTRSVPLVPGAPLALRLFIDSSSIEVFVNDGEACLSSRIYPDADRRQLSLFAW
HGTATLSEAGAWQLE
>Mgos_GH32_1
MKTKCGILMLIAFTIVNGICKAQDSPQKTYTEQYRPQVHFSPAAHWMNDPNGMVYYNGTYHLFFQYYPKDIVWGPMH
WGHAESKDLIHWKQLPIALYPDSLGYIFSGSAVVDANNTSGFGKNGKTPLVAIFTHHDPKGEKEGTDKFQNQSLAYS
LDGGSSWTKYSGNPVLKNPGIKDFRDPKVMWYAPEKKWIMTLATQDHISFYSAPDLKNWKKESEFGLKEGAHGGVWE
CPDLFTLKMNGKTYWILIVNLNPGGPNGGSATQYFVGNFDGNKFSPLDSSTRWLDYGPDEYAGITWSNTGNRKIFLG
WMSNWQYANQVPTKTWRNAATIARELSLQQSKGQILLASRPVAELKNIETKPIALSQLTVNGSLDLSSKINKLKSRY

VLKLSLNTLKDYGIRLSNAKGEEVLIGYDQTKEQYYIDRTKAGKMDFQKDFSGRFTAPRLSTAKNSDLTLVIDKSSV
ELFADGGLTTMTTIYFPTEDFSKLAIKTNGKLMIDNLSISGLKSIWK
>Basb_GH32_1
MPYGEQHRPQFHFSPPAKWMNDPNGLVYYHGEYHLFYQYYPGNTVWGPMHWGHAVSKDLVHWDNLSVALYPDPLGYI
FSGSAFIDGKNTSGLQTGKHPPMIAFYTHHDESRKKEGRKDYQNQSLAYSKDMGRSFIKYKYNPVILNPGEEDFRDP
KVIWNENLHIWILILTAGKKVKFYRSANLLQWEYVCDFGSGTGEQGGVWECPDLFPVKSENGTKWVLVVSIVQGAPN
GGSGTQYFIGDFDGRNFINDNTDDTTLWLDYGPDNYAGVSWSDIPGEDGRRIFLGWMSNWNYAEKVPTDPWRGAMTI
PRSLALKNTRNGLRLTAEPVKELENLRRNKHNLEWNTDVVIQLSGLNEIIMTANLIKTTAEDFGFIFFNRLDEKLVV
GFNRLSNQFYIDRTGSGKTNFSFAFPGRFYAPRITNDSGFKWHLFLDRASLEFFADNGLVSMTEIFFPNENIDRVSF
FQYKGKVKIQACTLYELKSIWQTDNT
>PBS3_GH32_1
MIKAAGLTFIVAVMAACSSLKNNRQEYKEPYRLQFHFSPKAKWVNDPNGMVYLNGKYHLFFQYYPDSTVWGPMHWAH
AVSKDLMHWEQLPIALYPDSLGWIFSGSAVYDKDNTSGFAKDGKMPLVAIFTHHNPKLEKAGRKDFQYQSIAYSLDE
GTTWTKYAGNPVLKNPGITDFRDPKVIWYAPGKKWVMTLATKDHVTFYSSPDLKNWTKESEFGQQSGAHGGVWECPD
LFSIKAGGKEHWVLLVSVNPGAPNGGSGTQYFIGNFDGHTFTSTSATTKWLDYGTDNYAGVTWSNTGDNRIFIGWMN
NWQYANRVPTQVWRGATTLPRTLELREVDGAYFLSAMPVNTFNTLRQKEVRWQNIRVNDSLDISAKVKNFKDKFVLQ
FKTTTLADFAIKLSNTTGEQLIVGYDKQTNSYYIDRTKSGETSFETGFAKKIVAPRISQSGNITGKLVIDAASAELF
ADDGLTNQTAVFFPGKPYTALTLLAGNLNMETLSYAPINSIWP
>DCY312_GH32_1
MKKIIICLFLIAALAGSRATAQEFKEKYRPQFHFTPKANWMNDPNGMVYHNGIYHLFYQYYPNDKVWGPMHWGHATS
KDMISWKEQPIALYPDSLGYIFSGSAVVDKNNTAGFGKDALVAIFTHHDPVQEKQKTGKHETQSIAYSLDDGKTWTK
YKGNPVVKNPGISDFRDPKVSWFEAQKKWVMTLAAKDHIAFYSSPDLKSWTHESDFGANEGGHGGVWECPDLFPIKH
EGKDIWVLIVNINPGGPNKGSAGQYFLGDFDGKTFMSNSKETKWLDFGTDNYAAVTFANTGNRNILMGWMSNWQYAN
QVPTDPWRSANTIARELGLKTVGKEIYLTSAPVKELDVLNTTGFSLKTVKVKDQLDLTEKAKNKTGLFRLDLTTKNT
ADFSIVLANEAGNELVVGYDKAKNQYYIDRTKSGKIDFEKGFGEIHIAPRFATGSIIPLTLIADVASVELFADNGLT
VMTDIFFPETPMSKLHIKSATGITVDNLKYTVLKPSME
>Mseg_GH32_1
MNLLKLPLIGCLMLAAGAVCSQTPAKVYHEGYRPQVHFSPKAKWTNDPNGLVFYKGTYHLFFQHYPDDIVWGPMHWG
HAVSTDLIHWKQMPIALYPDKLGYIFSGSVVVDENNTSGFGKNGQTPLVAIFTHHDPEGEKSGSNTFQNESLAYSLD
AGQTWTKYAANPVLKNPGIKDFRDPKVFWYAPQHKWVMSLATKDRITFYSSKNLKNWTKESEFGKTVGAHGGVWECP
DLISLQLNGKQYWVLLVSINPGGENGGSATQYFVGEFNGHTFTPVSTNTKWIDYGPDDYAGVTWSNTGNRKIFLGWM
SNWIYANQVPTKTWRNAMTIPRELSLKQVGSDIFLTGTPVKELGRIASKSLRINPATAKLPAQYTLKFDVTKINDYS
LEFSNDAGEKLIIGYDRQKSRYYIDRSRSGEIAFNKEFVNMAYAPRLSASSTSTVELVMDASSVELFADGGLTIMTG
IFFPSKTYTHLKVVSNQADIKNMVLTPLSSIW
>Cfla_GH32_1
MKSITKLAFVSVALLLVSCKDKSQATSENSDTTKVDTVMHEAYRPQLHFSPKEKWMNDPNGMFFLDGEYHLFYQYFP
GGTKWGPMHWGHAVSKDLIHWEHLPIALYPDSTGYIFSGSAVVDHHNTSGLAKNGEIPIVAYYTIHDDKKAKAGRVD
YQTQAMAYSLDKGRTWTKYDQNPAISNPGIIDFRDPKVSWNSKSKQWVMTLAVKDHISFYGSTNLKQWKKLSDFGLN
EGDHGGVWECPDLLTMKDEHGVEKNVLIVSINPGGPNRGSATQYFVGDFDGKTFKNDTPGKNSGWVDYGPDNYAGVT
FNNIPKEDGRCIMIGWMSNWFYAELVPTEKWRSAMTIPRVLTLRTIGKNHVVQSLPVVEIEETIESTGKISTREVDT
LDITAEAKVDVRLSRIQGRIKKANFVFELSNAKGEKILFGLDDVGGKFFVDRSASGKVNFHPDFKLGIQAPRVRDSE
WVEFTAIVDVASIELFFDQGATVMTAIYFPNDGYSKLKLYGKNGKVSTDAIVVDELKTIW
>Saeb_GH32_1
MKNLFLLTIFISSLHTSFAQRPESWYNEPHRPQIHFSPEANWMNDPNGMVYYDGEYHLFYQYYPDSTVWGPMHWGHA
VSTNLTHWTHLPVALAPDKHGYIFSGSVVVDKNNTSGLQKGKEAPLIAMFTYFDPEKNKAGTNDDQTQGIAYSNDRG
RTWTKYSGNPVIPNTEKIKDFRDPKMFWYEEGGYWVMVLAVGDHVRFYKSDNLKNWTLTGEFGKKEGSHGGVWECPD
LFPLKVENSKTTRWVLLVSLGTGGPNGGSATQYFVGTFDGKTFVNDNKAEDIMWIDYGRDNYAGVTWSNTPDDDRIF
LGWMSNWQYAQVVPTSAWRSAMTLPREMELEQTPAGIRLVQEPVEQVKKLRGKPYGNLKSWVSGKDAGGLRIDGPVE
IELEIDLENSVATELGMELSNARGEVFVLGYEVATKRYFTDRTKAGKHAFSPDFAKTRHYAPRTATENELEWHLFID
RSSLEWFADDGETVMTELFFPSEPFTAVTFFSKGGALKIEEVEAWPLKTIWK
>SKUNC_GH32_1
MRNSLATLALVALLGHYSAGQSTQADMKSEQYRPQFHFSPKAHWINDPNGMVYYKGTYHLFYQYYPEASVWGPMHWG
HTTSKDMVHWQEQPIALYPDSLGYIFSGSAVVDANNTSGFGKNGQVPLVAIFTHHNPKLEKEKPEQVQSQSLAYSLD

EGKTWTKYAGNPVLPNPGITDFRDPKVRWYEPQKKWIMTLATKDRVTFYSSPNLKAWSKESDFGSDAGAHGGVWECP
DLFPLKHNGKDVWVLIVNINPGGPNSGLAAQYFLGDFDGKHFNAYAKKTKWMDYGPDNYAGVTFSNTGNRTILMGWM
NNWQYANKVPTPSWRGATTVPRELGLKDVNKELYLTSSPVKELDALAGKTTSLSNLTVKGSSDLTAKTGGKYNTFKL
ELSAPATNDFSIVLANEQGNELTIGYDKAANSYFIDRTKSGKTDFEKGFGKRSTAPRLSTDKTISLTLLVDVASAEL
FADDGLSIMTAVFFPDKEFSKLSIKSATGISLNKLAFTKLSSSVQ
>Rsli_GH32_1
MKKIVQFAVLFIWLAACQKKQENRADYSEQYRPQFHFSPQKGWMNDPNGLIYHAGEYHLFYQYYPDSTVWGPMHWGH
AVSTDLTHWQHLPIALYPDSLGYIFSGSVVLDSANTTGFGKDNEAPLVAVFTYHNMEWEKAGRKDRESQGIAYSLDK
GRTWTKYTNNPVLKNQGDVDFRDPKVFWHAPTKRWIMPLATGDFLQIFTSTNLKSWEKASEFGKNEGAHGGVWECPD
LFAITTPAGIKKWVLIQNMGRGAVNGGSGTQYFVGSFDGKIFKNDNPPTQTLWLDYGADNYAGVTWLNAPNNRRLFI
GWMSNWDDYANKTPTHPWRSAMTVPRELSLQKTAEGFRLFQMPVTELEKLRQGEALTLENKNIDSTLKISAESVQKE
VLVEFDLSKTTATQLGFVLANAKNERVEIGYDHLKKQLYLDRTQAGKSDFSTKFAKRHTAPYVAGSRLVVRALIDNS
SVEVFVDNGRIAFTDLFFPNQDFTQLTLFAKGGSAHLAQVKVYELRSIWQ
>FQH1_GH32_1
MTKNSTIHTLCLLLTVVVLVSCSRKEDKPAVVSSTSAFYNEQHRPQFHFSPKEHWMNDPNGMVYYQGEYHLFYQHYP
EGNTWGPMHWGHAVSEDLIHWKHLSIALFPDSAGMIFSGSVVVDHQNVTGFGTADNPPLIAIYTVHNMEGEKSGRND
YQNQAIAYSADRGRTWTKYNGNPVLKNPGKKDFRDPKVFWHAPSQAWIMILAVGDHVELYRSTNLTSWTKSGDFGID
QGSHGGVWECPDLFPLQVDNGKQKWVMLVSLSDGGPNGGSGTQYFVGNFDGTGFTNDNPKDKILWIDYGRDNYAGVT
WSNIPEGDGRTIFIGWMSNWKYANVVPTTVWRSANTLPRTVQLKETGDGLRLTSMPVSEFDSLALTSNEVTQQNISD
SLDLSSTITALSTYELDVEISNADKTGFVIELQNEVNQKVVFGYSPSEGNRYYVDRLMAGKNDYSLNFPGKHYATRI
SSDTTVKLRMIVDRSSIEIFADGGLTVFTDLVFPDRVFSKLKIKSQGKPITLKSANVTAIDTIWKVE
>Hisb_GH32_1
MHAALILIVILSIAACSDQKTKTREVSYYNEPYRPQFHFTPEAGWMNDPNGLVYYKGEYHLFYQYYPDSTVWGPMHW
GHAVSTDLVHWKHLPIALFPDSLGYIFSGSAVVDENNTSGFQTGDEKPMVAIFTYHNMQYEKAGRTDRESQGIAYSP
DRGRTWTKYSGNPVIKNKGDLNFRDPNVFWYEPGKHWVLALAVGDHVEFYASPDLKNWKLTGEFGKGQGSHGGVWEC
PNLFPMQTPEGEKWVLLQNMDRGAVSGGSGTQYFIGRFNGTTFINENDSSEVLWFDYGADDYAGVTWNHAPDGRSIF
IGWMSNWNDYAQKVPTQTWRSAMTVPRELSLKKTDDGYRLFQLPVKELEILRNDTILMNAQIINDSTQFKITSSVQK
DIELVFDVSKSTAENFGLILSNSKNEFVKIGFDAAKSEFFIDRIHSGNTGFSPLFAAKHVAPYRPGNMLSIRALVDV
SSVEVFVDDGKLAMTEIFFPTEDFNQVRLYSENGNAELSGGVIYGLKPAW
>Acab_GH32_1
MKRTIFWLMLILTLKAAAQYDEPHRPQLHFSPPANWMNDPNGMVYADGEYHLFYQYYPNATVWGPMHWGHAVSRDLV
HWENLPVALAPDKLGLIFSGSAVLDARNSSGFGTAKKTPLVAMFTYHDMDGEKAGRTDFQYQGLAYSLDKGRSWTKY
DKNPVIPNTEKLKDFRDTKLFWNEALKQWSVVFAVGDHVRFYSSPDLKSWTKTGEFGKSDGTHAGVWECPDLFPLTV
GGRTKWVLLVSINPGGPNGGSATQYFVGDFDGKTFRNDNPPQNIFWLDQGRDDYAGVTWSNAPRGRRIFLGWMSNWD
YAQQVPTAKWRSAMTLPRELGLAETAEGVRLVQNPVRETQALRGRKFLSMKNYRLENEMNLDNGGSPLAELELEIDL
AKTPATKFGVALTNAKGETYRVGYDAEAKRFFSDRRKAGRNDFSDLFAVKPHTAPRVVTDQTLRLHLIFDTASMELF
ADDGTVAMTDIFFPTEDFTRLKFFGLKSASQWNVPESVFFTQANAWNLKSIWR
>Cyeb_GH32_1
MRLLQNLFLIALLTTVISCSKKEPETATIIEPHRPQFHFSPPAKWMNDPNGMVYYNGEYHLFYQHYPDSTMWGPMHW
GHAVSKDLIHWEHLPIALYPDSLGYIFSGSAVVDANNTAGFQSGNEKPLVAIFTYDKQGYETQAIAFSNDKGHTWTK
YENNPVIKNPGEKDFRDPKVFWHVESEHWIMSLAVANRIQFFRSKNLKDWELTGEFGREHGNHGGVWECPDLFQLPV
TGTTETRWVLLVSINPGSPNGGSGTQYFIGNFDGKTFTSEHGPSTERFIDYGRDNYAGVTWGNAPDGRTIFLGWMSN
WNYAQVVPTETWRSAMTLPRDLSLHNTSAGIRLVSTPSKEVEQLRTERKDITVDQPFIHTGLAEVKVEFDLNETAAK
DFGIELFNSKNENIRIGYDRESNWFYIDRTNPGKKEFSTDFVGIQYAPRFSDSHILTLHIFVDVASVELFADGGVSC
MTAIFFPTEDFTQLKIYGDGEVKLLSTEAYELKSIW
>Fdor_GH32_1
MKKKISTCNVTQEIKYKISGKSLIINNRLLEITFLSFFLMLIFNSNSNSQSKEAHRPEFHFSPQKMWMNDPNGMVFY
QGEYHLFYQHYPDNTVWGPMHWGHAVSKDLIHWEHLPIALFPDEHGYIFSGSAVVDHENTSGFGTKDNPPMVAIFTY
HDAEKAKKGSIDFQTQGIAYSLDKGRSWTKYENNPVLNNPGIKDFRDPKVFWHGPSQKWIMILAVQNHVQLFNSSNL
KDWSFLSKFGENSGSHGGVWECPDLFELSVDGKKNEKHWVMLVSINPGGPNGGSATQYFIGDFDGEKFTNSNSSETS
LWVDYGKDNYAGVTWSDIPKKDGRRIFIGWMSNWQYANQVPTEKWRNAMTIPRKLGLTETNDGVRLQNFPVQELKKL
RTDKLNLKDLEVEGKYSISEEKNWTSNTFELDLKIALEKSTGENFGIELSNSMGEKILIGFDQNQRQCYIDRRFAGK
SDFSEKFKGKSTAPRIKSSNELKIRIIVDVASVEVFFDDGTIAMTEIFFPNEDFSTISFFSEEKKMHIDSAVLYKY

>ECRI_GH32_1
MKISACLFLCLFITIQVLAQNSPSADYREEHRPQFHFSPPAKWMNDPNGLVYYEGEYHLFYQYYPEATVWGPMHWGH
AVSKDLLRWENLPIALFPDKHGYIFSGSAVIDSKNTSGFKQGPNAPMVAMFTYHLMEGEKAGKSDFQTQGIAYSNDK
GRTWTKYEGNPVIRNESGIRDFRDPKVFWHEASGQWVMILAAGDRAHIYNSPDLKSWTKASEFGPGQGAPGKPWECP
DLFELTADGKSRWVMLVSLGNGAPNGGSGTEYFVGTFDGKTFKNDNPASTTLWVDHGTDNYAGVTWNNAPYGRRLFI
GWMSNWAYSQKVPTEKWRSVMTLPRELSLFKSGNLTLLASKPVKEVEKLRKSRFVLDMKKPYHATSALQEVLLEIDL
SKTTSTDIGLEISNKKGEKIIVGFDVKAGQFYIDRTEAGKRTFSDVFAARHTAKRVSGSNIMKMRLLLDTSTAELFA
DDGSVVMSDIFFPNEDFKTLKLLQNTGSLKVIKAEGFELKGTW
>Cysb_GH32_1
MRFNQITCLSSIAVLLVMESCQEKKTSSNKIAAVKNTALYQEAHRPQFHFSPPANWMNDPNGMVYYQGEYHLFYQYF
PDSTVWGPMHWGHALSKDLIHWENLPVALYPDDSGYIFSGSAVVDENNTAGFQTGSEKALVAFFTHHNPKNELQVQS
LAYSNDKGRTWQKYAHNPVLANPGIKDFRDPKVSWYAPAKRWIMTLAVQDRVHFYSSPDLKNWQFESKFGEGNVGAH
GGVWECPDLFPLTVAGKQKWVLLVSINPGGPNGGSATQYFIGDFNGKTFKNENAPETTLWLDYGTDNYAGVTWSGVP
STDGRRLFIGWMSNWLYGPTVPTANWRSAMTIPRALTLQNTPAGIRLANTPVKELQKLRTSSQVIKSQPITGNLDLS
KTLDLKTSLLELDLNFDLAKSENVEVNISNQKGEHITIGYSVPEKQLYINRSKSGQTKFDKNFAAKHTAPLLLENGK
LKLHLFLDVASVEVFANNGTVVMTDIFFPNQDFTQVELTAKGTGILAESHAYSLQSIWK
>Llut_GH32_1
MKKNILMLCFSLLLVSAKSQQAVAEQHRPQLHFSPKEKWINDPNGLVYHKGVYHLFYQYYPNSTVWGPMHWGHATSK
DLLTWQHQPVALYPDSLGYIFSGSAVVDVNNTAGFGKGAIVAIFTHHNPKGEQEKRIDFQNQSIAYSLDDGKTWSKY
AGNPVLKNPGIVDFRDPKVMWFEKENKWVMTLATKDRITFYSSPDLKNWSKESEFGETLGAHGGVWECPDLFPLTLN
GKQVWVLLVSINPGGPNGGSATQYFTGEFNGKTFTPFETETKWMDYGTDNYAGVTFSNTANRRILIGWMNNWQYAQA
VPTKSWRGAMTIPRELYLKEVNKKIFLASKPVKEVDKLQVKSTTLSNINIKNSYNLSSKLNAKSSVFQLSLNEIAAN
DFSIVLSNKKGEELVIGYDKTSNQYYIDRSKSGAVDFEKGFAKKHVAPRISTNKNISLRIIVDVASVELFADDGLTV
MTDVFFPAEPYSSISIRSVKGVTLNSIQYMPLKSTMK
>Saeb_GH32_2
MKLGRQILLFIVVFATLMSCKNTNSDTAKNEKTQQVRQTTTEQHRPLFHFSPQSGWMNDPNGMVYFKGEYHLFYQHY
PDSTVWGPMHWGHAVSKDLVHWEHLPIALYPDSIGYIFSGSAVVDEKNTSGFGKNGEPPLVAIYTYHDMAGEKSGKN
NFQTQGIAYSLDNGRTWTKYAQNPVIKNPNVKDFRDPKVMWYEPTKNWILALAVADHIEFYASKDLKNWEKTSEFGK
TEGAHGGVWECPDLFPLKVEGSNTEKWVLIVNIGNGAPNKGSGGQYFIGTFDGKTFKNDNKPTDLLWLDYGTDNYAG
VTWSNSPDNRRTLIGWMSNWQYAQVVPTTTWRSATTIPRDLTLRQTPQGVRLVQKPVKELEILRGSSQTIAASDIQD
FKIIDSTSVAKEIELSFDLSKSNAKTFGVVLSNTKNEKVEIGYDAVTKQFFIDRREAGKKSFSDKFPSRQTAPRFSS
DNTLKMHLYIDVASVELFADDGNPVLTTIFFPNEDFKTAKIFAQNGLSHLLKGQVWALK
>Pdev_GH32_1
MNKKKSVLVAAIFISAVLAKSSNAQQLYREKHRPQVHFSPKEKWTNDPNGMVYHNGIYHLFFQYYPGSTIWGPMHWG
HATSTDLVHWQEQPIALYPDSLGYIFSGSAVVDKNNTSGFGKNGQVPLVAIFTHHDPKGEKEGRNNFQHQSIAYSLD
NGKTWTKYAGNPVLKNPGITDFRDPKVMWYEPQKKWVMTLATKDHITFYSSPDLKNWTKESEFGKELGAHGGVWECP
DLFTLNDHGKQVWVLIVNLNPGGPNGGSATQYFLGAFDGKQFTPFDTTTKWLDYGPDEYAGISWANTGNRKVFLGWM
SNWQYANVVPTETWRNAMTIPRELKLKRIGKEMYVASEPVAELNKIRSKPVTLQNIPVKNSFDVVAKTGKVSLPARV
DLNLEQLRGFSILLSNDAGEEVVIGYDEQQQQYFIDRTKSGKTDFHKDFAGRHVAPRLTSSGKLDCSLIIDVSSLEL
FADGGLTVMTETFFPTEPFKHIRIQSPGNTTIKKLTISSFRSIW
>Msor_GH32_1
MHDTLRELAVADPLRPGYHFTSPAGWLNDPNGTCQRDGVFHLFYQYNPESPQHRSIQWGHATSTDLVSWRDLPIALA
PSEGPDAEGCWSGVLVDDGGRPVIVYSGHAEGRQTACLAYGDESLTSWTKEPGNPVLERPEGVDVTEFRDHAVWREG
GSWRQIIGSGIRGEGGTAFLYSSDDLKEWSLIGPLAVGDADALPDDDPLWTGTMWECIDFFRLRPDGSTAAPDGESG
ETHVLIYSAWDDGRTMHPLAATGSYDGRALSIERTQRLDLGGRHAYAPQTFVDEAGRRILWSWMQEARSDAAMVEAG
WSGAMALPRTLALDESGTIRQSPVAELEGARGARLAWSGGEKSAISRGTRAELAFEARIPEGSGVGVDLFASDDGSE
RTTLRLGRDAAGDVTIELDRSASSLGEGLDTRAHTGVVPGAGETVAVRAFLDGSSLEVFVDGIATTTRVYPTRDDAD
GIRVGARGGAAVANLEGWEMLDQEQPVRQMLPGASEGDGHER
>Ihyd_GH32_1
MTDAPAPRPLLHFAPERNWINDPNGLVFHKGRYHLYYQCNPDGVIHDHLSWGHASSTDLVEWDHHPVAIRNDEAGEI
YSGSAVVDRGNTSGLGSADAPALVALYTQASKHPNHQAQALAHSVDDGLTWTKYAGNPVLDRGTGEFRDPKVFRYEG
PAGSYWVMVAVEATDRQVLLHRSDDLTRWTFLSSFGPERAVDGVWECPDLFPLAVDGDPSDVRWVLLISLNPGGIAG
GSGTQYVIGRFDGVTFTADADATGEAIDWLDFGRDCYAGVTFDGLAREDRTLIAWMSNWDYARYLPFTEGTLQHGMM

ALPRRLSLVRVDGRLRLRQQPITASPGEEQVTQGVLVDAPRAIGELPDAGRITVRIDTQEASGFRLRLNGDSPEGGG
VVLAYDRGARRLTLDRRHGADGIHESFGSVESMPVSGGHVIELVIWIDRASVEVFADGGTRVLTDLIAPQQGRVLAV
EGIGGEVRVDRIAVAPAG
>Npop_GH32_1
MIKTKVFFSLVSIMIASGSSSKAQQLYHERHRPQIHFSPKQKWTNDPNGMVYHNGTWHLFFQHYPDSTIWGPMHWGH
ATSKDLVHWQEQPIALYPDSLGYIFSGSAVVDKNNTAGFGKNALVAIFTHHDPQGEKEKRNNFQNQSIAYSTDEGRT
WTKYAGNPVLKNPGITDFRDPKVCWYEPQKKWIMTLATKDRITFYSSPDLKGWTKESEFGKELGAHGGVWECPDLFQ
LDDDNGKKVWVLIVSINPGGPNGGSATQYFLGDFDGSRFAPVDTNTRWIDYGPDNYAGITWSNTGNRKIFLGWMSNW
LYANIVPATTWRNAMTIARELKLKRTGNEMYVASEPVAELKKIQSKPVVLHNIQVQKSFDVASKAGKITFPARLDLN
FEKNSDYSIVLSNDAGEEVAIGYNKAQQQYYIDRSRSGQTNFQKDFPARHVAPRLSKAGSMDLSLILDVNSVEMFAD
SGLTVMTSIYFPTKPFTHIRIESPENVTIKKLTVSSLKSIW
>FHMF_GH32_1
MKNVLTLIPLLAILSHVALGQAKTGSVPHRPQFHFSPKAHWMNDPNGMLYLNGTYHLFFQYYPDATVWGPMHWGHAT
SKDMLHWQELPIALYPDSLGWIFSGSAVVDVNNTSGFGKDGKTPLVAIFTHHNDKLEKAKSDKFQYQSLAYSLDEGK
TWTKYAGNPVLPNPGITDFRDPKVRWYAPQKKWIMTLATKDRITFYSSPNLKNWTRESDFGKDLGAHGGVWECPDLL
PMTHNGKQAWVLLVSINPGGPNGGSATQYFVGDFDGKTFKPYSTKTKWMDYGTDNYAGVTFSNTGNRTILMGWMSNW
QYARDVPTDPWRSANTVPRELSLREVDKELYLTSTPIKELDKLNGKTTSLSNVPVKGQYDVSAKTGQKSALFKLALS
VPAVNDFSVVLANEQGNELVIGYEKSTNSYYIDRSKSGKTDFEKGFGKRHTAPRIATGNTLSLTLVADVASVELFAD
GGLTVMTDIFFPNQPLNKLYIKSATGISISGLFTPLTKAVQ
>Nchi_GH32_1
MTDVTTPSLSRRFALSSIVALGAAGLGAGARAADTKPSTPEKPTPPQAPDATHKAEPNASHPSPADQPGGTSHPVVK
TESDIFYRPTIHFTPTIGFMNDPNGLVFDGTNFHLYYQYDPFAPYAGHVHWGHATSNDLLHWQDQPIAIPETKDGEA
FTGCAVIDRTNASGLFENAQGGMAALYTRASSHRQAQYLAISHDNGQTFTEHAHNPVLDIGSNSFRDPQVIFHEPTK
QWVMVVAKSRLHQIAFYASIDLAHWVHLSDFGPSGLFGVDYECPNLIEVPLEGGGRRWVLFVSVNPGGPTGGSITQY
FVGEFDGTRFIPDDTVIGLTDFAKDAYAMQVYSNMPQDEAVSIAWLGNWQYCQELPTQSWRGAMTLPRTMTLKRDFA
GWIRLAQTPRHIETLRGTAIPFATRRIAAGSSAQVALPPGTAIELAMSVTVDERPHDLPLGDKGRTGRFIIVFGNEQ
GETLTIGFDAFSGQLWLDRNDLKGFAQPFFTGQFSTVLNPDDRHFDIRVILDASTLEIFANGGLSVGTALIFPAGPL
DFLRLEASGAAATVESLSLYPLKKTMPRDTAI
>Pret_GH32_1
MKKQLELANEAIMKKQLELANEAIKTARKNMDLQFYPQYHLAPYAGWMNDPNGLIYHQGQYHAFYQHHPFSAIWGPM
HWGHATSTDMVHWEHQAIALAPSEDYDRDGCFSGSAISYDNKLYLFYTGHIWLANPGDDSQIIQSQCVAISEDGIHF
EKKGVVLSAPDGYMHFRDPKVWRQDGKWWMVVGARDSQDQGQILLFKSTDLLNWDQNYQVLAKTDDDNVYMWECPDF
FPLGEQFVALFSPQGKKAKNYQYRNLFQNGYLVGNWSPNSSYKISHAFTELDFGQDFYAPQTFLAKDGRRIAIAWMD
MWESHMPTQKHGWSGCFTLPRELTLNEQGKIIAKPIEELKTLRQSASHFPATTLAKNSTIILNENATSCEIELIWDL
QQSPAEKFGFWVGYGVQFYIDNQSQQLTLMRHYPEYTISDSRCAPIPEGHQLKINAFIDHSSLEVFINDGELNFSCR
IYPHQSERDLRLFAINQQAKLEKAIYWEIKKSIE
>Kgeo_GH32_1
MTFSIADAEQELQKRSATLNPRWYPRYHLAARAGWMNDPNGLVWFDGWYHAFYQHHPYSTQWGPMHWGHARSKDLVH
WEHLPVALAPEGPEDKDGCFSGSAVVDGDTLALIYTGHKFHGDPGNDDNLYQVQCLATSRDGIHFERQGQIIDTPAG
LHHFRDPKVWREGDTWYLIVGSRVGDTGQVRLYSSADLRQWQEEGILGEAQEGMGYMWECPDFFMLNGKRILMFSPQ
GLAAEGYRHRNLFQSGYMIGDWLPGQPFVAQSEFIELDHGHDFYAPQSFLTPDGRRIVIGWLDMWESPMPEQQDGWA
GMLSLPREIRLGADNRLQMVPAEEVATLRGSYYPLLAQQLHNQRSHIVDEAEAIELELVWSMKNATAESYGLALGDG
LRIAVDTQAQRLTLERRYPQFALEGTRSVALPAGDRLSLRIFIDRSSVEVFVNDGEACLSSRIYPLEGQRELTLFAS
NGRATLHSGGYWPLDK
>Cfrec_GH32_1
MTSLLSKANEALSQQKDHVNPRWYPRYHLAPPAGWMNDPNGLSWFDGYYHAFYQHYPWQPVWGPMHWGHARSRDMVN
WEHLPIALAPEGPEDKDGCFSGSAVVEGNKLALIYTGHKFDGEAKEENLYQVQCLATSTDGIHFERQGMILDTPRGV
HHFRDPKVWQEGDSWYMVVGARVDDVGEVQLYRSQDLQHWQFANTLGRADDGMGYMWECPDLFPLNDKLVFMFSPQG
IAADGYDYRNLFQSGYLVGEWQDKLHFHVTRAFQEMDHGHDFYAPQSFTTPDGRRIVIGWLSMWESPMPEQADGWAG
MLTLPREVTLDAEQRLRMNPVKELESLRGELHVWPVSELHNRTLMVEEQAHAIEVDLSLDITRSSAEEYGIALGDGL
RVYVDAQAQRLVLDRRYPQHGLSGYRSVPLPVGDLLDLRLFIDSSSVEVFVNHGEYTLSSRIYPEPDDRQLTLFSQN
GHAIFNQGHAWPLSAK
>ERIT_GH32_1

MESLLQAANSAIKENQKEIKPRWYPEYHLAPPVGWMNDPNGLTWFDGYFHAFYQHHPSSPEWGPMHWGHARSRDMVR
WEHLPVALAPEGPEDRDGCFSGSAVVAGEKLALIYTGHKFHGQPVDENLYQVQCLATSSDGVTFQREGMVLDTPEGL
HHFRDPKVWREKEAWYMVVGAKKQGKGEVQIYRSADLRHWDVESAIAGEAICSAYMWECPDLFPLGDKHILMFSPQG
IAAEGYRYRNLFQSGYVLGRWQPGKAFAPEGAFQELDHGHDFYAPQSFTTPDGRRVIMGWLAMWESVMPEKADGWAG
MLSLPRELTLSEDGRVCQHPVKELEALRGTCEQWQPAKLQQQTMILKEHVRAMEVQIEWDTSSSDAERYGLSLGSGA
RIYVDNQSQRIVLERHYPEHNISGYRSVPLPEGDLLRWRLFIDSSSLELFINAGEYTMSSRIYPTDDDRQMALFSHS
GNATVHHACAWPLSAGEPS
>Cfre_GH32_3
MTSLLSKANEALNQQKDLVNPRWYPRYHLAPPAGWMNDPNGLSWFDGYYHAFYQHYPWQPVWGPMHWGHARSRDMVN
WEHLPIALAPEGPEDKDGCFSGSAVVEGNKLALIYTGHKFDGEAKEENLYQVQCLATSTDGIHFERQGMILDTPRGV
HHFRDPKVWQEGDSWYMVVGARVDDVGEVQLYRSQDLQHWQFASTLGRADDGMGYMWECPDLFPLNDKLVLMFSPQG
IAADGYDYRNLFQSGYMVGEWQDNHQFHVTQPFQEMDHGHDFYAPQSFITPDGRRVVIGWLSMWESPMPEQADGWAG
MLTLPREVTLDADLRLRMNPVKELEALRGQLHVWPVSDLNNRTLMVEEQAHALEVELRFDIARSHAEQYGIALGDGL
RVFVDTQAQRLVLDRRYPQHGLSGYRSVPLPVGDLLDLRLFIDSSSVEVFVNHGEYTLSSRIYPEPDDRQLTLFSQN
GHAIFNNGHAWPLAAN
>Kasc_GH32_1
MTALLATANEALNQQKDRINPRWYPRYHLAPPAGWMNDPNGLSWFDGYYHAFYQHYPWQPVWGPMHWGHARSRDMVN
WEHLPIALAPEGPEDKDGCFSGSAVVDGNKLALIYTGHKFDGEAKEENLYQVQCLATSTDGVHFERQGMVLDTPQGV
HHFRDPKVWKEGESWYMVVGARVDDVGEVQLYRSQDLQHWQFASTLAQADDGMGYMWECPDLFPLDDKLVFMFSPQG
IAADGYDYRNLFQSGYMVGEWQDNHQFHVTQPFKEMDHGHDFYAPQSFITPDGRRVVIGWLSMWESPMPEQADGWAG
MLTLPREVTLNADLRLCMNPVKELESLRGQLHVWPVSDLNNRTLMVEEQALALEVELSLDIARSQAEQYGIALGDGL
RVFVDTQAQRLVLERRYPQHGLSGCRSVPLPANDVLDLRLFIDSSSVEIFVNHGEYTLSSRIYPEPDDRQLTLFSQN
GHAIFNNGHAWPLAAK
>Lewi_GH32_1
MKKFFIPMLITLVVASCKTDPATVEEDSTATVTYQEPYRPQLHFSPPAKWMNDPNGMFFYEDEYHLFYQYYPDSTVW
GPMHWGHAVSTDMIHWEHLPIALYPDSLGYIFSGSAVVDRNNTSGLGTADNPPIVAIFTHHDPVGEREKRIDFQYQS
IAYSLDRGRTWTKYKGNPVVPNPGIRDFRDPKVSWHEASQSWIMILAAKDRVHLYRSPNLKDWTFASEFGAELGSHG
GVWECPDLFPLGSEDDMSFQKWVMLVSINPGGPNGGSATQYFIGDFDGTTFTLDPAFAPRVQATPDGNQQAVWLDYG
PDDYAGVTFSGVPPEDGRRLFMGWMSNWSYANMVPTAPWRSAMTLPRTLSLKQMPMGWIIRSRPVLEFEALRTGGFE
VVRGPEQGEKEITPVIPHAPLPVEINLRAMASPTESVGRFGIKLSNDPGEHLSIGYDPQAMQYFIDRRESGPADFSD
AFPKMLTAPRLSDDPAINLRMIIDVSSVELFADYGSSVMTALFFPSEPFNHVSLFAEGGELEWQRIDGRSLQSIW
>Cyeb_GH32_2
MRLSLLITLLLIAGCKQSTDSKQTLAKPMMNEKHRPQFHFTPAANWMNDPNGMVYHNGEYHLFYQYYPDGNVWGPMH
WGHAVTKDLIRWEHLPIALYPDSLGYIFSGSAVVDHKNTSGFGTLDNPPLVSIYTYHDPEQEKAGRIDYQTQGIAYS
LDHGRTWTKYDKNPVLKNPGIKDFRDPKVFWHEGTQAWIMILAVLDHVQLFNSTDLKQWKKISEFGTDQGSHGGVWE
CPDLFPLSVDGKEKWVMLVSLNNGAPNGGSGTQYFIGSFDGINFINENPKDKILWIDYGKDNYAGVTWSDVPKEDGR
RLFIGWMSNWNYANVVPTTVWRSAMTTPRELKLINTAAGSRLISIPVTEIKSLYGKQAQQEAQIITASLDLTSEITR
DSQYELMIDFNTQTNQRFSIELSNAVNEKLVVTYTPVDNTLSVDRTMAGKHDFSSDFGGVHSVQREIQSGISSLRLI
VDCSSIELFADDGLTVITELMFPSEVFGNLKLHSAENETLKINRITITELKRIW
>PHMF_GH32_1
MNFKKLLLAGCCLLAASISKGQTSQADLFGEPYRPQIHFSPKLHWTNDPNGMVYYKGVYHLFFQHYPAGTVWGPMHW
GHATSKDLVHWQEQPIALYPDSLGYIFSGSAVADVNNTSGFGKDGKPPLVAIFTHHDPKGEKEKSDKFQNQSLAYSL
DDGKAWTKYEGNPVLRNPGITDFRDPKVMWYEAGKKWIMTLATKDRITFYSSPNLKEWNLESEFGKDLGAHGGVWEC
PDLFPLSLNGKQYWILIVSINPGGPNGGSATQYFVGDFDGKNFRPRDTTVRWIDYGPDDYAGITWSNTGSRKIFLGW
MSNWIYANEVPTEKWRNAMTIPRELSLKKEGQNILVASTPVKELGVITTGKSVSLSKLDIGKGDLSDKTGSLPGQFE
LKITGKLLKDFSLELSDVNGEKLKIGFDSKVNAYYIDRVNSGQTDFNKEFAARHTAARLTAGKAFDLTLIVDRASVE
LFADNGLTVMSSIFFSKQPFTKLSATAEESLLVDELRITPLKSIWK
>Uadh_GH32_1
MKLNTILYLLTILLAAVSACTEQKKEQNATATTQNKISAAGAYTETHRPQFHFSPPAKWMNDPNGMVYHNGEYHLFY
QHYPDSTVWGPMHWGHAVSKDMVNWENLPIALYPDSLGYIFSGSAVVDQQNTSGFGTKEKPALVAIYTYHLAEGEKA
GRNDYQTQGIAYSLDNGRTWQKYAKNPVIKNPGIKDFRDPKVSWNEAAQQWVMTLAVLDHIEFYGSANLKDWQKLSE
FGKGNVGAHGGVWECPDLFPLTVNGQQKWVLLVSINPGGPNGGSATQYFVGNFDGKTFKNDNSPATTLWVDQGADNY
AGVTWANIPSSDGRRLFLGWMSNWFYANQVPTANWRSAMTVPRELTLQNTVQGIRLVSTPVKELQQLRRETKDLKAA

EITGDLNLSKTYNFTAPLAELDLNVDVSKSQELIVRFANDKGEHLDVGYSVRQQQLFIDRTKAGQTNFEPRFAKKHV
APLTLENGKLRLHLFLDVASVEVFANNGQTVMTDIFFPTEKFTNISLMAEGTASLRESKIYALKSIWR
>Ferr_GH32_1
MKWKIFITLVYSLTLILDVNSIVAQPTVKLKSEMYRPQIHFSPKAYWANDPNGMVYNKGVYHLFYQHHPYSSVWGPM
HWGHATSKDLIKWKHEPIAIYPDSLGTIFSGSAVVDKNNSSGFGKNGRAPLVAIFTHHSMEVEKAGRNDFQNQSIAY
SNDDGKTWTKYAGNPVLKNPGIVDFRDPKVMWYEPQKKWVMTLATKNCITFYSSTNLKSWVKESEFGATIGAHGGVW
ECPDLFTMDDNGKMVWVLIVSINPGGPNKGSATQYFIGDFDGNKFTPLTTDVKWIDYGPDNYAGITWSNTGSRKIFL
GWMSNWMYANTVPTETWRSAMTFPRELKINHVGHEILLASQPAVELSKIQSKPITISNIKITNSFDLAEKIGKVKFP
CRINLSLDEIKDFSLVVSNDIGEKLIIGFDKKENKYFIDRTQSGITGFQKEFAARPVAPRFTDNNKMNISLLIDVSS
VELFADDGLTVMTAIFFPNKPYNQIHIQSTENAVIKNMEYINLKSGR
>RSYSU_GH32_1
MGCNLSRDAASQTGQDVVSRQVTEQHRPQFHFTPPAKWMNDPNGMVYHKGEYHLFYQHHPGGTTWGPMHWGHAVSKD
MVNWKHLPIALYPDENGTIFSGSAVVDVNNTSGLGTKENPAMVAIFTYHSEKLEKAGRNDFQTQGMAYSLDNGRTWK
KYEQNPVLKNPGIRDFRDPKVSWNEEAGQWVMTLAVLDHIEFYGSNNLKEWTKLSEFGKTAAAHGGVWECPDLFPLS
INGKQKWVLLVSLNPGGPNKGSATQYFIGDFDGKNFKSDNPMKTTLWLDYGTDNYAGVTWANVPKSDGRRLFLGWMS
NWLYANEVPTATWRSAMTVPRELKLQQMSEGVRLFSTPVKELQKLRTGTASLKAQEVNGTLNLSERYKLNSSLLELD
LNLDVSQGQEVIIRFSNTKGEHIDIGYSVTGKELYIDRTKSGKMGFKDTFPGKHTAPLTLENGKLRLHLLVDVASVE
VFANDGKTVMTDIFFPTEEFTKVELISTGTTRLQDSKAYSLKSIWNNPA
>Basb_GH32_2
MNQFKCFLIASVFVLHSADTNAQVKGNIYNEPHRPQVHFTPKEKWMNDPNGMLYHKGIYHLFYQYYPDSTVWGPMHW
GHATSTDMIHWKHQPIALYPDSLGYIFSGSAVVDVHNTSGFGKNGKAPLVAIFTQHDPKGEAQGKSDYQNQSLAYSI
DDGKTWIKYANNPVIKNPGIKDFRDPKVMWFEKGKKWIMTLAVSNRIMFYSSKNLKDWIKESEFGENVGAHGGVWEC
PDLFTLEYGGKTVWILIVNLNPGGPNKGSATQYFLGDFDGKSFLPQGTETKWLDYGPDEYAGVTWSNTGKRRLFLGW
MSNWLYANVVPTETWRSAMTIPRELNIIKANNELLIASAPVKEIASIRSKPIVISNVSLSKKIEISKSIKNLSFPCI
INFSIDGSKDFSITLSNELGEELEIGFDKIQNKYFIDRTRSGKTNFQADFAARHFAPRFTSNDKMDISMVIDVSSVE
LFADGGLTVMTEIFFPNKPYSKISVQSADNALMKNFEYIRLNNIWK
>Muri_GH32_1
MHRQVISCFLVLALVACKQEQKKASTPTDKDAIYQEAYRPQFHFSPSEKWMNDPNGLVYNDGWYHLFYQYYPDDIVW
GPMHWGHAMSRDLVHWEHKPIALYPDEHGLIFSGSAVVDKNNTTGFGKNGETPLVAIFTYHSMEGEKEGRTDYQTQG
IAYSLDNGITWTKYEGNPVIPNPGIKDFRDPKVFWHEASSSWIMALVAGDHAQFYTSKDLKDWKYMSDFGKTQGAHG
GVWECPDLFPLPVQGSEEEKWVLLISINPGAPNGGSGTQYFVGDFDGTTFTSQQQEPKWLDYGTDNYAGVTYNNVPS
EDRIFIGWMSNWDYARDTPTQKWRSAMTVPRKLSLHKVEDDYVLANYPIASVGKLTSDMLLSSTQVPAATSDTLVVD
GLHQSEIQLKTASKNMKFLFKNDRDEVLQVQMGLDTLQVDRSLSGQVDFQKDFGARIHKAPIVLPKGELRNLRVLMD
WSSMELFVNEGLLAITEQVFPTEPYDTLIIVNEDSQHPIIIESIKKMESVWQN
>Mfor_GH32_3
MLTTNNRLQQAEEALEKAKEKLNERYRLGYHIMAPANWINDPNGLVQFKGEYHVFYQHHPYDENWGPMHWGHAKSKD
LVHWEHLPVALAPGDACDKDGCFSGSAVDNDGELTLIYTGHHYTDKEKDLFTQNQNIAVSQDGVTFEKAGENPVIAE
PPADSAHHFRDPKVWKHGDSWYMILGNATKDEVGRVILYRSADLRKWVYIGVLAQSEGTLGYMWECPDFFELDGKHV
LLISPQGLEAEGDLYNNRFQTGYLIGEYDYETNKFSHGEFIEMDNGHDFYAVQTLKDDKGRRIAIGWMDMWESNMPT
KEDGWCGALTLPRELTLDKSNKVLMNPVDELTLLRETEHKVCANQSISGNYLAKTNQDLIEIKVEFDLRKTTAKAVG
FKIRGLDQEETVLRYQVTEQKLVLDCTKHGKMEDGIRRTALEAGDRLSLRVFIDRSSIEIFANDGLATMTSRIYPQE
KRLGIELFAEDGDAQVSEFMYWSLKDIWK
>Ntax_GH32_2
MLTTNKLQQAEQAIQNAKQQLNKRYRLGFHIMAPANWINDPNGLVQYKGEYHAFYQHHPYDENWGPMHWGHVKSKDL
VHWEHMPIALAPTESYEKDGCFSGSAVDDNGVLTLIYTGNIFVDKEQDILDQSQCIATSTDGITFTKETANPVISKH
PAEGSGHFRDPKVWKHEDSWYMIIGTRQEDTGKVVLYKSQDLRAWQYVGVLAESDGTLGYMWECPDFFELGGKHILL
FSPQGVGAEGDLYNNLFQTGYFVGDYDYKTNAFKSGSFTELDNGHDFYAVQTLLDDKGRRIAIGWMDMWESSMPTKE
DGWCGALTLPRELTLNENNKILMKPVEELSLLRTQQQKVCTDKSLSGSYLVEATQDLIELKLDFDLTKTSAQAIGLK
IRGTNQDVTKLAFDVEQQKLVLDCSKSGKGEDGVRRTALKADQQLSLRVFIDRSSIEVFANDGEVTMTSRIYPTEER
LGFELFSESGETEVIDFTCWELEDIWG
>Rchu_GH32_1
MTSQAMSPSVEGGPETISAVLPAGSTIHAWIKALHGGAPGKLTAHVDGDPAGAVETSNPHEFEFRLLALESGGETAF
SYDPVTTEVSVLYAFRQSRVLEEGVQLLHVRPSNAAPQTAGSYHFRPPFGWMNDPNGFGRFGGRLHLFYQHYPHSLR

WNNMHWGHAVSDDYLHWTHLPIFLPPSHELAARADGRGGAFSGSAVPLDGAGIRIFFTEHMKDRQPEEQVQFTATSR
DLVKVEAASLILPARPAGLDLTTDFRDPYVFMGPDGKWKMLLGTRDREGGVILLYETDDPAAATGWTFLGMLHRENR
FGMTAAECPCLAPLDGPANDPSTRWALIFGLLTSRDPATGRRNMTIATVGRFDGRSFSVEFEQELDFGTDAYAFQAF
VDDDGPVGIAWLANWTDVSKEIDMATAMTLPRRLALHEGALITPPIAGVKSLRQRRLDAHGLVDGRTVDLANGSVEI
ALALKEPGNAFRLTFEHPEAAVEVLLNDDGLSIPFSVANAKTSPRYIAAGARPSTIRVFLDAGSIEVFADNGRWTGT
KRLPGFTGVSSVRLTALEGNVLAAEIWQLGL
>Rgra_GH32_1
MTIQTGTLSLSRENVSAYLHAGDVIHIWLKALIAETEASVSVTVEDVELARVASARSEEFEFQHIVVSDGGKVVLSY
EPSTTAVSVIYSFDPGRVLEEGIRVLHCTAANAPPQLQSGYHFRPPFGWMNDPNGFGRFGGKAHLFYQHYPHRSRWN
TMHWGHAVSQDYLHWTHLPIFLFPAAELSMRDDGRGGAFSGSAIPVAADAGDEIRVFFTEQVRDRKPEEQIQLSATS
ADQISAGNPSIALPARPEGLGLTLDFRDPYVFKGPDGLWKMLLGSRDRSGGVILLYETSDHDAAGGWTFVGVVHRED
RFGMTAAECPCILPLGDVASDETRWALIFGLLTSRDPATGRRNMSSVTVGRFDGREFQPDFEQELDFGSDAYAFQAF
IDHDGPVGLAWLANWTDVSKSVDFPTAMTLPRRVLLDGSAILTPPLEAVDGLRHKLLDDTSLLAGEAVELVDGAAEI
LLDLTQAGAPFELSLDHPEISLGVRLGEEGLEILFDARTGKPSPRYLAAGAKPAQVRIFIDTGSIEVFADNGRWVGT
KRIPSFAAVRSATMKTGTGGVAAARVWQIKL
>Eadh_GH32_1
MTSQTIPASPALEIIEAEFAAGTVLHLWIKARETGGTAKLFVSVERNDIADPSTRRTEEFEFFAVTLGTGGHTVLAY
DAETTALSVVYAFHPQTVLEEGIRVLHHDMRTAPPQVPAGYHFRPPFGWMNDPNGFGRFAGRGHLFYQHYPHGLRWN
TMHWGHAVSTDLIHWTHLPMFLFPADHLSEKDDGRGGAFSGSAIPISGPEGQEIRVFYTEHVRDRQPEEQIQLSALS
RDGIVAGPSEVVLPVRPEGLNLTTDFRDPYVFKGPDGRWKMLLGSRDRSGGVVLLYETTDPKGAAGWTFLGIIHRED
RFGMTAAECPCMVPLGGKADDPDTRWALIFGLLTSRDPATGRRNLTSVTVGRFDGRKFLSEFEQELDFGSDAYAFQA
FVDGDEPVGIAWLANWTDFSKKDDFPTAMTLPRRVLLDGDAVLTPPSVAAESLRHTQLDEAALAAGETVPLESGAVE
IVLSLPEPGAAFELAFDHPDVKLGVKLDGDGLAILFDAGSGKEPPRYLASGAKPSELRIFLDAGSIEVFADNGRWTG
TKRIPGFSTVRSATLTGAAASAKIWQLKL
>Urhi_GH32_1
MSLQAKSVNQQLETIQAELPAGTVLHLWLKARTAGGQAKLSIAVDGGDAGEPSTRRSAEFEFFAVTLAKGGSATLSY
DAATTSLSVAYAFRPETVMEEGIRLLHSDARTAAPEVPDSYHFRPPFGWMNDPNGFGRFGGNAHLFYQHYPHELRWN
TMHWGHAVSKDFLRWTHLPMFLFPADHLSEDDDGRGGAFSGSAIAVGEDIRVFYTEHVRDRQPEEQIQLSAVSRDGI
VAGPSETVLPLRPEGLDLTTDFRDPYVFKGPDGRWKMLLGSRDKTGGVVLLYETAAADGADGWTFLGIIHREDGFGM
TAAECPCMVPLTARNGETRWALIFGLLTSRDPATGRRNLTSVIVGRFDGRTFTAEFEQELDFGSDAYAFQAFVDGDE
PVGIAWLANWTDFTKKDDFPTAMTLPRRVLLEGDTVLTPPVAAAESLRHQLLDDTALAAGKTVPLGTGAVEIVLDLT
APGAAFELAFDHPDVDLGVKLDGDGLAIVFDARTGKTPPRYIAAGAKPSSLRIFLDAGSIEVFADNGRWAGSKRIPG
FAAARSATLTGTVAKASVWQLKL
>Mend_GH32_1
MSEQKTADETRLRKLRSHVPVNAVLHVWLKAIHPHAPAEMRLSLNEQVFGNIFVQNSEEYEFRMFFVPHGGELECAY
DPETTTVSVAYWFTDAKVLEEGITVLRTHAANAPAPAPGSYHFRPPFGWMNDPNGFGRFNGSAHLFYQHYSHGLTWN
TMHWGHAISEDFLHWRHMPIFLFPSEDLSHRPAKRGGAFSGSAIPLDNSAGIRVFFTESVTDRAPEREIQMMATSQS
LIFAGEAHPLIDNRPELEGLTLDFRDPYVFRGPDGYWKMLLGSRRNEEGVVLLYQTADVTAAGGWEFVDVLHSEGRH
NTQVIECPCLLPLDGPAGDPNTHWVLMCGLMHSHDEATGRRNLTSATVGHFDGTTFTPEFEQELDFVTDNYAFQAFL
DQGTPVGIGWLANWADAAPEIDFPTAMTLPRNLVYDPTDNVLLTPPIEGVEKLRDHLIDETQLAAGELITLETGQAE
IVIELDGSNESFELDLYHNNIEIVIKVDAEGMRLIYLDDIERATPDYVAPGAKPSHIRVFLDIGSVEVFADNGRWTG
TKRIAGFDPIHSLRIRSGGIKTAKVWGLSL
>Urhi_GH32_2
MTSQTISRSDEVGLETISAVLAAGSTIHAWIKALHNGAPGRLTAHVDGDPAGAVETSNPNEFEFRLLTLQSGGETAF
SYDPATTEVSVLYAFLPPRVLEEGVRLLHVSPANAAPEVSGGYHFRPPFGWMNDPNGFGRFGGKLHLFYQHYPHSLR
WNNMHWGHAVSKDYLRWTHLPIFLPPSDQLAARADGRGGAFSGSAIALPGDETGLRIFFTEHMKDREPEEQVQFTAI
SRDLVNVEPASLILPARPAGLGLTTDFRDPYVFSGPDGNWKMLLGTRDREGGVILLYQTDDPAAAAGWTFLGTLHRE
NRFGMTAAECPCMVPLDGPANDPSTRWALIFGLLTSRDPATGRRNMTLATVGRFDGRSFSVEFEQELDFGTDAYAFQ
AFVDNAGPVGIAWLANWRDISREIDMPTAMTLPRRLALQGGALITPPVSGVESLRRRRLDAGGLLDGRTVDLANGSV
EILLTLRQAGSTFRLDLEHPEATVAVQLSDEGLSIPFSVANAKASPRYIAAGARPSAIRIFLDAGSIEVFADDGRWT
GTKRLPGFKGVSAARLIAAEGNVLAAEIWQLGL
>Rgra_GH32_2

MTIQTGTLSLSRENVSAYLHAGDVIHIWLKALIAETEASVSVTVKDVELARVASARSEEFEFQHIVVSDGGKVVLSY
DPSTTAVSVIYSFDPGRVLEEGIRVLHCMAANAPPQLQSGYHFRPPFGWMNDPNGFGRFGGKAHLFYQHYPHSMRWN
TMHWGHAVSQDYLHWTHLPIFLFPAAELSMRDDGRGGAFSGSAIPDGDDEIRVFFTEQVRDRKPEEQIQLSATSADQ
ISAGNPSIALPARPEGLGLTLDFRDPYVFKGPDGLWKMLLGSRDRSGGVILLYETSDHDAAGGWTFVGVVHREDRFG
MTAAECPSILPLGDVASDETRWALIFGLLTSRDPATGRRNMSSVTVGRFDGREFQPEFEQELDFGSDAYAFQAFVDH
DGPVGLAWLANWTDVSKSVDFPTAMTLPRRVLLDGSAILTPPLEAVDGLRHKLLDETSLLGGKTVEFVDGTAEILLD
LTQAGAPFELSLDHPEISLGVRLDEEGLEILFDARTGKPSPRYLAAGAKPAQVRIFIDTGSIEVFADNGRWVGTKRI
PSFAAVRSATMKTGTGGVAAARVWQIKL
>EENS_GH32_1
MTSQTKPASPVLEIVEAELPAGTVLHLWLKARKIGDEAILSVTLDRSEIAGPSTRRAGEFEFFAVTLGTTGRTVLAY
DAETTALSVAYAFHPQTVMEEGIRVLHCDVRTAPPEVPGSYHFRPPFGWMNDPNGFGRFKGLGHLFYQHYPHRLRWN
TMHWGHAVSKDLIRWTHLPMFLFPADHLSEKDDGRGGAFSGSAVPVSGPDGDDIRVFYTEHVRDREPEEQIQLSAVS
RDGIVAGPSEVILPIRPEGLNLTTDFRDPYVFKGPDGRWKMLLGSRDRSGGVVLLYETADLQGATGWTFLDIIHRED
GFGMTAAECPCMLPVGGMADDPETRWALIFGLLTSRDPATGRRNLTSVTVGRFDGRAFTAEFEQELDFGSDAYAFQA
FVDGDEPVGIAWLANWTDFSKKDDFPTAMTLPRRVLLDGGAVLTPPVAAVESLRHRLLDETALAAGETVPLESGAVE
IVLALPEAGAAFELVLDHPDVALGVRLDDEGLAILFDAGTGKPPPRYLASGARPSQLRIFLDAGSIEVFADNGRWTG
TKRIPGFAAARSARLTGAVTGAKIWQLKI
>R13T_GH32_1
MTSPAISPSDEAGIEPIRAVLPAGSTIHAWIRASHGGTSGRLAAHVDGNPAGAVETSNPDEFEFRPMTLERGGETAF
SYDPATTEVSVLYAFLEPRVLEEGIRLLHVRPANATPETPGGYHFRPPFGWMNDPNGFGRFGGKPHLFYQHYPHSLR
WNNMHWGHAVSKDYLRWTHLPIFLFPSDELAARADGRGGAFSGSAIALPGDQAGLRIFFTEHMKDREPEEQVQFTAT
SRDLVNVEPARLILPARPAGLGLTTDFRDPYVFRGPDGKWKMLLGTRDREGGVILLYETDDPAAAAGWTFLGTLHRE
NRFGMTAAECPCMLPLDGPANDPATRWALIFGLLTSRDPATGRRNMTLATVGRFDGRSFSVEFEQELDFGTDAYAFQ
AFVDEGGPIGIAWLANWTDVSKGIDMPTAMTLPRRLALQSGALTTSPVPGVESLRQRQLDAKALLDGRTVDLANGSV
EILLTLRQAGNAFRLDLEHPEATVAVQLNDDGLSIPFSVANAKASPRYIAAGARPSTIRIFLDAGSIEVFADDGRWT
GTKRLPGFKGVGAVRLTAPEGNIIAAEIWQLGL
>Rhal_GH32_1
MPSNQTASTEAVRILHADLPVNSTLHIWMKAISPNAPGVISMHSSNGQFAQLMATNHEEYEFRIYHVFGGGHIELHY
NTETTAISVAYWFTPSDVLETGITVIHTNPGNAPPNLQDGYHFRPPFGWMNDPNGFGRFGGRPHLFYQHYSHGLQWN
NMHWGHAVSSDYLRWRHLPIFLFPSEALRARPDKRGGAFSGSAIPLPNGPGIRVFFTEQEKDRTPEEQIQMTAVSPD
LFSAGHAEVILEQRPENQGLTLDFRDPYVFRGPDGLWKMLLGSQSEEGGVILLYETQDMTAAGGWTYIGKIWVEKRY
ATTAIECPCLLPLDGPANDPATRWGLLYGLMNSTDAKTGRHNLSMVDVGWFDGKTFVKEFEQELDFGTDNYAFQAFI
DGDHGVGIGWLANWADTGYAVDFPTAMTLPRRLLLSDGVLLTPPIGAAESLRSHILDRTRLSAGQVVTFVNGAIEII
FELTEPGAPVVLELDHPHVHLAVEVNENGLLIRHEEEGKASPEYIAAGARPSRIRVFLDYGSIEVFADHGRWTGTKR
IAGFEPVRSAQLKAAPGLVSHATVWALRP
MTSQAISRSDEVGLETISAVLAAGSTIHAWIKALHSGAPGRLTAHVDGDPAGAVETSNPHEFEFRLLTLQSGGETAF
SYDPATTELSVLYAFLPPRVLEEGVRLLHVSPANAAPEVSGGYHFRPPFGWMNDPNGFGRFGGKLHLFYQHYPHSLR
WNNMHWGHAVSKDYLRWTHLPIFLPPSDQLTARADGRGGAFSGSAIALSGGEAGLRIFFTEHMKDREPEEQVQFTAT
SRDLVNVEPADLILPARPAGLGLTTDFRDPYVFSGPDGRWKMLLGTRDREGGVILLYQTDDPAAAAGWTFLGTLHRE
NRFGMTAAECPCMVPLDGPANDPSTRWALIFGLLTSRDPATGRRNMTLATVGRFDGRSFSVEFEQELDFGTDAYAFQ
AFVDNAGPVGIAWLANWTDISREIDMPTAMTLPRRLALQGGALITPPVSAVESLRQHQLDAAGLLDGRTVDLANGSV
EILLTLGQAGNAFRLDLEHPAATVAVQLNDDGLSIPFSVANAKASPRYIAAGARPSTIRIFLDAGSIEVFADDGRWT
GTKRLPGFKGVSAARLTAAEGNIITVEIWQLGL
>Rros_GH32_1
MKKVPFLILCWLAGTLPGTLLAQAVGSEPHRPQVHFTPRAHWMNDPNGMLYYQGTYHLFFQYYPDGTTWGPMHWGHA
TSPDMVRWQEQPIALFPDSLGYIFSGSAVVDVKNTSGFGQPGQTPLVAIFTHHNPVLEKAKRNDVEYQSLAYSLDAG
KTWTKYPGNPVLPNPGIRDFRDPKVRWYEPQQKWIMTLATQDRITFYSSPNLKDWTRESEFGRDVGAHGGVWECPDL
FPLRHQGKDVWVLLVSINPGGPNQGSATQYFLGDFDGKDFKAYTKDIQWMDYGPDNYAGVTFANTGDRTLLMGWMSN
WQYAEKVPTHPWRSAMTIPRELGLQEVNKTLYLTSQPAQELKVLEGESTLLKKIKVPGTYNLTEKIKNPTGVFRLEL
TTPSTGDFAVVLANEQGEEVEIGYDRTAHAYYIDRSKAGKVDFEKGFGKRSTAPRLAQSTQVSLTLVVDASSVELFA
DNGLTVMTSLFFPNKDLTKVLLKAAPGTSVPQLRYTALKPATK
>Aneb_GH32_1

MVTATPADLRKRLAADPHRPRFHFLPPANWMNDPNGFIQWRGQWHLFYQHNPFGPLWGNMHWGHAVSNDLVYWSDLP
MGLAPTPGGPDESGCFSGCAVDNGGVPTFIYTATRGAHHEIQTQCLATSEDGLRTWQKYAQNPVLSEVPPEAKQTRD
FRDPFVWRESDGWWYMVLGSRIQDVGGVIFLYRSQDLIRWEYLHPLLTGDIQQTGVGWECPNFFKLDDRWVLIISIY
TLDGINKVIYYVGSYENQRFTPISEGILDYGVLYAPLSTLDDQNRRVLIGWLRETRSNEAMQAAGWSGVQSIPRVLT
LDAQNRLCMTPIKLDAIRGTKHHFEGRVVDGHFPLDVRGSALDVEAVFEPQTSGQCGIALACSADGRQRLEVVYDAD
TQQLRVREVSPAIQQEQAAPHMLEPQERLHLRILLDGSVVEIIANERTSLTYRIYPTQAADNGVQLIGKQARLHSLD
IWEMVSIWD
>Bgly_GH32_1
MNKRLLQIGAMLTMMLPAGPVYAADPSYYEEKHRPKYHFTPEANWMNDPNGMVYYAGEYHLFYQYHPYGLQWGPMHW
GHAVSKDLVRWEHLPAALSPDDNGTIFSGSAVVDWNNTGGFQTGKEKPLIAIYTQDREGEQVQSIAYSNDKGRTWTK
YPGNPVIPNPGKKDFRDPKVFWHEETKRWVMVLAAGDRIMIYTSPNLKQWTFASEFGEGQGSHGGVWECPDLFQLSV
DGNPHKKKWVMQVSVGNGAVSGGSGMQYFVGSFDGTSFKNENPPDKTLWTDYGKDFYAAVSWSDIPSSDGRRLWLGW
MSNWQYANDVPTSPWRGATTIPREVKLKAFSNGVGIVQTPVKELRSTRGASQGWRHQIISSAGCSLPANLSGDSYEI
NAEFQVSAGSASEFGFKVRQGENQYTKIGYHRGNGTLFVDRSQSGNVSFHPSFNTGKQVAPLKPVNGKVKMRIFVDR
SSVEVFGNDGQRVLTDLIFPDQSSNGLEVYASNGFVKLNSLTIHPLKKIWGQSPFKSNLAGWTTVNGLWADTIDGKQ
GRSDGDSFITSSASGTDFTYESDITIKDGNGRGAGALVFRADQDVKNGYLANVDAKHDVVKFFKFENGSAAVIAEHK
TPIDVNKTYHLKTVARGANFKIYLDDHLVIDAHDSTFKHGTFGLNVWEATAVFQDVYVDQ
>Ansb_GH32_1
MKILYAVTTILLFIVLITSCSTPDNEDKPANTEDEVTPSETNMPEPTSEPEPTPEIDAQATIESNAAAAQRQMEDAG
IYTEKYRPQFHFSPMRGWIGDPDGMLRYQDTYHVWWWGHAESKDLVHWENQIPSAMAGDDGSFIYFSGSVVVDENNS
SGFGDGTKPPMIALYTMFFNDVKPEVQGLSVSHDYANFVFYKGNPVLEAEPKFFRDPMVFWHDETGRWIMAIALPEK
HQVSFYASDNLKEWEHLSDFGPAGALNGWWEVPDLYQLPVDGDPNNTRWVLQIGRGPNRVQYFIGDFDGTQFTLNPE
ENGTTTNWLDYGPDYYAVRTFRDYDHVEDRIVTFGWMGNWQYANNVPTSWGKGALSLPRELELRTYEGELQIVQRPI
PALEQLRGDEVTLENVELTDTRPLSEFTPALNSYELDLTIRITDPDTRFGIRLAESDEHAFTLGYDTSTSALFLDRM
KGERAFLGDEFPKQLTAPLQPQADGTIRLHIFVDQSSVELFANEGEVTMTALMFPEPDSTGITLFVEGGTAVLERLT
AWELTSIWQGILE
>Arsb_GH32_1
MSLLALACALVVAPQDRQGDIPVMDFRGANYGSWTMTGDAFQPGPASGDLLVRLEIVGVRDGRCISSEVEGDQPRGT
LISPPITIERDFITFLIGGGDYERCTCLDLVVDGEVVRSATGRNSDTLAPQSWDVRAWRGKKAHIVVVDKASGDWGH
INVDRIVQTDQPETPPVSVGPLYKEALRPGFHFTARQWTMDRLEPAQRQEGWINDLNGLIYYDGEWHLFAQRWAKCW
LHAVSRDLVHWTELQPAFWEESEGSGVQSGTCVIDYHNTSGLSVDPKKPPMVAFWSRFDNRSQCISFSLDHGRTWTR
YAGNPVFEHPERDPKVFWYDPPGGGAGHWVMVMYGEESYHILTSPDLLHWKDEGHPIPQSFECPDFFQLPLDGDKSR
KQWVLVQGSGNYSIGMFDGHEFKEQPGRFACDLGPNFYATQSWHNTDTGDGRRIQVAWMRGPHFPGMPFNQQVSFPC
ELSLRSTPAGPRLFRSPIKEVSKLEAKSRLWPETRLEPGEVLQLSKKGEMFRLIADVDIPEGANLIFALRGEAVTLS
SRGMVSGEAHGNIQGQVKRVEILLDRASIETFVNDGELSSTRTVLPRSNGLFLKADGGPIVLRSLRVAPLSSSWR
>Caeb_GH32_1
MQYYKPVGDFFAGDCMPFFHDGVFHLYYLLDENHHKALNGLGGHQWAHASTRDLIHWQHHPLALAIDAEFEKSICTG
SLFYHQGIYYAFYATRLANWHQHLSWATSHDAIHFEKQPPNPLASPPVGYSPLHFRDPHPFQDPETGLFHLLVTAML
EEPALAGRGGCLAQLTSPDLKRWTMQAPFLIPGYADAPECPDAFFWNGWYYLLFSNHLTAHYRMAQHPLGPWLCPPI
DTLDGTLARVMKTAAFGPDRRLGVAWLGTRTDDKDDGKPQWGGHILFRELVQHEDGTLGSKFVPEMLAVDQRQPPLA
WAEVGTGGIQQADQLHLRAWQSLQALPVTNLPPNFYLRMRLHPQPGSVRFGLRLRGSGNFASGYDLTIHIPERMVRL
HNEAIYAVNGLDQPFTLEAVVKDDIIDVCIDQRRCIVNRCPELQGDRLFVFAHNAEVYVDEIEIRPWKTAVG
>Bgly_GH32_2
MNKRLLQIGAMLTMMLPAGPVYAADPSYYEEKHRPKYHFTPEANWMNDPNGMVYYAGEYHLFYQYHPYGLQWGPMHW
GHAVSKDLVRWEHLPAALSPDDNGTIFSGSAVVDWNNTGGFQTGKEKPLIAIYTQDREGEQVQSIAYSNDKGRTWTK
YPGNPVIPNPGKKDFRDPKVFWHEETKRWVMVLAAGDRIMIYTSPNLKQWTFASEFGEGQGSHGGVWECPDLFQLSV
DGNPHKKKWVMQVSVGNGAVSGGSGMQYFVGSFDGTSFKNENPPDKTLWTDYGKDFYAAVSWSDIPSSDGRRLWLGW
MSNWQYANDVPTSPWRGATTIPREVKLKAFSNGVGIVQTPVKELRSIRGASQGWRHQIISSAGRSLPANLSGDSYEI
NAEFQVSAGSASEFGFKVRQGENQYTKIGYHRGNGTLFVDRSQSGNVSFHPSFNTGKQVAPLKPVNGKVKMRIFVDR
SSVEVFGNDGQRVLTDLIFPDQSSNGLEVYASNGFVKLNSLTIHPLKKIWGQSPFKSNLAGWTTVNGLWADTIDGKQ
GRSDGDSFITSSASGTDFTYESDITIKDGNGRGAGALVFRADQDVKNGYLANVDAKHDVVKFFKFENGSAAVIAEHK
TPIDVNKTYHLKTVARGANFKIYLDDHLVIDAHDSTFKHGTFGLNVWEATAVFQDVYVDQ
>Bhal_GH32_2

MKKKLIQVVILFTMLFTMALPVSAADSSYYDEDYRPQYHFTPEANWMNDPNGMVYYAGEYHLFYQHHPYGLQWGPMH
WGHAVSKDLVHWKHLPIALSPDDKGTIFSGSAVVDWNNTSGFQTGKEKPLVSIYTQDHEDGQVQSVAYSNDKGRTWT
KYSGNPVIPNPGKKDFRDPKVFWYEKDKKWVMVLAAGDRILIYTSKNLKQWTYASEFGQGQGSHGGVWECPDLFELP
VDGNPKQKKWVMQVSVGNGAVSGGSGMQYFVGSFDGTTFKNENPSDKVLWTDYGKDFYAAVSWSDVPSSDGRRLWLG
WMSNWQYANDVPTSPWRSAMTIPREVKLKAFSKGIRVIQTPVTELQSIRGAAQKWKNKIISPESSNVLAGISGDAYE
MNAEFQVNPGSASEFGFKVRTGRNQYTKVGYHQKSGKLFVDRSQSGNVTFNPTFNTGKQAAPLKPVNGKIKMRIFVD
RSSVEVFGNDGQQVLTDIILPDRSSKGLELYAVNGRVKVNSLTIHPLNKAWGASPFISNLTGWTTVNGTWADTIDGK
QGRSDGDSFMMSSASGADFTYESDIIIKDGNGKGAGALVFRSDKDAQNGYVANVDAKHDLVKFFKFEDGIASVIAEH
QTPIDINKKYHLKTVAHGAHFKIYLDDRLVIDAEDSAFSEGQFGLNVWDATAAFQNVNAKAD
>Bpar_GH32_1
MKKRMIQMGIIGAMMFPEAFSAAAADPDYYNEDHRPKYHFTPEANWMNDPNGMVYYAGEYHLFYQYHPYGLRWGPMH
WGHAVSKDLVKWEHLPVALYPDEKGTIFSGSAVVDRHNTTGFQTGTEKPLVAIYTQDRDGEQVQSIAYSNDKGRTWT
KYSGNPVIPNPGKRDFRDPKVVWHKQTKKWVMVLAGGDRILFYTSPDLKHWTYASEFGEGEGSHGGVWECPDLFELP
VEGRPNETKWVMQVSVGDGAVSGGSGMQYFVGSFDGTTFKNENPQNRVLWTDYGKDFYAAVSWSDIPRSDGRRLWLG
WMSNWQYANDVPTSPWRSAMSIPRELKLKAFSEGFRIVQAPVTELKSIRGASQMWKNKIISPRNRNFLKALSDNAYE
INAEFQVNTGTAAEFGFKVRTGENQYTKIGYSKNSASLFVDRSQSGNVSFNPNFNTGKHAAPLQPVGGKVKMRIYVD
RSSVEVFGNDGRQVITDIILPDRSSKGLEVYASNGFVKLNSMTVHPLKNVWGTSPFQSNLTGWTTVNGVWADTIDGK
QGRSDGDSFILSSAKGTDFTYEADVTVKDGNGRGAGALVFRADKDVQNGYLANVDAKHDVVKFFKFENGSASVIAEH
RTPIETGRKYHLKAVARDSNFHIYLDGRLVISARDSTFTEGAFGLNAWDATAVFQHVYADR
>Bhay_GH32_1
MKKRMIQMGIIGAMMFPEAFSAAAADPDYYNEDHRPKYHFTPEANWMNDPNGMVYYAGEYHLFYQYHPYGLRWGPMH
WGHAVSKDLVKWEHLPVALYPDEKGTIFSGSAVVDRHNTTGFQTGAEKPLVAIYTQDRDGEQVQSIAYSNDKGRTWT
KYSGNPVIPNPGKKDFRDPKVIWHEKTKKWVMMLAGGDRILIYTSPDLKQWTYASEFGEGEGSHGGVWECPDLFELP
VEGRPNETKWVMQVSVGDGAVSGGSGMQYFVGSFDGTTFKNENPPNRVLWTDYGKDFYAAVSWSDIPQSDGRRLWLG
WMSNWQYANDVPTSPWRSAMSIPRELKLKAFSEGLRIVQAPVAELQSIRGASQTWKNKIISPQNGNFLKALSGDAYE
INAEFQVNTGAAAEFGFKVRTGENEYTKIGYSKNSASLFVDRSQSGNVSFHPNFNTGKHAAPLQPVGGKVKMRIYVD
RSSVEVFGNDGRQVITDIILPDRSSKGLEVYASNGFVKLNSMTVHPLKKVWGASPFQSNLTGWTTVNGVWADTIAGK
QGRSDGDSFILSSAKGTDFTYEADVTIKDGNGRGAGALVFRADKDVKNGYLANVDAKHDVVKFFKFENGSASVIAEH
RTPIEAGRKYHLKAVARGSNFNIYLDDRLVISARDSTFADGAFGLNAWDATAVFQHVYAKR
>Blic_GH32_1
MKKRMIQMEIIGAMMFPEAFSAAAADPDYYNEDHRPKYHFTPEANWMNDPNGMVYYAGEYHLFYQYHPYGLRWGPMH
WGHAVSKDLVKWEHLPVALYPDEKGTIFSGSAVVDRHNTTGFQTGTEKPLVAIYTQDRSGEQVQSIAYSNDKGRTWT
KYSGNPVIPNPGKRDFRDPKVIWHEQTKKWVMLLAGGDRILIYTSPDLKRWTYASEFGEGEGSHGGVWECPDLFELP
VEGRPNETKWVMQVSVGDGAVSGGSGMQYFVGSFDGTTFKNENPPNRVLWTDYGKDFYAAVSWSDIPKSDGRRLWLG
WMSNWQYANDVPTSPWRSAMSIPREVKLKAFSEGLRMIQAPVAELQSIRGASQTWKNKIISPRNGNLLKGLSGDAYE
INAEFQVNTGAAAEFGFKVRTGENEYTKIGYSKNSASLFVDRSQSGNVSFNPNFNTGKHAAPLEPVAGKVKMRIYVD
RSSVEVFGNDGRQVITDIILPDQSSKGLEVYASNGFVKLNSMTVHPLKKVWGASPFQSNLTGWTTVNGVWADTIDGK
QGRSEGDSFILSSAKATDFTNEADVAIKDGNGRGAGALVFRADQDVKNGYLANVDAKHDVVKFFKFEDGSASVIAEH
RTPIETGRKYHLKTVARGANFYIYLDDQLVISARDSTFTDGAFGLNAWDATAVFQHVYAKR
>Blic_GH32_2
MKKRLIQMGIIGAMMFPEAFSANAADPDYYNEDHRPKYHFTPEANWMNDPNGMVYYAGEYHLFYQYHPYGLRWGPMH
WGHAVSKDLVKWEHLPVALYPDEKGTIFSGSAVVDRHNTTGFQTGAEKPLVAIYTQDRDGEQVQSIAYSNDKGRTWT
KYSGNPVIPNPGKRDFRDPKVIWHEQTKKWVMMLAGGDRILIYTSPDLKQWTYASEFGEGEGSHGGVWECPDLFELP
VEGRPNETKWVMQVSVGDGAVSGGSGMQYFVGSFDGTTFKNENPPNRVLWTDYGKDFYAAVSWSDIPQADGRRLWLG
WMSNWQYANDVPTSPWRSAMSIPRELKLKAFSEGLRIVQAPVAELQSIRGASQTWKNKIISARNGNFLKALSSDAYE
INAEFQVNTGAAAEFGFKVRTGENEYTKIGYSKNSASLFVDRSQSGNVSFNPNFNTGKHEAPLQPVGGKVKMRIYVD
RSSVEVFGNDGRQVITDIILPDRSSKGLEVYASNGFVKLNSMTVHPLKKVWGASPFQSNLTGWTTVSGVWADTIDGK
QGRSDGDSFILSSAKGTDFTYEADVTIKDGNGRGAGALVFRADQNVKNGYLANVDAKHDVVKFFKFENGSASVIAER
RTPIEAGRKYHLKTVARGANFNIYLDDQLVISARDSTFTDGAFGLNAWDATAVFQHVYAKR
>Hgum_GH32_1
MNRFYSALPLVGIVAAALCLGGCQKETAARPAAPPATTASNALTCTPRAENPDYQFYPLGDNSAPIGDVMPYYDAAT
GQFNVYYLKDIWNDATHQRHPWYELKTSNFYGYSGLSAGQILACSTNPCQQDYALGTGHIVKKGSTYYAFYTGHNPN
YPSSCVTRKEGIMLATAPGLNQQFTKSSGFATIYAPTGQGFDEQDNFRDPFVYFDDASATYYLSAAARKNVSGTWRG

VVVKYTSPDLLSWTYQGLLYDGGPDNFFMLEAPELFRMGSTYYLLFSDIDSKNLLYRKSSSLSGPWTSPSGAARFEG
KGIYAAKTALDQYGDRYLFGWTNRLAGNADAGAWQWGGNLVVHKIYQRSNQDLAVTIPHTLKNHAEAQNEPLVKNSQ
WGNVTSLSPGPHSYTLSSPADKDVANVLFEPLAPRRYKLHCTVSYSSAAKDFGFLLGACDGYDDVYSLRFVPGQQRF
SFDKVKRSALTSSTAPVTDVPFPMSPNTEYNVDIVFENSMVVVYLDNVAALSARIYRAPGTSWGIFVDNSTATFRNI
TVSKP
>HSP3_GH32_1
MSHANEVHFVNDQQRPAFHMMPPANWMNDPNGPIYYKGEYHLFYQYNPAGAFWANMHWGHAVSKDLVHWEHLPIALT
PSESYDKDGVFSGCTVINESEPTIIYTGIPDLEKKIEVQCIATSKDMINWEKSNKNPIIDALPGELETTGFRDPFVW
KEEDNWYMILGSGIKGVGGAILLYRSKDLREWEYINPIYIGEDEELENMWECPNFFKLEDKYILVVSPYGKVVYWIG
SYENYKFIPEKKGYIDLGDSYYAPNSFIDDKGRRIMWGWLKEQRSKEVQKAAGWSGVMSVPREIFLHKDGSLGMKPI
EELKVLRGEHKCLSDIPISADSTNLLDNVQGDRLEIIAEFEKGDASSIGLVVRKSPDNSEYTEIICNIAEQNLSINT
KNSSLAKEVDKGVFGDCYNFNDDTDGLVKLHIFIDKSVIEVFVNERQCVTARVYPTCGDSLGLGLLAWEGKARLKDI
DIWWMKKI
>Bsub_GH32_1
MKKRLSQAIIMFTLLFTMAFPVEAADSSYYDEDYRPQYHFTPEANWMNDPNGMVYYDGEYHLFYQYHPYGLQWGPMH
WGHAVSKDLVTWKHLPVALAPDDKGTIFSGSAVVDRNNTSGFQTGEEKPLVAIYTQDREGHQVQSIAYSNDKGRTWT
KYAGNPVIPNPGKKDFRDPKVFWYEKENKWVMLLAAGDRILIYTSKNLKQWTYASEFGQEQGSHGGVWECPDLFELP
VDGNPNQKKWVMQVSVGNGAVSGGSGMQYFVGDFDGTHFKNENPSNKVLWTDYGKDFYAAVSWSDIPSSDGRRLWLG
WMSNWQYANDVPTSPWRSAASIPRELKLKTLTGGVRVVQTPVKELQTIRGTSKKWKDQTISPTSQNVLAGLSEDAYE
LHAEFQVTPGSVAEFGFKVRTGGNQFTKVGYDRKNAKLFVDRSESGNVTFNPTFNTGKQTAPLKPVNGKLQMRIFVD
RSSVEVFGNEGQQVITDIILPDRSSNGLELYAANGGVKVKSLTIYPLKKVWGTSPFISNLAGWKTVNGTWADTIEGK
QGRSDGDSFNLSSATGSDFTYESDITIKDGNGRGAGALIFRSDKDAKNAYLANVDAKHDLVKFFKFEDGAASVIAEY
KTPIDVNKQYHLKTEAEGDHFKIYLDDRLVIDANDSTFSEGQFGLNVWDATAVFQNVNKEP
>Psoi_GH32_1
MNSAYPTVNPASVSLPASKSIINGDSQLLRATVSPANASNKDVIWTSSNNAVASVVNGKVTAKSVGTATITAKTRIG
NFTANSTITVTAEPAHDGLINGDFDDNLSGWTILSGNAFSSQDVTDANDWGWGGPFNQHGNYHLWGGKDGGDSQTGS
IRSQKFILGGNGQINFLVGGGNNIYDEYVALVRSSDGKELFKVSGGDKESYTRVHWDAADYIGVECYIKVVDNATGG
WGHINVDDFQVPVQPPTTTNITNPDFETGSLSGWTIVSGTAFNNLDVCTDTTYWTPARPFNHNGTYHMWGFKDGGDS
QVGVMKSSTFTLYGTGWIDFLIGGGQDPNKDVPGQDLTTLYVALVRASDGAELIKETAYNDEAYTRIFWDASAHIGE
QVYIKVVDNLTGDWGHINVDDFHVYNTASDITTADRYEQYRPQLHFSPDRKWMNDPNGLVYYDGEYHLFYQHNPTGT
TWGPMSWGHAVSTDMVNWKDLPIALEPDSIGFIWSGSVVVDTNNTSGFQMGMEKPMVAMFTHEKGSVQVQSIAYSND
RGRTWAKYAGNPVITQPQGVEVFRDPKVIWHAPTNKWVMVISAGDRVQFYTSTDLKVWNYADEFGSTHGSHGGTWEV
PDLFSLAVDGDGANTKWVLTASISNGAPAGGSGTQYFIGTFNGTTFTNSNSPGTVLWADYGSDFYAGITFSDIPTAD
NRRIMISWMNNWNYSQRIPTSIWRSSMTVPRELKLTDTGGGAVRMTQTPVAELSGIRGTSNSWTNQTITPGSNLLSS
VTGNTVEIVAEFQNNTATATEYGFKVRKGGDNFTTIAYNKTNSKFFVDRTASGESHFNGTFAAKHEVTLSADNNKIK
MRIFVDRSSIEAFGNDGKVSITD
>Ppro_GH32_1
MKATGYNDEGYTRLFWDASAYIGEQVYIKVVDNATGAWGHINVDDFHVYNTASDITTADRYEQYRPQLHFSPDRKWM
NDPNGLVYYDGEYHLFYQHNPTGTTWGPMSWGHAVSTDMVNWQDLPIALEPDANGFIWSGSVVVDTNNTSGFQTGTV
KPMVAMFTHESGGTQVQSIAYSNDKGRTWTKYASNPVITMPAGLTVFRDPKVFWHAGTSKWVMVISAGDRVQIYTSS
NLKSWTYASEFGSTHGSHAGTWEVPDLFPLAVDGNGANTKWVMTASISNGAPAGGSGTQYFVGSFNGTTFTNDNSAG
TVLWADLGSDFYAGISFSDIPAADGRRIMLSWMNNWNYGQSIPTSIWRSSMTIPRELKLTDTGGGAVRMTQTPVAEL
SGIRGTSNSWTNQTITPGSNLISGVTGNTVEIVAEFQNNTATATEYGFKVRKGGSNFTTIAYNKANSKFFVDRTASG
ESHFDSSFAAKHEVTMSAENNKIKMRIFVDRSSIEAFGNDGKVSITDQIFPDLAKNGLELYSTGGNVTLNSLTIYQL
GATTELLNHDFESGDLTGWTVVSGTAFSNKVVSEDRNWGWGGLFNKSNVYHLWGYKAGDAPVGVLKSANFIVGGSGK
IDFLIGGGNNLTNLYVALVRVSDGVELLKATGANSEALSHVIWDAAAYKGVNCYIKIVDNNTGGWGHLNVDDINVPV
K
>Deab_GH32_1
MAFNRFRPTSHFIPPHSWSNDPCGAVYIPETKEYRICYQWNPGTTRGGNSAWGMARSKDLVTWEDCSPALRNGATYD
RLGVFSGSIVSRIVDGRRVLFLFYTSVSAVPIHWSLPYLDGCESQSVAFSTDYGNSWQRYEHNPLMTIPPKLERTTG
WRDPFVSKWEPMSKLLGVDSSMNYMMIASGERGRGPQLLLYQSNDLLDWQPTCTLLDVEANAKITPTSELNFGMNFE
CASFFTIGERHYIIVGIEEDKTSKRHDGHALLWLSGTLSLQNGKPKFEITSHGQLDHGIAYAAHIFRDAEGRILQLG
WANEAANREAVEKQGWAGLLTHPKELYEISVPISQVEGDMDRWNIDESSVTMTTLGMRLAPQVSQLRQSQTAPITSL

EGLRSLRSTNYDISATFRNTSGTERFVFNVRESPSSAEVTKIVFDLANHRISIHRSRSSLENLGESSPDEGKFFLLS
GEDLDVRIVVDNSIIEVFANERFAVTSRVYPSLESSKGVSWDFGGFAENGVEVSCWEGLKGAWPTRRESAAGIEEVM
TEKVVRDEKHRMTANELELVHA

Figure S3. Three dimensional structures of the identified proteins predicted using AlphaFold-2. The names of the proteins were given based on the first one letter of the genus followed by the first three letters of the species' scientific name plus the order number of the gene identified from the same species. For example, Focc-GH32-2 means the protein sequence derived from the second GH32 gene identified from the thrip *Frankliniella occidemtalis*. All animal species were given in Figure S1 along with their classification. All protein sequences were listed in order in Figure S2.

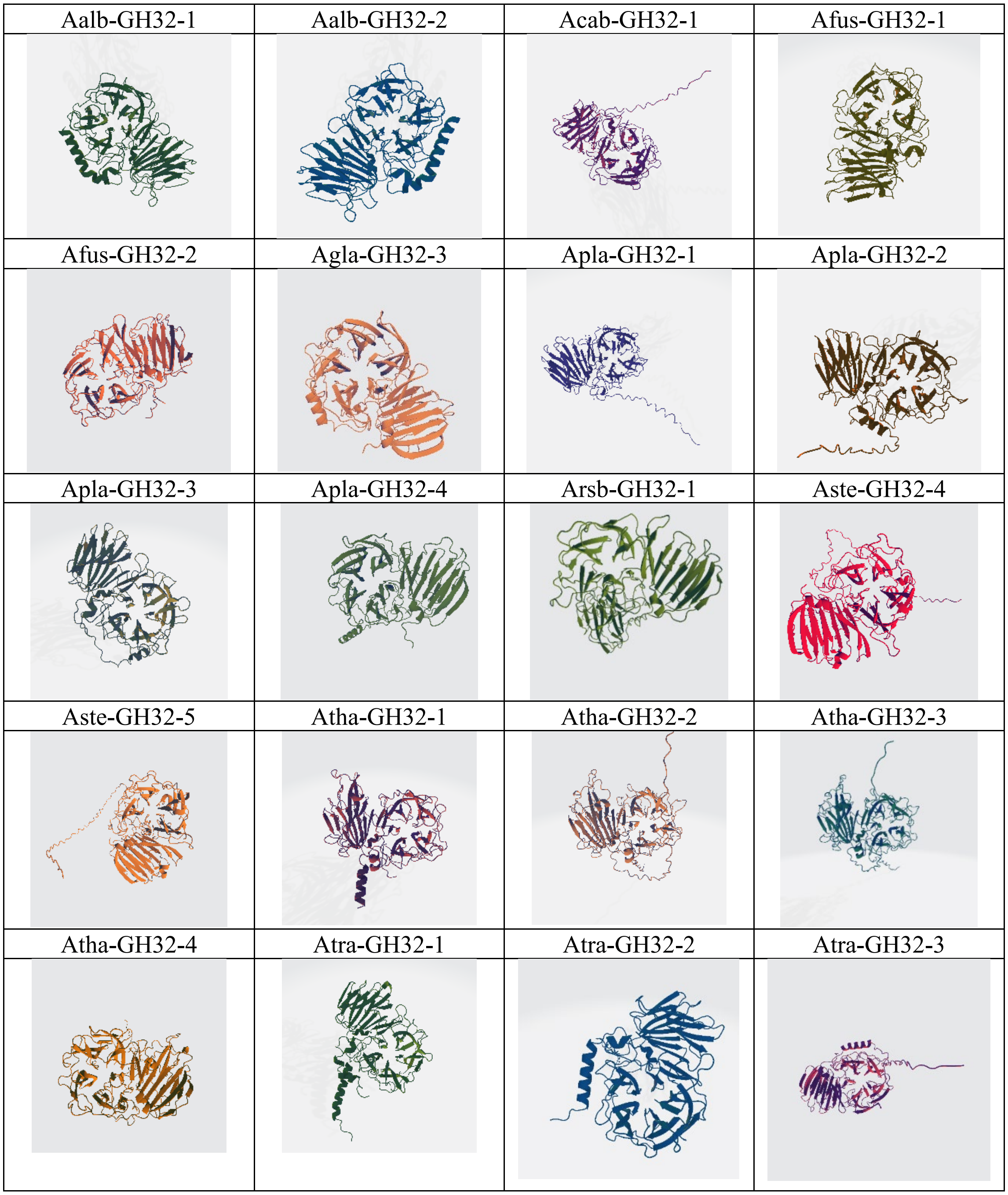

Atra-GH32-4	Atra-GH32-5	Avag-GH32-1	Aven-GH32-1
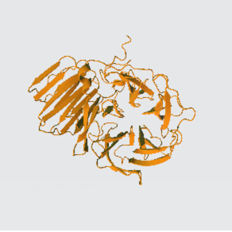	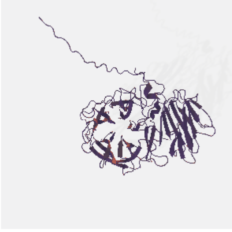	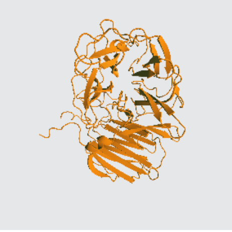	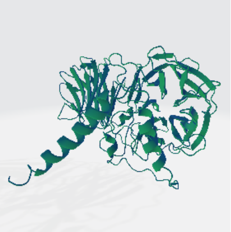
BAFS09-GH32-1	Basb-GH32-1	Basb-GH32-2	Bbom-GH32-1
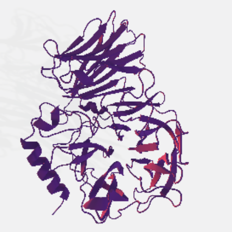	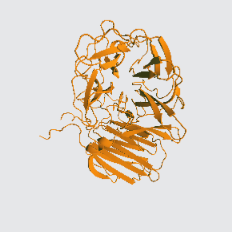	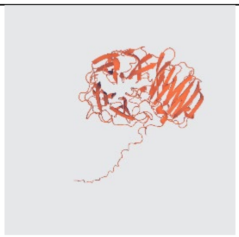	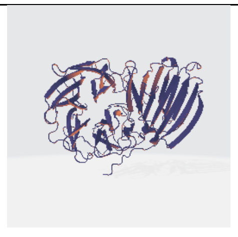
Bbre-GH32-1	Bbru-GH32-1	Bcop-GH32-1	Bcop-GH32-2
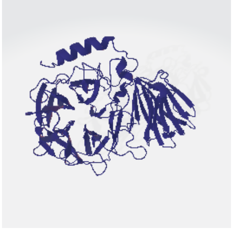	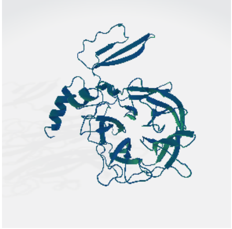	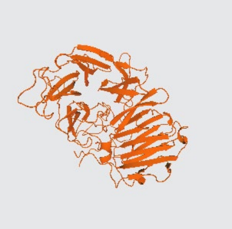	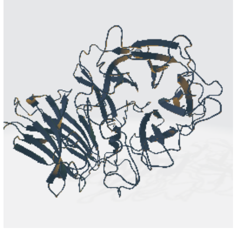
Bgly-GH32-1	Bgly-GH32-2	Bhal-GH32-2	Bhay-GH32-1
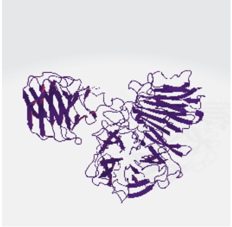	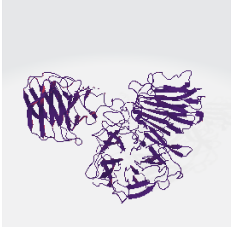	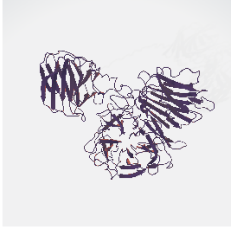	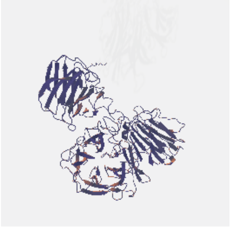
Bint-GH32-1	BJCM-GH32-1	Blic-GH32-1	Blic-GH32-2
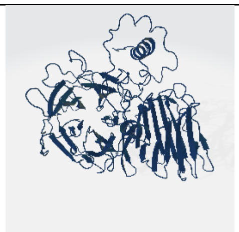	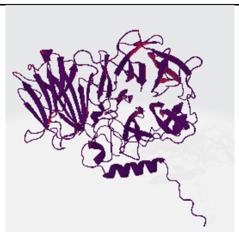	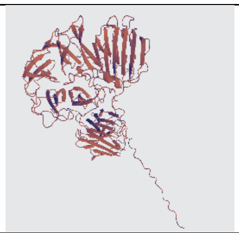	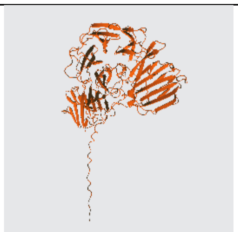
Bmrc-GH-1	Bodo-GH32-1	Bodo-GH32-2	Bodo-GH32-3
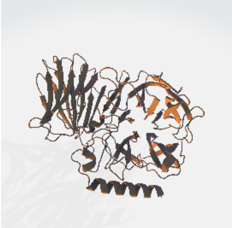	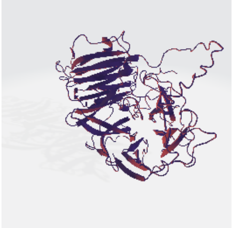	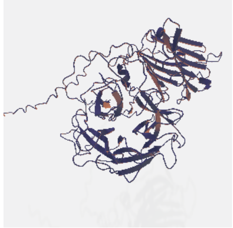	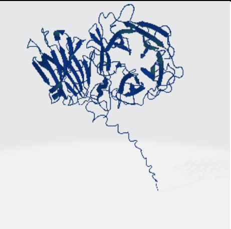
Bodo-GH32-4	Bodo-GH32-5	Bpar-GH32-1	BRHF-GH32-1

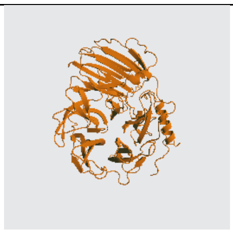	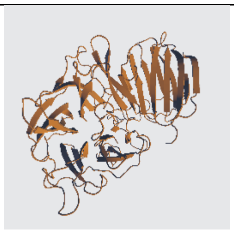	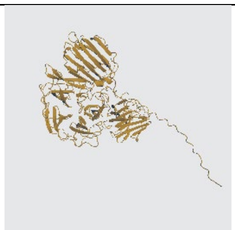	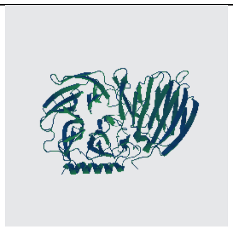
BRHFB-GH32-1	BSA1-GH32-1	Bsub-GH32-1	Btab-GH32-2
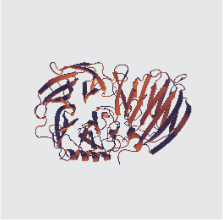	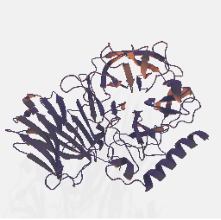	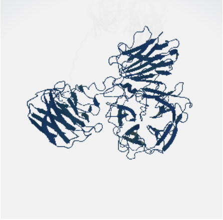	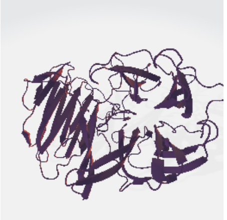
Calb-GH32-1	Cdar-GH32-1	Cfla-GH32-1	Cfre-GH32-1
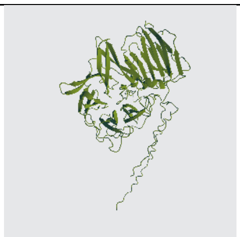	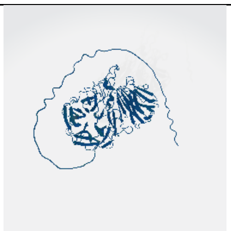	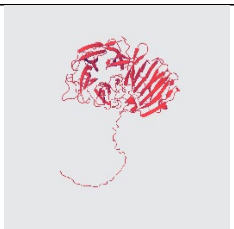	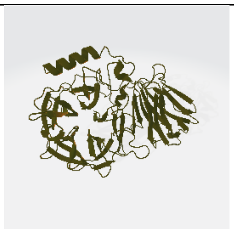
Cfre-GH32-2	Cfre-GH32-3	Cfrec-GH32-1	Cnas-GH32-8
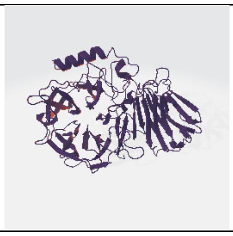	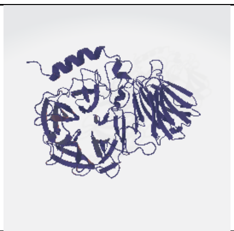	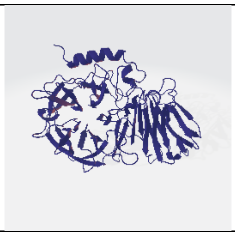	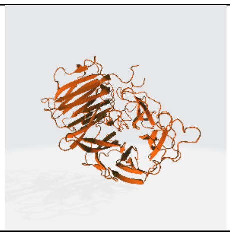
Cnas-GH32-9	Cyeb-GH32-1	Dalg-GH32-1	Dann-GH32-1
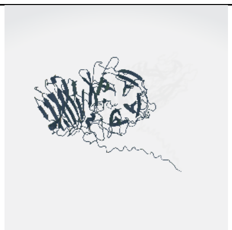	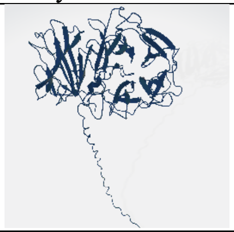	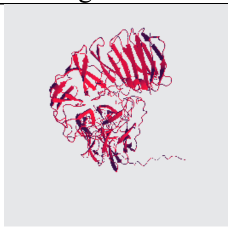	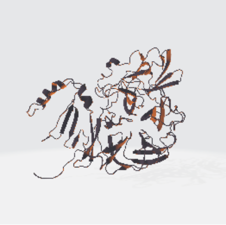
Dann-GH32-2	Dann-GH32-3	Dcar-GH32-1	Dcar-GH32-2
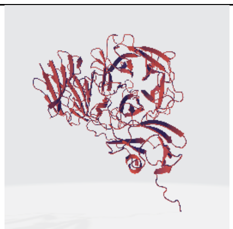	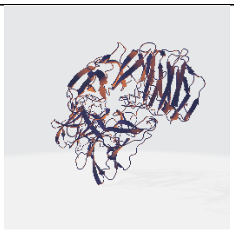	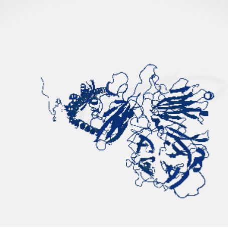	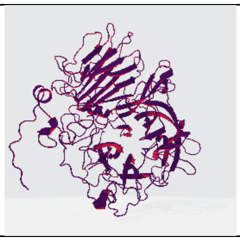
Dcar-GH32-3	Dcar-GH32-4	Dcar-GH32-5	Dcar-GH32-6

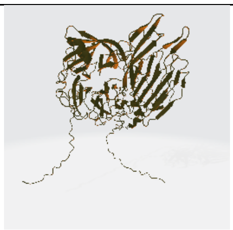	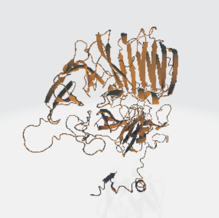	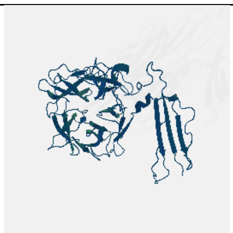	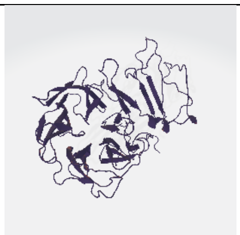
Dcar-GH32-7	DHDWA-GH32-1	DHDWB-GH32-1	Dmin-GH32-1
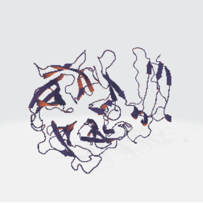	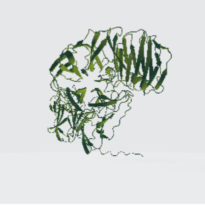	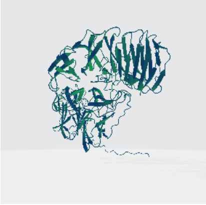	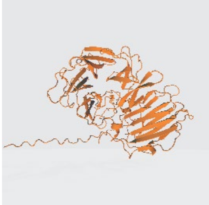
Dple-GH32-1	Dple-GH32-2	Dple-GH32-3	Dque-GH32-1
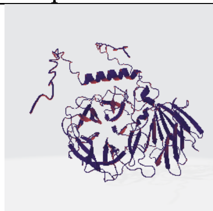	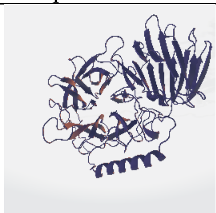	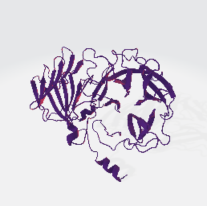	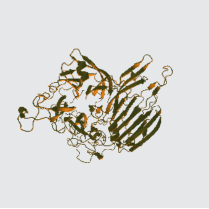
Eadh-GH32-1	Eann-GH32-1	Eann-GH32-2	Eann-GH32-3
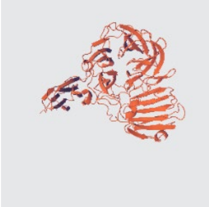	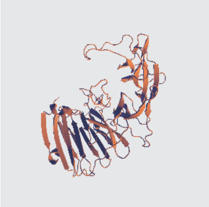	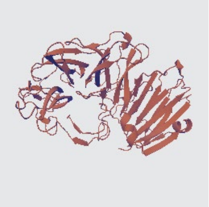	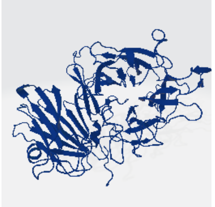
EENS-GH32-1	ERIT-GH32-1	Fdor-GH32-1	Focc-GH32-1
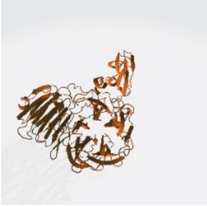	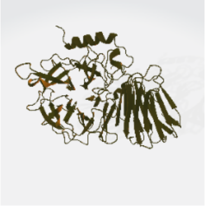	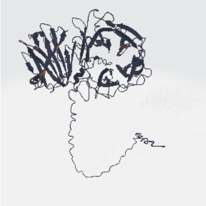	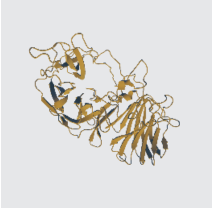
Focc-GH32-2	Focc-GH32-3	Focc-GH32-4	FQH1-GH32-1
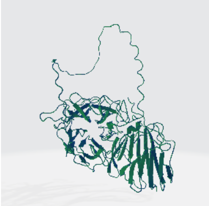	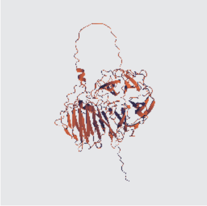	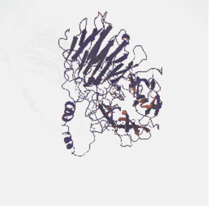	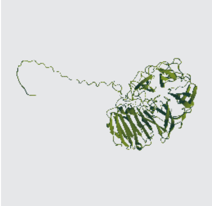
Fsar-GH32-1	Gros-GH32-1	Gros-GH32-2	Gros-GH32-3
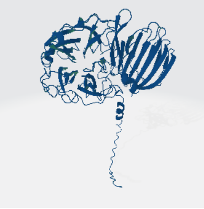	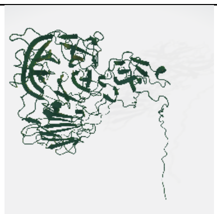	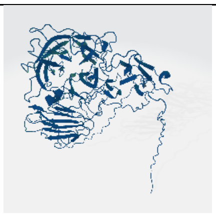	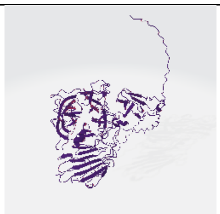

Gros-GH32-4	Gste-GH32-1	Halv-GH32-1	Harm-GH32-8
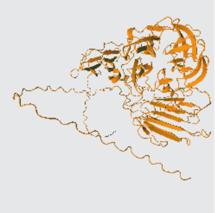	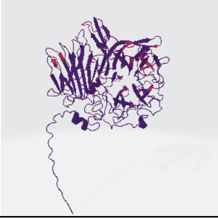	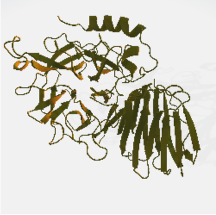	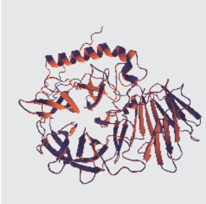
Harm-GH32-9	Hduj-GH32-2	Hduj-GH32-3	Hduj-GH32-4
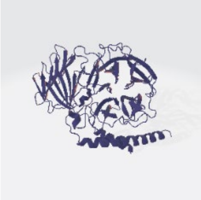	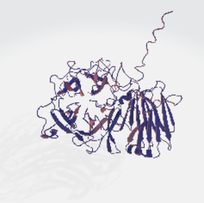	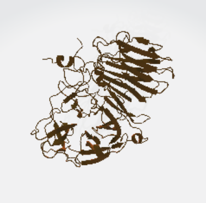	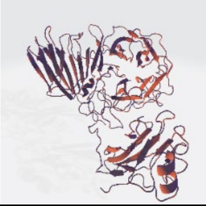
Hgly-GH32-1	Hgum-GH32-1	Hhat-GH32-1	Hisb-GH32-1
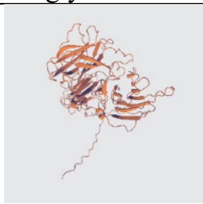	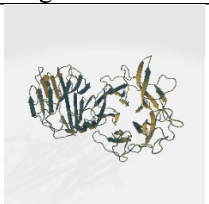	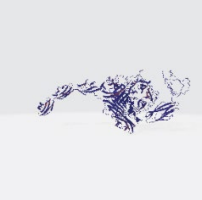	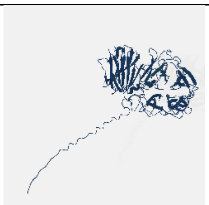
Hrad-GH32-1	Hrad-GH32-2	Hrad-GH32-3	Hrad-GH32-4
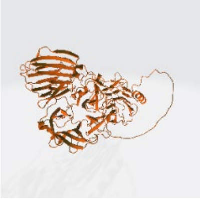	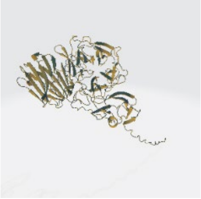	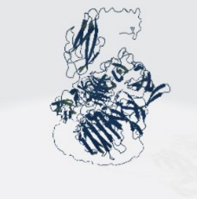	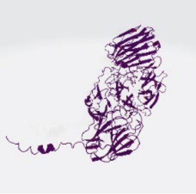
Hrad-GH32-5	Hsch-GH32-1	Hsch-GH32-2	HSP3-GH32-1
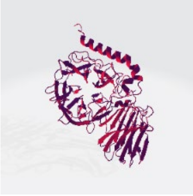	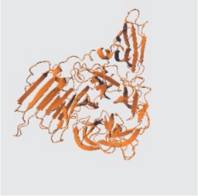	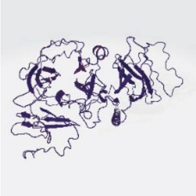	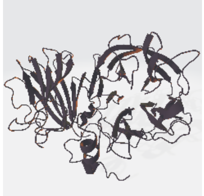
Hspor-GH32-1	Ilum-GH32-3	Kego-GH32-1	Kmar-GH32-1
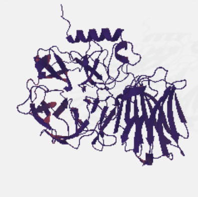	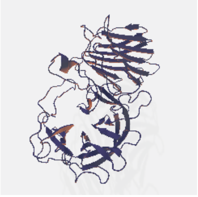	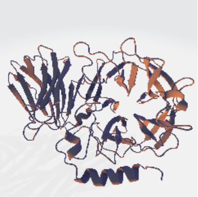	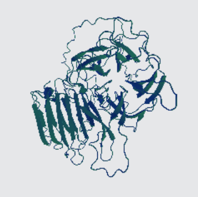
Lani-GH32-1	Lcai-GH32-2	Lcai-GH32-3	Ldel-GH32-1
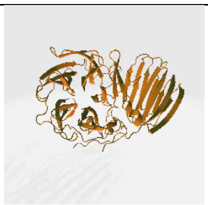	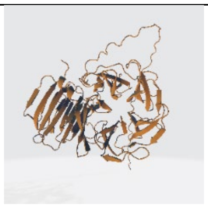	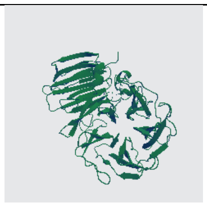	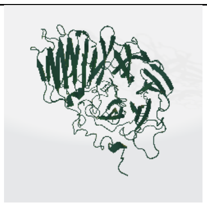
Ldel-GH32-2	Ldel-GH32-3	Ldel-GH32-4	Ldel-GH32-5

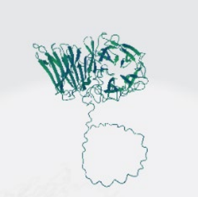	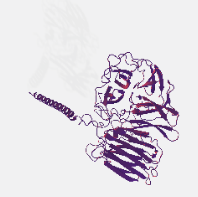	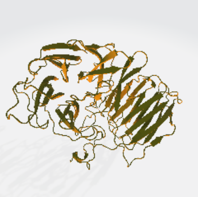	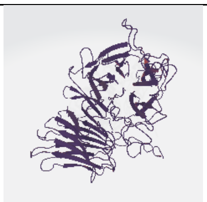
Llut-GH32-1	Lore-GH32-1	Lore-GH32-2	Lore-GH32-3
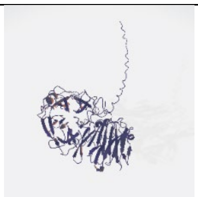	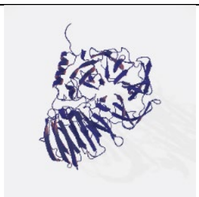	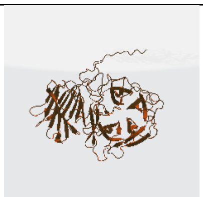	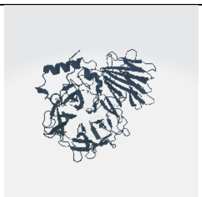
Lore-GH32-4	Lpla-GH32-1	Luva-GH32-1	Mabs-GH32-1
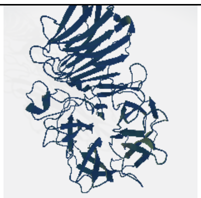	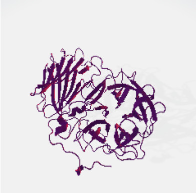	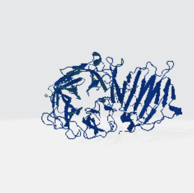	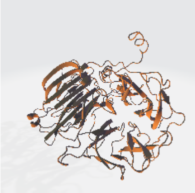
Mdes-GH32-1	Mdes-GH32-2	Mdes-GH32-3	Mdes-GH32-4
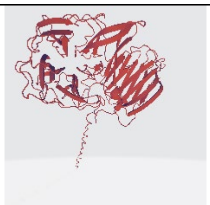	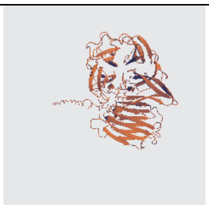	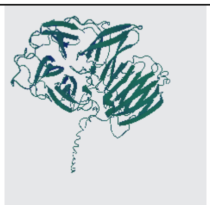	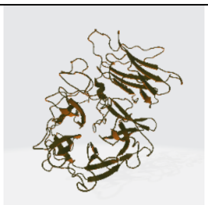
Mdes-GH32-5	Mdes-GH32-6	Mdes-GH32-7	Mdes-GH32-10
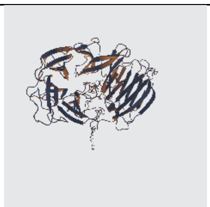	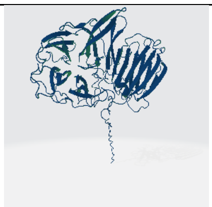	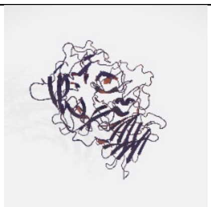	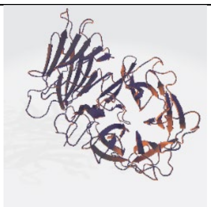
Mend-GH32-1	Ment-GH32-1	Ment-GH32-2	Ment-GH32-3
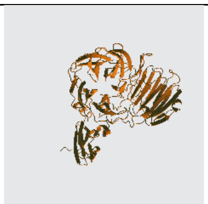	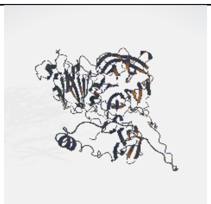	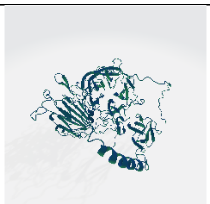	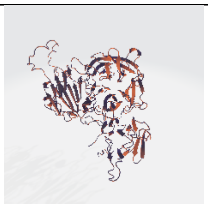
Ment-GH32-4	Ment-GH32-5	Ment-GH32-6	Ment-GH32-7
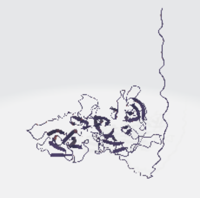	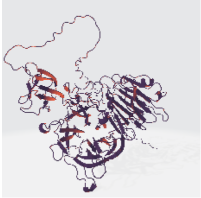	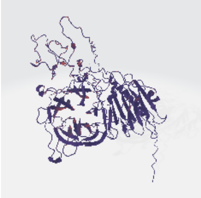	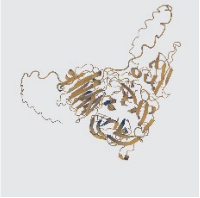
Mfor-GH32-1	Mfor-GH32-2	Mfor-GH32-3	Mlon-GH32-1

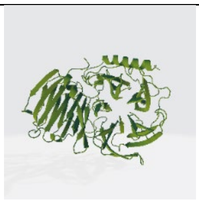	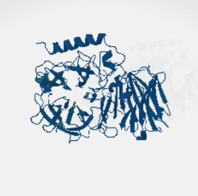	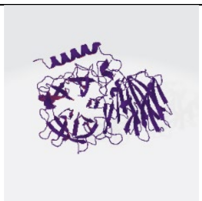	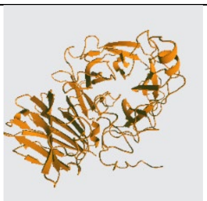
Mora-GH32-1	Mqua-GH32-4	Mqua-GH32-6	Mqua-GH32-7
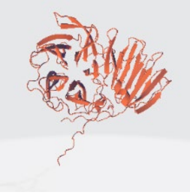	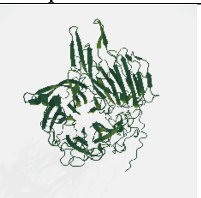	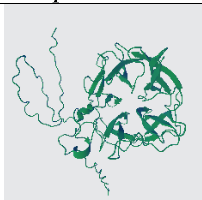	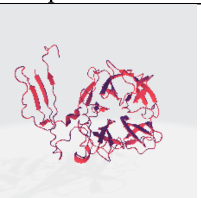
Mseq-GH32-1	Msex-GH32-1	Msex-GH32-2	Msex-GH32-3
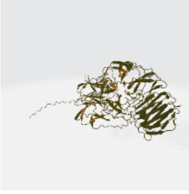	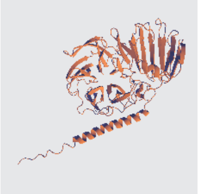	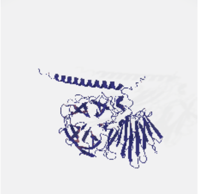	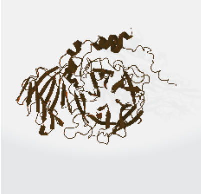
Msex-GH32-4	Msor-GH32-1	MTW1-GH32-1	Nchi-GH32-1
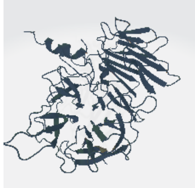	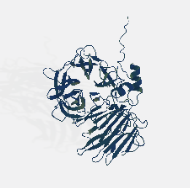	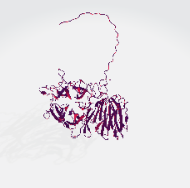	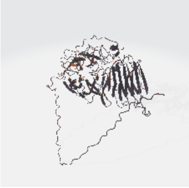
Ocin-GH32-1	Ocin-GH32-2	Ofur-GH32-3	Ogib-GH32-1
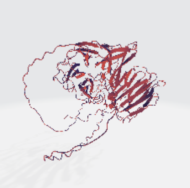	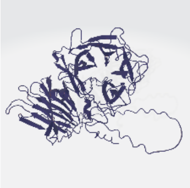	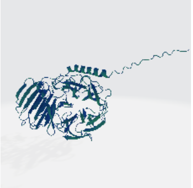	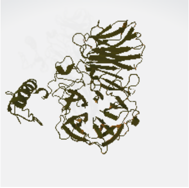
Ostr-GH32-1	Paeg-GH32-1	Paeg-GH32-2	Paeg-GH32-3
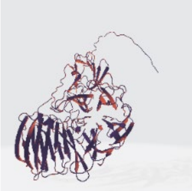	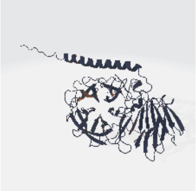	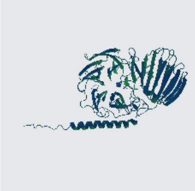	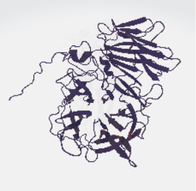
Paeg-GH32-4	Pgor-GH32-1	Phie-GH32-1	Pmeg-GH32-1
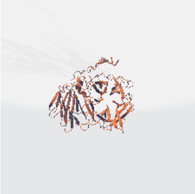	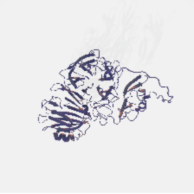	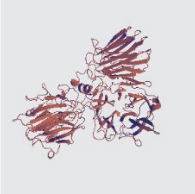	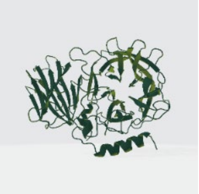
Pmeg-GH32-2	Pmeg-GH32-3	Pmeg-GH32-4	Ppol-GH32-1

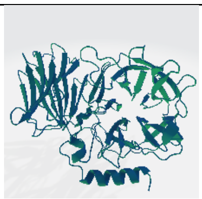	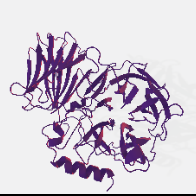	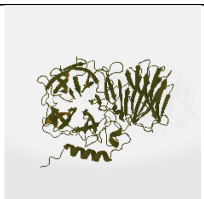	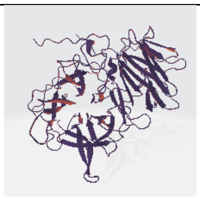
Ppol-GH32-2	Ppol-GH32-3	Ppro-GH32-1	Preg-GH32-1
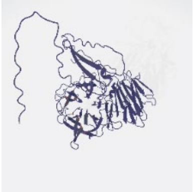	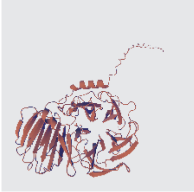	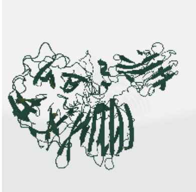	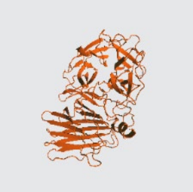
Pret-GH32-1	Psim-GH32-1	Psoi-GH32-1	R13T-GH32-1
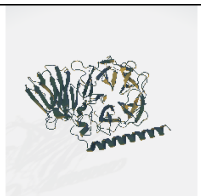	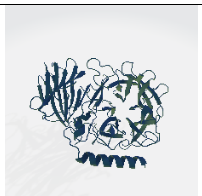	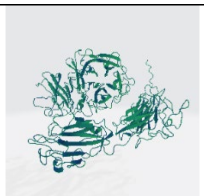	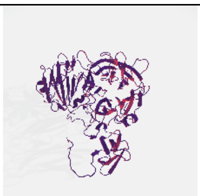
Rban-GH32-1	Rchu-GH32-1	Rfer-GH32-1	Rgra-GH32-1
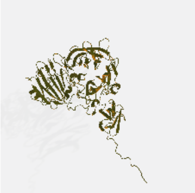	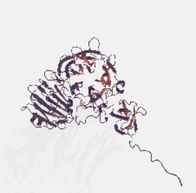	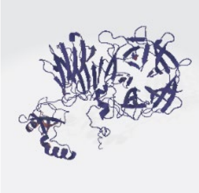	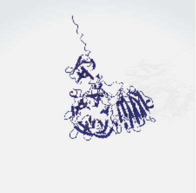
Rgra-GH32-2	Rhal-GH32-1	Rsli-GH32-1	Rsor-GH32-1
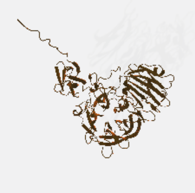	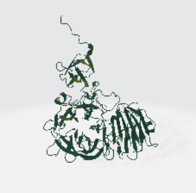	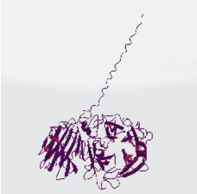	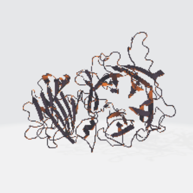
Rsp1-GH32-1	Rsp1-GH32-3	Rsp1-GH32-5	Rsp2-GH32-1
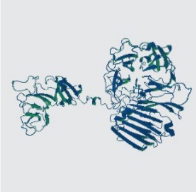	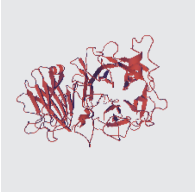	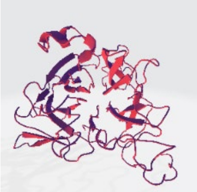	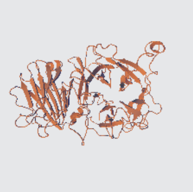
Rsp2-GH32-2	Rsp2-GH32-3	Rsp2-GH32-4	Rsp2-GH32-5
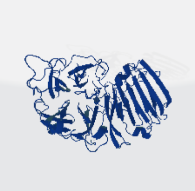	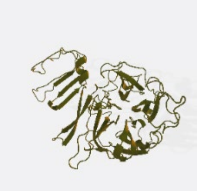	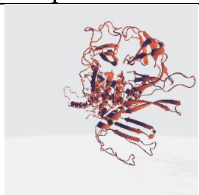	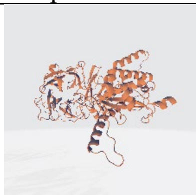
RSYSU-GH32-1	Rvar-GH32-1	Saeb-GH32-1	Saeb-GH32-2

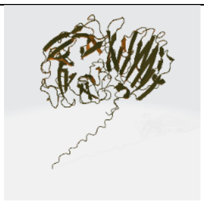	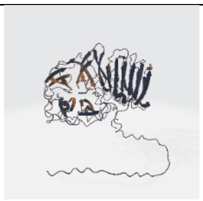	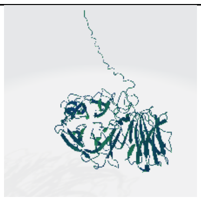	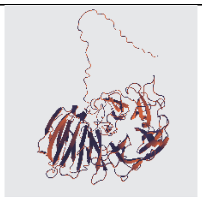
Salb-GH32-1	Salb-GH32-2	Salb-GH32-3	Scur-GH32-1
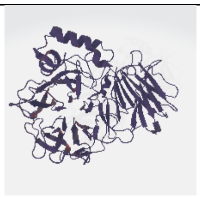	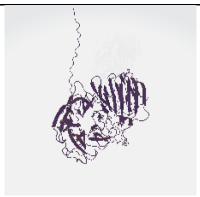	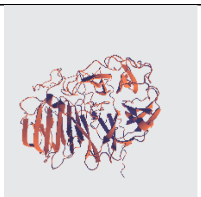	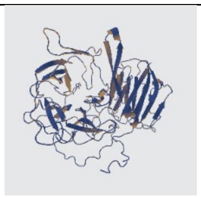
Scur-GH32-2	Scur-GH32-3	Slev-GH32-1	Smos-GH32-1
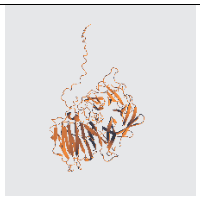	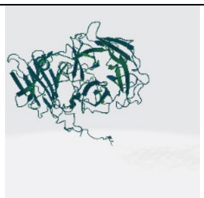	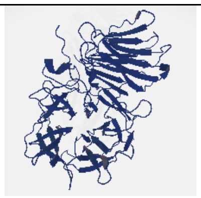	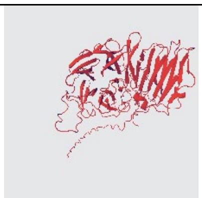
Smos-GH32-2	Smos-GH32-3	Smos-GH32-4	Socc-GH32-1
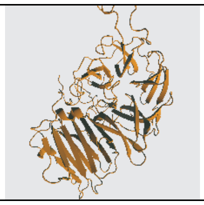	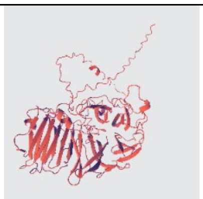	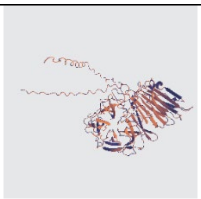	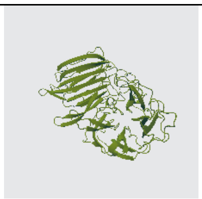
Sory-GH32-1	Sory-GH32-2	Sphy-GH32-1	Ssim-GH32-1
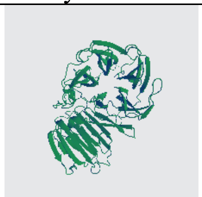	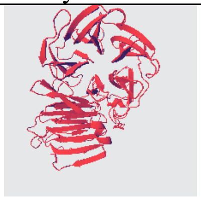	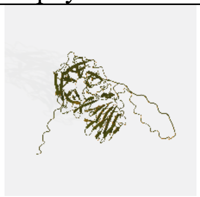	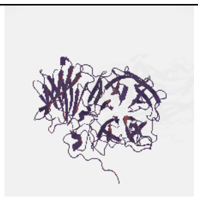
Taes-GH32-1	Taes-GH32-2	Taes-GH32-3	Tcin-GH32-1
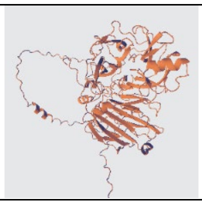	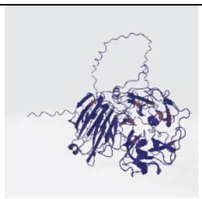	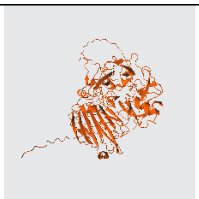	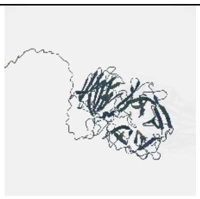
Tcin-GH32-2	Tclap-GH32-1	Teva-GH32-1	Tmar-GH32-1
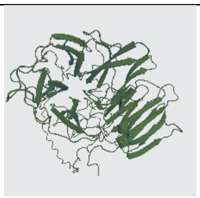	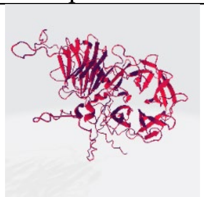	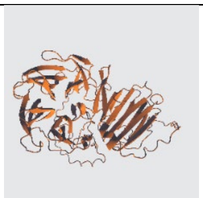	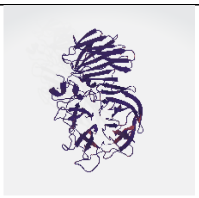
Tmel-GH32-1	Tmel-GH32-4	Tni-GH32-1	Tni-GH32-2

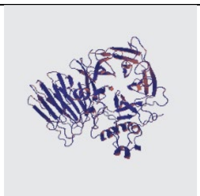	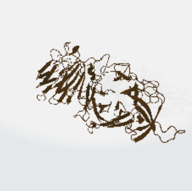	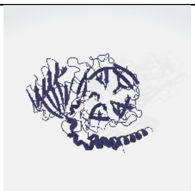	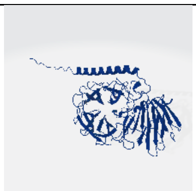
Tni-GH32-3	Tni-GH32-4	Tpal-GH32-1	Tpre-GH32-1
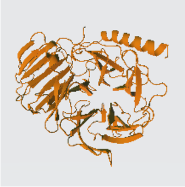	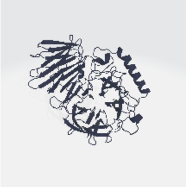	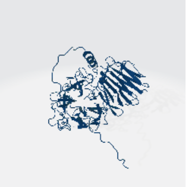	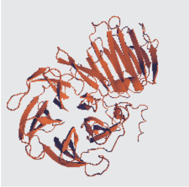
Tqin-GH32-1	Tqin-GH32-2	Turt-GH32-2	Tvul-GH32-1
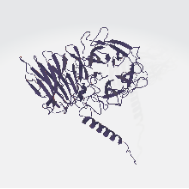	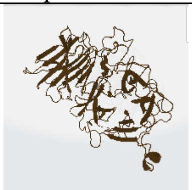	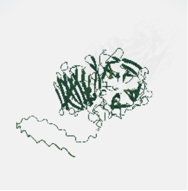	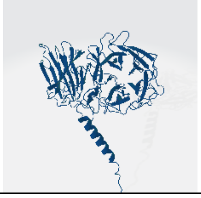
Tvul-GH32-2	Uadh-GH32-1	Urhi-GH32-1	Urhi-GH32-2
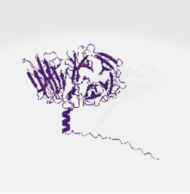	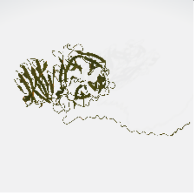	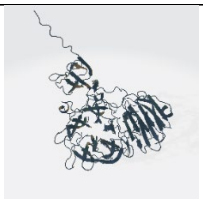	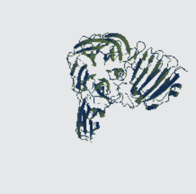
Vago-GH32-1	Yper-GH32-1	Yper-GH32-2	
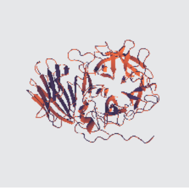	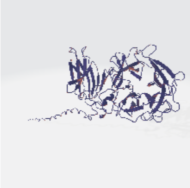	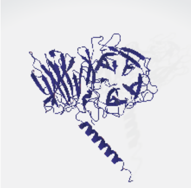	
